# Metal–ligand cooperativity enables zero-valent metal transfer†

**DOI:** 10.1039/d4sc07938h

**Published:** 2025-01-22

**Authors:** Martin-Louis Y. Riu, Jing-Ran Shan, K. N. Houk, Matthew Nava

**Affiliations:** a Department of Chemistry and Biochemistry, University of California Los Angeles California 90095-1569 USA mnava@chem.ucla.edu houk@chem.ucla.edu

## Abstract

Group 13 aminoxy complexes of the form (L)E(TEMPO)_3_ (TEMPO = 2,2,6,6-tetramethylpiperidine 1-oxyl; L = THF (tetrahydrofuran) or Py (pyridine); E = Al, Ga, In) were prepared and structurally characterized. The complexes (THF)Ga(TEMPO)_3_ (1·THF) and (Py)In(TEMPO)_3_ (2·Py) are shown to heterolytically cleave H_2_ under mild conditions (3 atm, 20 °C, *t* ≤ 1 h). 1·THF reacts reversibly with H_2_ to form a formal H_2_-adduct that bears a Ga(iii) hydride site and a protonated TEMPO ligand with concomitant loss of THF, consistent with Ga(iii) and TEMPO functioning as Lewis acid and base, respectively. Conversely, 2·Py is reduced by H_2_ to form an intermediate dimer complex of monovalent {In(TEMPO)}_2_, which undergoes further reactivity with H_2_ to form elemental indium as determined by powder X-ray diffraction. Treatment of 2·Py with H_2_ and Ph_3_PSe forms binary InSe, in addition to Ph_3_P and TEMPOH, demonstrating that 2·Py functions as a molecular source of zero-valent indium under mildly reducing conditions. Computational studies support an intramolecular metal–ligand cooperativity pathway in the heterolytic cleavage of H_2_.

## Abstract 2

Group 13 aminoxy complexes, (L)E(TEMPO)_3_ (TEMPO = 2,2,6,6-tetramethylpiperidine 1-oxyl; L = THF (tetrahydrofuran) or Py (pyridine); E = Al, Ga, In), display ambiphilic reactivity with H_2_ and function as synthons for the preparation of materials.

## Graphical abstract

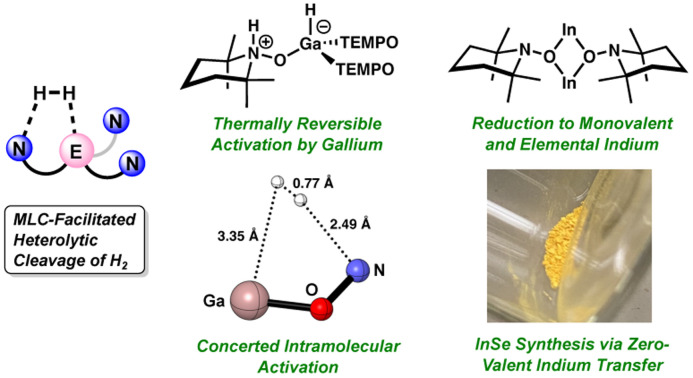

## Introduction

Metal–ligand cooperativity (MLC), which describes the synergistic interaction of metal and ligand with substrate in bond forming or breaking events, is a well-established tool in transition metal-mediated bond activation and catalysis^1^ and commonly found in nature.^2^ While this concept may be applied generally and used to impart redox innocent metals with ambiphilic properties, the field of MLC based on the p-block elements is considerably less developed.^3^ This approach complements existing major strategies in the burgeoning field of bond activation and catalysis by the p-block elements^4,5^ which rely on steric^6^ or geometric^7,8^ frustration or the generation of low-valent element sites;^9,10^ though we should note that parallels between MLC and frustrated Lewis pair (FLP) chemistry have been previously identified and the line distinguishing the two fields is often blurred.^11^

With the aim of imparting ambiphilicity on complexes of redox innocent trivalent group 13 metals, we have identified TEMPO (2,2,6,6-tetramethylpiperidine 1-oxyl) as a suitable ligand that may facilitate the heterolytic cleavage of H_2_ through MLC. In fact, tripodal tris(aminoxy)-supported group 13 complexes were recently shown to reversibly react with O–H bonds through a MLC pathway.^12^ Herein, we describe our initial investigation that revealed examples of remarkably facile dihydrogen activation. For indium, this process led to zero-valent metal generation, and when repeated in the presence of a selenium source, binary InSe was obtained, consistent with TEMPO functioning as a facilitative and removable ligand following H_2_ activation. Canonically, MLC has been leveraged for the activation of small molecules and in select cases catalysis, but to our knowledge the engineering of MLC to facilitate the preparation of materials is rare.

## Results and discussion

**Fig. 1:**
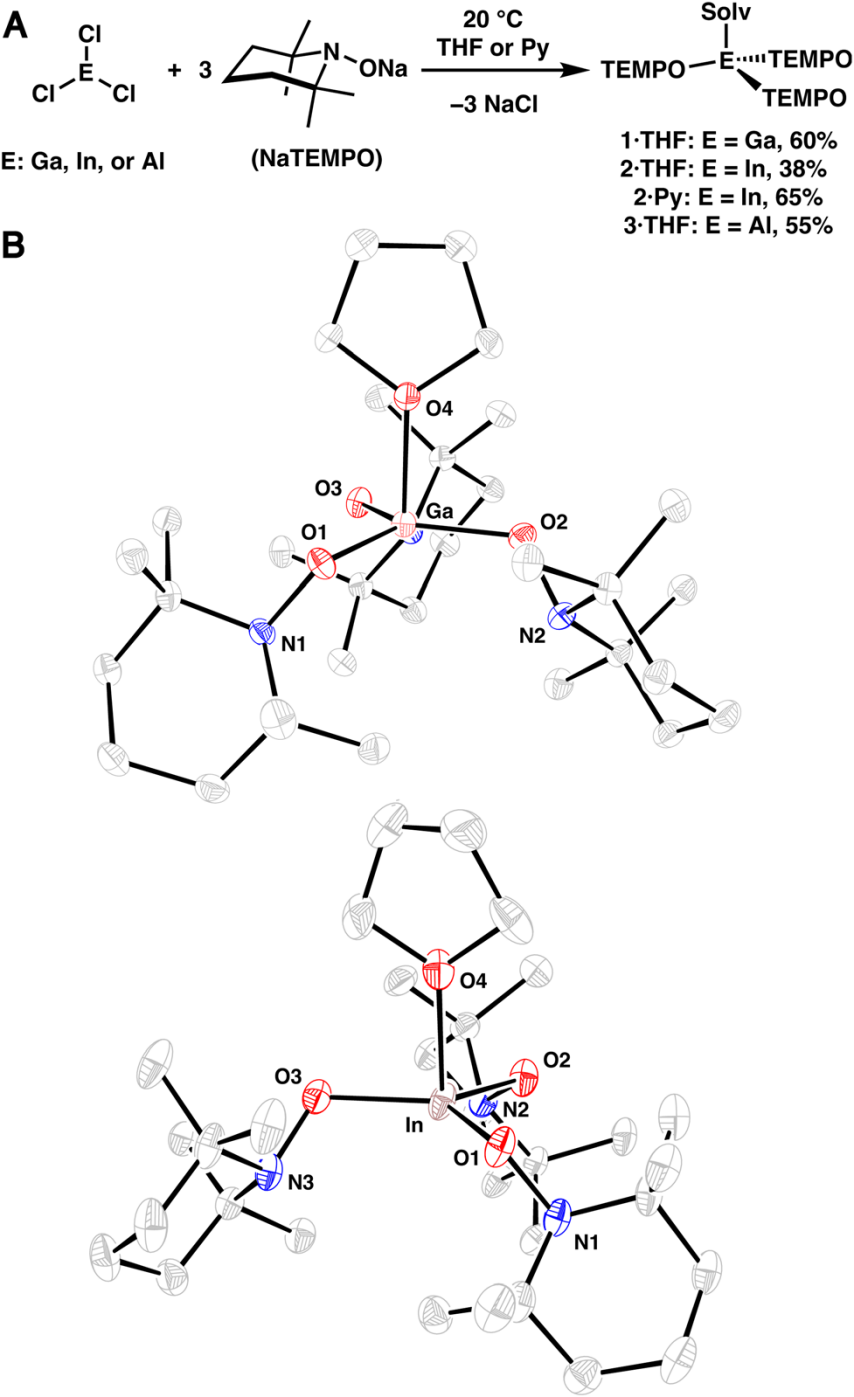
(A) Synthesis of aminoxy complexes 1·THF, 2·THF, 2·Py, and 3·THF. (B) SC-XRD structure of 1·THF (top) and 2·THF (bottom) with thermal ellipsoids shown at the 50% probability level. Hydrogen atoms are omitted for clarity.

Group 13-aminoxy complexes, in analogy to homoleptic first row TM complexes,^13^ were prepared *via* salt metathesis reactions of ECl_3_ (E = Ga, In, Al) and NaTEMPO (3 equiv.) in tetrahydrofuran (THF), leading to 1·THF, 2·THF, and 3·THF (Fig. 1A). The complexes were isolated in 60%, 38%, and 55% yield (1·THF, 2·THF, and 3·THF, respectively) following crystallization. Single crystal X-ray diffraction (SC-XRD) experiments revealed nearly trigonal monopyramidal geometries with THF occupying the apical position of the complexes, consistent with the significant steric bulk of the aminoxy ligands (Fig. 1B; see S.2†). The SC-XRD experiments also revealed average (avg.) N–O bond lengths (Table 1) that are similar to those of related dialkylaminoxy group 13 complexes and relatively short M–O(TEMPO) bond lengths.^14–19^ Reflecting the sterically congested geometries, longer than expected metal-THF bond lengths were observed (In: 2.251(10) Å, Ga: 2.0518(11) Å, Al: 1.9130(17) Å; Table 1).^20,21^ Dissolution of the complexes in pyridine-*d*_5_ results in the corresponding pyridine adducts and free THF, suggesting rapid ligand exchange at room temperature and is consistent with the greater basicity of pyridine. Heating the complexes in a poorly coordinating solvent (benzene-*d*_6_, 80 °C) leads to complex mixtures within 1 h (see S.1.6†), indicating that coordination of solvent is crucial to the stability of the complexes. Complex 2·THF exhibits particularly poor stability in non-donor solvents; therefore, the corresponding pyridine adduct 2·Py was prepared and isolated independently in an improved yield of 65% (Fig. 1A).

**Table 1:** Table of selected bond lengths (Å) determined *via* SC-XRD experiments (average, avg.)

Complex	M–THF	M–O(TEMPO) (avg.)	N–O (avg.)
3·THF	1.9130(17)	1.74	1.45
1·THF	2.0518(11)	1.82	1.46
2·THF	2.251(10)	2.01	1.46

**Fig. 2:**
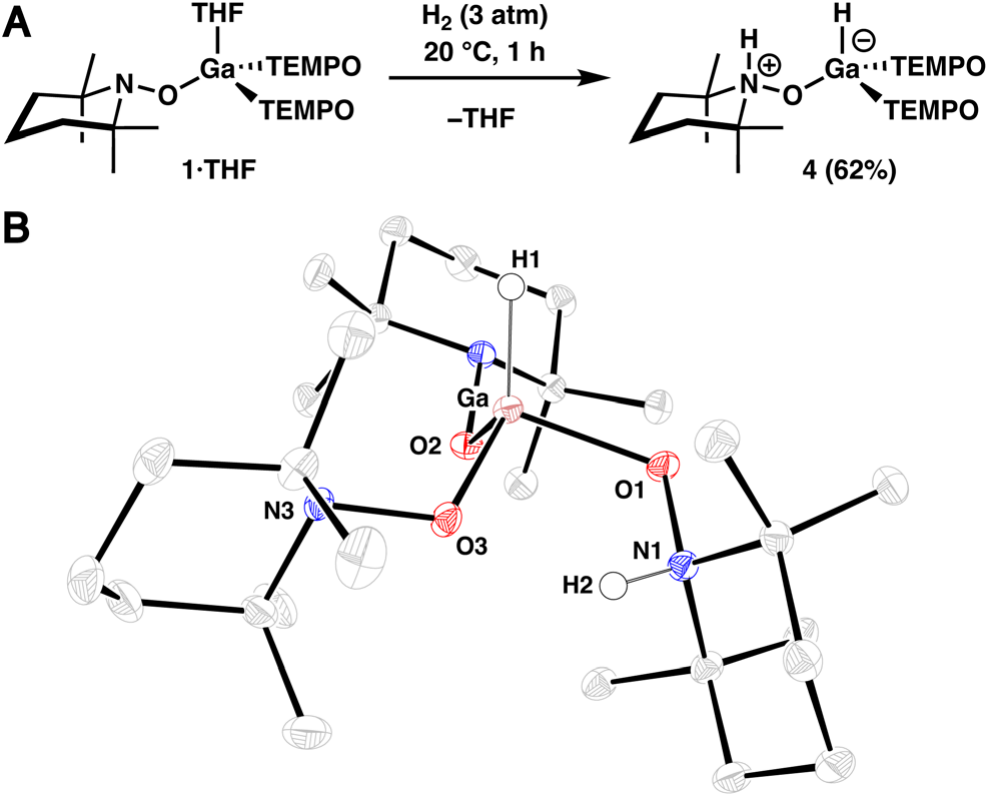
(A) Synthesis of dihydrogen adduct 4. (B) SC-XRD structure of 4 with thermal ellipsoids shown at the 50% probability level. Selected hydrogen atoms are omitted for clarity.

Recognizing the potentially ambiphilic nature of the complexes described above, where the metal atom and TEMPO ligand function as a Lewis acid and base, respectively, reactions with dihydrogen were investigated. Treatment of 1·THF in *n*-hexane (0.02 M) with H_2_ (3 atm) results in complete conversion to 4 at 20 °C within 1 h, which was isolated *via* crystallization in 62% yield (Fig. 2A). Consistent with our assignment, ^1^H NMR spectroscopic analysis of the crude reaction mixture in benzene-*d*_6_ revealed a broad (*ω*_1/2_ = 45 Hz) singlet at 5.72 ppm, diagnostic of a terminal Ga–H resonance,^22^ and a deshielded resonance at 8.88 ppm, which corresponds to Ga-bound N-protonated TEMPO ligand.^23^ Additionally, infrared (IR) spectroscopy of this mixture revealed bands at 1930 and 3570 cm^−1^, which correspond to the Ga–H and N–H stretches, respectively.^22,23^ Furthermore, the structure of 4 was confirmed in a SC-XRD experiment (Fig. 2B). The overall neutral charge of 4 indicates that H_2_ is split heterolytically, resulting in the formation of a protonated amine (H^+^) and a gallium hydride (H^−^). In contrast to the relatively small (THF)–Ga–O(TEMPO) average bond angle of 95.3° observed in 1·THF, complex 4 exhibits an average H–Ga–O(TEMPO) bond angle of 113.6°, consistent with a strong hydride adduct. Additionally, the Ga–O1 bond length in 4 is 1.9407(14) Å, which is relatively long when compared to that of Ga–O2 and Ga–O3 (1.8404(13) Å and 1.8634(13) Å, respectively), reflecting protonation of N1 in the complex. An intramolecular hydrogen-bonding interaction is also likely present in complex 4, with the O3–H2 interatomic distance measuring 2.038(25) Å.

To confirm the origin of the newly installed hydrogen atoms, a deuterium labeling experiment was performed using D_2_ in place of H_2_ which resulted in the disappearance of the N–H and Ga–H resonances and vibrational bands in the ^1^H NMR and IR spectra, respectively (see S.1.8.2†). While we were able to confirm isotopic substitution *via*^2^H NMR spectroscopy, we were unable to observe the Ga–D and N–D vibrational modes possibly due to overlapping solvent. The thermal stability of 4 in pyridine-*d*_5_ was also investigated (see S.1.9†), and we observed free H_2_ and the partial formation of 1·Py by ^1^H NMR spectroscopy after heating a solution of 4 to 80 °C for 20 h, indicating that 1 is able to reversibly bind H_2_.

For context, Aldridge reported H_2_ activation by an ambiphilic Ga^III^ complex, which was supported by an appreciably basic β-diketiminato (Nacnac)-derived dianionic diamide ligand framework, under albeit more forcing conditions (4 atm, 70 °C, 12 h).^24,25^ Goicoechea reported the facile activation of H_2_ by a phosphine-substituted phosphagallene,^26,27^ while Mitzel has developed an Al–O–P system,^28^ both of which similarly function as single-component FLPs. Additionally, the heterolytic cleavage of H_2_ under mild conditions was also observed for intermolecular FLPs based on P^*t*^Bu_3_ and potent Lewis acids E(C_6_F_5_)_3_ (E = Ga or In), leading to a E–H–E bridging hydride.^29^ Notably, none of the Ga-based systems above are reported to reversibly bind H_2_.

**Fig. 3:**
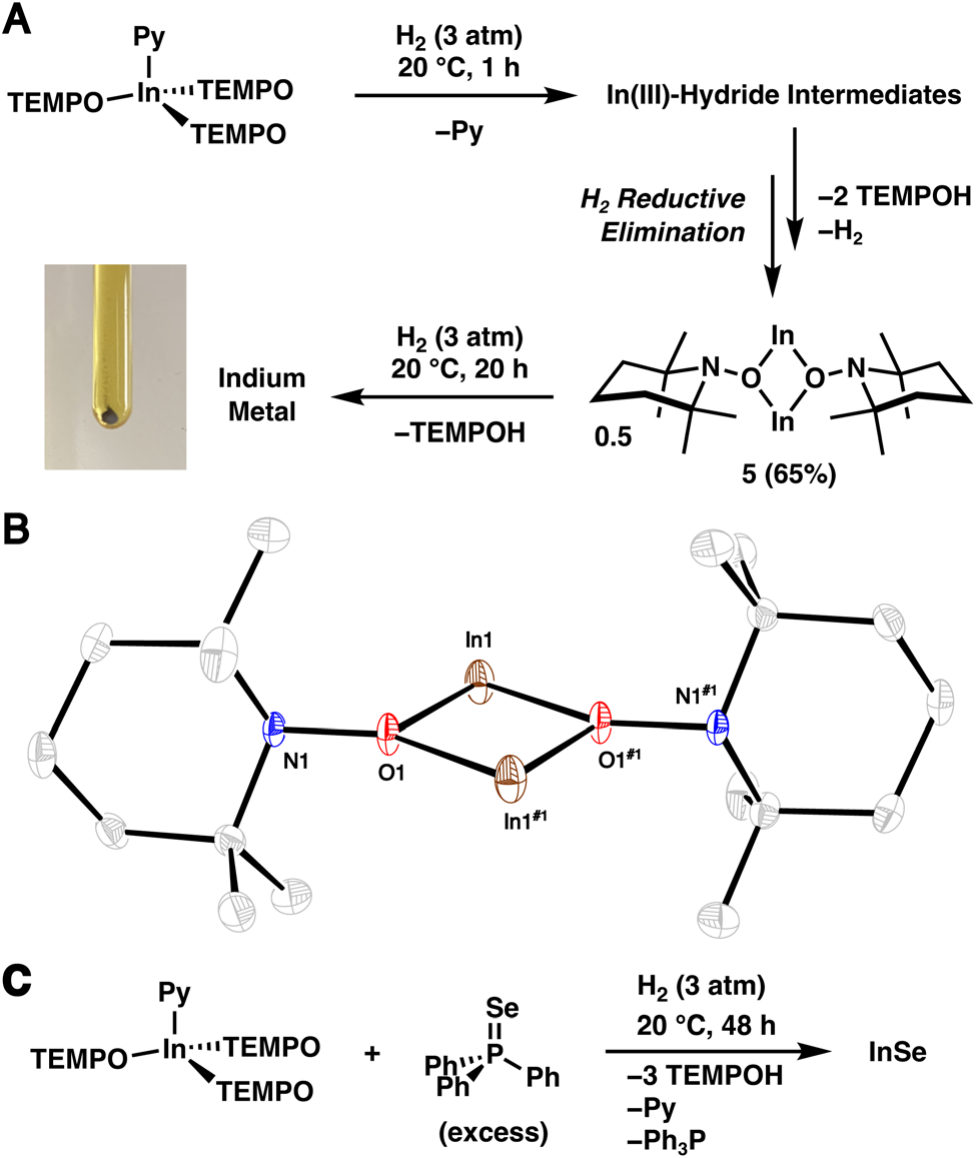
(A) Treatment of 2·Py with H_2_ leading to 5, *via* trivalent indium hydride intermediates, and its reduction to In metal (photograph of generated In metal shown to the bottom left). (B) SC-XRD structure of 5 with thermal ellipsoids shown at the 50% probability level. Hydrogen atoms are omitted for clarity. (C) Formation of InSe from 2·Py and Ph_3_PSe.

Similar to 1·THF, 2·Py readily activates H_2_. However, treatment of 2·Py in *n*-hexane (0.03 M) with H_2_ (3 atm) at 20 °C results in free TEMPOH and the In^I^ complex 5 in a 2 : 1 ratio within 1 h as assessed by NMR spectroscopy (Fig. 3A). The reaction of 2·Py with H_2_ appears to be tolerable of donor solvents and cleanly provides TEMPOH and 5 when combined in pyridine-*d*_5_ (see S.1.10.4†). Complex 1·THF, on the otherhand, reacts sluggishly with H_2_ in pyridine-*d*_5_ (see S.1.8.4†), possibly indicating the presence of a free coordination site plays a more important role in the latter complex. Complex 5 was isolated as a yellow, crystalline material in 65% yield and its identity was confirmed by SC-XRD (Fig. 3B). We propose that monovalent 5 forms *via* H_2_ reductive elimination following two sequential H_2_ activation reactions with 2 and subsequent dissociation of two TEMPOH ligands (see Section S.3.1.2† for a more detailed discussion on the potential mechanism). Note that reductive loss of H_2_ following H_2_ activation and subsequent dissociation of TEMPOH is expected given the high lability of In–H bonds in the absence of a stabilizing ligand environment.^30–32^ When the reaction was repeated with D_2_ in place of H_2_, a new band at 2634 cm^−1^ corresponding to the O–D stretch of free TEMPOD was observed (Fig. S.48†), matching previously reported data.^33^ To the best of our knowledge, the demonstration of H_2_ activation by an In complex under mild conditions is unprecedented.^29^

Complex 5 exhibits N1–O1–In1 and N1–O1–In1^#1^ angles of 107.34(14)° and 143.84(15)°, respectively, suggesting a weak interaction between N1 and In1. This interaction resembles the intramolecular nitrogen–silicon donor–acceptor interactions observed in aminoxy-substituted trifluoromethylsilanes F_3_SiONMe_2_ and (F_3_C)F_2_SiONMe_2_.^34,35^ Additionally, the interatomic distance of 2.9670(22) Å between In1 and N1 is shorter than the sum of the van der Waals radii (4.09 Å);^36^ though, we should note the limitations associated with identifying non-covalent interactions based on van der Waals radii.^37^

Extending the reaction time between 2·Py and H_2_ to 24 h leads to a gray-brown suspension. Remarkably, powder X-ray diffraction (PXRD) of the isolated solids revealed that they correspond to elemental In (Fig. S.54†),^38^ suggesting that 5 reacts further with H_2_ to form TEMPOH and In metal. To verify this transformation, a benzene-*d*_6_ solution of isolated 5 was treated with H_2_ (3 atm), and clean conversion to TEMPOH was observed within 20 h as assessed by ^1^H NMR spectroscopy (see S.1.11†). Gray solids were also generated and confirmed to correspond to In metal by PXRD. The complete reduction of 2 to elemental In is in line with its relatively low standard aqueous reduction potential (−0.34 V *vs.* SHE for In^III^ ⇌ In^0^).^39^ When the treatment of 2·Py with H_2_ was repeated with Ph_3_PSe (1.5 equiv.), orange solids corresponding to binary InSe^40^ precipitated from solution within minutes, revealing that 2·Py functions as a molecular synthon of elemental In when treated with H_2_ (Fig. 3C). Notably, in a separate experiment, no reaction is observed between In powder and Ph_3_PSe. Although PXRD analysis suggests the as prepared InSe is amorphous, inductively coupled plasma mass spectrometry (ICP-MS) indicates an approximate 1 : 1 In to Se stoichiometry. Consistent with aminoxy-assisted reduction, NMR analysis of the crude reaction mixture revealed TEMPOH and Ph_3_P. InSe is on the binary indium–selenium phase diagram^41^ and may be prepared from heating elemental indium and selenium to 900 °C^42^ or electrodeposition from solutions containing indium and selenite salts.^43^ The structure of the β form is known and contains an In–In bond, hence the divalent indium oxidation state.^40^ Note that our sample of InSe is amorphous likely due to the very low temperature of synthesis.

**Fig. 4:**
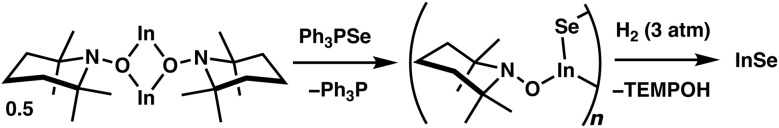
Generation of a proposed oligomeric intermediate following selenium transfer from Ph_3_PSe to 5 followed by the removal of the TEMPO ligands *via* the addition of H_2_.

To gain further insight into the discrete steps that may take place in this transformation, 5 was combined with Ph_3_PSe in the absence of H_2_ (Fig. 4; see S.1.18†). Within several minutes, the reaction mixture became intensely yellow and remained homogeneous, and ^31^P{^1^H} NMR analysis revealed the complete conversion of Ph_3_PSe to Ph_3_P. Notably, broad resonances were observed in the ^1^H NMR spectrum and may be attributable to an oligomeric TEMPO-substituted indium selenide intermediate (Fig. 4). Treatment of this crude reaction mixture with H_2_ (3 atm) led to the precipitation of an orange solid that is consistent with InSe within minutes. Additionally, clean formation of TEMPOH was also observed indicating that the intermediate generated upon Se transfer also participates in MLC-promoted H_2_ activation en route to the binary material.

Examples of molecular In complex reduction using H_2_ under mild conditions are exceedingly rare, highlighting the possible utility of ligand-assisted methods of depositing and transferring zero-valent metals. Moreover, the observed reduction of phosphorus in Ph_3_PSe opens the possibility of using alternative non-canonical chalcogenide sources for the preparation of materials. Our preparation brings to mind the synthesis of amorphous GaAs *via* the alcoholysis of molecular precursor (C_5_Me_5_)_2_Ga–As(SiMe_3_)_2_; although, we should note that the formal reduction of the metal atom is not observed during the course of this reaction and the significant driving force of Si–O bond formation.^44^

Activation of H_2_ by 3·THF was also investigated; however, no reaction with H_2_ (8 atm, 48 h) was observed after workup of the reaction mixture. We suspect that the greater electropositivity of Al leads to a more tightly bound metal–THF adduct and a weaker metal–hydride bond which result in thermodynamically unfavorable H_2_ activation. Consequently, the relative Lewis acidities of the aminoxy complexes were determined using the Gutmann–Beckett method with Et_3_PO as the Lewis base.^45^ The study suggests that 3, followed by 1 and then 2, is the most Lewis acidic, and the observed trend is supported by computed fluoride ion affinities (FIA, Table 2). Hydride ion affinities (HIA), which better represents soft interactions due to the greater polarizability of hydride, were also computed for the aminoxy complexes.^48^ This study revealed an opposing trend, where 2, followed by 1 and 3, exhibits the greatest HIA (Table 2). For comparison, Table 2 provides Gutmann–Beckett, FIA, and HIA values for pentafluorophenyl-substituted Lewis acids known to readily activate H_2_ when combined with an external Lewis base,^29,49,50^ highlighting the attenuated Lewis acidities of the aminoxy complexes presented in this work.

**Table 2:** Assessment of Lewis acidity using the Gutmann–Beckett method and computed FIA and HIA for 1, 2, 3, and selected Lewis acids (DSD-BLYP-D3BJ/def2-QZVPP//PBEh-3c/def2-mSVP). Δ*δ*_31P_ corresponds to the ^31^P{^1^H} NMR shift of free triethylphosphine oxide (TEPO) subtracted from that of the TEPO-Lewis acid adduct

Lewis acid	Δ*δ*_31P_^a^ (ppm)	Δ*δ*_31P_^b^ (ppm)	FIA^c^ (kJ mol^−1^)	HIA^d^ (kJ mol^−1^)
1, Ga(TEMPO)_3_	20.1	16.7	−353	−355
2, In(TEMPO)_3_	18.3	14.9	−341	−374
3, Al(TEMPO)_3_	21.1	17.4	−415	−327
B(C_6_F_5_)_3_	29.7 (ref. 46)	27.0 (ref. 47)	−442	−478
Al(C_6_F_5_)_3_		26.0	−549	−475
Ga(C_6_F_5_)_3_		23.1	−451	−467
In(C_6_F_5_)_3_		19.4	−420	−459

a In C_6_D_6_ at 25 °C.

b In CH_2_Cl_2_ at 25 °C.

c Fluoride ion affinity.

d Hydride ion affinity.

**Fig. 5:**
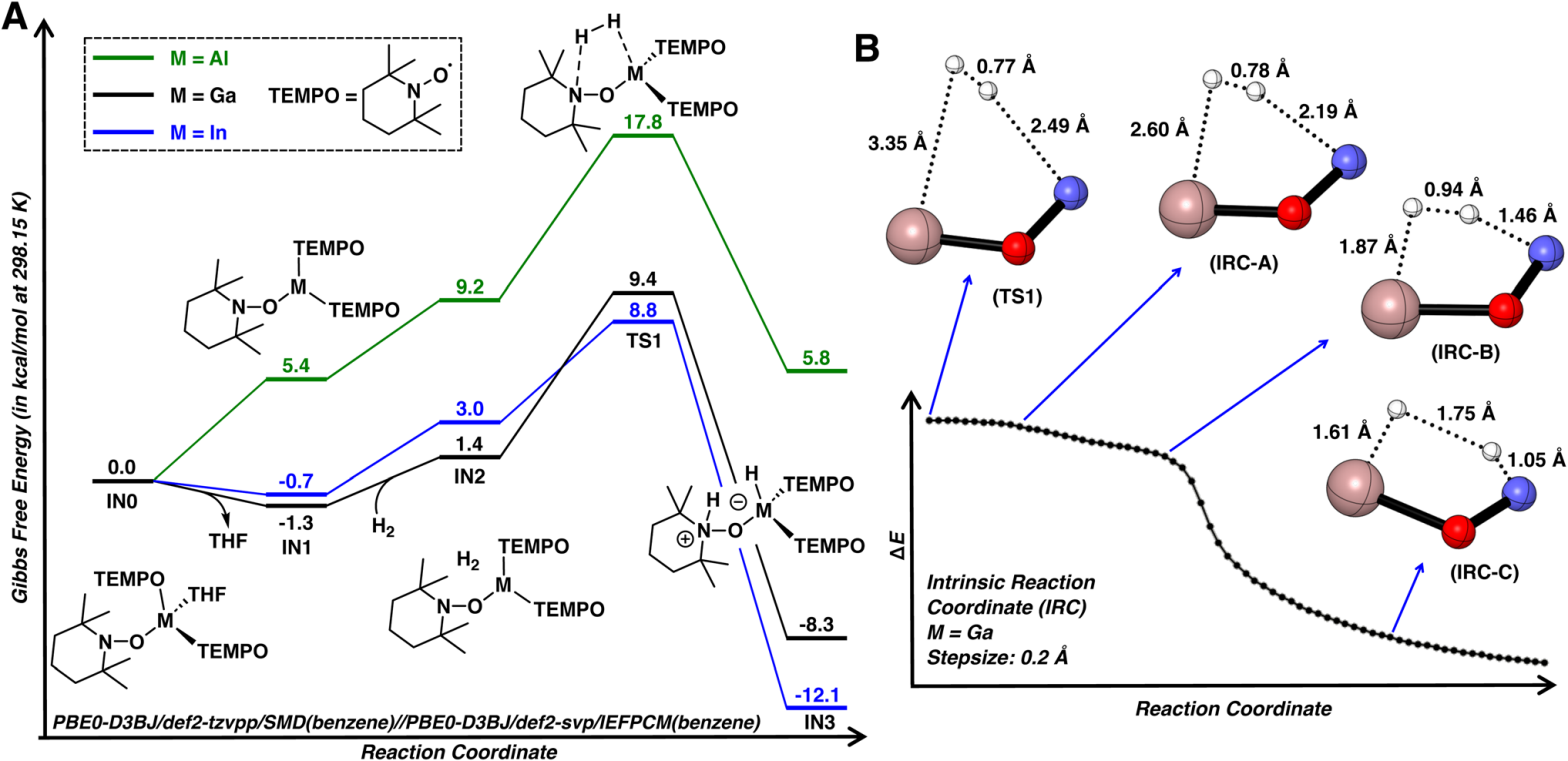
(A) Computed reaction pathway for H_2_ activation by complexes 1·THF, 2·THF, and 3·THF. (B) Forward intrinsic reaction coordinate for TS1 (M = Ga) and associated bond lengths of selected structures. Selected atoms were omitted for clarity. Color code: N, blue; O, red; Ga, salmon; H, white.

Density functional theory (DFT) calculations were performed to investigate the mechanism of dihydrogen activation using 1·THF as the model substrate (Fig. 5A). The reaction initiates by dissociation of THF, leading to three-coordinate *C*_3h_ symmetric IN1. While it's unlikely that dissociation of THF from 1·THF and 2·THF is exergonic, the calculations ultimately suggest that dissociation of THF is nearly thermoneutral. This is consistent with the poor stabilities of complexes 1·THF, 2·THF, and 3·THF in non-donor solvents and the rapid displacement of the THF ligand by pyridine, as described earlier. The subsequent formation of van der Waals complex IN2 is slightly endergonic by 2.7 kcal mol^−1^ due to the entropic cost and is followed by the cleavage of H–H bond *via*TS1 with a calculated activation barrier of 10.7 kcal mol^−1^. This cooperative interaction of a hydride and proton acceptor bears a qualitative resemblance to diphosphine molecular catalysts bearing pendant amines.^51^TS1 is followed by IN3 (4 when M = Ga), which is downhill by −8.3 kcal mol^−1^ as compared to 1·THF. The activation barrier for the reverse pathway was found to be 17.7 kcal mol^−1^, consistent with reversible dihydrogen activation observed for 1·THF when paired with strongly coordinating pyridine.

In TS1 (M = Ga; Fig. 5A), the H–H bond length is 0.77 Å, nearly identical to that of free dihydrogen (0.76 Å, calculated at the same level of theory) and the gallium atom retains its trigonal planar geometry. These observations indicate that TS1 is an early transition state, structurally and energetically similar to dihydrogen adduct IN2. To gain further insight into the dihydrogen activation process, the forward intrinsic reaction coordinate (IRC) was calculated from TS1 to IN3 (Fig. 5B). A shoulder area was first observed in the IRC curves, implying large molecular and electronic structural changes in the course of reaction. Further examination into the IRC reveals that the reaction is comprised of two stages. Initially, the dihydrogen molecule approaches the reaction site without significant structural changes at the gallium atom and H–H bond (IRC-A). Upon reaching the Ga and N atoms (IRC-B), the H–H bond is rapidly elongated, resulting a quick decrease in energy until the dihydrogen is completely cleaved to form IN3. The concerted nature of H–H bond cleavage is consistent with previous computational studies on intramolecular frustrated Lewis pairs.^52^

Similar to 1, complex 2 readily activates dihydrogen with a barrier of 9.5 kcal mol^−1^, leading to a metastable indium hydride (Fig. 5A; see S.3.1.2† for a more detailed discussion on the subsequent formation of 5). However, a significantly higher barrier of 17.8 kcal mol^−1^ was found for 3, consistent with the relatively lower computed HIA for 3. Although this barrier can be surmounted at room temperature, the resulting aluminum hydride is thermodynamically unfavorable by 5.8 kcal mol^−1^, which precludes its isolation. Note that group 13 complexes of the form [MH_2_(TEMPO)quin] (M = Al, Ga; quin = quinuclidine) or [MH(TEMPO)_2_quin] (M = Al) were previously prepared *via* the oxidation of the corresponding quinuclidine bound metal hydrides with TEMPO.^16^ The low temperature (*ca.* −50 °C) evolution of hydrogen at the onset of the reaction of quinuclidine bound metal hydrides with TEMPO suggests a thermodynamic driving force for the replacement of M–H bonds for M–TEMPO bonds in earlier group 13 metals.

## Conclusion

The present work describes the facile heterolytic cleavage of H_2_ by trivalent group 13 complexes with structurally affixed pendant amines, highlighting the utility of MLC in the context small molecule activation by complexes bearing a redox-innocent metal atom. This mode of activation is likely generalizable to other aminoxy–metal complexes and may have been inadvertently observed for Zn(TEMPO)_2_.^53^ Additionally, for trivalent indium, complete reduction to indium metal was observed and this pathway was leveraged in the synthesis of binary InSe. Notably, analogous chemical reactivity was not observed from bulk In metal, highlighting that low valent metal redox chemistry hitherto inaccessible may be exploited for the syntheses of materials through MLC. The established synthetic and reaction chemistry of aminoxyl radicals,^54–56^ coupled with the simple synthesis of aminoxy–element complexes, provides a ripe opportunity for developing highly tunable systems for bond activation reactions.

## Data availability

Crystallographic data are available from the Cambridge Structural Database under refcodes 2347342, 2347343, 2347344, 2347345, and 2347346. Full synthetic and computational details, including preparative procedures and spectroscopic data for characterization of compounds, are available in the ESI.†

## Author contributions

M.-L. Y. Riu performed all experiments and prepared the manuscript and ESI.† DFT calculations were conducted by J.-R. Shan, K. N. Houk, and M.-L. Y. Riu. M. Nava directed the project and revised the manuscript. All authors have given approval to the final version of the manuscript.

## Conflicts of interest

There are no conflicts to declare.

## Supplementary Material

SC-016-D4SC07938H-s001

SC-016-D4SC07938H-s002
