# Supplementary material for: Metal–ligand cooperativity enables zero-valent metal transfer
Source: Chem Sci. 2025 Jan 22;16(9):3888–94. doi: 10.1039/d4sc07938h (PMC11777705; doi:10.1039/d4sc07938h)
Supplement: SC-016-D4SC07938H-s001 [file SC-016-D4SC07938H-s001.pdf]

Supplementary Information (SI) for Chemical Science.
This journal is © The Royal Society of Chemistry 2025

Supplementary Materials for
Metal-Ligand Cooperativity Enables Zero-Valent Metal Transfer

Martin-Louis Y. Riu, Jing-Ran Shan, Prof. K. N. Houk*, Prof. Matthew Nava*

correspondence to: houk@chem.ucla.edu; mnava@chem.ucla.edu

Table of Contents

S.1 Synthetic Details **S.3**

S.1.1 General Information S.3

S.1.2 Synthesis of (THF)Ga(TEMPO)$_3$ (**1**·THF) S.4

S.1.3 Synthesis of (THF)In(TEMPO)$_3$ (**2**·THF) S.7

S.1.4 Synthesis of (Py)In(TEMPO)$_3$ (**2**·Py) S.10

S.1.5 Synthesis of (THF)Al(TEMPO)$_3$ (**3**·THF) S.12

S.1.6 Investigating the Thermal Stability of the Complexes **1**·THF, **2**·Py, and **3**·THF S.15

S.1.7 Gutmann-Beckett Studies S.19

S.1.8 Synthesis of (H_2)Ga(TEMPO)$_3$ (**4**) S.22

S.1.8.1 Vibrational Analysis of the Crude Reaction Mixture S.25

S.1.8.2 Treatment of **1**·THF with D_2 . . . . . S.26

S.1.8.3 Quantification of Conversion using an Internal Standard S.30

S.1.8.4 Treatment of **1**·THF with H_2 in pyridine-d_5 . . . . . S.34

S.1.9 Investigating the Thermal Stability of **4** S.34

S.1.10 Synthesis of {In(TEMPO)}$_2$ (**5**) S.38

S.1.10.1 Treatment of **2**·Py with D_2 . . . . . S.41

S.1.10.2 Quantification of Conversion using an Internal Standard S.44

S.1.10.3 Generation of Indium Metal from **2**·Py S.48

S.1.10.4 Treatment of **2**·Py with H_2 in pyridine-d_5 . . . . . S.50

S.1.11 Treatment of **5** with H_2 . . . . . S.52

S.1.12 Investigating the Thermal Stability of **5** S.54

S.1.13 Treatment of InCl with NaTEMPO S.56

S.1.14 Synthesis of InSe via the Treatment of **2**·Py with Ph_3PSe and H_2 . . . . . S.57

S.1.14.1 Elemental Analysis of InSe S.57

S.1.14.2 ICP-MS Analysis of InSe S.57

S.1.14.3 PXRD Analysis of InSe S.58

S.1.14.4 Diffuse-Reflectance UV-Vis Data of InSe S.59

S.1.15 SEM-EDX Analysis of InSe S.61

S.1.16 Monitoring the Treatment of **2**·Py with Ph_3PSe and H_2 . . . . S.63

S.1.17 Treatment of In Powder with Ph_3PSe S.65

S.1.18 Treatment of $\{In(TEMPO)\}_2$ with Ph_3PSe, followed by H_2 . . . . S.66

S.1.19 Variable Temperature ^1H NMR Study on the Treatment of **3**·THF with H_2 S.69

S.2 X-Ray Diffraction Studies **S.71**

S.3 Computational Studies **S.82**

S.3.1 Reaction Pathway Calculations S.82

S.3.1.1 General Information S.82

S.3.1.2 H_2 Activation S.83

S.3.1.3 Benzene C−H Activation by **1**·THF S.85

S.3.1.4 Cartesian Coordinates of DFT Optimized Structures Involved in H_2 Activation S.86

S.3.1.5 Cartesian Coordinates of DFT Optimized Structures Involved in Benzene C−H Activation S.150

S.3.2 Calculated Fluoride and Hydride Ion Affinities S.163

S.3.2.1 Cartesian Coordinates of DFT Optimized Structures S.165

References **S.200**

S.1 Synthetic Details

S.1.1 General Information

Except as otherwise noted, all manipulations were performed in a Vigor glovebox under an inert atmosphere of purified N_2 (O_2 <2.0 ppm). All solvents were obtained oxygen-free by bubble degassing (Ar) or by being placed under dynamic vacuum for >15 min with stirring. Tetrahydrofuran (THF) and diethyl ether were also purified by passage through columns of alumina using a solvent purification system (Pure Process Technology, Nashua, NH).[1] Trace water was removed from the solvents by storage over 4 Å molecular sieves.[2] Deuterated solvents were degassed by three freeze-pump-thaw cycles and stored over 4 Å molecular sieves for at least 48 h prior to use. Celite and 4 Å molecular sieves were dried by heating above 200 °C under dynamic vacuum for at least 24 h prior to use. All glassware, stirbars, and glass microfiber filter paper were dried in an oven for at least two hours at temperatures greater than 150 °C. NaTEMPO was prepared by stirring 2,2,6,6-tetramethyl-1-piperidinyloxy (TEMPO) with an equimolar amount of sodium metal for 48 h and isolated by vacuum filtration.[3] Ultra high purity hydrogen gas (impurities <0.1 ppm) was purchased from Airgas and used as received. All other reagents were purchased from commercial suppliers and used as received.

Nuclear magnetic resonance (NMR) spectra were collected on a Bruker Avance 400 MHz, Bruker Avance 500 MHz (equipped with a nitrogen-cooled cryoprobe), and a Bruker Avance NEO 600 MHz (equipped with a nitrogen-cooled cryoprobe) spectrometer. ^1H, ^2H, and ^{13}C NMR spectra were referenced internally to solvent signals.[4] ^{31}P NMR spectra were externally referenced to 85% H_3PO_4 (0 ppm). Infrared (IR) spectroscopy data were collected using a Cary 600 Series FTIR Spectrometer. Powder X-Ray Diffraction (PXRD) data were collected using a Panalytical X'Pert Pro X-ray Powder Diffractometer. Elemental combustion analyses were performed by Midwest Micro Laboratories (Indianapolis, IN, USA). UV-vis data were collected using Cary 5000 UV-Vis-NIR Spectrophotometer.

S.1.2 Synthesis of (THF)Ga(TEMPO)$_3$ (**1**·THF)

To a stirring solution of $GaCl_3$ (0.400 g, 2.27 mmol, 1.00 equiv) in THF (20 mL) was added a solution of NaTEMPO (1.22 g, 6.81 mmol, 3.00 equiv) in THF (40 mL) dropwise over 20 min. After the solution stirred for *ca.* 16 h, all volatile materials were removed under reduced pressure, resulting in pale pink solids. The solids were suspended in *n*-hexane (40 mL) and the suspension was passed through a 30 mL fine sintered frit containing a half-inch plug of Celite. The Celite plug was washed with additional *n*-hexane (2 × 15 mL). The combined filtrates were concentrated to ca. 60 mL under reduced pressure and placed in a −35 °C freezer for crystallization. This provided **1**·THF as a colorless crystalline material (0.825 g, 1.35 mmol, 60% yield). NMR analysis of **1**·THF in pyridine-d_5 reveals the presence of free THF (Fig. S.1 and S.2; see section S.1.6 for ^{1}H NMR data in benzene-d_6), consistent with the displacement of coordinated THF with pyridine-d_5. ^{1}H NMR (600 MHz, pyridine-d_5, 25 °C, Fig. S.1) δ 1.54, 1.47, 1.32 ppm. $^{13}C\{^1H\}$ NMR (101 MHz, pyridine-d_5, 25 °C, Fig. S.2) δ 60.24, 41.33, 34.03 (br), 20.26 (br), 18.32 ppm. Elem. Anal. Calc'd(found) for $C_{31}H_{62}N_3O_4Ga$: C 60.98(61.08), H 10.24(10.28), N 6.88(6.97).

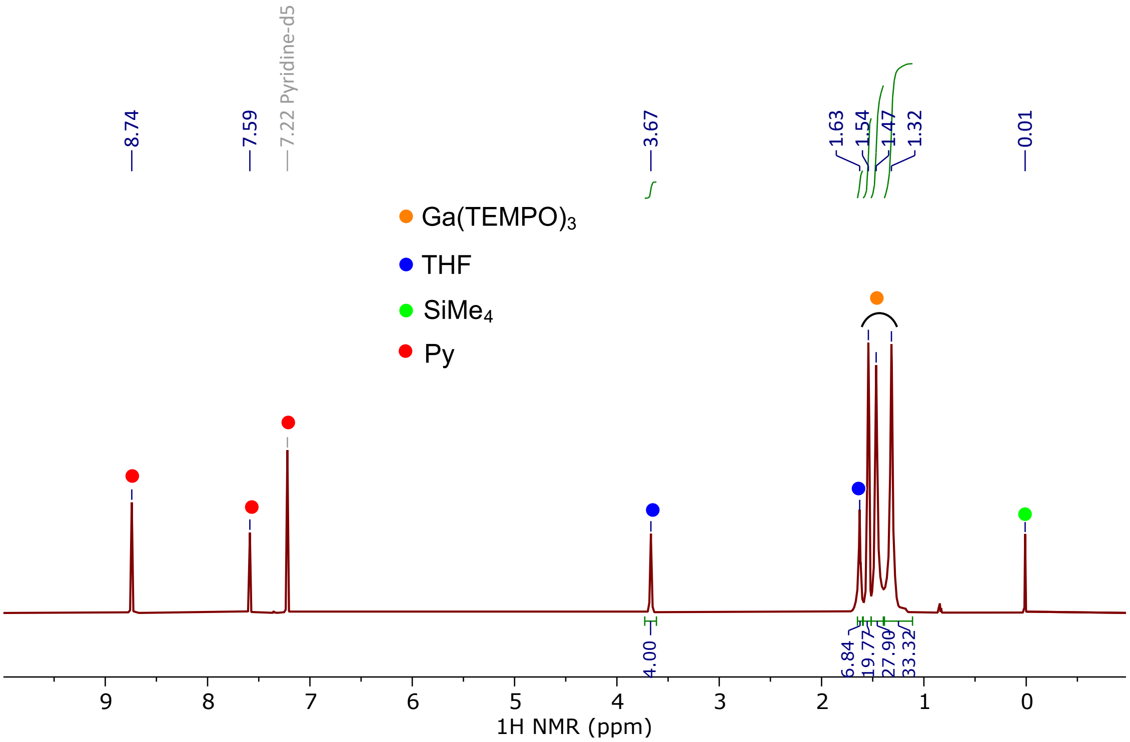

Figure S.1: ^{1}H NMR (600 MHz, pyridine-d_5, 25 °C) spectrum of **1**·THF.

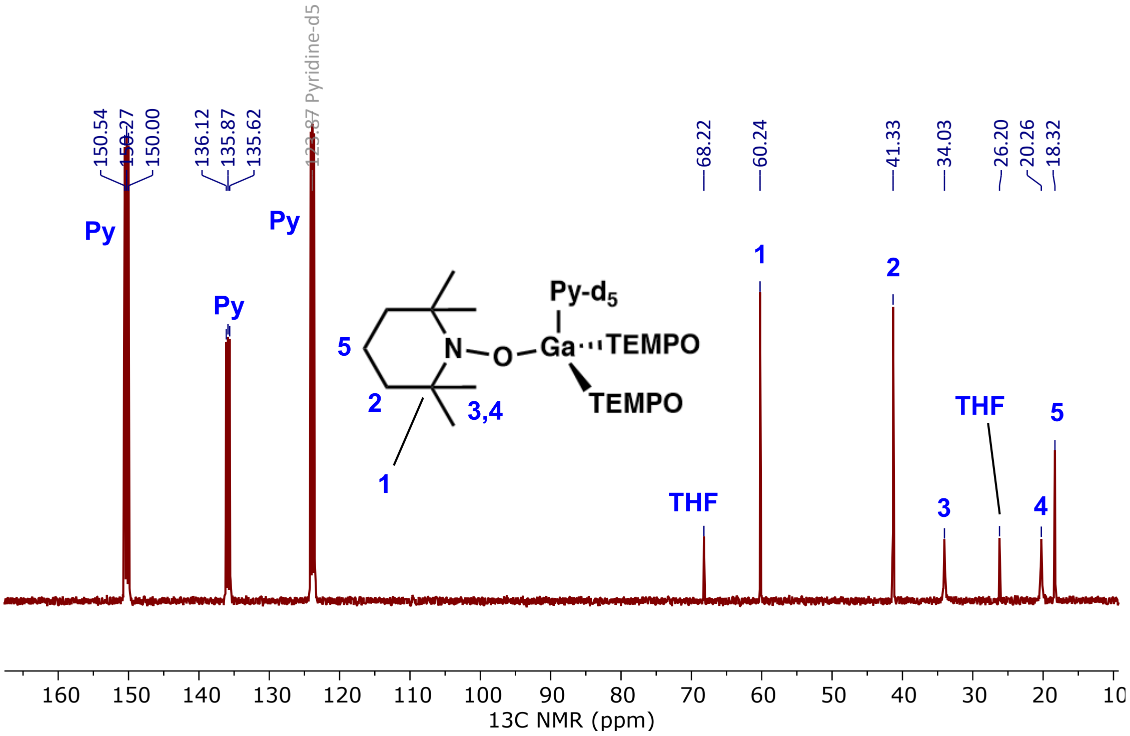

Figure S.2: ^{13}C{^{1}H} NMR (101 MHz, pyridine-d_5, 25 °C) spectrum of **1**·THF.

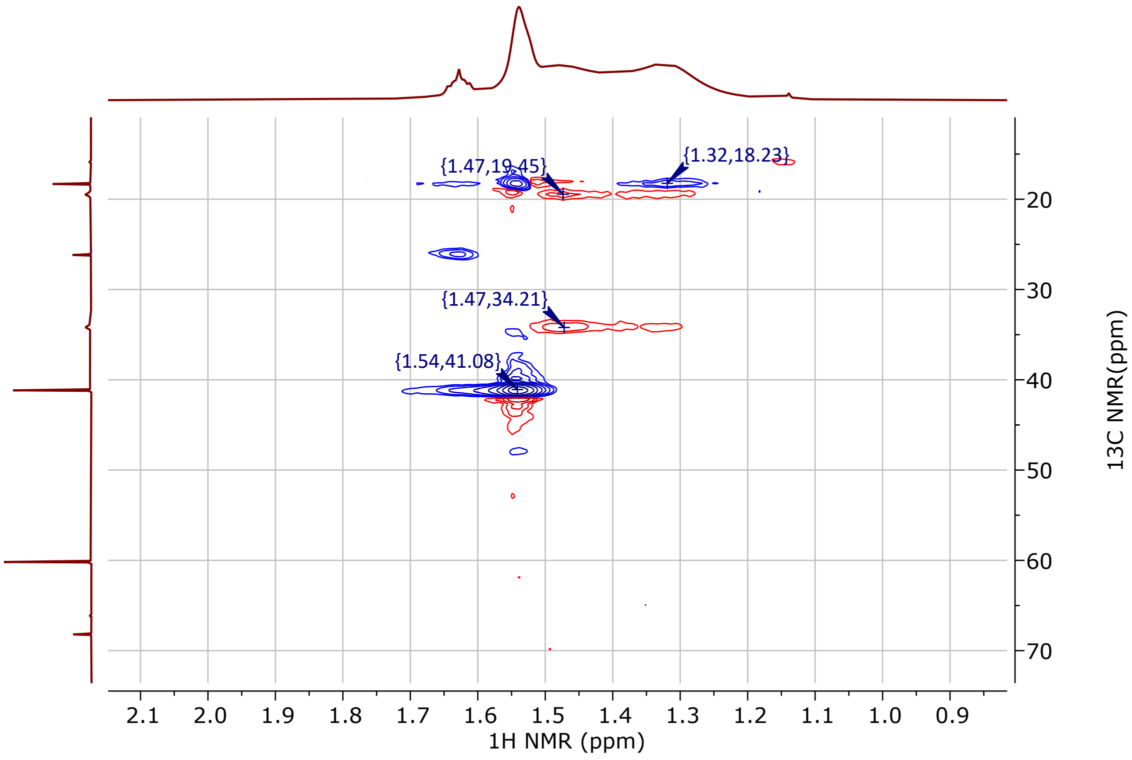

Figure S.3: Multiplicity-edited ^{1}H, ^{13}C-HSQC NMR (400 MHz, pyridine-d_5, 25 °C) spectrum of **1**·THF.

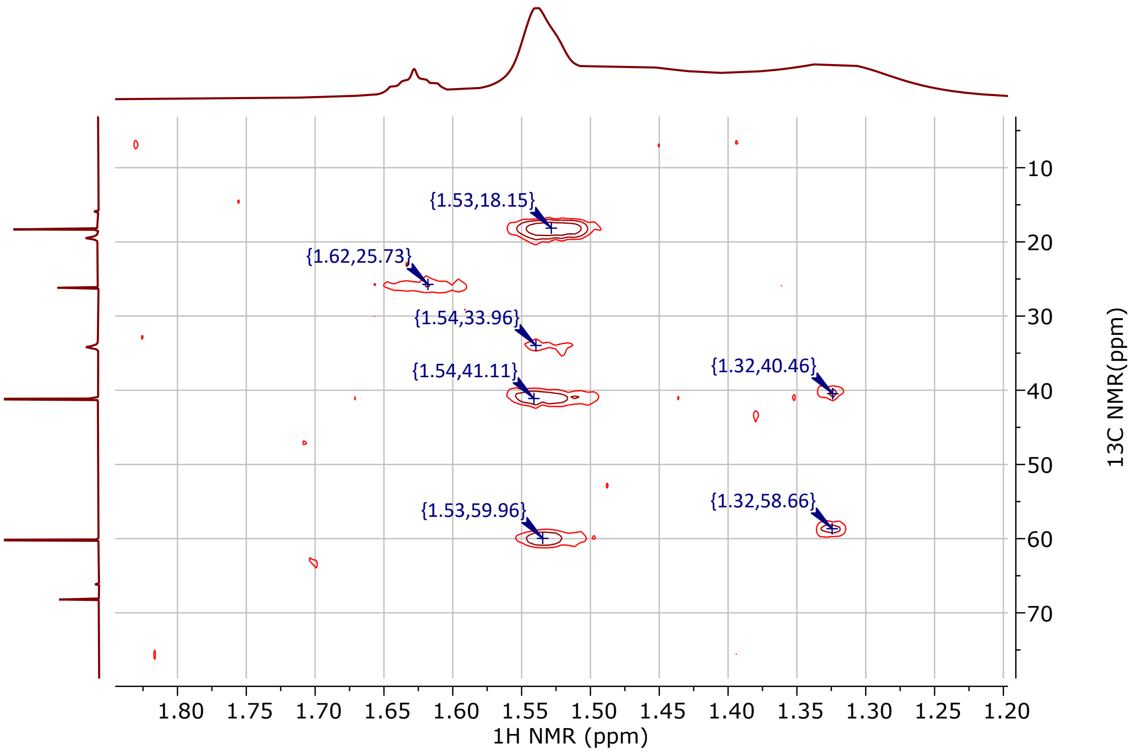

Figure S.4: ^{1}H, ^{13}C-HMBC NMR (400 MHz, pyridine-d_5, 25 °C) spectrum of **1**·THF.

S.1.3 Synthesis of (THF)In(TEMPO)$_3$ (2·THF)

To a stirring solution of $InCl_3$ (0.221 g, 1.00 mmol, 1.00 equiv) in THF (15 mL) was added a solution of NaTEMPO (0.538 g, 3.00 mmol, 3.00 equiv) in THF (15 mL) dropwise over 20 min. After the pale yellow, cloudy suspension stirred for 1 h, all volatile materials were removed under reduced pressure. The resulting pale yellow solids were suspended in diethyl ether (30 mL) and filtered through a 15 mL medium sintered frit containing a half inch plug of Celite. The Celite was washed with diethyl ether (10 mL) and the combined filtrates were concentrated to ca. 5 mL. The cloudy solution was filtered through a pipette containing a plug of glass microfiber filter paper and the filtrate was placed in a −35 °C freezer for crystallization. **2**·THF was isolated as a pale yellow, crystalline material (0.251 g, 0.383 mmol, 38% yield). NMR analysis of **2**·THF in pyridine-d_5 reveals the presence of free THF (Fig. S.5 and S.6; see section S.1.6 for ^{1}H NMR data in benzene-d_6), consistent with the displacement of coordinated THF with pyridine-d_5. ^{1}H NMR (600 MHz, pyridine-d_5, 25 °C, Fig. S.5) δ 1.54, 1.50, 1.31 ppm. $^{13}C\{^1H\}$ NMR (101 MHz, pyridine-d_5, 25 °C, Fig. S.6) δ 60.18, 41.16, 34.18 (br), 19.50 (br), 18.32 ppm. Compound **2**·THF was recrystallized from Et_2O for elemental analysis. Elem. Anal. Calc'd(found) for $C_{31}H_{62}N_3O_4In$: C 56.79(55.53), H 9.53(9.44), N 6.41(6.37).

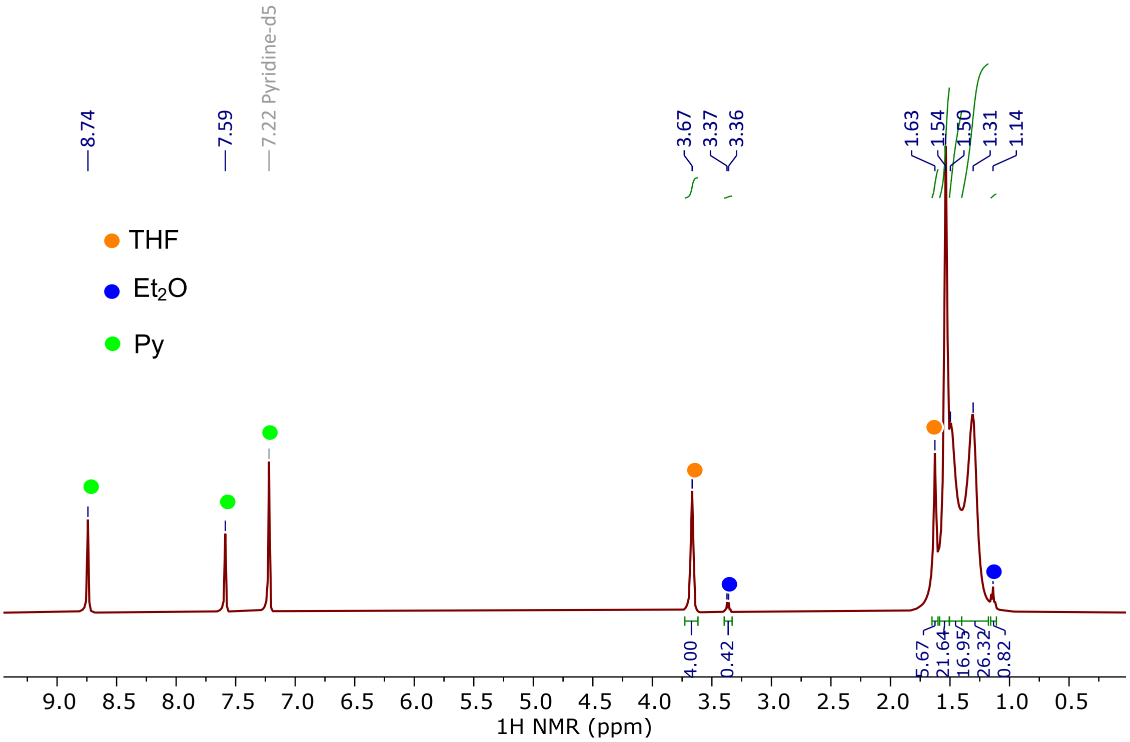

Figure S.5: ^{1}H NMR (500 MHz, pyridine-d_5, 25 °C) spectrum of **2**·THF.

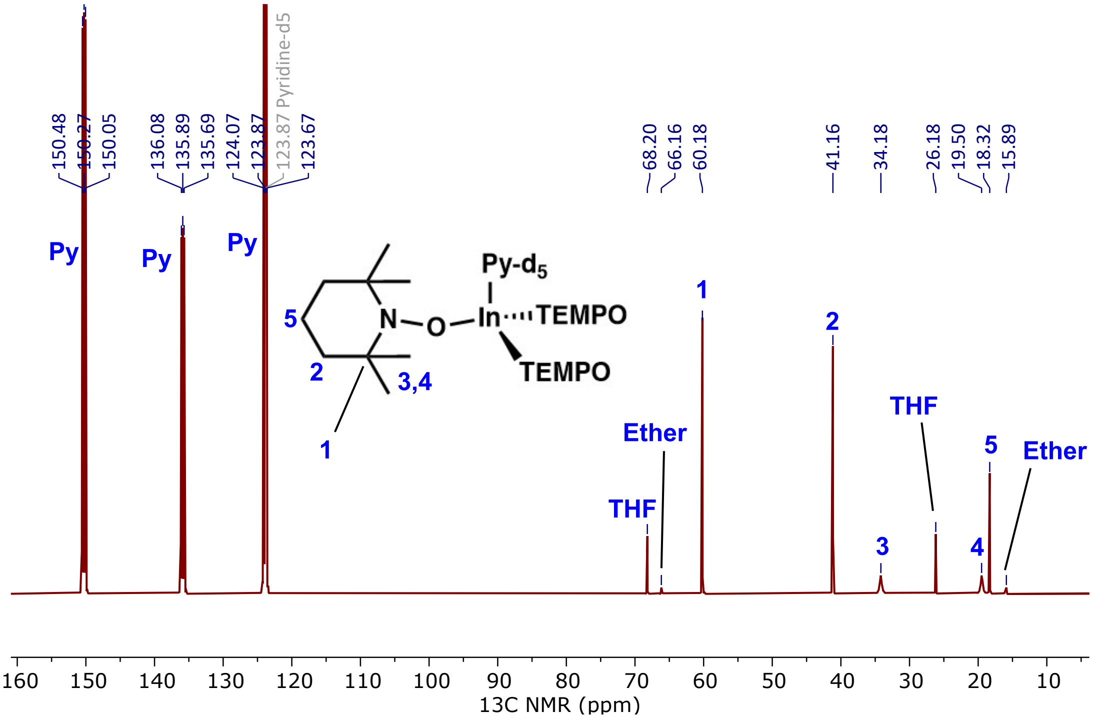

Figure S.6: ^{13}C{^{1}H} NMR (126 MHz, pyridine-d_5, 25 °C) spectrum of **2**·THF.

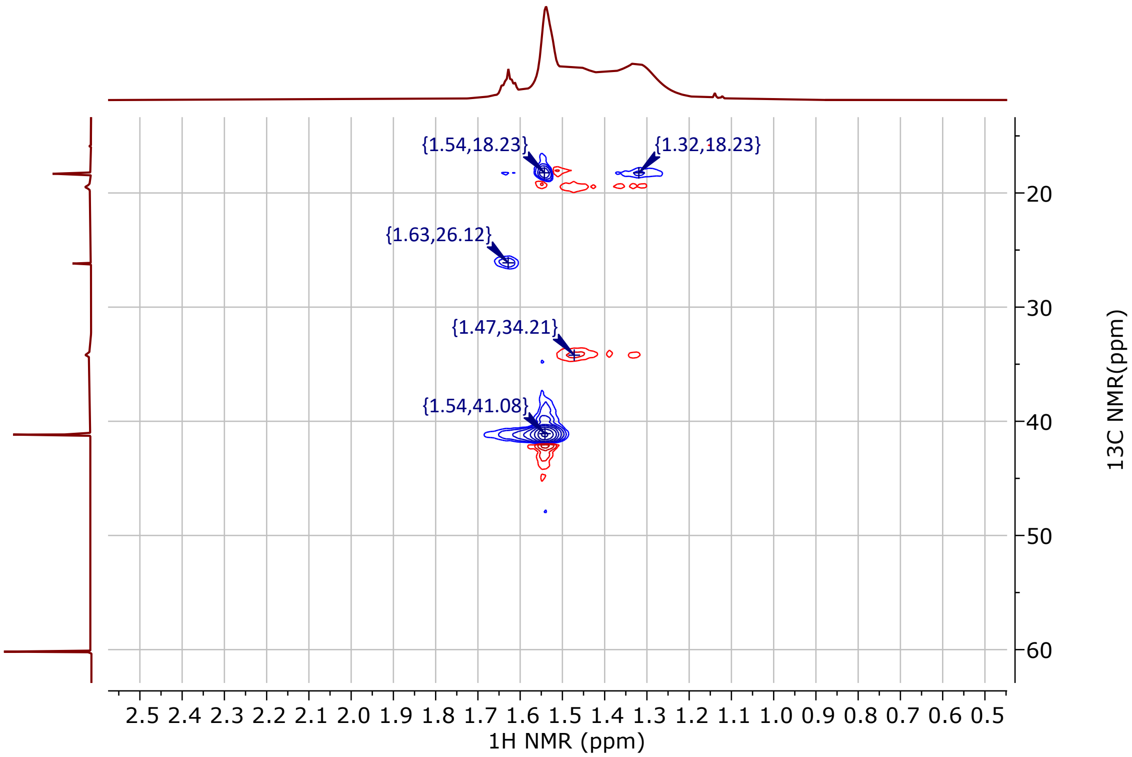

Figure S.7: Multiplicity-edited ^{1}H, ^{13}C-HSQC NMR (400 MHz, pyridine-d_5, 25 °C) spectrum of **2**·THF.

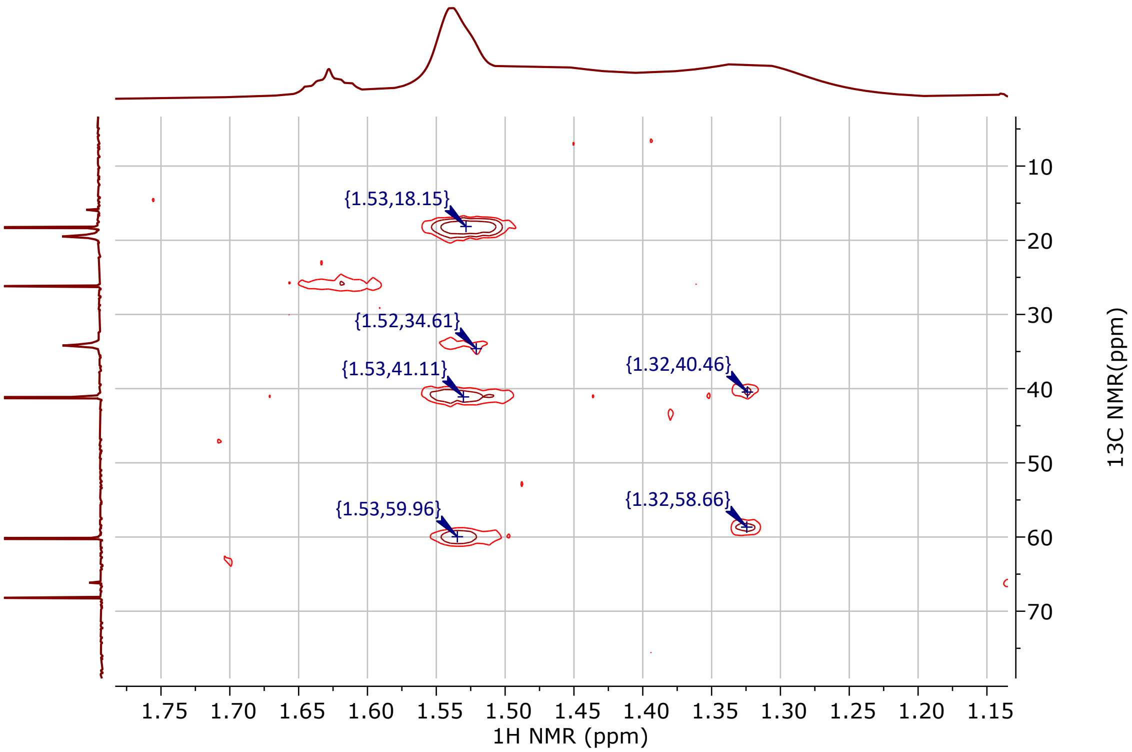

Figure S.8: ^{1}H, ^{13}C-HMBC NMR (400 MHz, pyridine-d_5, 25 °C) spectrum of **2**·THF.

S.1.4 Synthesis of (Py)In(TEMPO)$_3$ (2·Py)

To a stirring solution of $InCl_3$ (0.411 g, 1.86 mmol, 1.00 equiv) in pyridine (10 mL) was added a solution of NaTEMPO (1.00 g, 5.58 mmol, 3.00 equiv) in pyridine (20 mL) dropwise over 20 min. After the solution stirred for 1 h, all volatile materials were removed from the yellow solution under reduced pressure. The resulting yellow solids were suspended in *n*-hexane (40 mL) and passed through a 30 mL fine sintered frit containing a half-inch plug of Celite. The Celite was washed with *n*-hexane (2 × 10 mL) and the combined filtrates was placed in a −35 °C freezer for crystallization. **2**·Py was isolated as a pale yellow, crystalline material (0.802 g, 1.21 mmol, 65% yield). The NMR data matches those of **2**·THF (Fig. S.5 and S.6). Elem. Anal. Calc'd(found) for $C_{32}H_{59}N_4O_3In$: C 58.00(58.14), H 8.97(9.07), N 8.45(8.54).

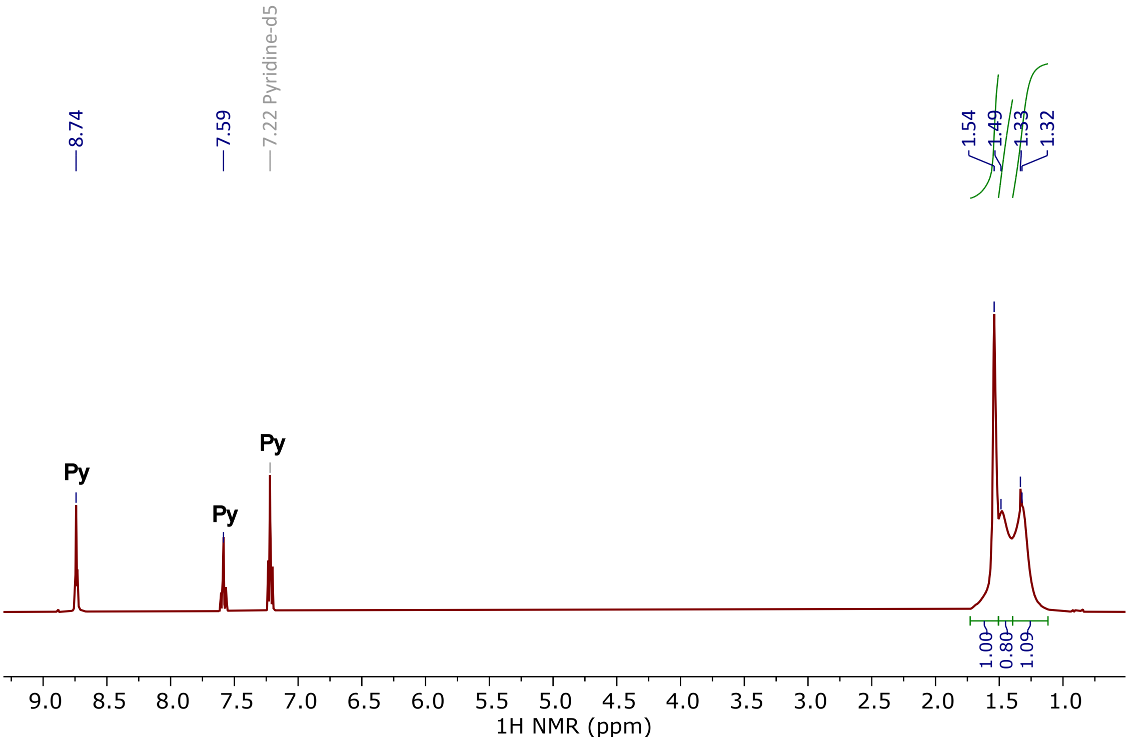

Figure S.9: ^{1}H NMR (400 MHz, pyridine-d_5, 25 °C) spectrum of **2**·Py.

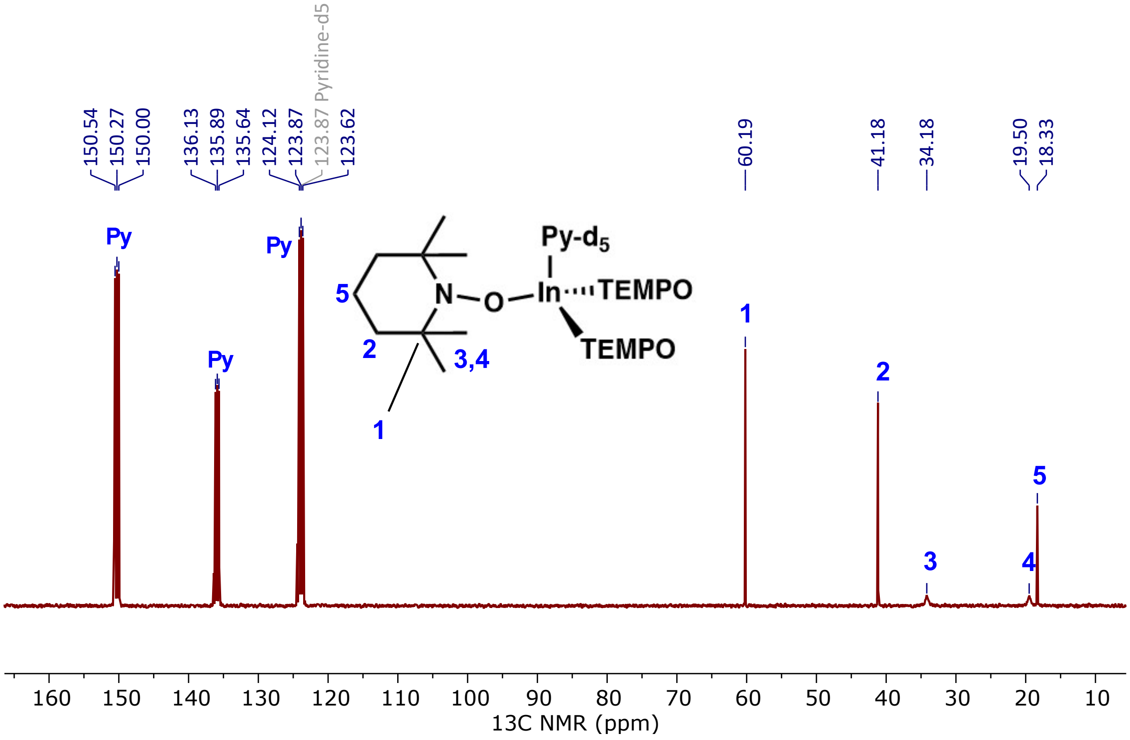

Figure S.10: $^{13}C\{^{1}H\}$ NMR (101 MHz, pyridine-d_5, 25 °C) spectrum of **2**·THF.

S.1.5 Synthesis of (THF)Al(TEMPO)$_3$ (3·THF)

To a stirring solution of $AlCl_3$ (0.320 g, 2.40 mmol, 1.00 equiv) in THF (20 mL) was added a solution of NaTEMPO (1.29 g, 7.20 mmol, 3.00 equiv) in THF (30 mL) dropwise over 20 min. After the solution stirred for *ca.* 16 h, all volatile materials were removed from the suspension under reduced pressure. The colorless solids were suspended in *n*-hexane (20 mL) and filtered through a 15 mL medium sintered frit containing a half-inch plug of Celite. The Celite was washed with *n*-hexane (10 mL) and the combined filtrates was concentrated to *ca.* 6 mL, which was placed in the −35 °C freezer for crystallization. **3**·THF was isolated as a colorless, crystalline material (0.753 g, 1.33 mmol, 55% yield). NMR analysis of **3**·THF in pyridine-d_5 reveals the presence of free THF (Fig. S.11 and S.12; see section S.1.6 for ^{1}H NMR data in benzene-d_6), consistent with the displacement of coordinated THF with pyridine-d_5. ^{1}H NMR (600 MHz, pyridine-d_5, 25 °C, Fig. S.11) δ 1.54, 1.35 ppm. ^{13}C{^{1}H} NMR (101 MHz, pyridine-d_5, 25 °C, Fig. S.12) δ 59.89, 41.15, 34.30 (br), 20.36 (br), 18.25 ppm. Compound **3**·THF was recrystallized from *n*-hexane for elemental analysis. Elem. Anal. Calc'd(found) for $C_{31}H_{62}N_3O_4Al$: C 65.57(64.43), H 11.01(10.66), N 7.40(7.37).

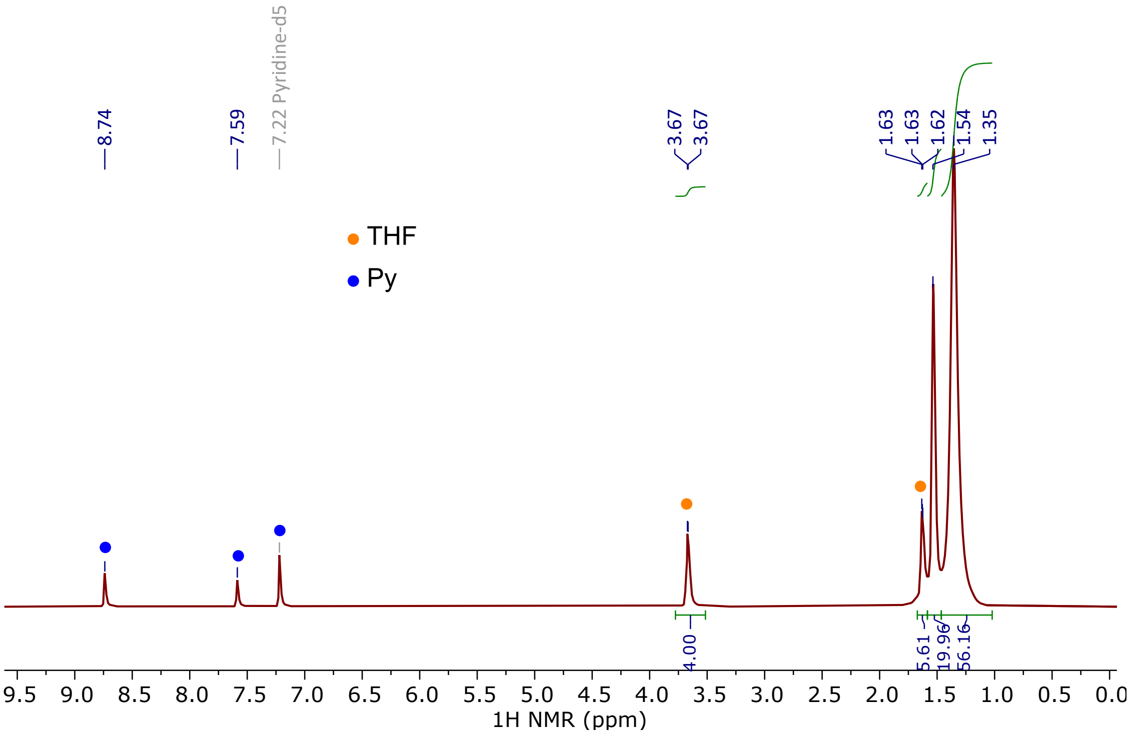

Figure S.11: ^{1}H NMR (400 MHz, pyridine-d_5, 25 °C) spectrum of **3**·THF.

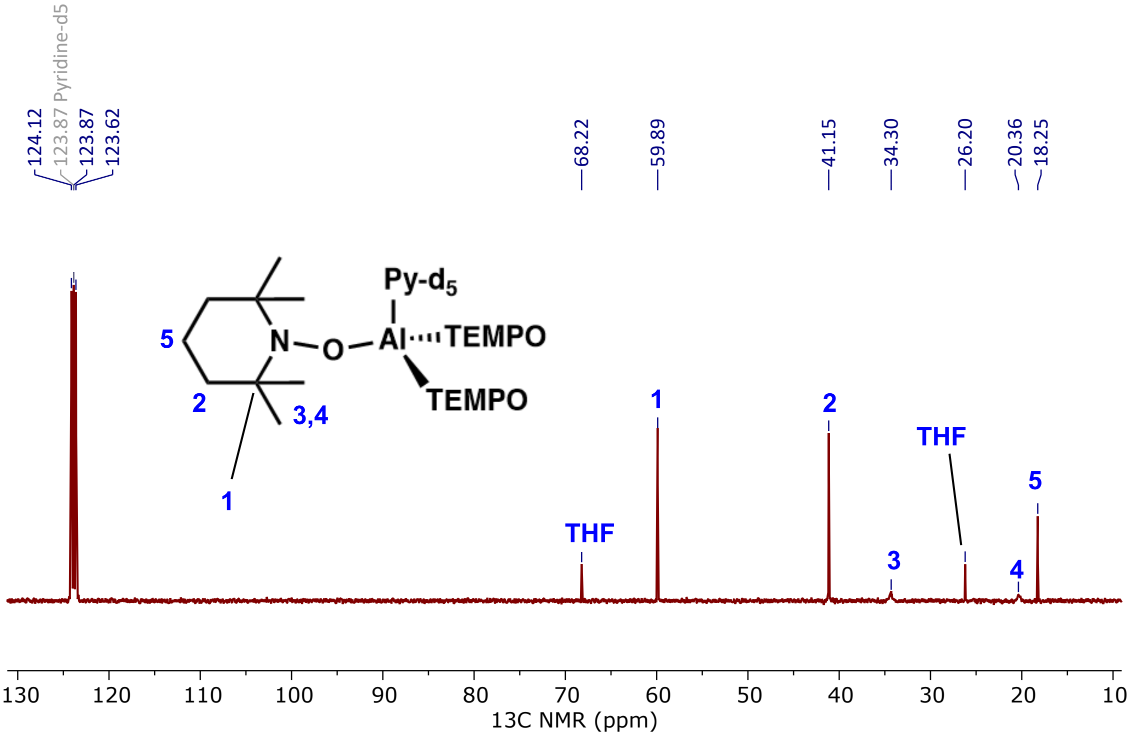

Figure S.12: ^{13}C{^{1}H} NMR (101 MHz, pyridine-d_5, 25 °C) spectrum of **3**·THF.

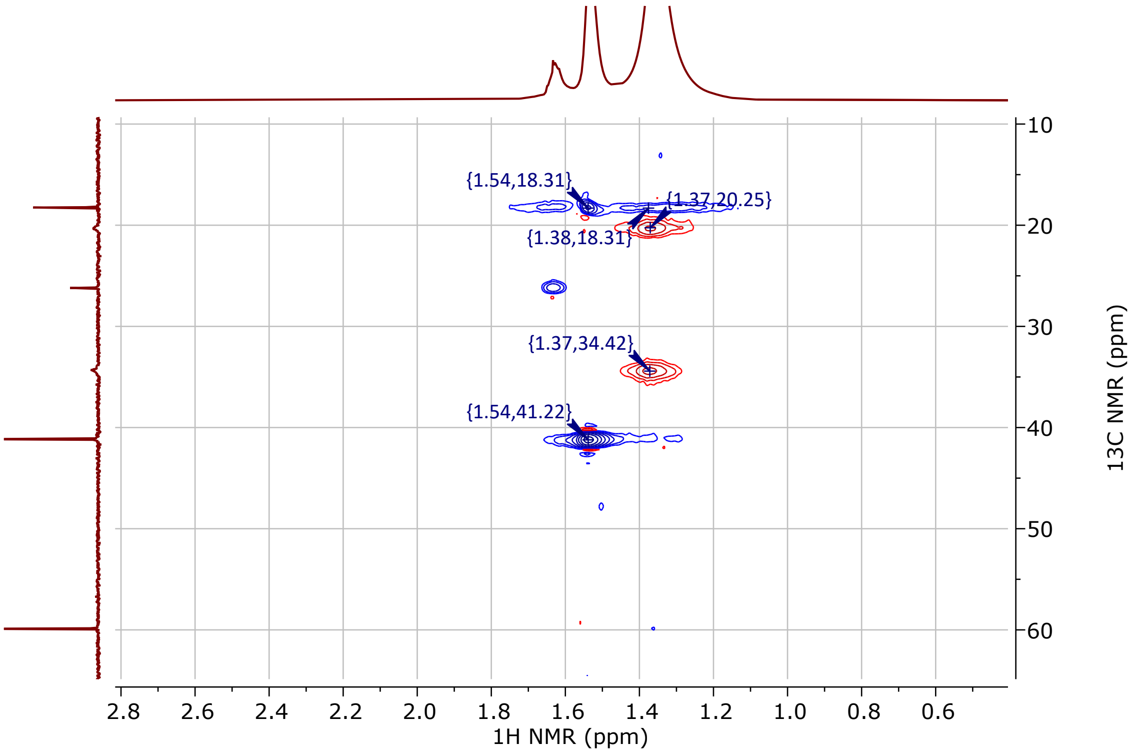

Figure S.13: Multiplicity-edited ^{1}H, ^{13}C-HSQC NMR (400 MHz, pyridine-d_5, 25 °C) spectrum of **3**·THF.

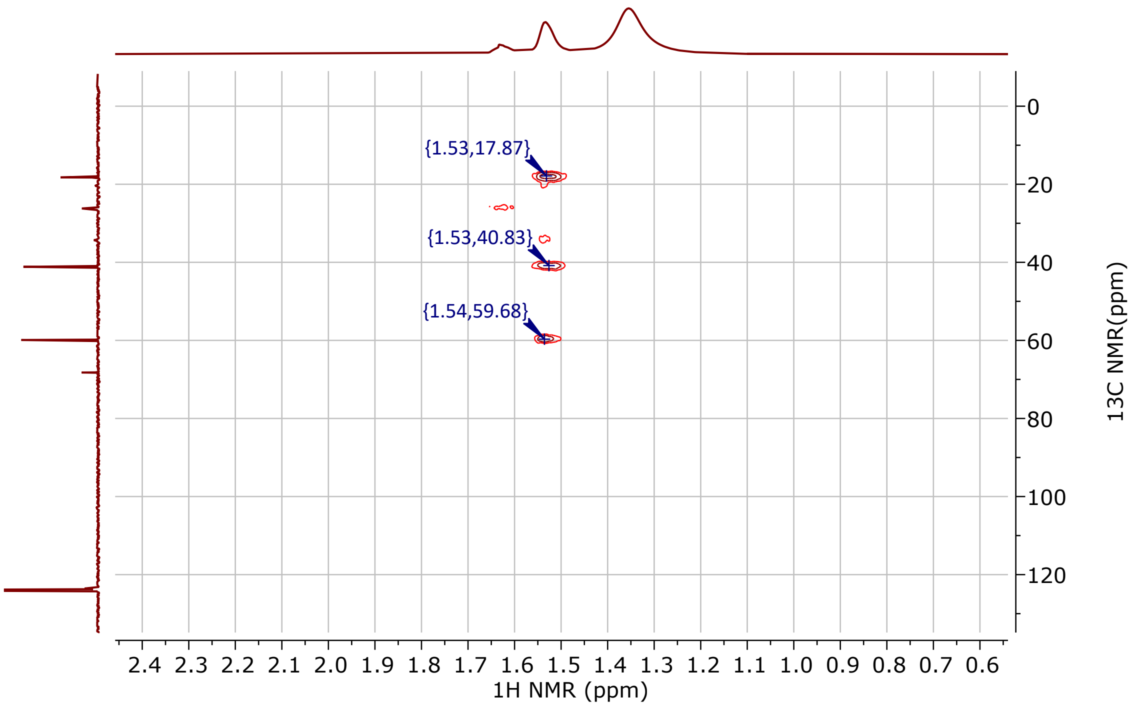

Figure S.14: ^{1}H, ^{13}C-HMBC NMR (400 MHz, pyridine-d_5, 25 °C) spectrum of **3**·THF.

S.1.6 Investigating the Thermal Stability of the Complexes 1·THF, 2·Py, and 3·THF

Separate benzene-d_6 solutions of **1**·THF, **2**·Py, and **3**·THF (0.02 M) were prepared. The solutions were transferred to J Young tubes, heated to 80 °C, and ^{1}H NMR data were collected within 1 h of heating. Complete decomposition was observed for all samples, leading to complex mixtures of unidentified products (Fig. S.15-S.20). Degradation of the complexes may be the result of benzene C−H activation (computational analysis of this pathway is provided in section S.3.1.3). Thermolysis of **2**·Py leads to 2,2,6,6-tetramethylpiperidine (TEMPH) and matches previously reported data.[5]

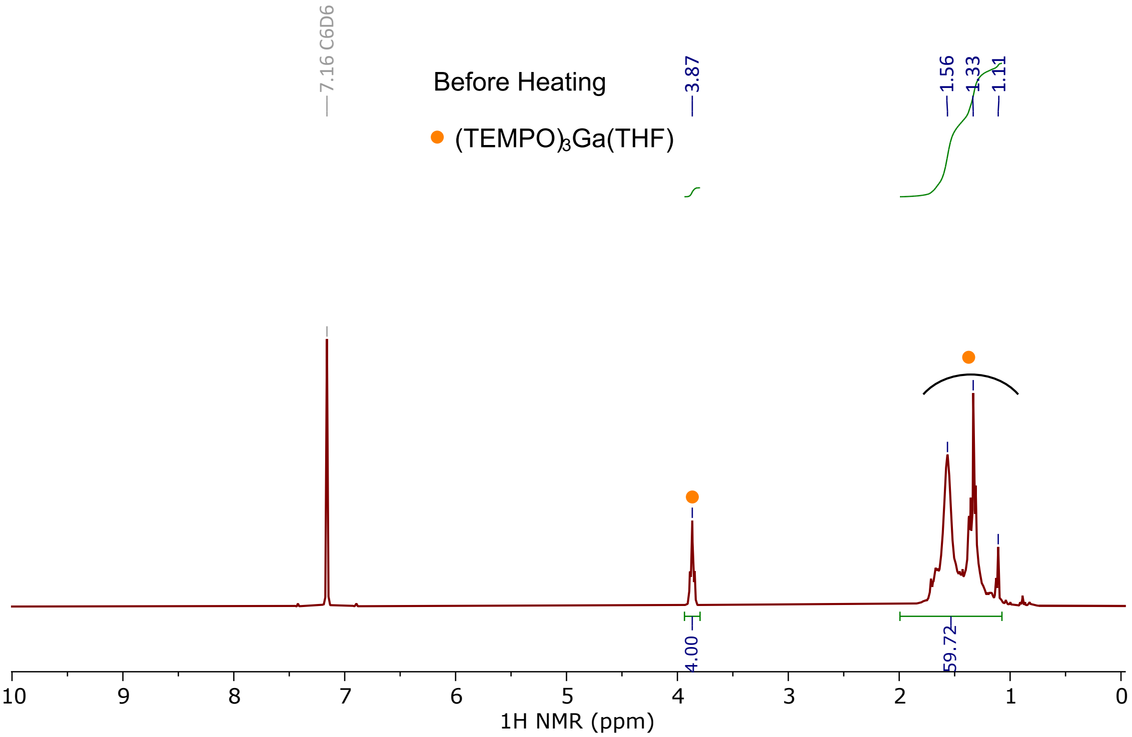

Figure S.15: ^{1}H NMR (400 MHz, benzene-d_6, 25 °C) spectrum of **1**·THF before heating.

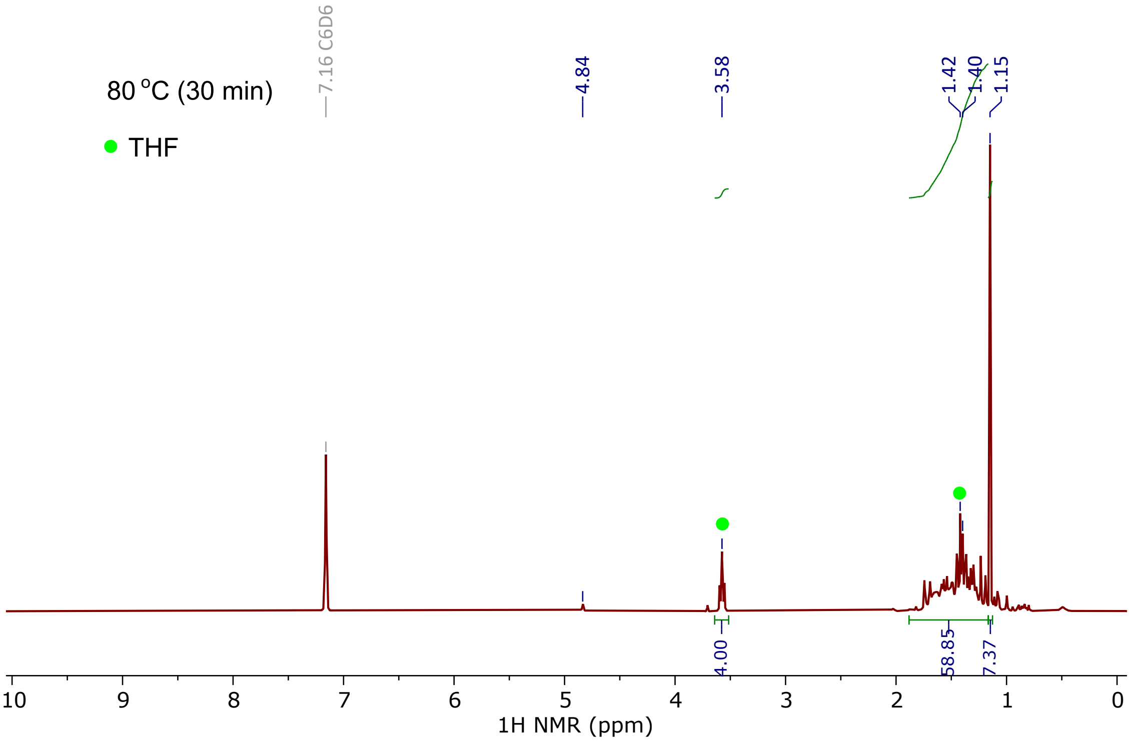

Figure S.16: ^{1}H NMR (400 MHz, benzene-d_6, 25 °C) spectrum of **1**·THF after heating to 80 °C for 30 min.

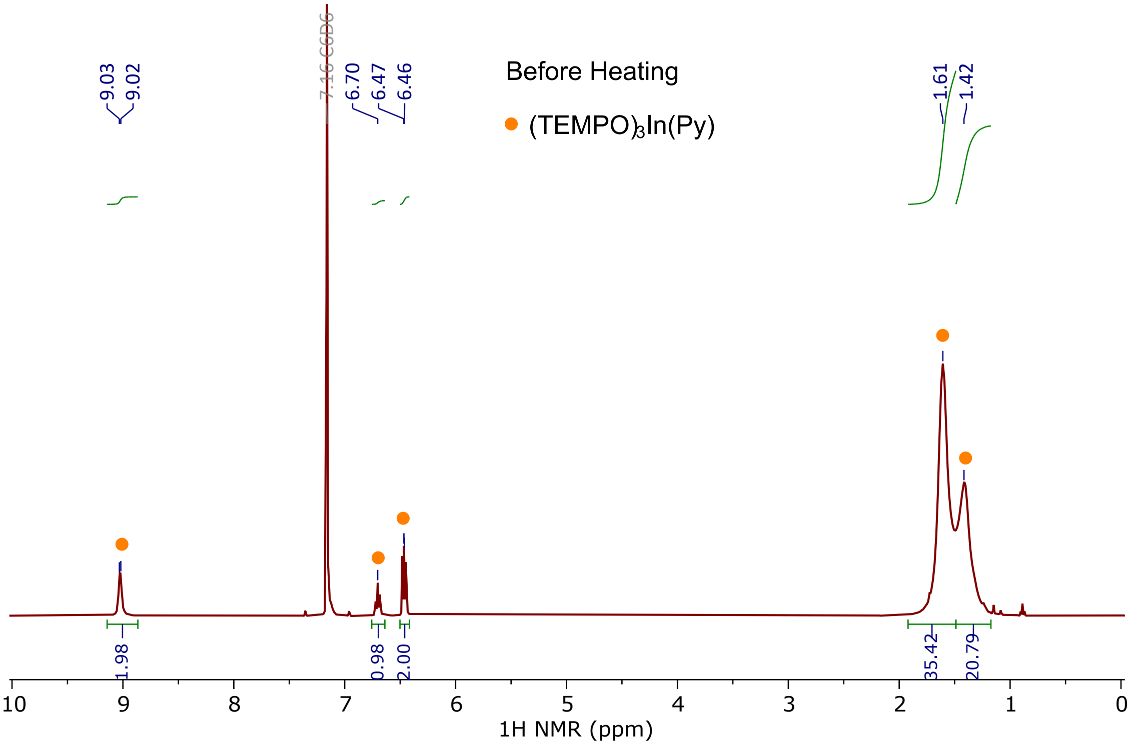

Figure S.17: ^{1}H NMR (400 MHz, benzene-d_6, 25 °C) spectrum of **2**·Py before heating.

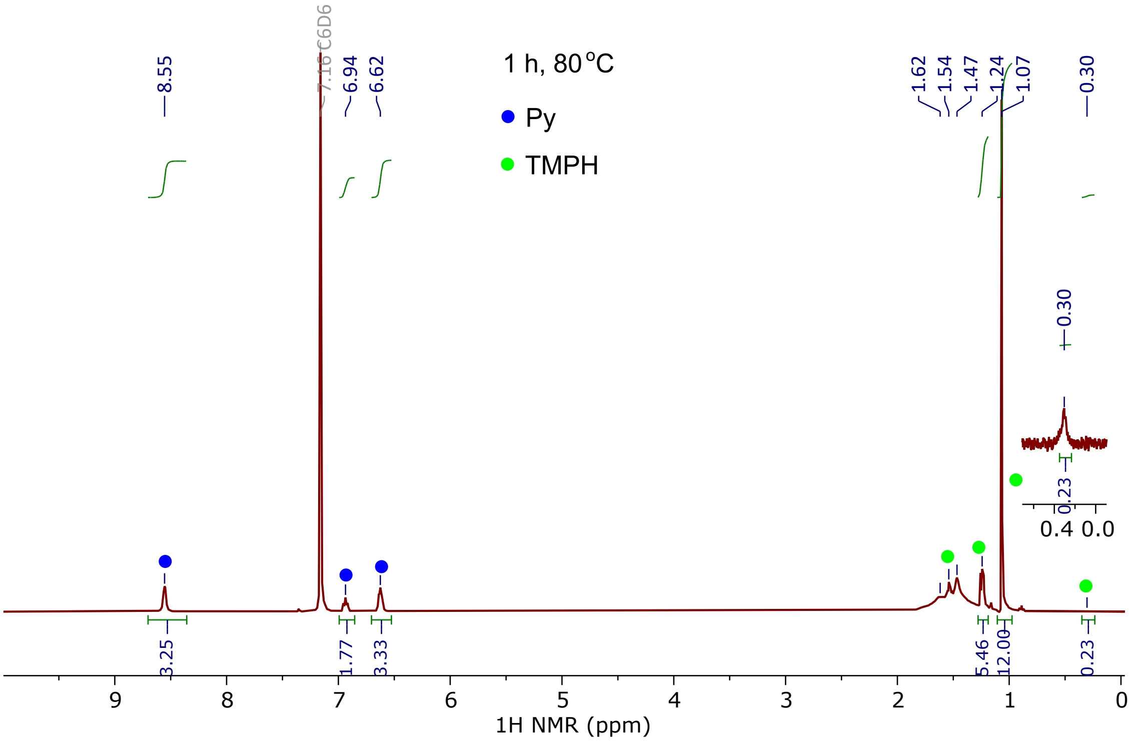

Figure S.18: ^{1}H NMR (400 MHz, benzene-d_6, 25 °C) spectrum of **2**·Py after heating to 80 °C for 1 h. Inset shows zoomed-in region of the NMR spectrum.

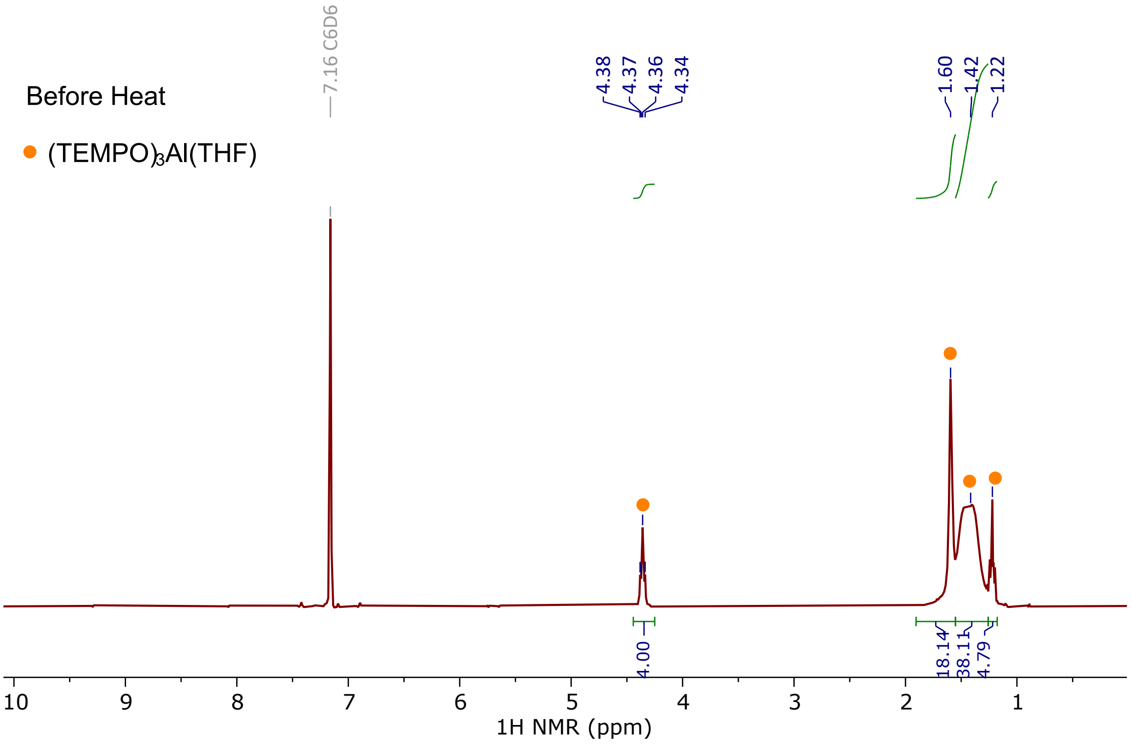

Figure S.19: ^{1}H NMR (400 MHz, benzene-d_6, 25 °C) spectrum of **3**·THF before heating.

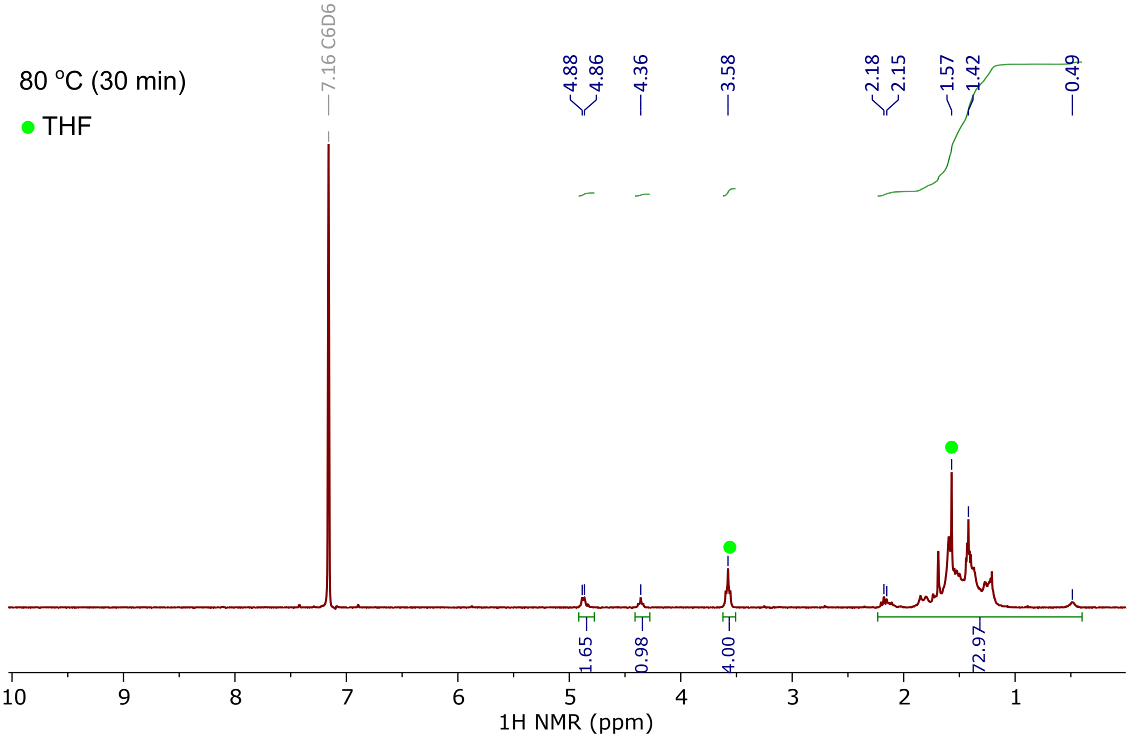

Figure S.20: ^{1}H NMR (400 MHz, benzene-d_6, 25 °C) spectrum of **3**·THF after heating to 80 °C for 30 min.

S.1.7 Gutmann-Beckett Studies

Separate benzene-d_6 solutions of **1**·THF, **2**·Py, and **3**·THF (0.60 mL, 0.017 M, 1.0 equiv) were prepared and transferred to NMR tubes. To each tube was added stock solution of triethylphosphine oxide (TEPO) in benzene-d_6 (0.10 mL, 0.033 M, 0.33 equiv) and analyzed by $^{31}P\{^1H\}$ NMR spectroscopy. Notably, the ^{31}P NMR chemical shift depends on the complex formation equilibrium constant; therefore, a substoichiometric amount of TEPO was used to ensure more complete adduct formation as described previously.[6] Given that the Gutmann-Beckett chemical shifts are also dependent on the solvent used, the experiment was repeated in dichloromethane. A table of the Gutmann-Beckett shifts is provided in Table S.1 and $\Delta\delta^{31}P$ NMR corresponds to the $^{31}P\{^1H\}$ NMR shift of free TEPO subtracted from that of the TEPO-Lewis acid adduct. The NMR data in benzene-d_6 and dichloromethane are shown in Fig. S.21 and Fig. S.22, respectively.

Table S.1: Table of Gutmann-Beckett Shifts

Lewis Acid	$\Delta\delta^{31}P$ NMR (ppm, C_6D_6)	$\Delta\delta^{31}P$ NMR (ppm, CH_2Cl_2)
**3**·THF	21.1	17.4
**1**·THF	20.1	16.7
**2**·Py	18.3	14.9

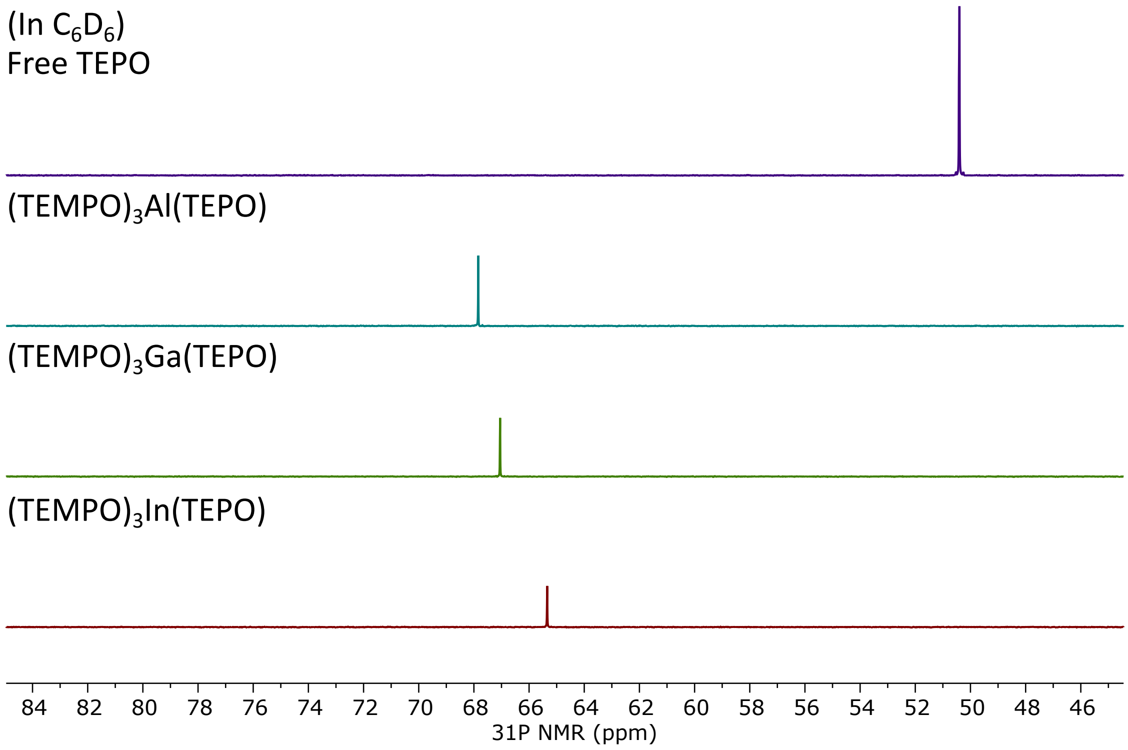

Figure S.21: Stacked $^{31}P\{^1H\}$ NMR (243 MHz, benzene-d_6, 25 °C) spectra of Gutmann-Beckett experiments.

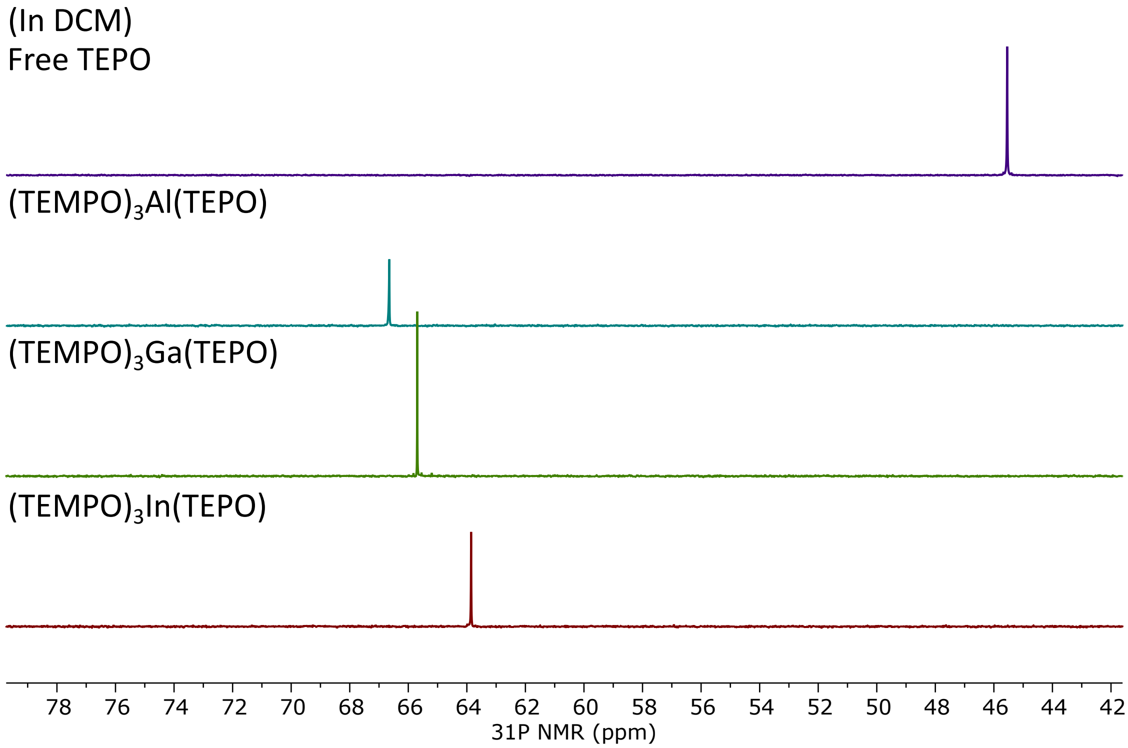

Figure S.22: Stacked $^{31}P\{^{1}H\}$ NMR (243 MHz, CH_2Cl_2, 25 °C) spectra of Gutmann-Beckett experiments.

S.1.8 Synthesis of $(H_2)Ga(TEMPO)_3$ (4)

1·THF (0.200 g, 0.328 mmol, 1 equiv) was dissolved in *n*-hexane (15 mL) and the solution was transferred to a Schlenk storage flask (50 mL) containing a stirbar. The solution was removed from the glovebox and degassed by three freeze-pump-thaw cycles. The flask was then backfilled with H_2 (*ca.* 3 atm, 6 mmol, 20 equiv) and set to stir for 1 h, during which time colorless crystalline material crashed out of solution. The flask was brought back into the glovebox where all volatile materials were removed under reduced pressure, providing a colorless solid. The solids were dissolved in diethyl ether (3 mL) and filtered through a pipette containing a plug of glass microfiber filter paper. The filtrate was placed in a −35 °C freezer for 2 days for crystallization. Complex **4** was isolated as a colorless, crystalline material (0.125 g, 0.204 mmol, 62% yield). ^{1}H NMR (600 MHz, pyridine-d_5, 25 °C, Fig. S.23) δ 8.88 (N−H), 5.72 (Ga−H), 1.53, 1.47, 1.33, 1.28, 1.15 ppm. ^{13}C{^{1}H} NMR (101 MHz, pyridine-d_5, 25 °C, Fig. S.24) δ 59.51, 58.84, 40.84, 40.38, 34.76 (br), 26.76 (br), 19.66 (br), 18.30, 13.10 ppm. Elem. Anal. Calc'd(found) for $C_{27}H_{56}N_3O_3Ga$: C 60.00(59.70), H 10.44(10.45), N 7.77(7.76).

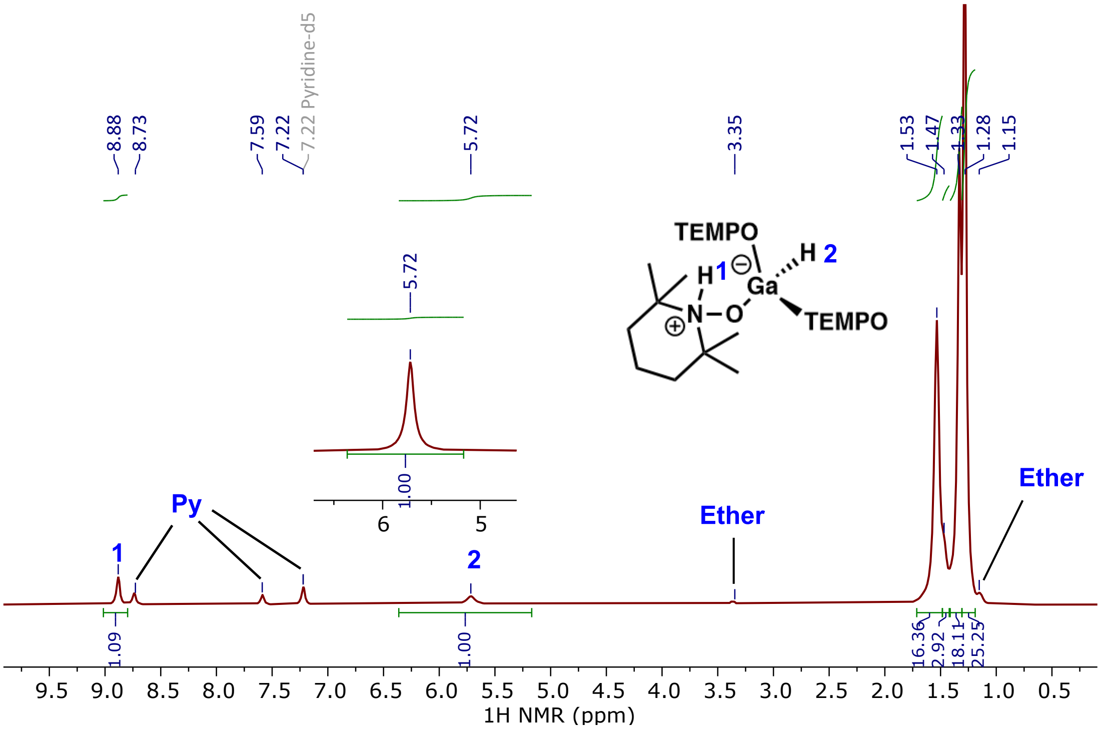

Figure S.23: ^{1}H NMR (500 MHz, pyridine-d_5, 25 °C) spectrum of **4**. Inset shows a zoomed-in region of the ^{1}H NMR spectrum.

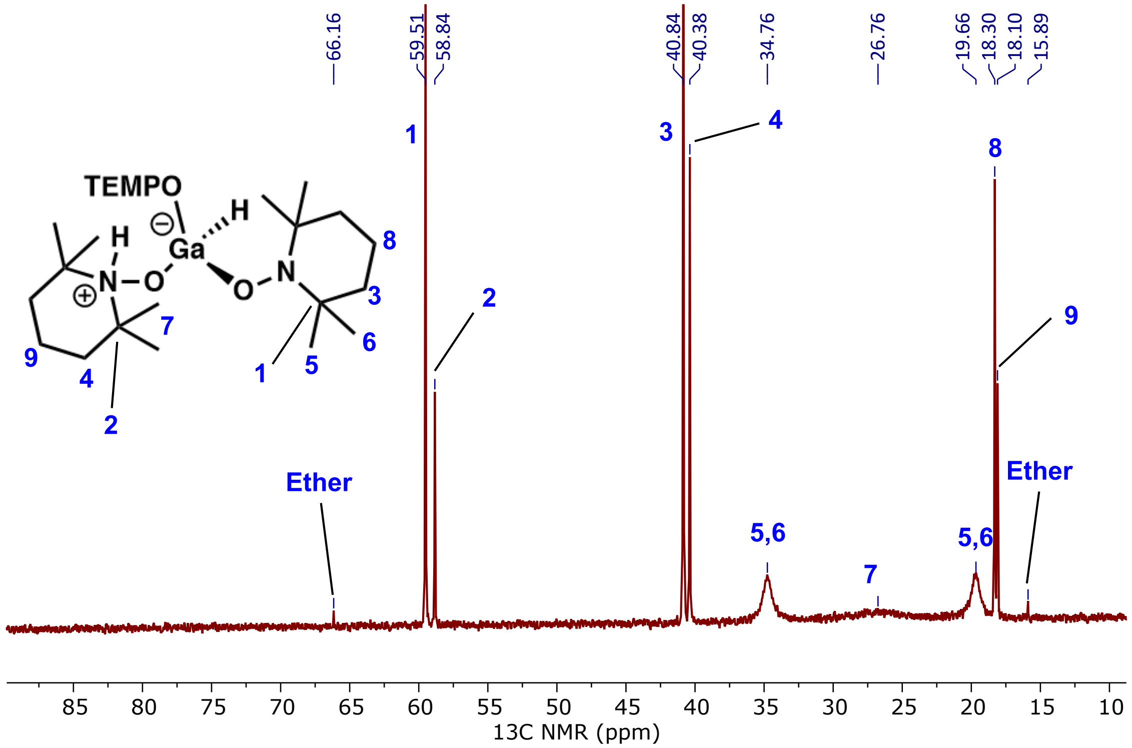

Figure S.24: ^{13}C{^{1}H} NMR (126 MHz, pyridine-d_5, 25 °C) spectrum of **4**.

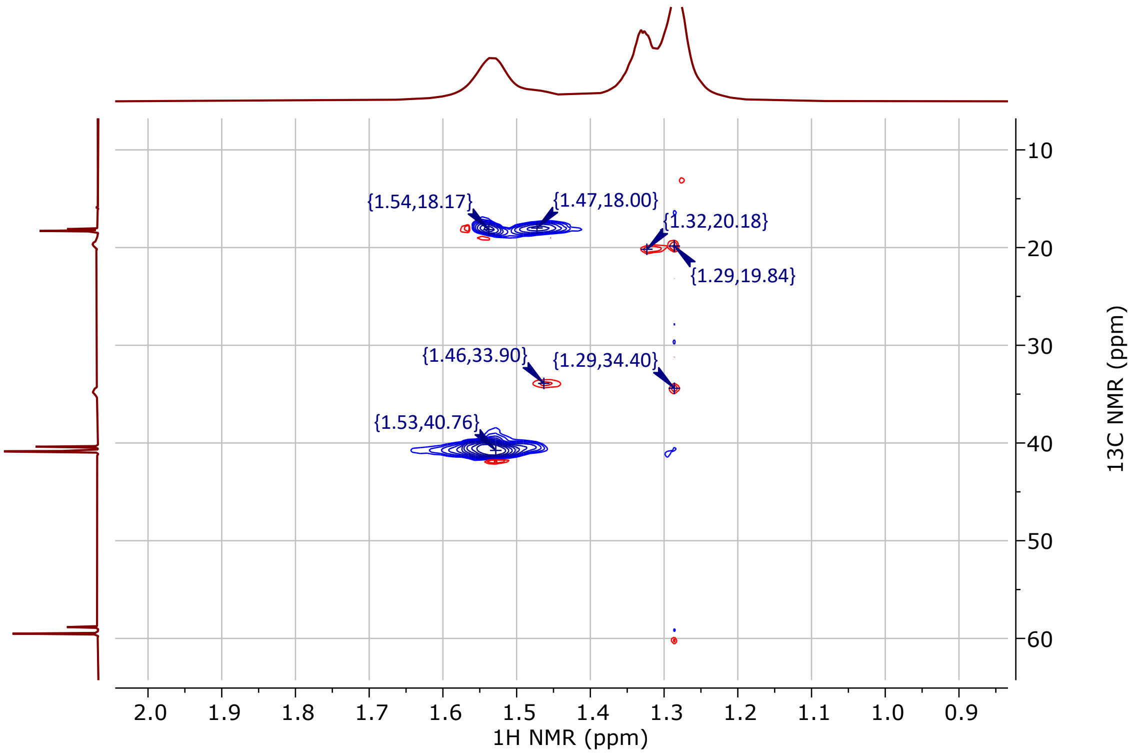

Figure S.25: Multiplicity-edited ^{1}H, ^{13}C-HSQC NMR (400 MHz, pyridine-d_5, 25 °C) spectrum of **4**.

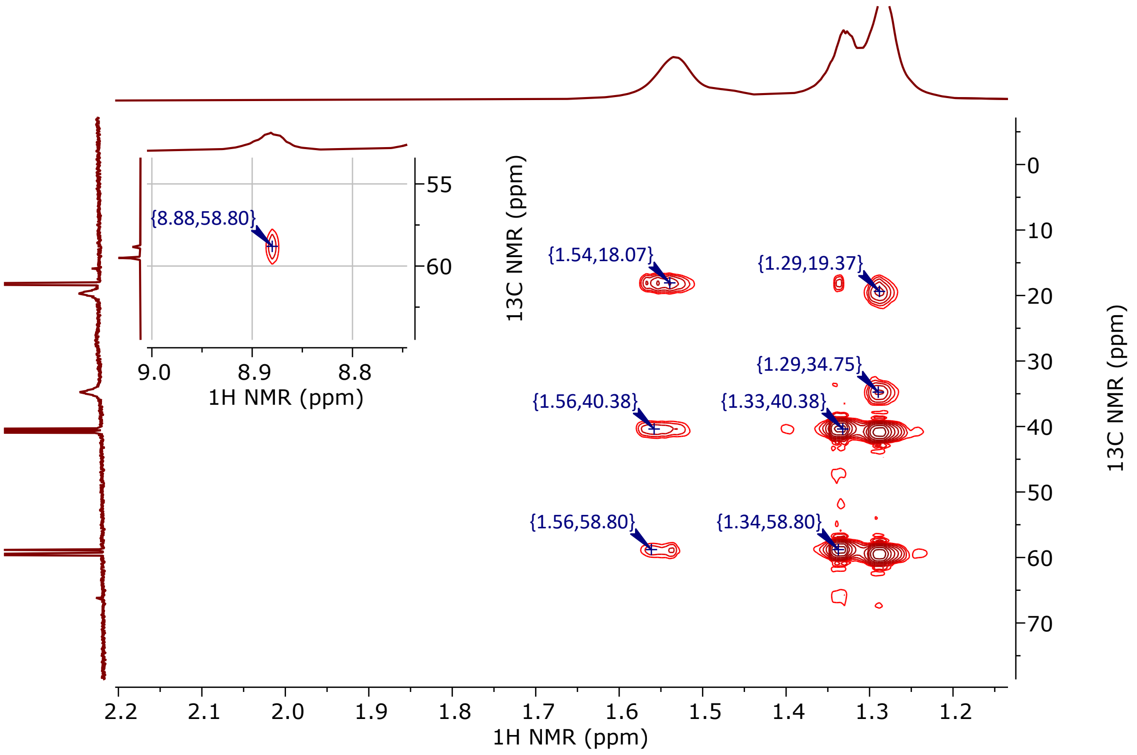

Figure S.26: ^{1}H, ^{13}C-HMBC NMR (400 MHz, pyridine-d_5, 25 °C) spectrum of **4**. Inset shows a zoomed-in region of the ^{1}H, ^{13}C-HMBC NMR spectrum.

S.1.8.1 Vibrational Analysis of the Crude Reaction Mixture

1·THF (0.005 g, 0.008 mmol, 1 equiv) was dissolved in benzene-d_6 (0.5 mL) and the solution was transferred to a J Young tube (2 mL). The solution was removed from the glovebox and degassed by three freeze-pump-thaw cycles. The tube was backfilled with H_2 (*ca.* 2 atm, 0.2 mmol, 25 equiv) and after 10 min, NMR data was collected to confirm the generation of **4**. The solution was transferred into a liquid IR transmission cell in the glovebox and IR data was collected. The data is shown in Fig. S.27. Bands at 3568 and 1929 cm^{-1} were observed and correspond to the N−H and Ga−H stretch of **4**, respectively. Assignments were confirmed *via* a deuterium labeling experiment (see section S.1.8.2).

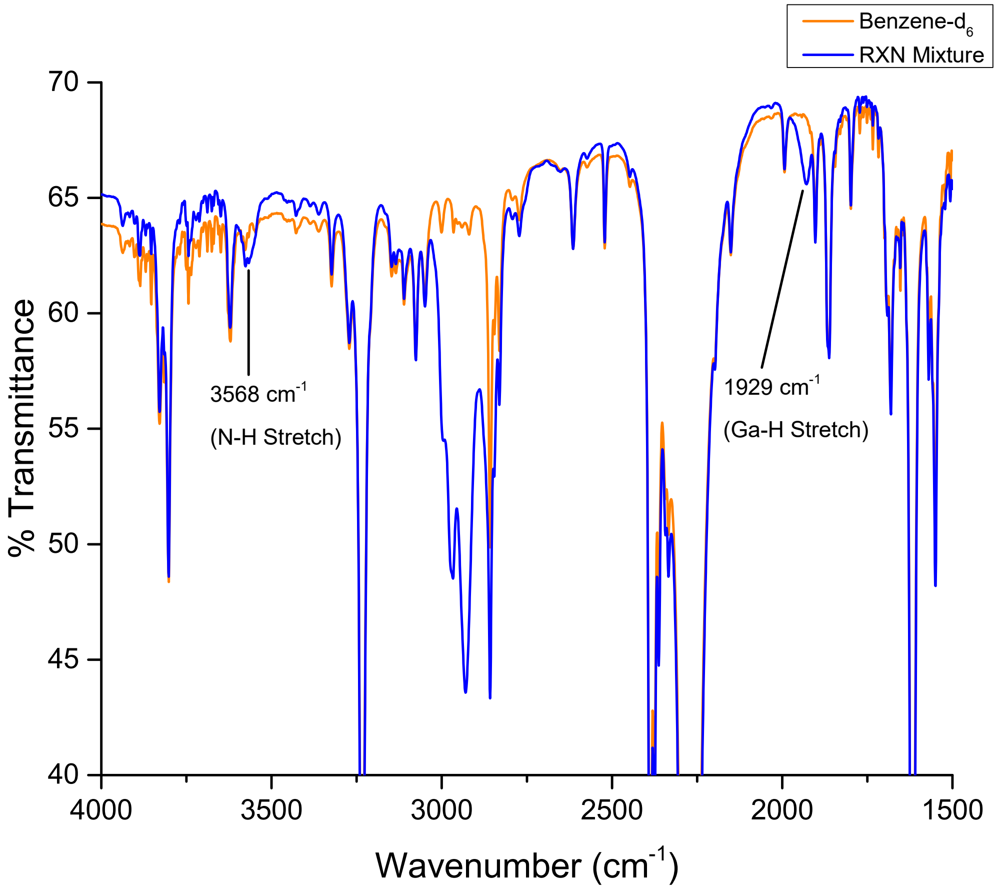

Figure S.27: Overlayed IR spectra of benzene-d_6 (orange) and generated **4** in benzene-d_6 (blue).

S.1.8.2 Treatment of **1**·THF with D_2

1·THF (0.005 g, 0.008 mmol, 1 equiv) was dissolved in benzene-d_6 (0.5 mL) and the solution was transferred to a J Young tube (2 mL). The solution was removed from the glovebox and degassed by three freeze-pump-thaw cycles. The tube was backfilled with D_2 (*ca.* 2 atm, 0.2 mmol, 20 equiv) and after 10 min, ^{1}H NMR and ^{2}H NMR spectra were collected (Fig. S.28 and Fig. S.29). No notable changes were observed after 1 h. The NMR sample was transferred into a liquid IR transmission cell in the glovebox and IR data was collected (Fig. S.30). Notably, treatment of **1**·THF with D_2 in place of H_2 leads to the loss of vibrational bands assigned to the N−H (3568 cm^{-1}) and Ga−H (1929 cm^{-1}) substituents of **4**. However, we are unable to identify vibrational bands associated with the N−D and Ga−D substituents of $(D_2)Ga(TEMPO)_3$ possibly due to overlapping signals.

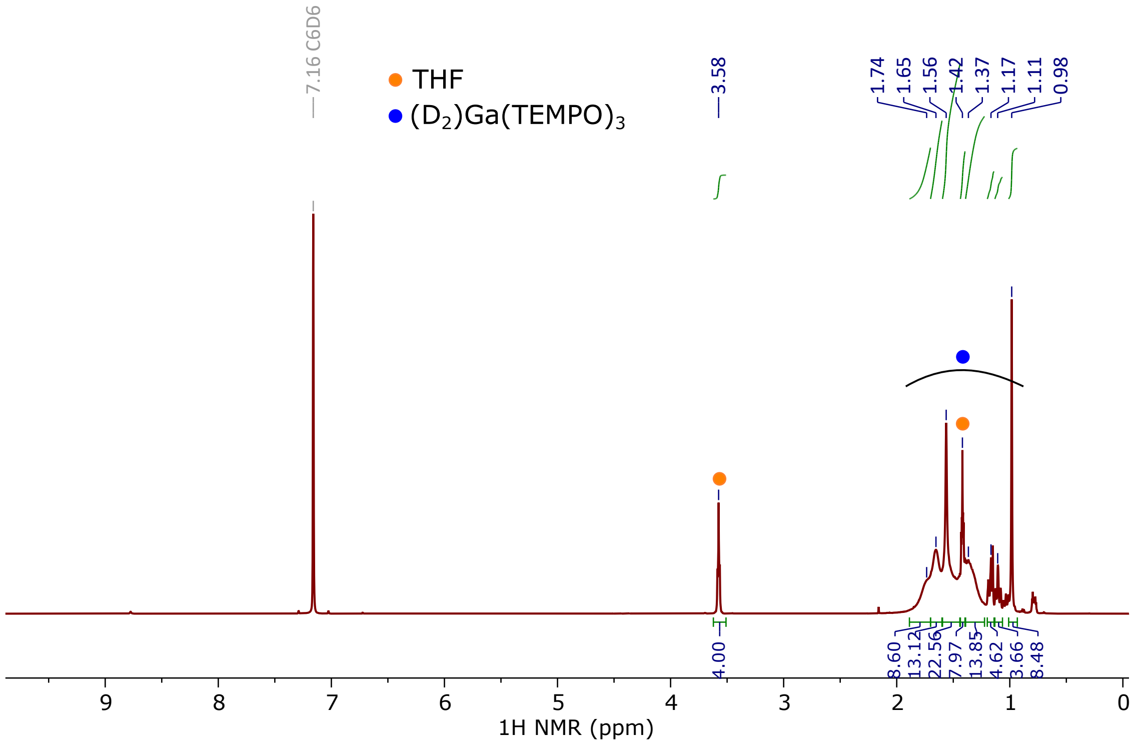

Figure S.28: ^{1}H NMR (600 MHz, benzene-d_6, 25 °C) spectrum of **1**·THF treated with D_2.

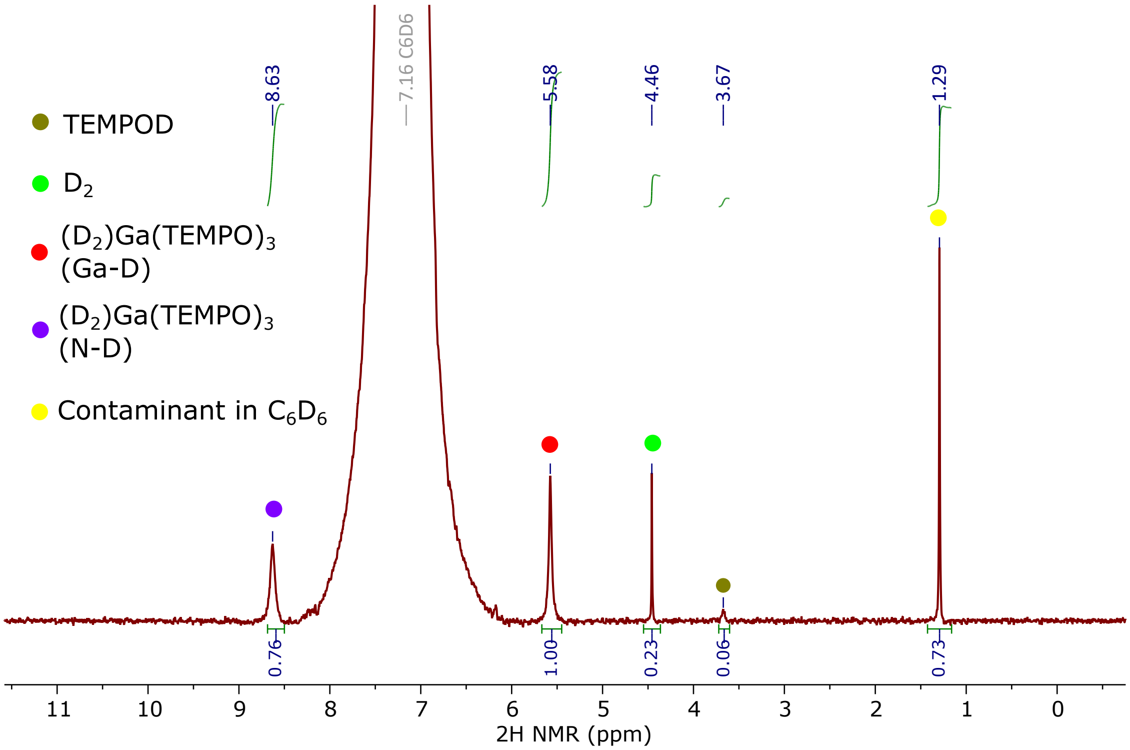

Figure S.29: ^{2}H NMR (353 MHz, benzene-d_6, 25 °C) spectrum of **1**·THF treated with D_2. The resonance at 1.29 ppm is present in benzene-d_6 received from Cambridge Isotope Laboratories and possibly corresponds to cyclohexane-d_{12}.

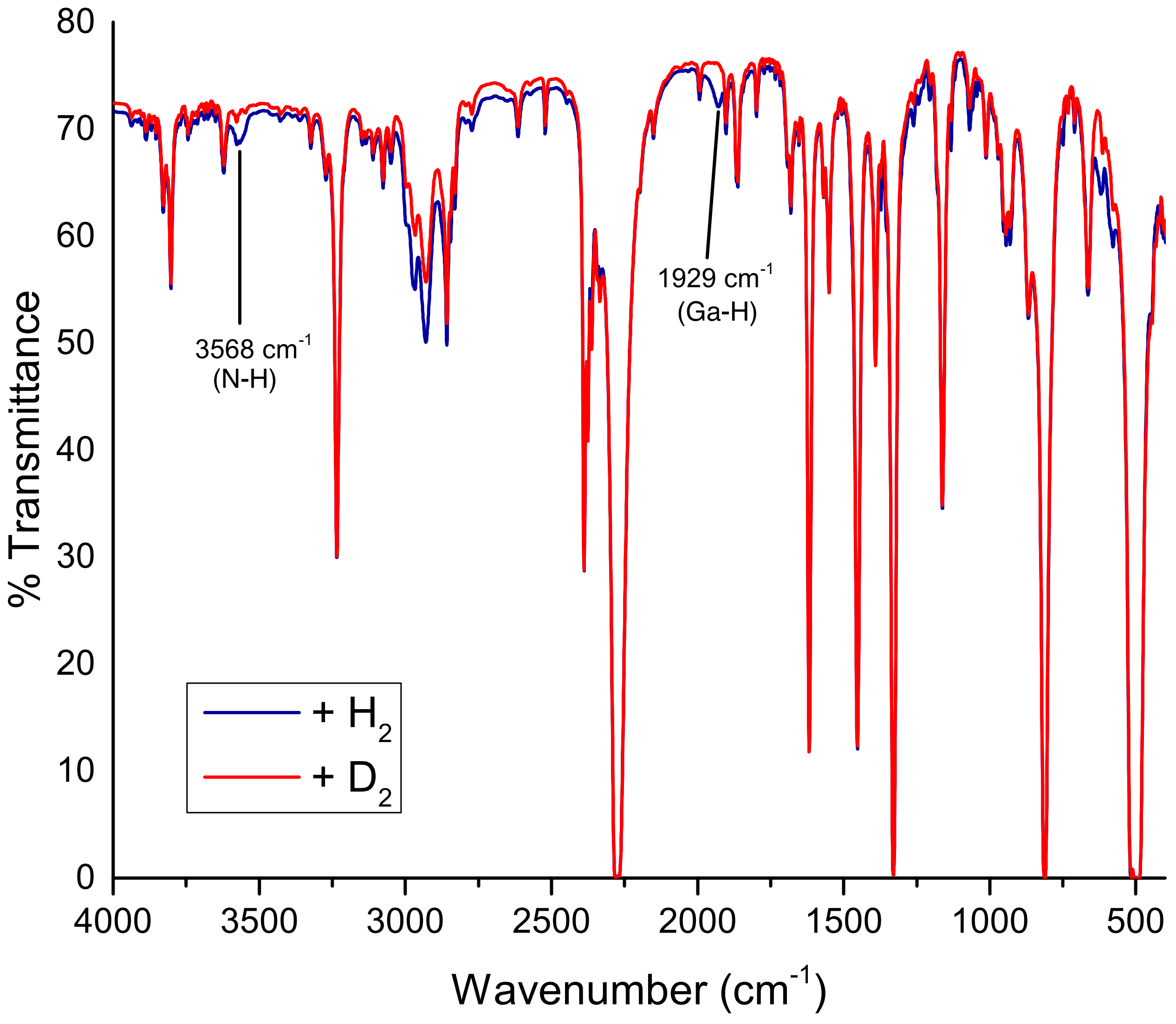

Figure S.30: Overlayed IR spectra of **1**·THF treated with H_2 (dark blue) or D_2 (red) in benzene-d_6.

The IR spectrum of $(D_2)Ga(TEMPO)_3$ was calculated and the simulated spectrum is provided below. The calculation suggests that the Ga−D and N−D stretches exhibit bands at 1304 cm^{-1} and 2325 cm^{-1}, respectively. The predicted values overlap with intense vibrational bands coming from the benzene-d_6 solvent (Fig. S.32), consistent with our inability to identify these stretches experimentally. Note that the simulated spectrum may differ from the experimental spectrum based on solvent effects (an implicit solvation model was used), no vibrational correction factors, and contributions from different conformers (follows Boltzman distribution).

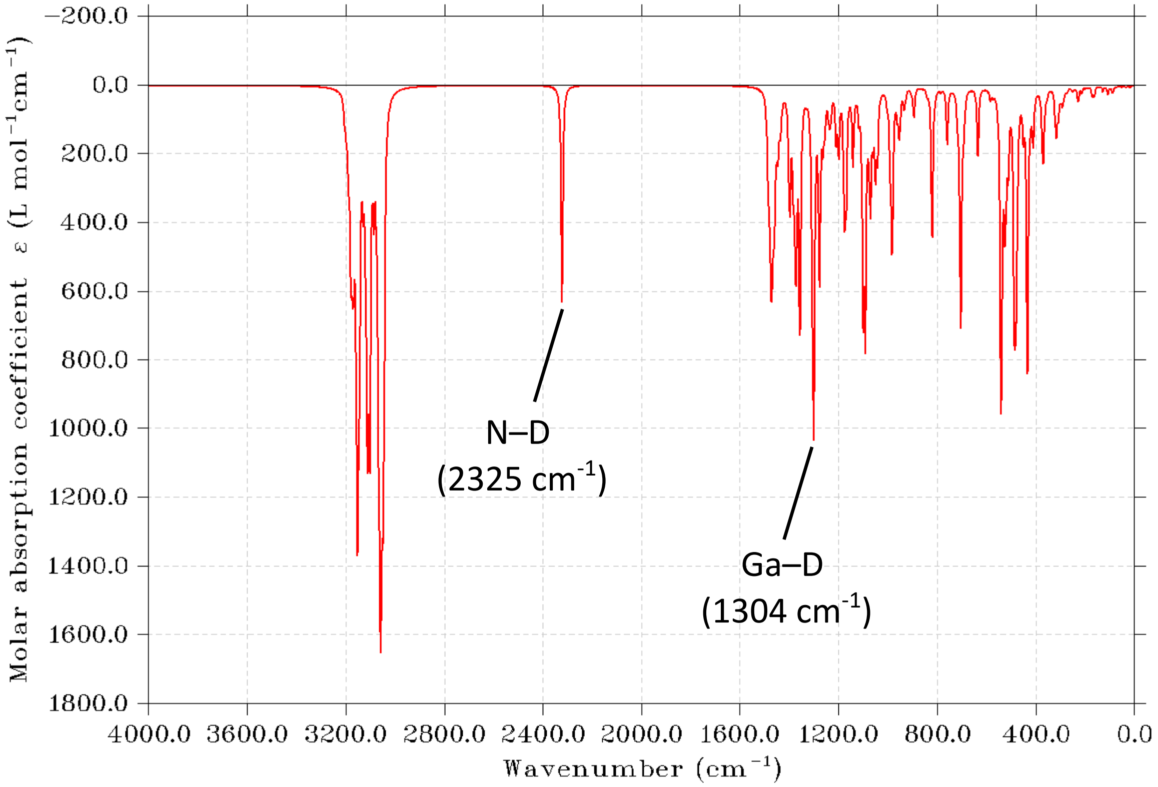

Figure S.31: DFT calculated IR spectrum of $(D_2)Ga(TEMPO)_3$.

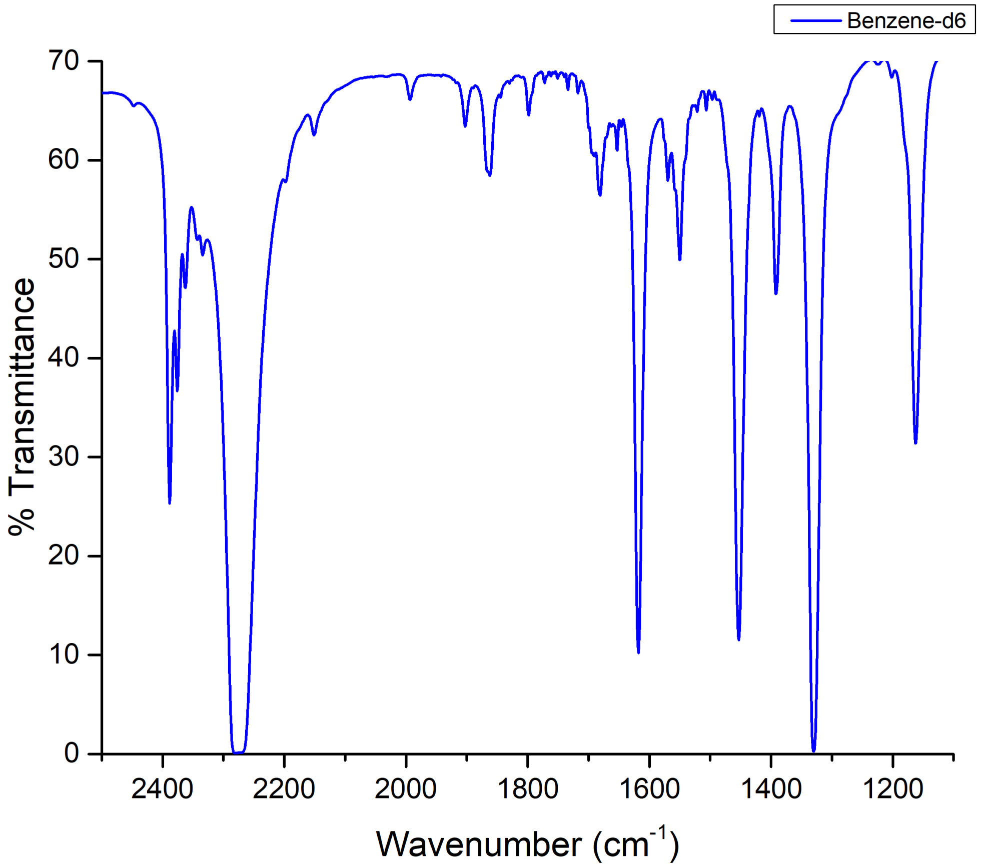

Figure S.32: IR spectra of benzene-d_6.

S.1.8.3 Quantification of Conversion using an Internal Standard

Prior to running this experiment, spin-lattice decay constants (T_1) were measured using an inversion recovery pulse sequence and T_1 times of less than 2.2 s (3.98 ppm resonance for **1**·THF, Fig. S.33), 4.3 s (3.31 ppm resonance for 1,3,5-trimethoxybenzene, Fig. S.33), 1.15 s (8.78 ppm resonance for **4**, Fig. S.34), and 1.15 s (5.55 ppm resonance for **4**, Fig. S.34). Therefore, all ^{1}H NMR spectra were recorded with a relaxation delay set to 30 s to obtain quantitative ^{1}H NMR data.

1·THF (0.010 g, 0.016 mmol, 1.0 equiv) and 1,3,5-trimethoxybenzene (0.0028 g, 0.016 mmol, 1.0 equiv) were dissolved in benzene-d_6 (0.5 mL) and the solution was transferred to a J Young tube (2 mL). An initial quantitative ^{1}H NMR spectrum of the mixture was

collected and is shown in Fig. S.35. The solution was degassed by three freeze-pump-thaw cycles and backfilled with H_2 (*ca.* 3 atm) and after 1 h, quantitative ^{1}H NMR data were collected (Fig. S.36). No notable changes were observed after the sample sat for *ca.* 16 h. The ^{1}H NMR data (based on the well-resolved integrated resonance at 8.78 ppm) indicates 90% conversion of **1**·THF to **4**.

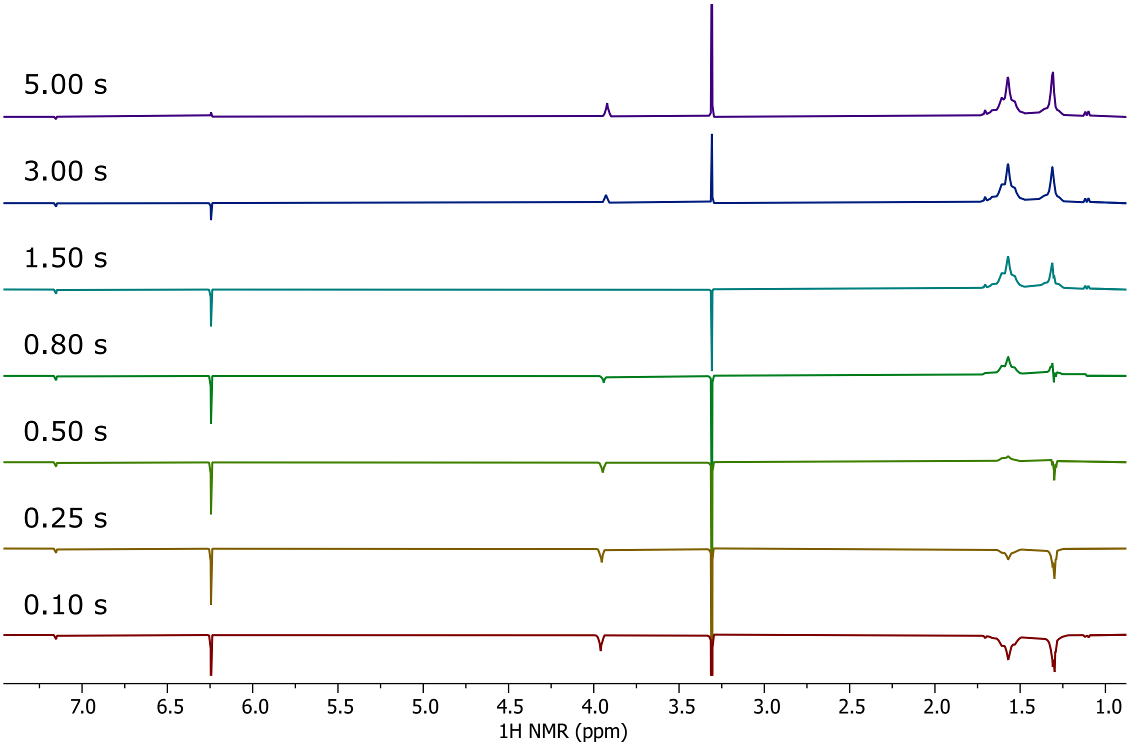

Figure S.33: Stacked ^{1}H NMR spectra of **1**·THF and 1,3,5-trimethoxybenzene in benzene-d_6 and associated relaxation delay times (D1) from an inversion recovery experiment.

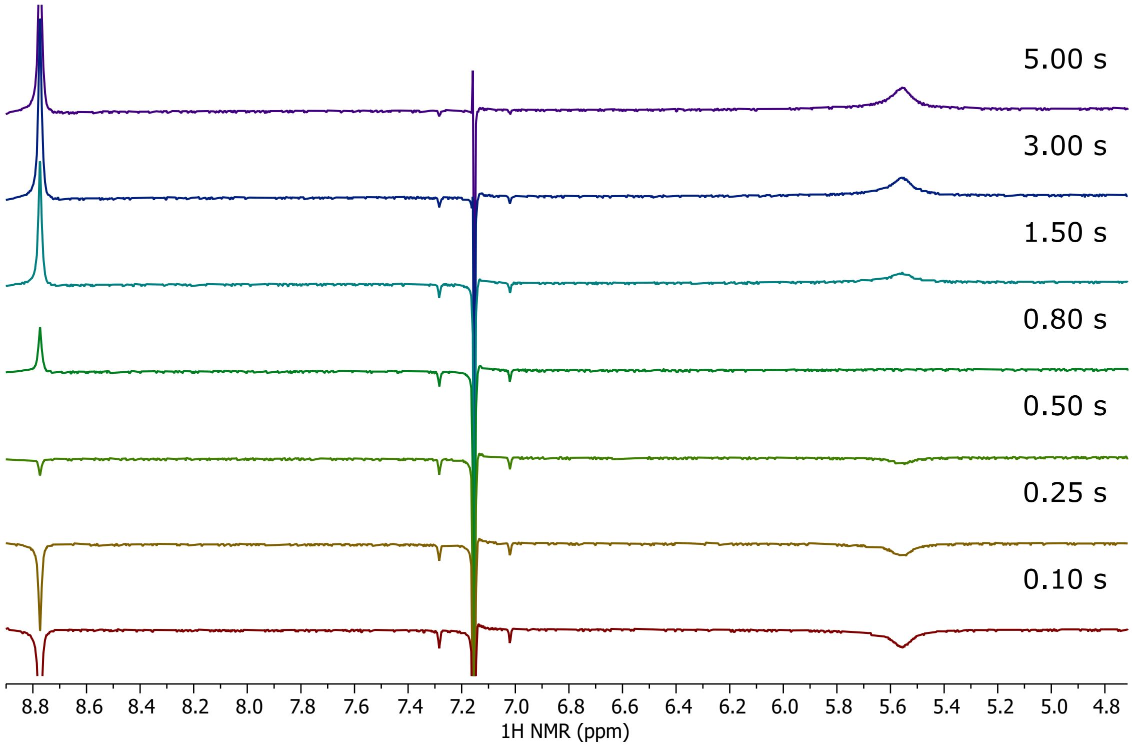

Figure S.34: Stacked ^{1}H NMR spectra of **4** in benzene-d_6 and associated relaxation delay times (D1) from an inversion recovery experiment.

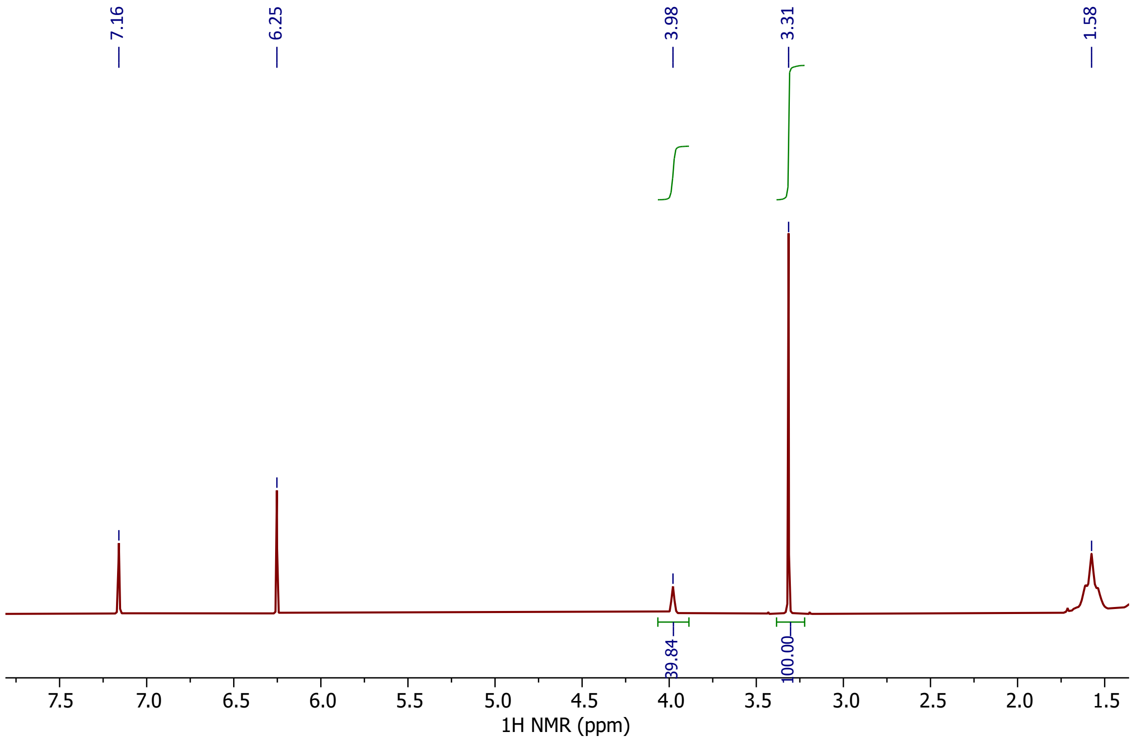

Figure S.35: Quantitative ^{1}H NMR (600 MHz, benzene-d_6, 25 °C) spectrum of **1**·THF and 1,3,5-trimethoxybenzene.

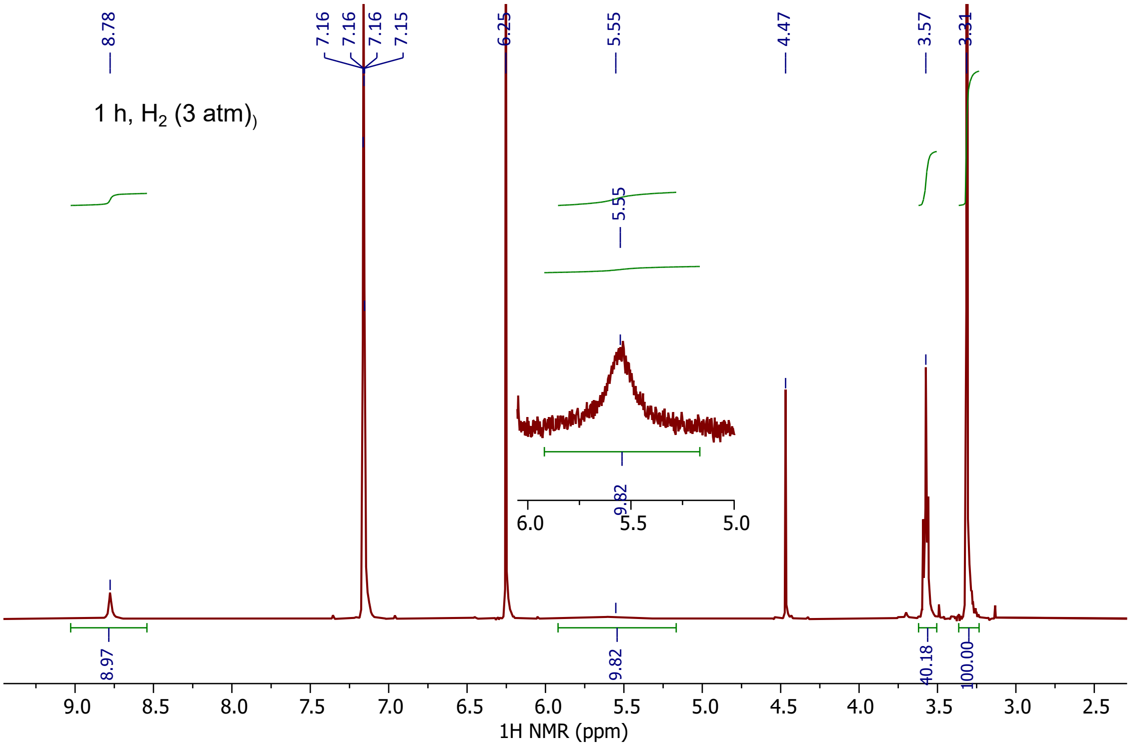

Figure S.36: Quantitative ^{1}H NMR (600 MHz, benzene-d_6, 25 °C) spectrum of generated **1**·THF and 1,3,5-trimethoxybenzene.

S.1.8.4 Treatment of 1·THF with H_2 in pyridine-d_5

1·THF (0.010 g, 0.016 mmol, 1.0 equiv) was dissolved in pyridine-d_5 (0.5 mL) and the solution was transferred to a J Young tube (2 mL). The solution was degassed by three freeze-pump-thaw cycles and backfilled with H_2 (*ca.* 3 atm) and after 3 h, ^{1}H NMR data were collected (Fig. S.37). Partial conversion to **4** was observed.

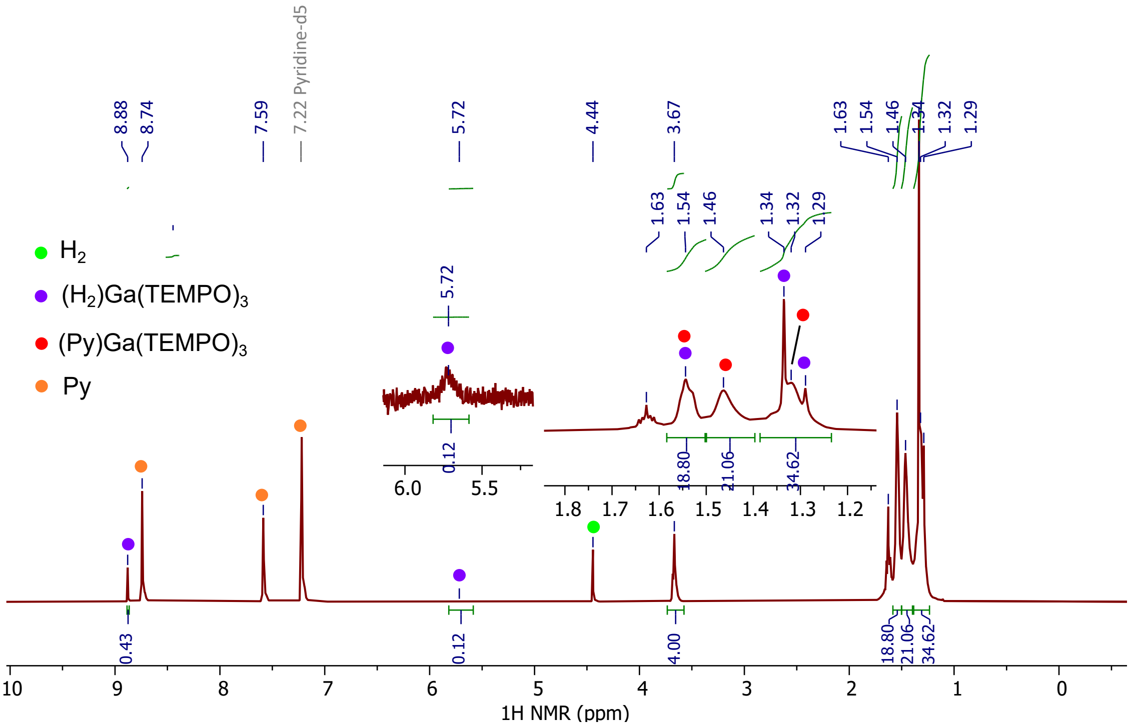

Figure S.37: ^{1}H NMR (400 MHz, pyridine-d_5, 25 °C) spectrum of **1**·THF 3 h after adding H_2 (*ca.* 3 atm).

S.1.9 Investigating the Thermal Stability of 4

4 (0.005 g, 0.009 mmol) was dissolved in pyridine-d_5 and the solution was heated for 20 h to 80 °C. New resonances corresponding to **1**·Py were observed, consistent with the loss of H_2 from **4** (Fig. S.38 and S.39). Notably, the originally broad Ga−H resonance at 5.7 ppm becomes a sharp singlet, making our assignment of **4** here tentative. After heating the sample to 80 °C for a total of 44 h, changes in the relative integration of the resonances are

observed in the ^{1}H NMR spectrum (Fig. S.40). For reference, stacked spectra of **4**, heated **4**, and pristine **1**·Py are provided in Fig. S.41.

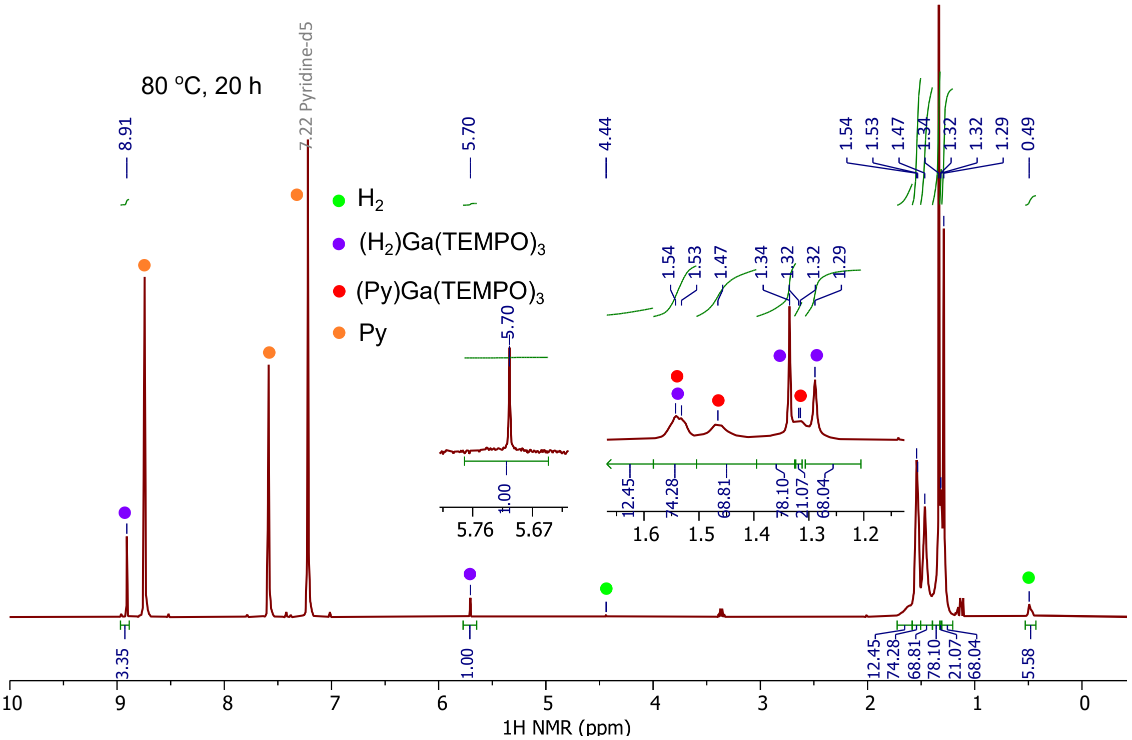

Figure S.38: ^{1}H NMR (400 MHz, pyridine-d_5, 25 °C) spectrum of **4** after being heated to 80 °C for 20 h.

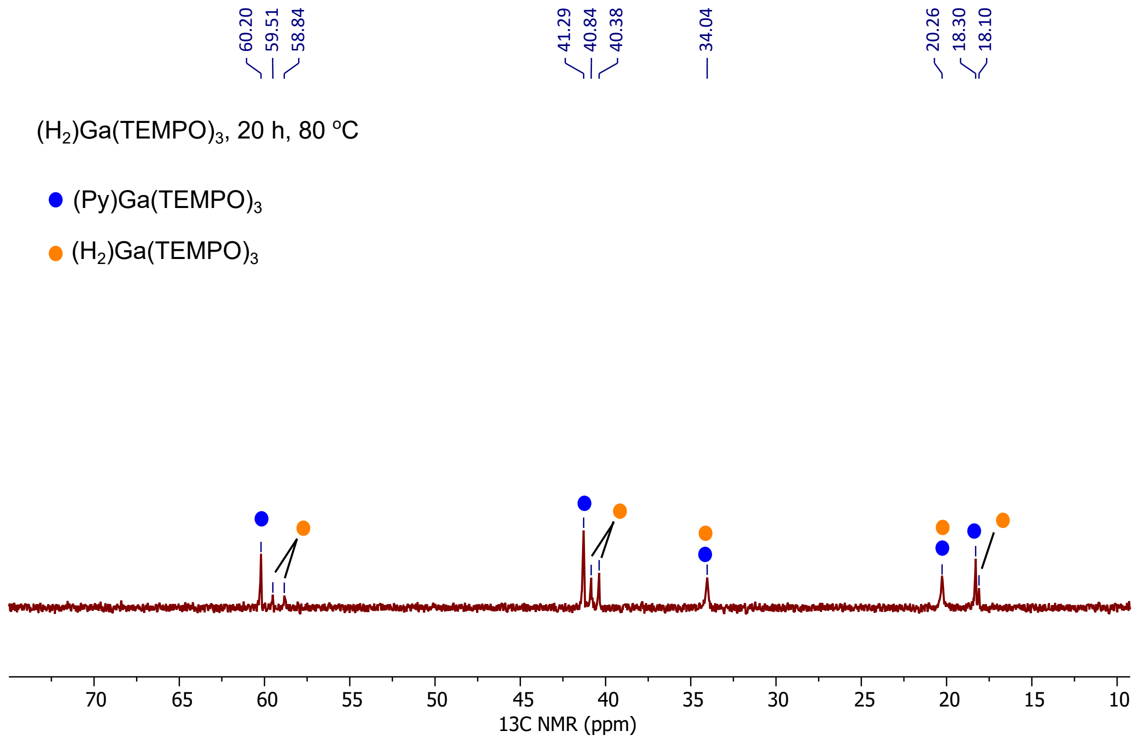

Figure S.39: $^{13}C\{^1H\}$ NMR (126 MHz, pyridine-d_5, 25 °C) spectrum of **4** after being heated to 80 °C for 20 h.

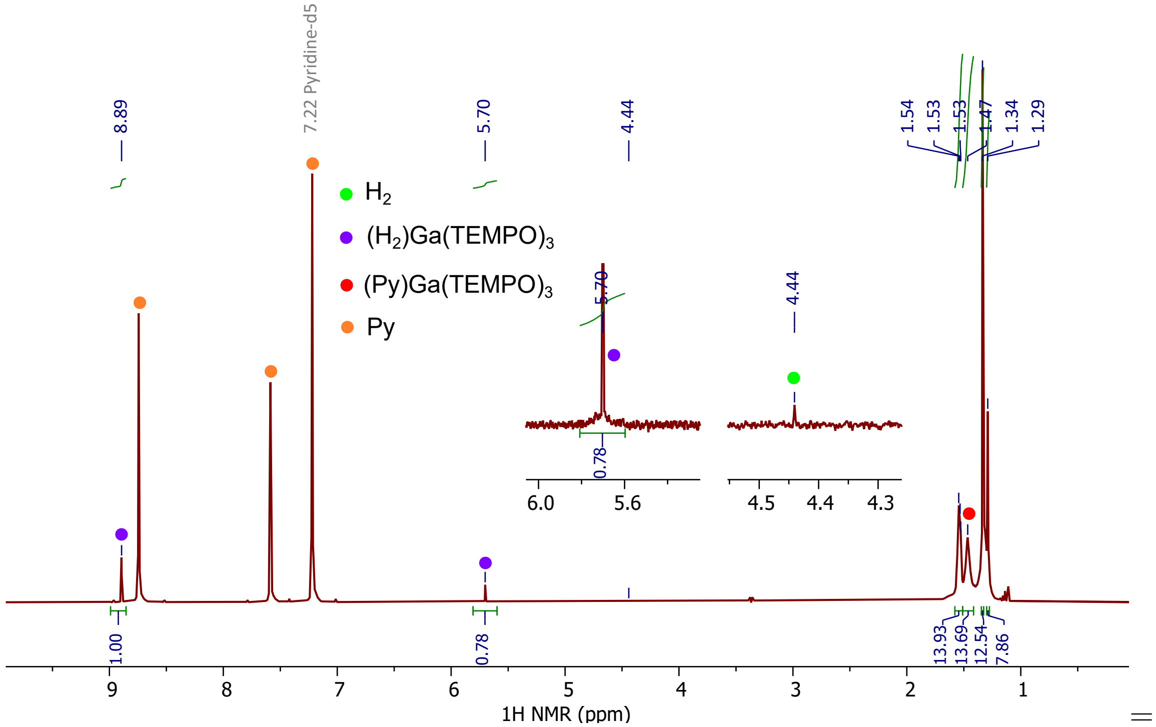

Figure S.40: ^1H NMR (400 MHz, pyridine-d_5, 25 °C) spectrum of **4** after being heated to 80 °C for 44 h.

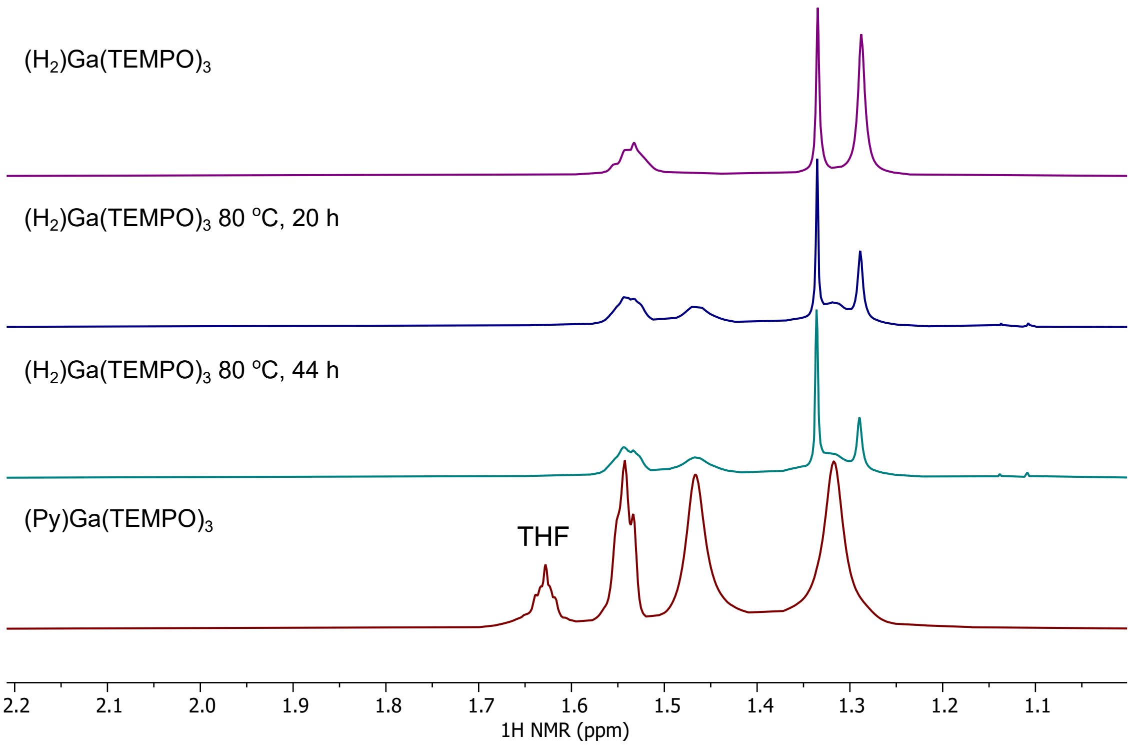

Figure S.41: Stacked ^{1}H NMR (pyridine-d_5, 25 °C) spectra of $(H_2)Ga(TEMPO)_3$, **4** after being heated to 80 °C, and **1**·Py (top to bottom).

S.1.10 Synthesis of $\{In(TEMPO)\}_2$ (5)

2·Py (0.300 g, 0.453 mmol, 1 equiv) was dissolved in *n*-hexane (15 mL) and the solution was transferred to a Schlenk storage flask (50 mL) containing a Teflon stirbar. The solution was removed from the glovebox and degassed by three freeze-pump-thaw cycles. The flask was then backfilled with H_2 (*ca.* 3 atm, 6 mmol, 10 equiv) and set to stir for 1 h, during which time pale yellow solids crashed out of solution. The flask was brought back into the glovebox where all volatile materials were removed under reduced pressure, providing pale yellow solids. The solids were suspended in diethyl ether (10 mL) and filtered through a pipette containing a plug of glass microfiber filter paper. The filtrate was placed in a −35 °C freezer for crystallization. **5** was isolated as a pale yellow, crystalline material (0.080 g, 0.295 mmol, 65% yield). When dissolved in pyridine-d_5 or benzene-d_6, the crystals formed a gray suspension that was filtered through glass microfiber filter paper prior to NMR data collection. ^{1}H NMR (400 MHz, benzene-d_6, 25 °C, Fig. S.42) δ 1.31, 1.35, 1.14 ppm. ^{13}C{^{1}H} NMR (101 MHz, benzene-d_6, 25 °C, Fig. S.43) δ 57.12, 40.05, 28.07, 18.11 ppm. Elem. Anal. Calc'd(found) for $C_{18}H_{36}N_2O_2In_2$: C 39.88(38.98), H 6.69(6.62), N 5.17(5.01).

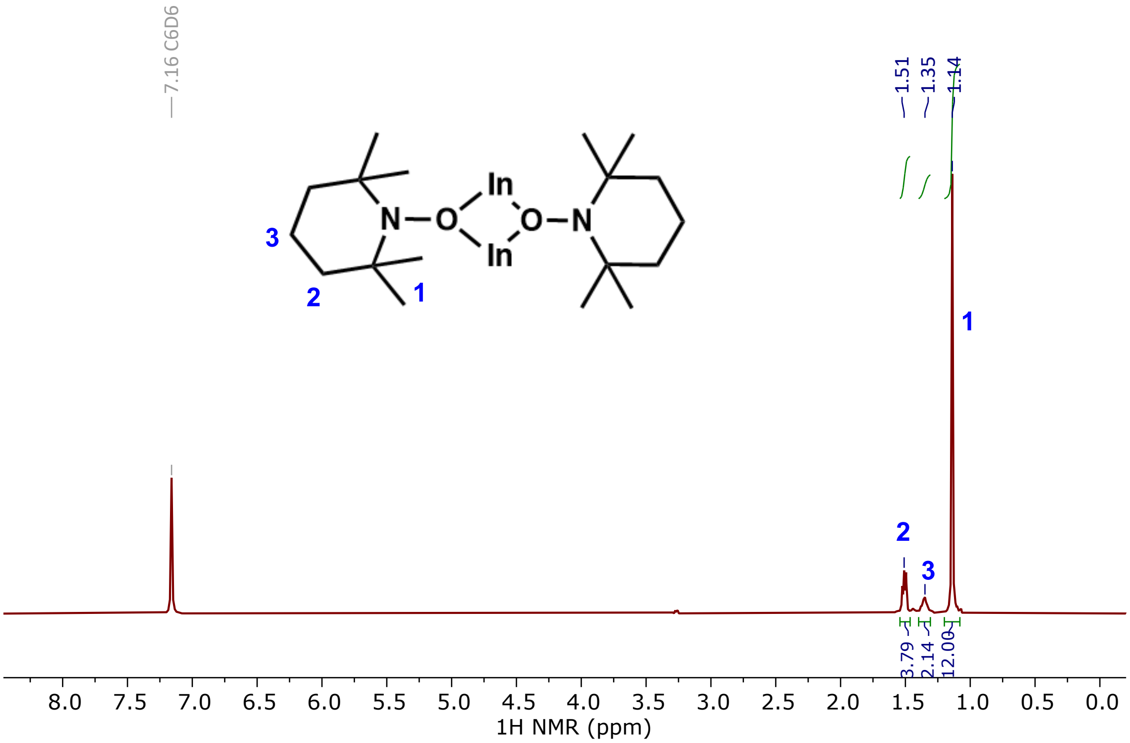

Figure S.42: ^{1}H NMR (400 MHz, benzene-d_6, 25 °C) spectrum of **5**.

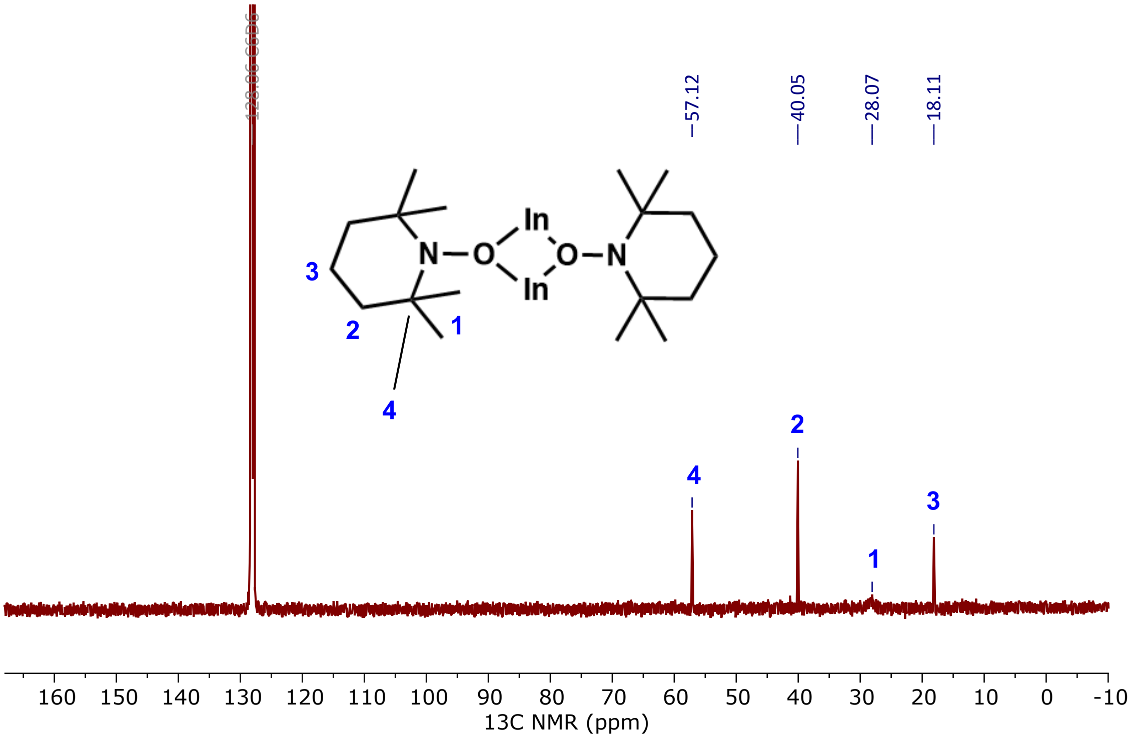

Figure S.43: ^{13}C{^{1}H} NMR (101 MHz, benzene-d_6, 25 °C) spectrum of **5**.

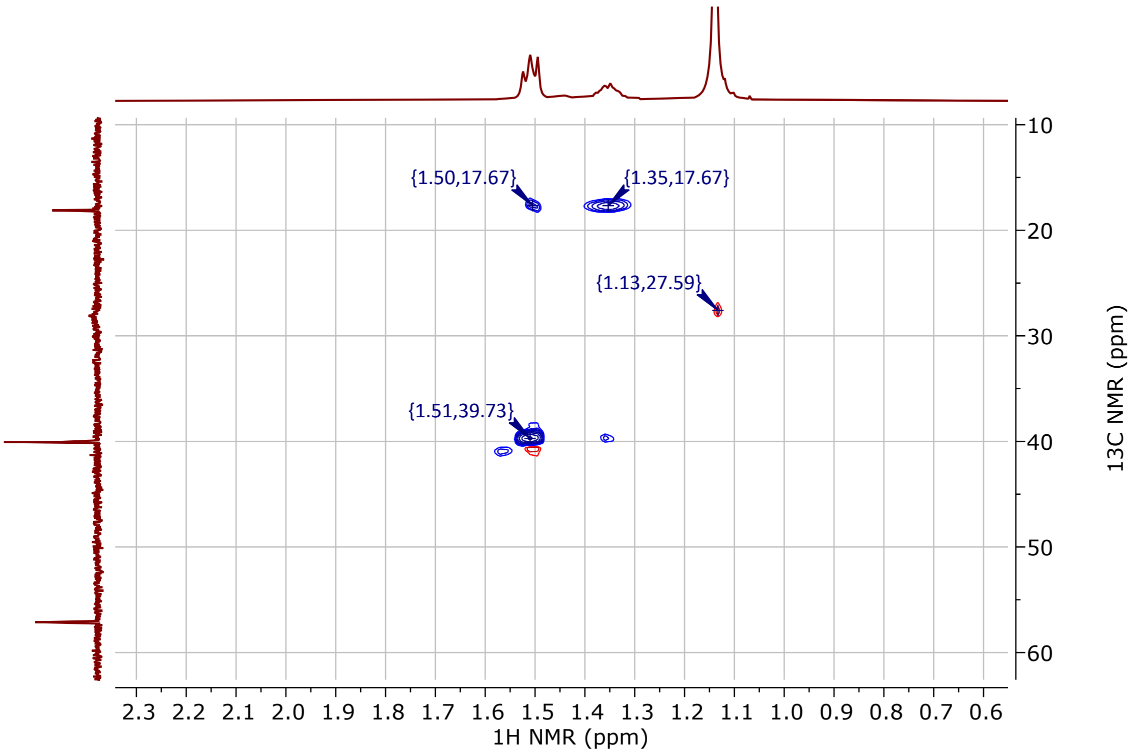

Figure S.44: Multiplicity-edited ^{1}H, ^{13}C-HSQC NMR (400 MHz, benzene-d_6, 25 °C) spectrum of **5**.

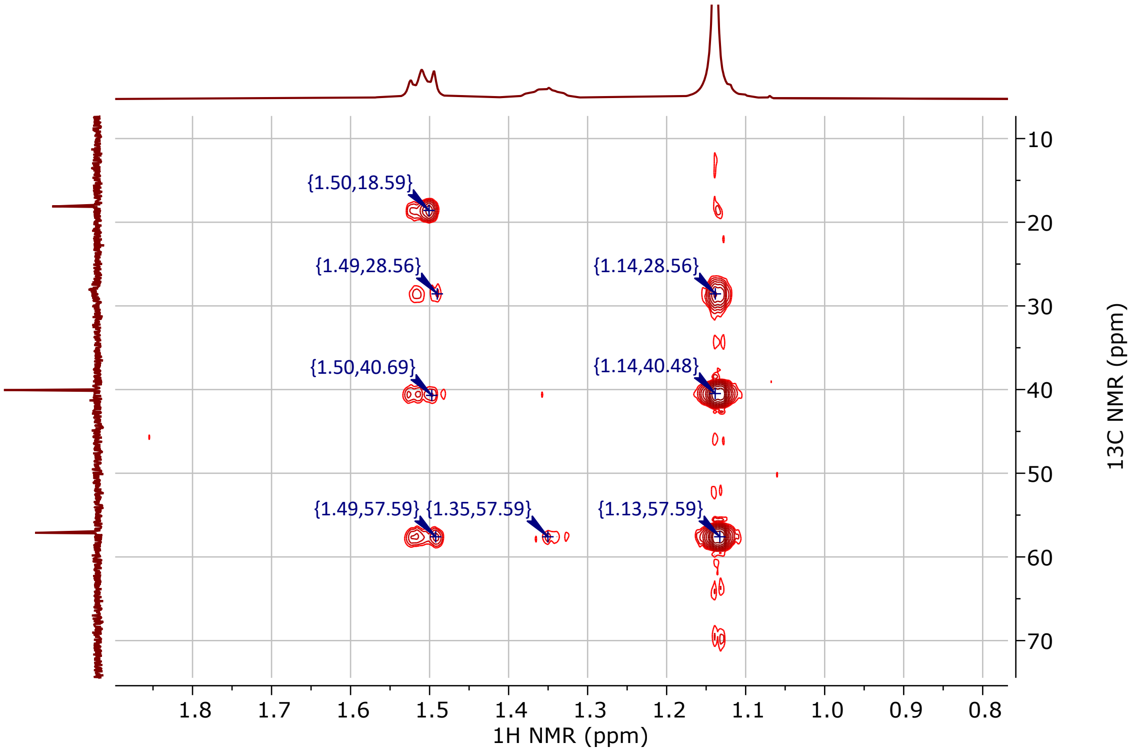

Figure S.45: ^{1}H, ^{13}C-HMBC NMR (400 MHz, benzene-d_6, 25 °C) spectrum of **5**.

S.1.10.1 Treatment of **2**·Py with D_2

2·Py (0.005 g, 0.008 mmol, 1 equiv) was dissolved in benzene-d_6 (0.5 mL) and the solution was transferred to a J Young tube (2 mL). The solution was removed from the glovebox and degassed by three freeze-pump-thaw cycles. The tube was backfilled with D_2 (*ca.* 2 atm, 0.2 mmol, 20 equiv) and after 10 min, ^{1}H NMR and ^{2}H NMR spectra were collected after approximately 10 min (Fig. S.46 and Fig. S.47).

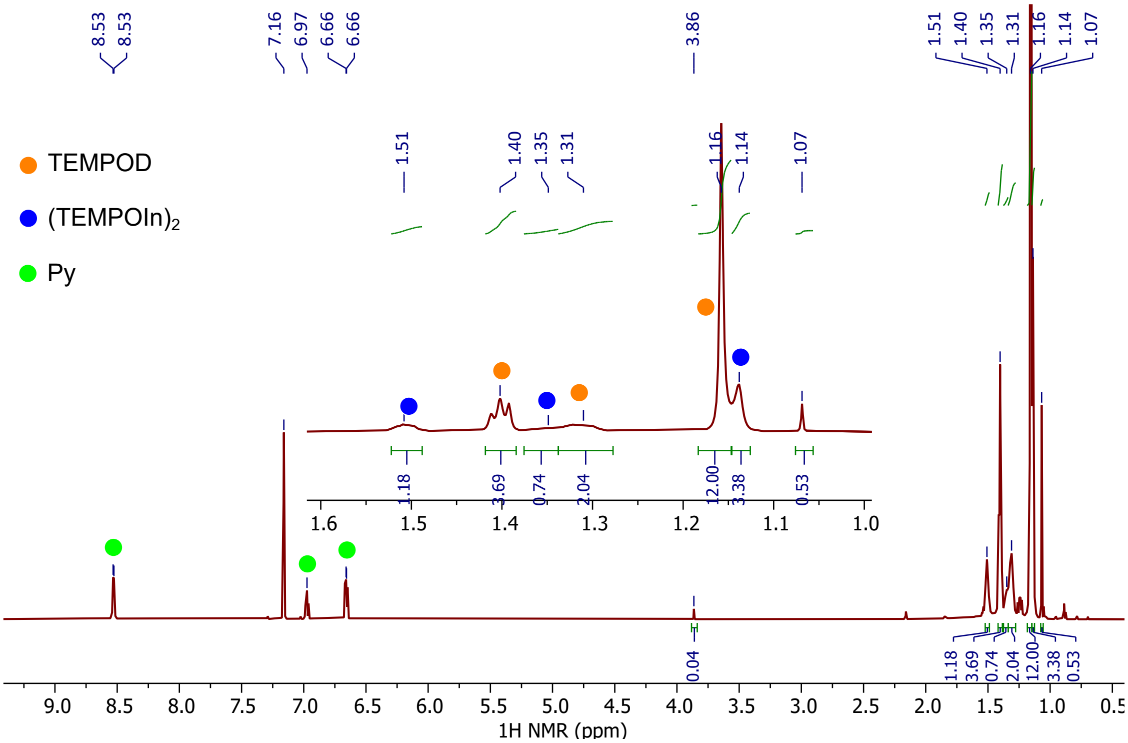

Figure S.46: ^{1}H NMR (600 MHz, benzene-d_6, 25 °C) spectrum of **2**·Py treated with D_2.

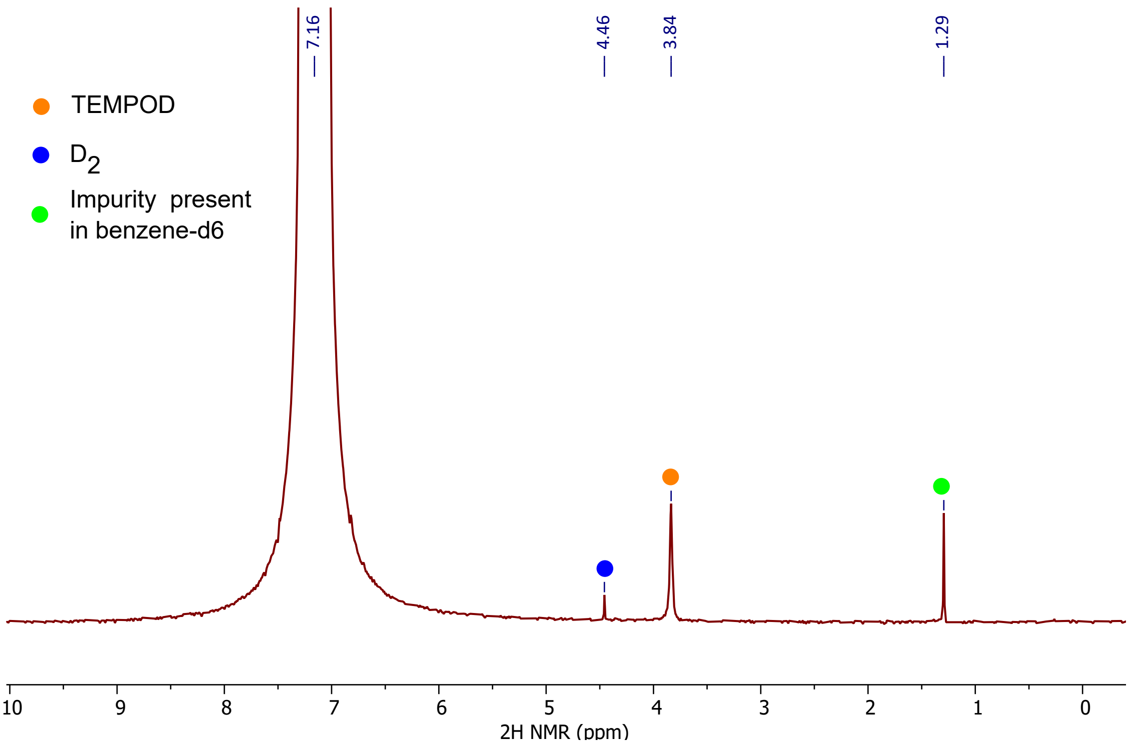

Figure S.47: ^{2}H NMR (353 MHz, benzene-d_6, 25 °C) spectrum of **2**·Py treated with D_2. The resonance at 1.29 ppm is present in benzene-d_6 received from Cambridge Isotope Laboratories and possibly corresponds to cyclohexane-d_{12}.

The NMR sample was transferred into a liquid IR transmission cell in the glovebox. IR data was collected and the data is shown in Fig. S.48. A band at 2634 cm^{-1} was observed and correspond to the O−D stretch of TEMPOD. This value matches previously reported data for TEMPOD.[7] For reference, IR data of TEMPOH, generated by treating **2**·Py with H_2 in benzene-d_6, is included in Fig. S.48.

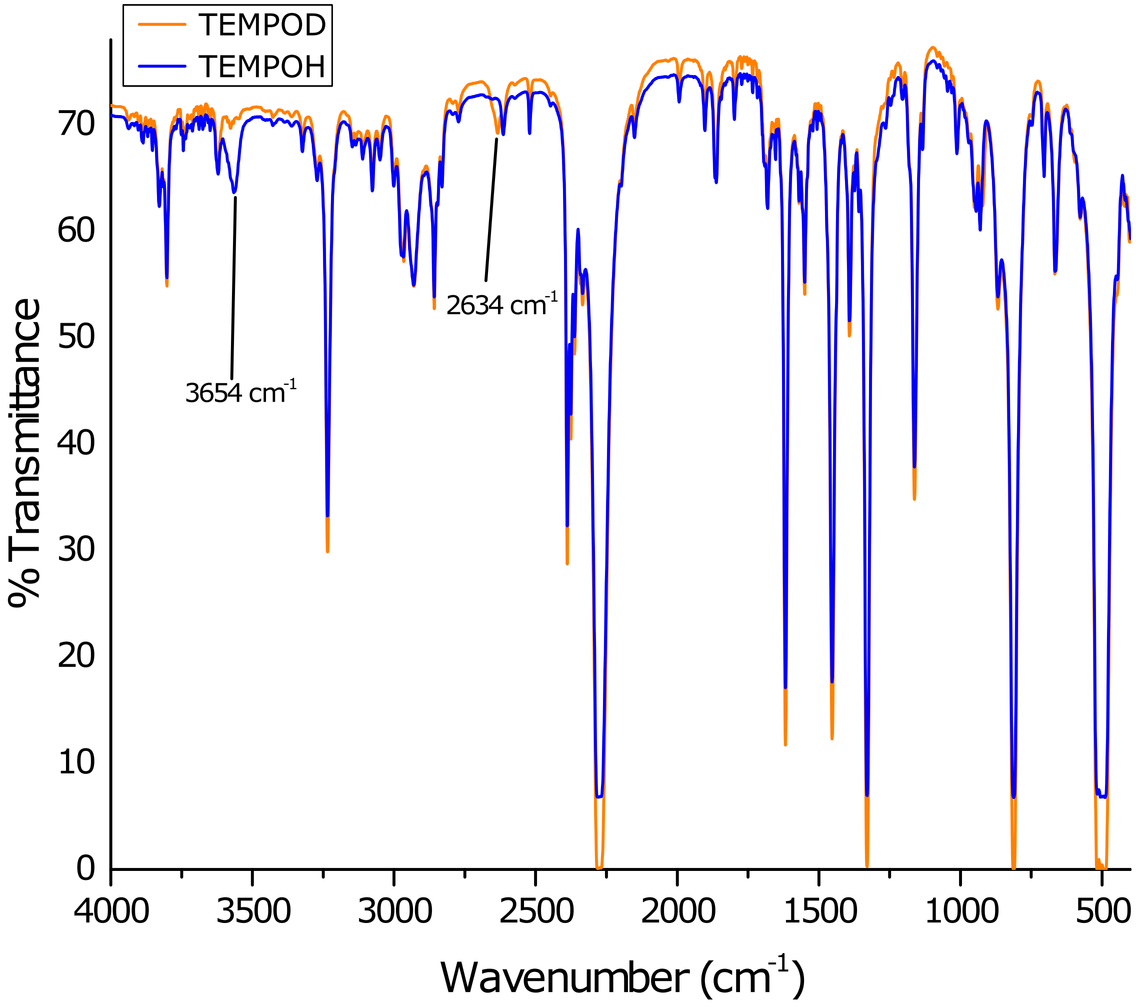

Figure S.48: Overlayed IR spectra of TEMPOH and TEMPOD generated from treating **2**·Py with either H_2 (blue) or D_2 (orange) in benzene-d_6.

S.1.10.2 Quantification of Conversion using an Internal Standard

Prior to running this experiment, spin-lattice decay constants (T_1) were measured using an inversion recovery pulse sequence and T_1 times of less than 4.3 s (6.73 ppm resonance for (Py)In(TEMPO)$_3$, Fig. S.49), 4.3 s (3.31 ppm resonance of 1,3,5-trimethoxybenzene, Fig. S.33), 2.2 s (1.40 ppm resonance of TEMPOH, Fig. S.50) and 1.2 s (1.51 ppm resonance of **5**, Fig. S.51) were measured. Therefore, all ^{1}H NMR spectra were recorded with a relaxation delay set to 30 s to obtain quantitative ^{1}H NMR data.

2·Py (0.010 g, 0.015 mmol, 1.0 equiv) and 1,3,5-trimethoxybenzene (0.0025 g, 0.015 mmol, 1.0 equiv) were dissolved in benzene-d_6 (0.5 mL) and the solution was transferred to a J Young tube (2 mL). An initial quantitative ^{1}H NMR spectrum of the mixture was collected and is shown in Fig. S.52. The solution was degassed by three freeze-pump-thaw cycles and backfilled with H_2 (*ca.* 1 atm) and after 10 min, quantitative ^{1}H NMR data were collected (Fig. S.53). The ^{1}H NMR data indicate 68% and 25% conversion of **2**·Py to TEMPOH and **5**, respectively.

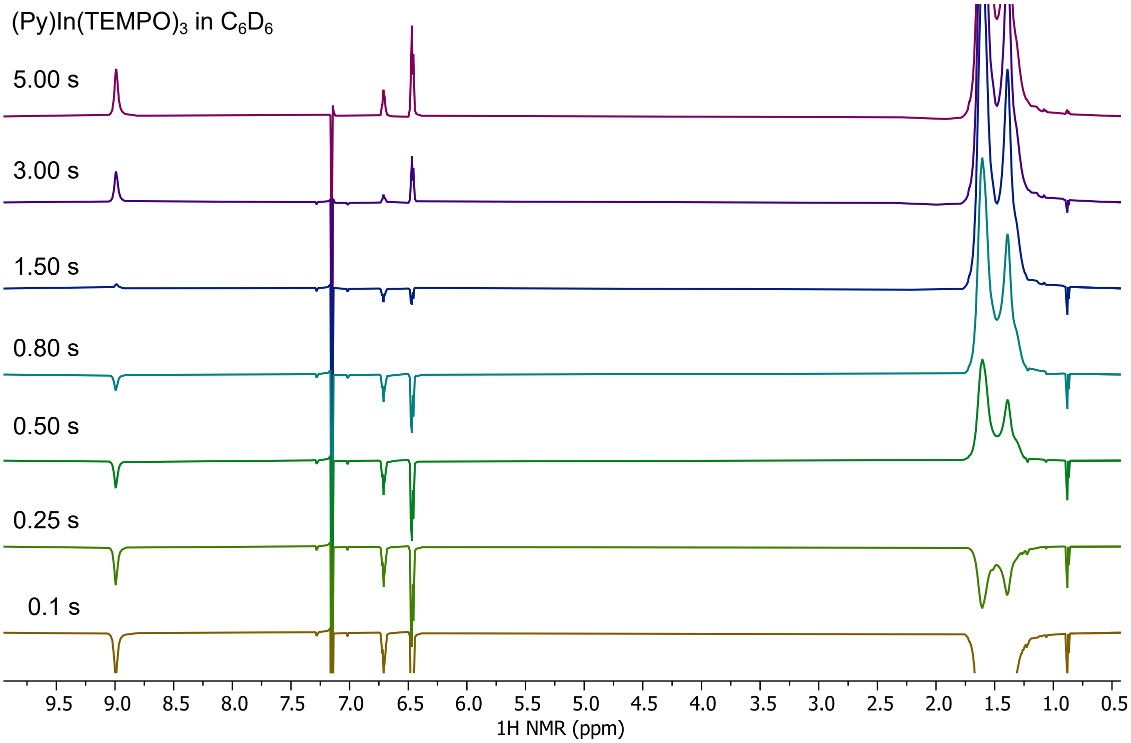

Figure S.49: Stacked ^{1}H NMR spectra of **2**·Py and associated relaxation delay times (D1) from an inversion recovery experiment.

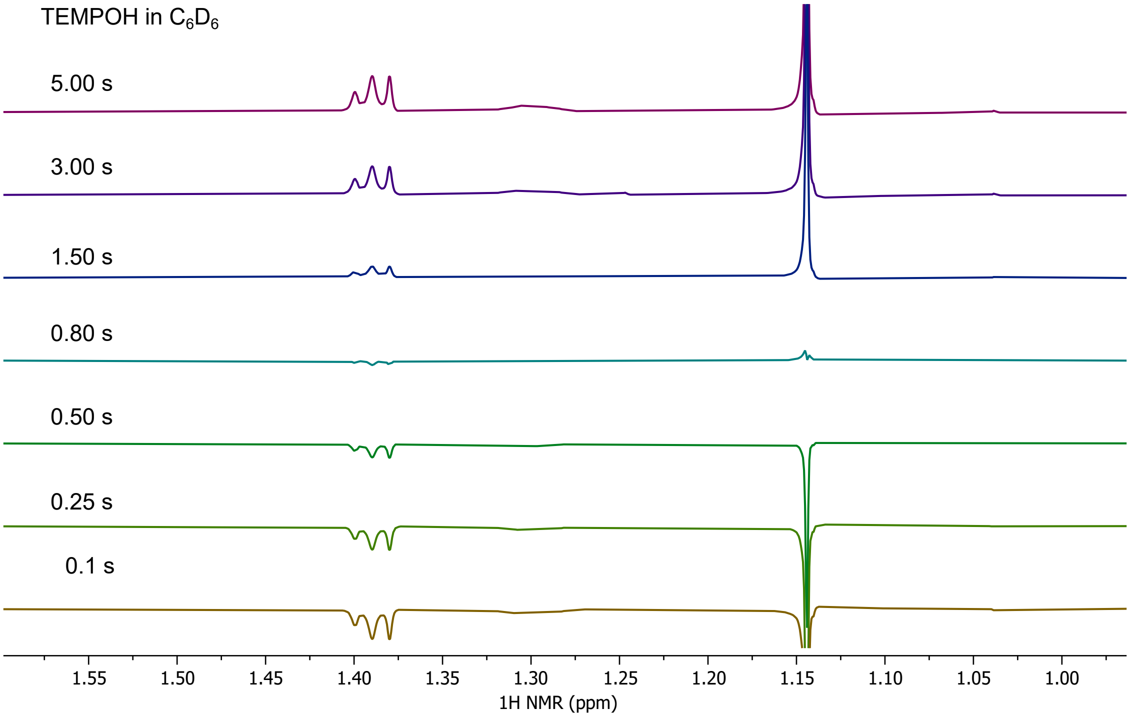

Figure S.50: Stacked ^{1}H NMR spectra of TEMPOH in benzene-d_6 and associated relaxation delay times (D1) from an inversion recovery experiment.

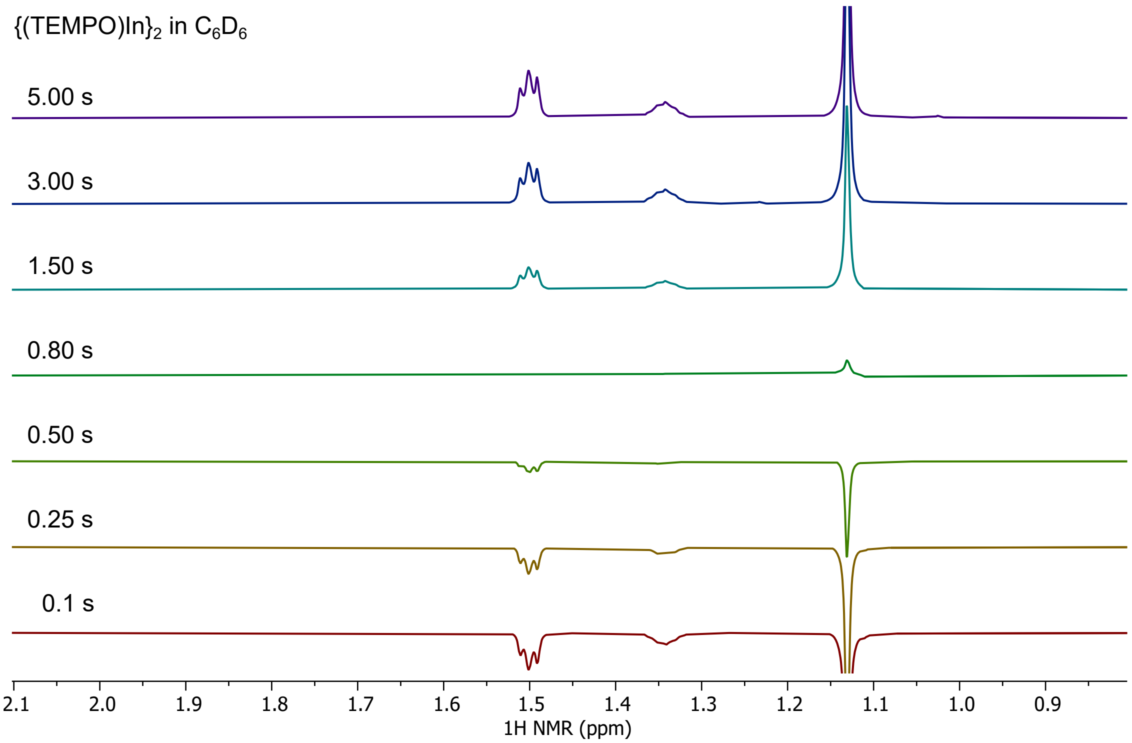

Figure S.51: Stacked ^{1}H NMR spectra of **5** in benzene-d_6 and associated relaxation delay times (D1) from an inversion recovery experiment.

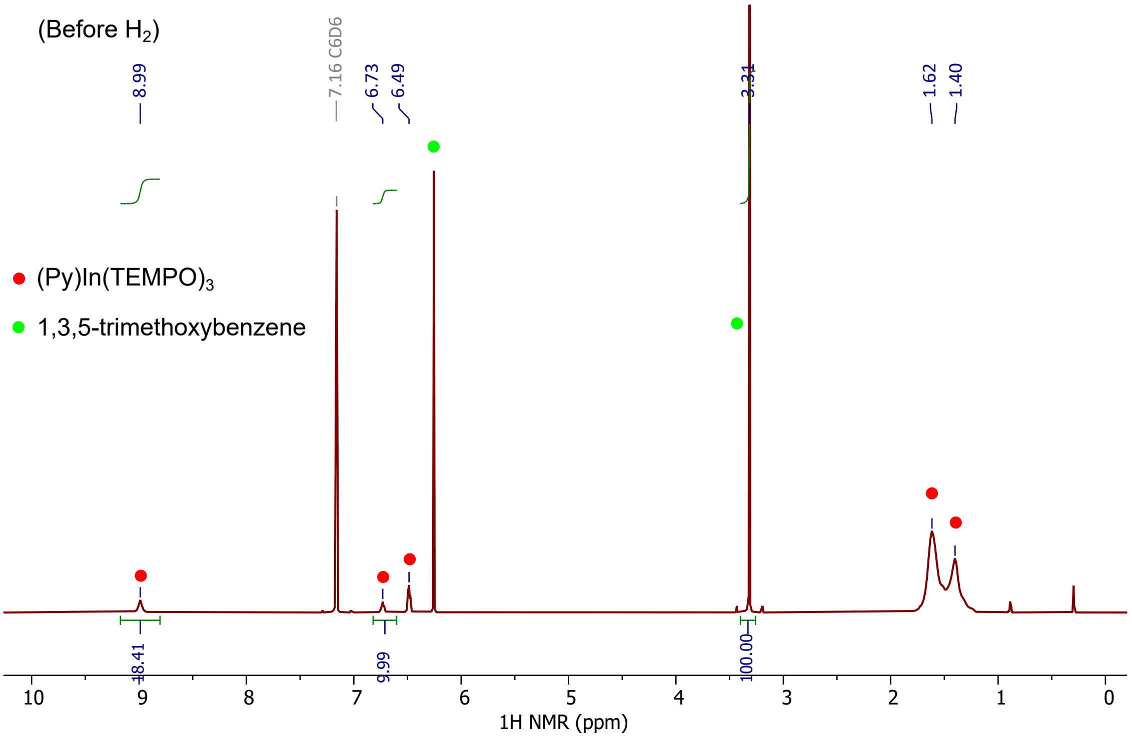

Figure S.52: Quantitative ^{1}H NMR (600 MHz, benzene-d_6, 25 °C) spectrum of **2**·Py and 1,3,5-trimethoxybenzene.

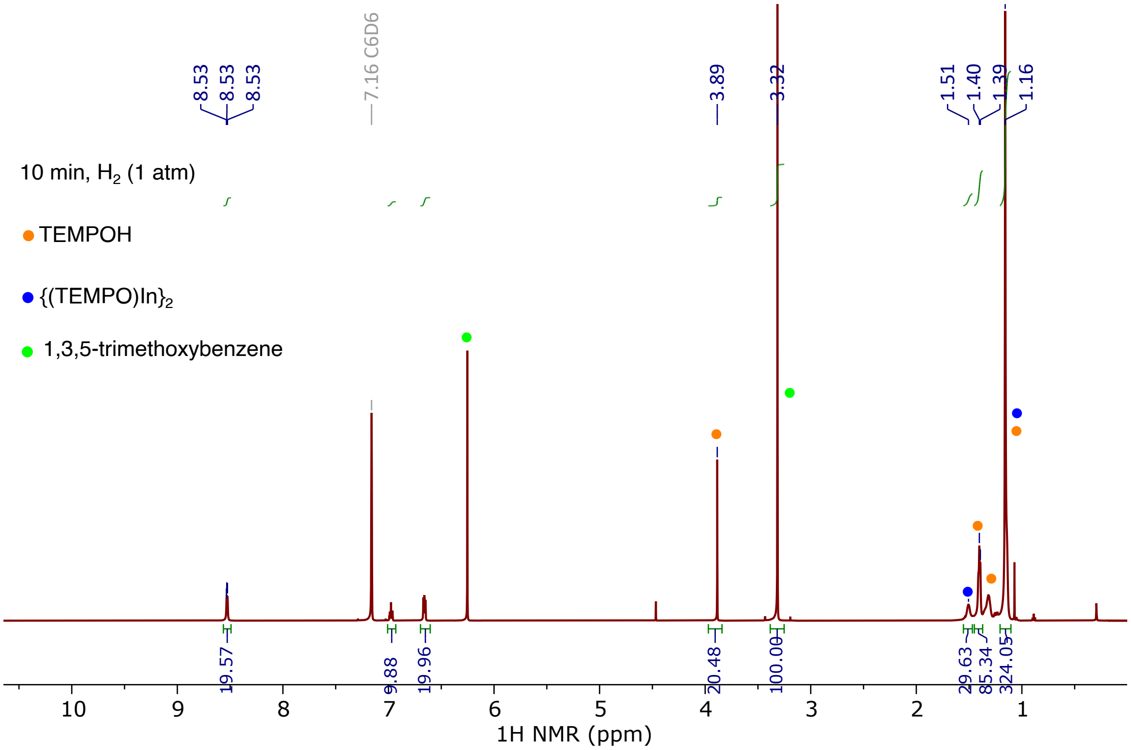

Figure S.53: Quantitative ^{1}H NMR (600 MHz, benzene-d_6, 25 °C) spectrum of **2**·Py and 1,3,5-trimethoxybenzene 10 min after the addition of H_2 (*ca.* 1 atm).

S.1.10.3 Generation of Indium Metal from **2**·Py

2·Py (0.200 g, 0.302 mmol, 1 equiv) was dissolved in *n*-hexane (12 mL) and the solution was transferred to a Schlenk storage flask (50 mL) containing a Teflon stirbar. The solution was removed from the glovebox and degassed by three freeze-pump-thaw cycles. The flask was then backfilled with H_2 (*ca.* 3 atm, 6 mmol, 20 equiv) and set to stir for 20 h. The flask containing a gray-brown suspension was brought into the glovebox where all volatile materials were removed under reduced pressure. The solids were suspended in diethyl ether (6 mL) and filtered through a pipette containing a plug of glass microfiber filter paper. The gray-brown solids were washed with diethyl ether (1 mL) and dried under reduced pressure. The solids (19 mg) were transferred to a zero diffraction plate and covered using Kapton tape to create an air-tight seal. PXRD data were collected using a CuK_α X-ray beam (λ = 1.5418 Å) in the 20–70° 2θ range with a step size of 0.017°, time per step of 60 s, and a 0.5° slit. The powder pattern is depicted in Fig. S.54 and are consistent with data reported for indium metal.[8]

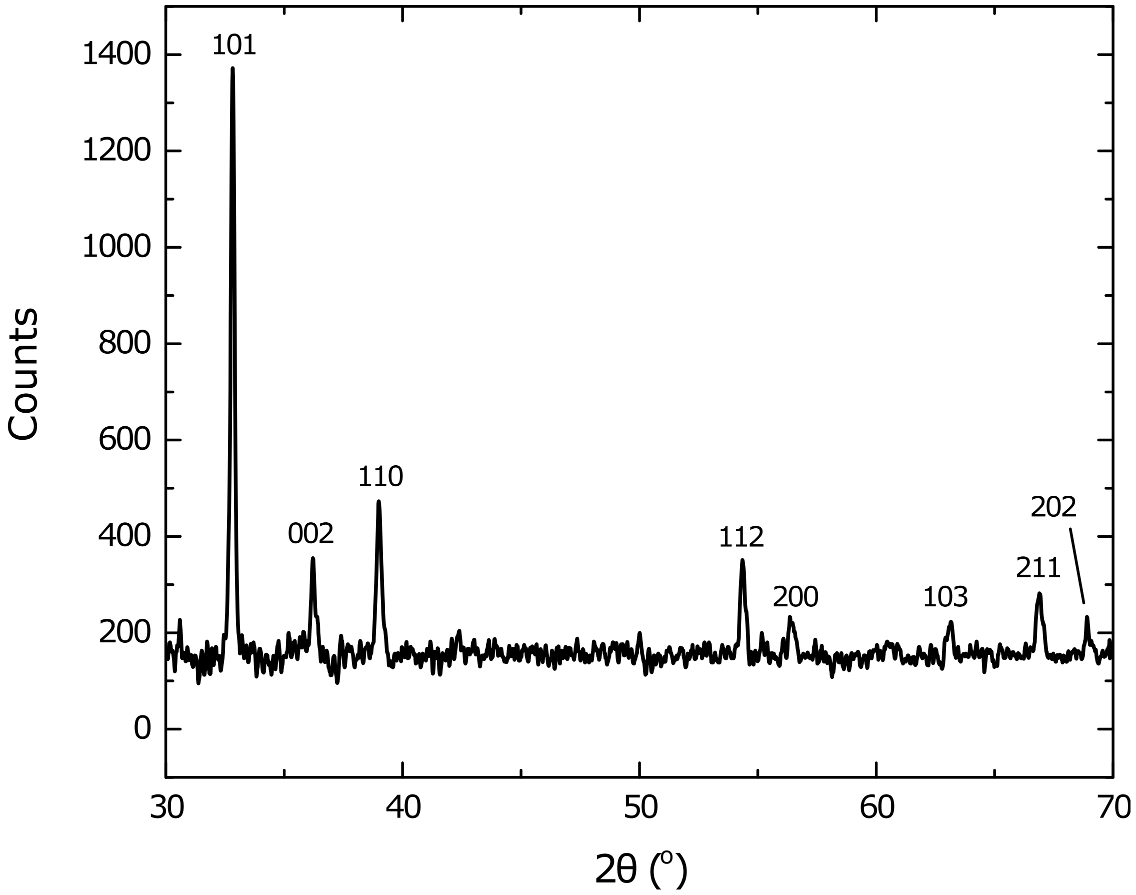

Figure S.54: PXRD pattern of generated indium metal (0.5° slit, 0.017° stepsize, 60 s/step) and assigned reflections.

S.1.10.4 Treatment of 2·Py with H_2 in pyridine-d_5

2·Py (0.005 g, 0.008 mmol, 1 equiv) was dissolved in pyridine-d_5 (0.5 mL) and the solution was transferred to a J Young tube (2 mL). The solution was removed from the glovebox and degassed by three freeze-pump-thaw cycles. The tube was backfilled with H_2 (*ca.* 2 atm, 0.2 mmol, 20 equiv) and ^{1}H NMR data was collected after 10 min and 4 h (Fig. S.55 and S.56, respectively). For reference, a ^{1}H NMR spectrum of **5** is shown in Fig. S.57. TEMPOH and **5** are generated in approximately a 2:1 ratio after 10 min. After 4 h, this ratio changes to approximately 12:1 and indium metal settled to the bottom of the J Young tube.

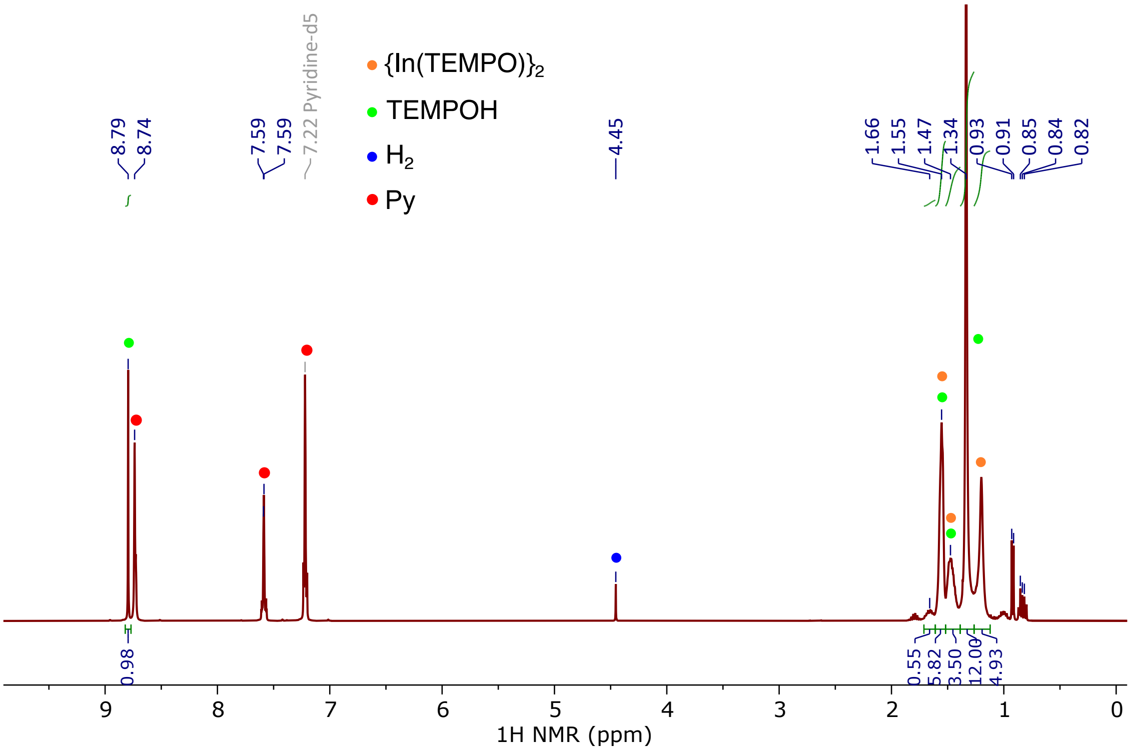

Figure S.55: ^{1}H NMR (400 MHz, pyridine-d_5, 25 °C) spectrum of **5** 10 min after the addition of H_2 (*ca.* 2 atm).

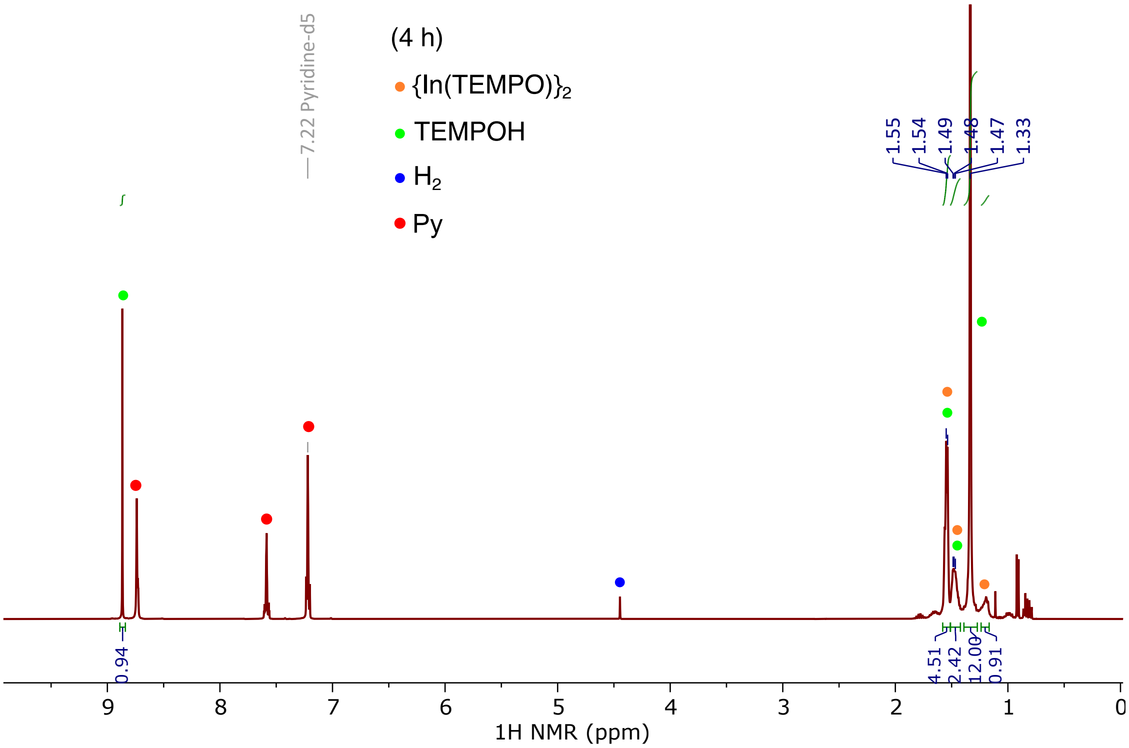

Figure S.56: ^{1}H NMR (400 MHz, pyridine-d_5, 25 °C) spectrum of **5** 10 min after the addition of H_2 (*ca.* 2 atm).

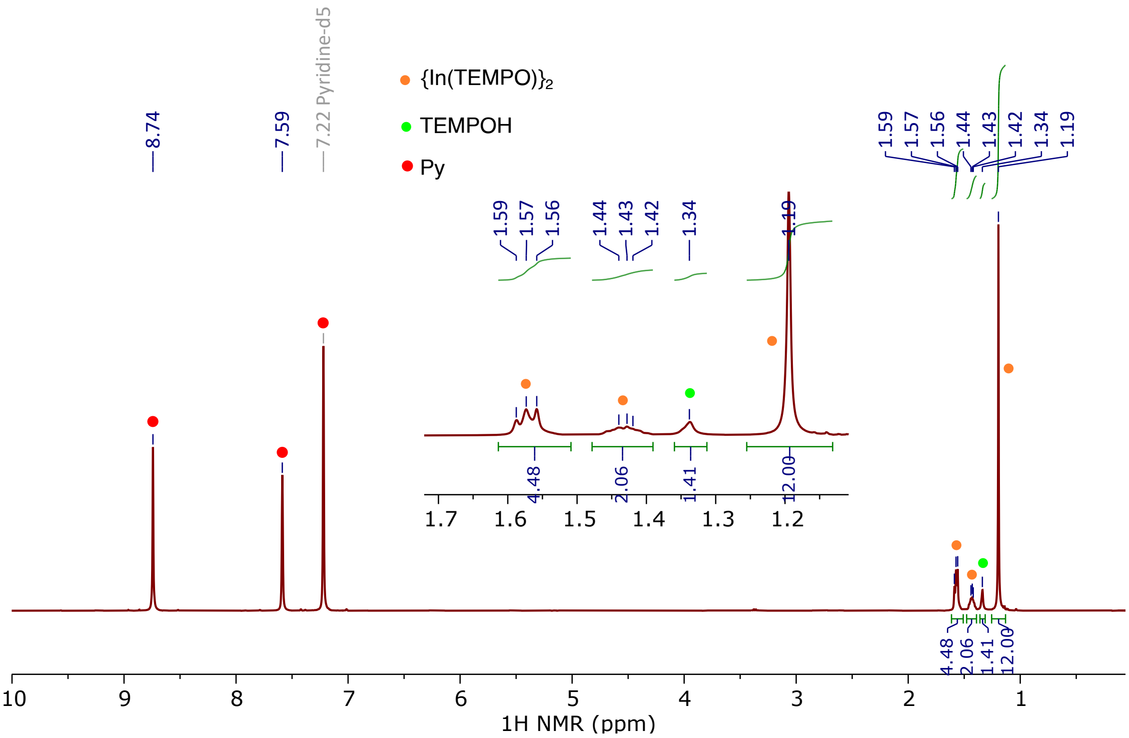

Figure S.57: ^{1}H NMR (400 MHz, pyridine-d_5, 25 °C) spectrum of **5**.

S.1.11 Treatment of 5 with H_2

Pale yellow crystals of **5** (0.005 g, 0.009 mmol) were added to benzene-d_6, resulting in a gray suspension. The suspension was filtered through glass microfiber filter paper into a J Young tube and the filtrate was degassed by three freeze-pump-thaw cycles. The tube was backfilled with H_2 (*ca.* 3 atm) and monitored over 20 h by ^1H NMR spectroscopy, during which time the initially homogeneous solution became faintly cloudy (Fig. S.58 and S.59). After 20 min, ca. 30% conversion to TEMPOH was observed after 20 min; additionally, trace TEMPH was observed.

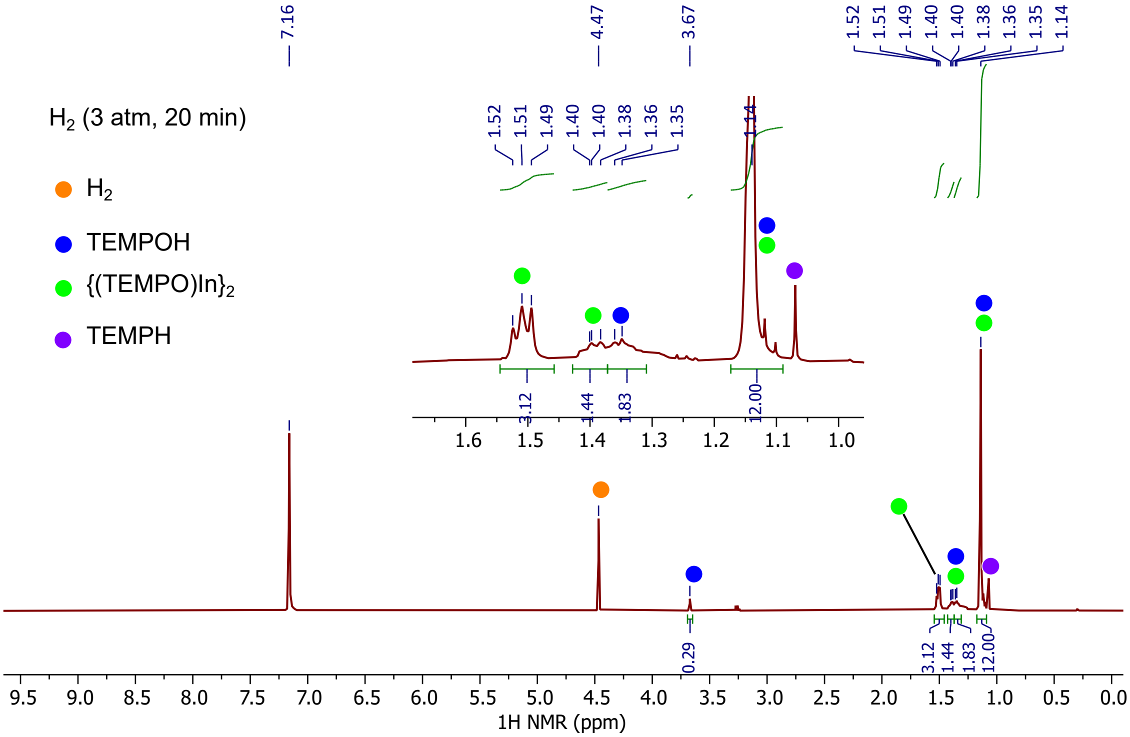

Figure S.58: ^1H NMR (400 MHz, benzene-d_6, 25 °C) spectrum of **5** after being treated with H_2 (*ca.* 3 atm) for 20 min.

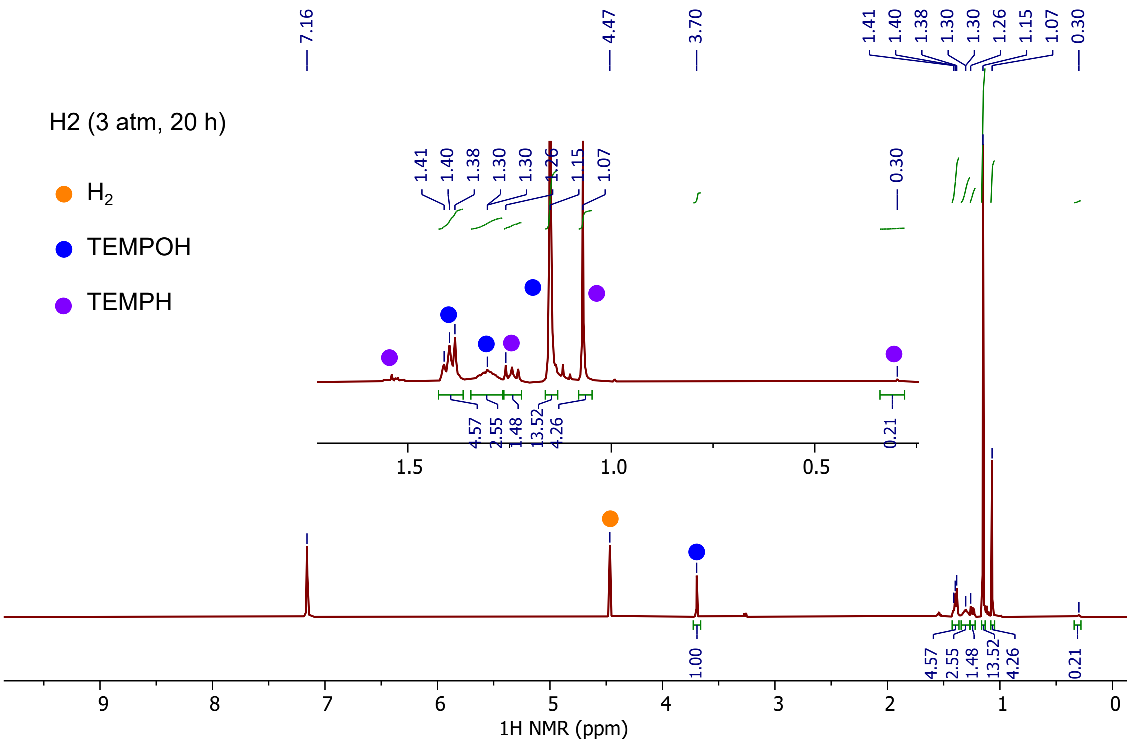

Figure S.59: ^{1}H NMR (400 MHz, benzene-d_6, 25 °C) spectrum of **5** after being treated with H_2 (*ca.* 3 atm) for 20 h.

S.1.12 Investigating the Thermal Stability of 5

Pale yellow crystals of **5** (0.005 g, 0.009 mmol) were added to benzene-d_6, resulting in a gray suspension. The suspension was filtered through glass microfiber filter paper into a J Young tube and the filtrate was heated to 80 °C and monitored by ^{1}H NMR over 3 h (Fig. S.60 and S.61). Partial conversion of **5** to TEMPH (or tetramethylpiperidide) and an unidentified species was observed after 15 min. After heating the sample for 3 h, complete consumption of **5** was observed.

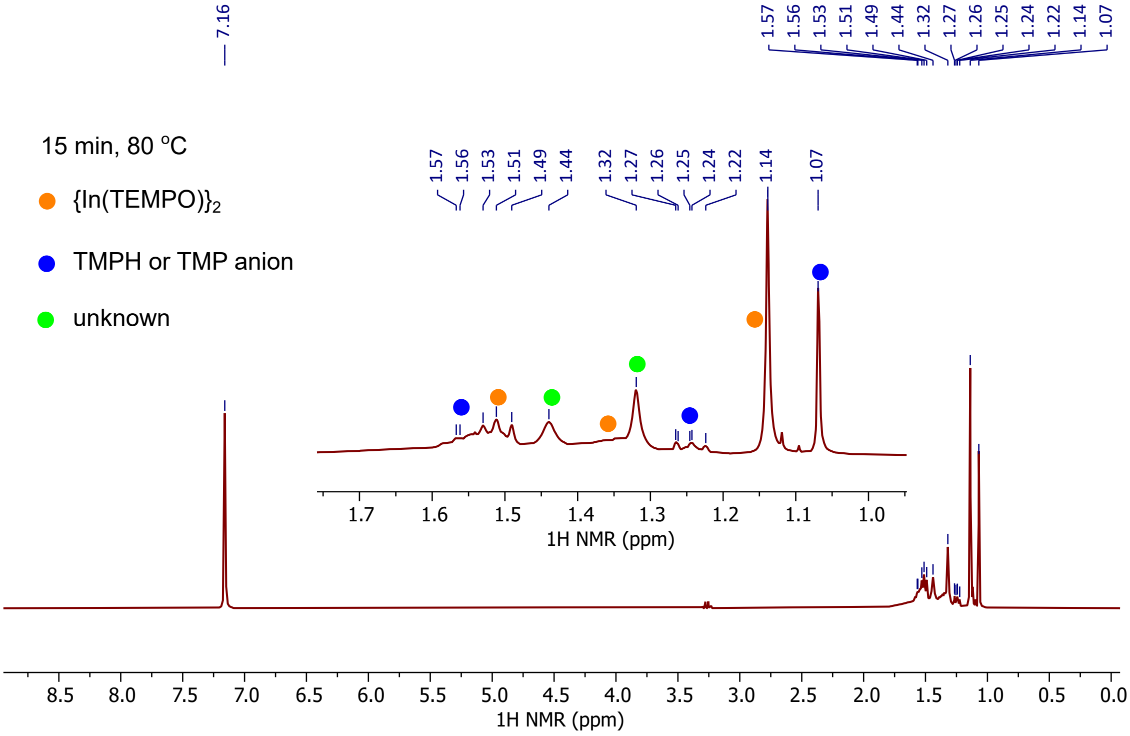

Figure S.60: ^{1}H NMR (300 MHz, benzene-d_6, 25 °C) spectrum of **5** after being heated to 80 °C for 15 min.

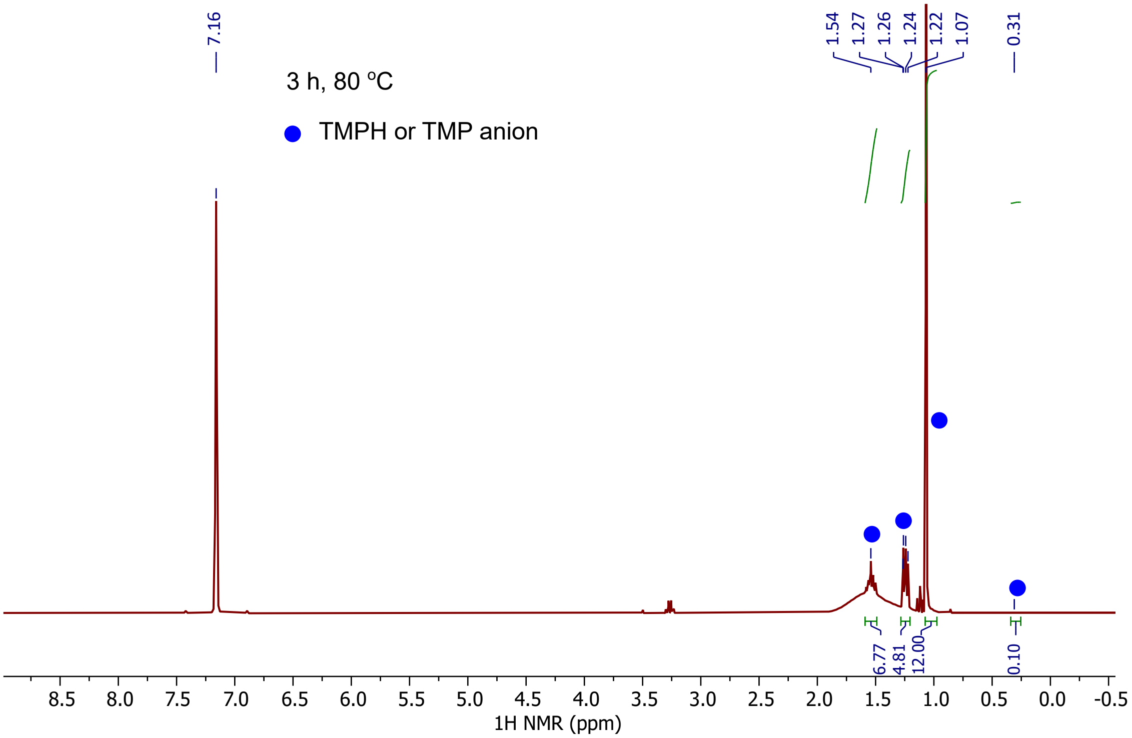

Figure S.61: ^{1}H NMR (300 MHz, benzene-d_6, 25 °C) spectrum of **5** after being heated to 80 °C for 15 min.

S.1.13 Treatment of InCl with NaTEMPO

To a suspension of InCl (0.084 g, 0.56 mmol, 1.0 equiv) in diethyl ether (2 mL) was added NaTEMPO (0.100 g, 0.556 mmol, 1.0 equiv) in diethyl ether (2 mL) dropwise with stirring. After the mixture stirred for 5 h, an aliquot was filtered through a pipette containing a plug of glass microfiber filter paper, and all volatile materials were removed from the filtrate under reduced pressure. The resulting pale yellow residue was dissolved in benzene-d_6 for ^{1}H NMR analysis, which revealed only unreacted NaTEMPO (Fig. S.62).

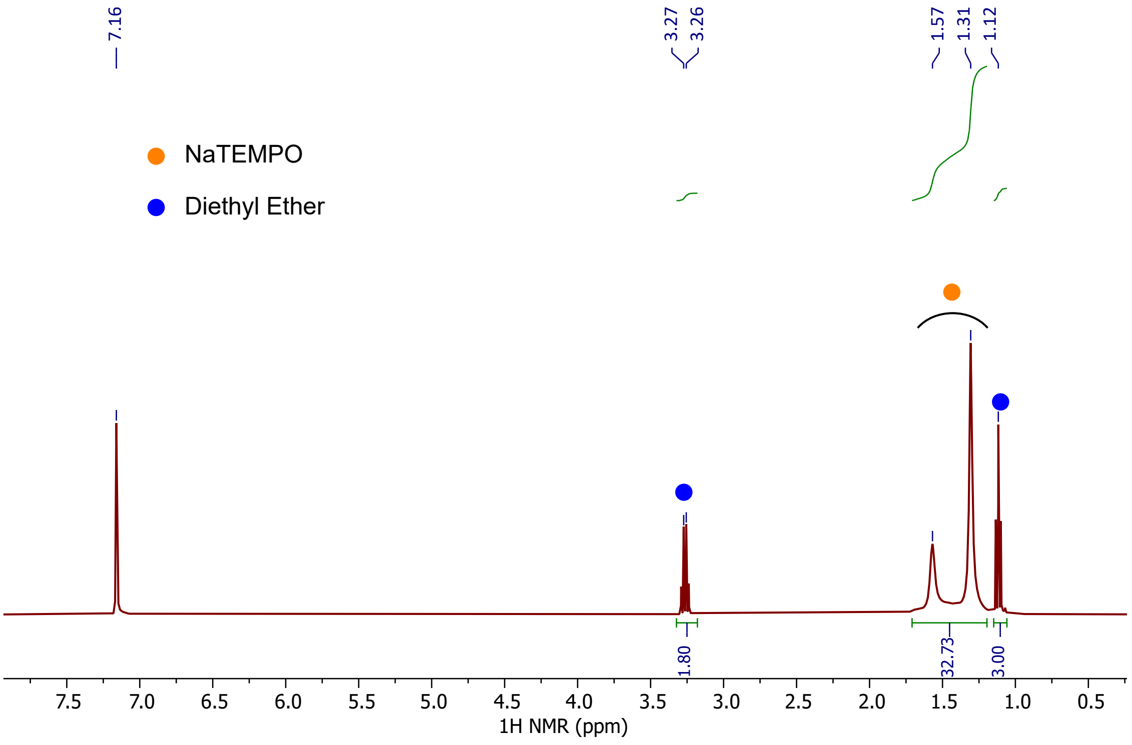

Figure S.62: ^{1}H NMR (400 MHz, benzene-d_6, 25 °C) spectrum of the crude reaction mixture obtain from combining InCl and NaTEMPO in diethyl ether.

S.1.14 Synthesis of InSe via the Treatment of 2·Py with Ph_3PSe and H_2

2·Py (0.300 g, 0.453 mmol, 1 equiv) and Ph_3PSe (0.232 g, 0.453 mmol, 1.50 equiv) were dissolved in benzene (10 mL), and the solution was transferred to a Schlenk storage flask (50 mL) containing a Teflon stirbar. The solution was removed from the glovebox and degassed by three freeze-pump-thaw cycles. The flask was then backfilled with H_2 (*ca.* 3 atm, 6 mmol, 10 equiv) and set to stir for 48 h, during which time pale orange solids crashed out of solution. The flask was brought into the glovebox where the orange solids were collected on a medium sintered frit via vacuum filtration. The material was washed with diethyl ether (3 x 10 mL) and dried under reduced pressure, yielding InSe as a free following orange powder (0.082 g, 0.042 mmol, 94%).

S.1.14.1 Elemental Analysis of InSe

Low carbon, hydrogen, and nitrogen content (11.48%, 1.64%, and 2.07%, respectively) was found for the obtained material consistent with the formation of binary InSe with some organic contamination.

S.1.14.2 ICP-MS Analysis of InSe

The ratio of In to Se was determined by inductively coupled plasma–mass spectroscopy (ICP-MS) performed on a PerkinElmer NexION 2000 ICP-MS. Consistent with an approximate 1:1 ratio of In to Se, concentrations of 1060±17 μg/mg and 976±8 μg/mg were found for In and Se, respectively. The analysis was performed in triplicated measurements with a background correction.

S.1.14.3 PXRD Analysis of InSe

PXRD data of InSe were collected using a CuK_α X-ray beam (λ = 1.5418 Å) in the 20–70° 2θ range with a step size of 0.017°, time per step of 60 s, and a 0.5° slit. However, only background signal from the PXRD sample holder was observed (Fig. S.63), suggesting that the material is amorphous. Notably, no reflections assignable to In metal or In_2O_3 were observed.

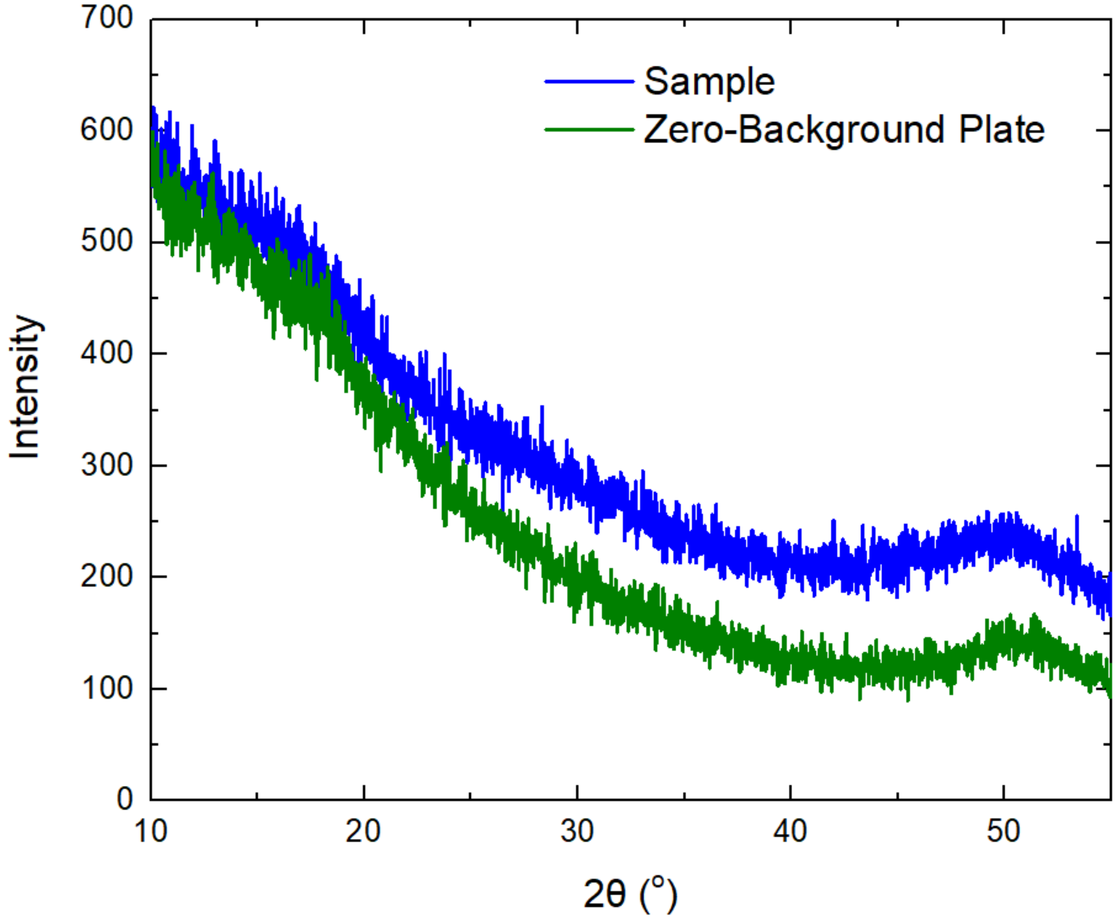

Figure S.63: PXRD data of generated InSe (0.5° slit, 0.017° stepsize, 60 s/step).

S.1.14.4 Diffuse-Reflectance UV-Vis Data of InSe

InSe, diluted by polytetrafluoroethylene (PTFE) powder (10% by weight InSe), was analyzed by diffuse-reflectance UV-vis spectroscopy, and the spectrum is shown in Fig. S.64. The spectrum was used to construct a Tauc plot, revealing an indirect optical gap of 1.95 eV (Fig. S.65).

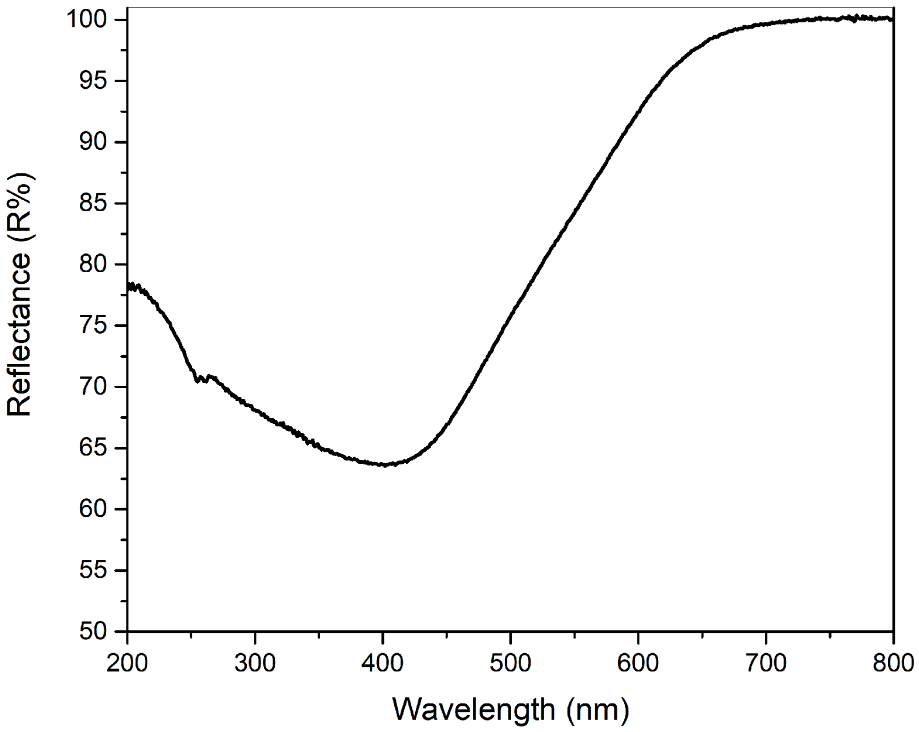

Figure S.64: Diffuse-reflectance UV-Vis spectrum of InSe diluted with PTFE powder.

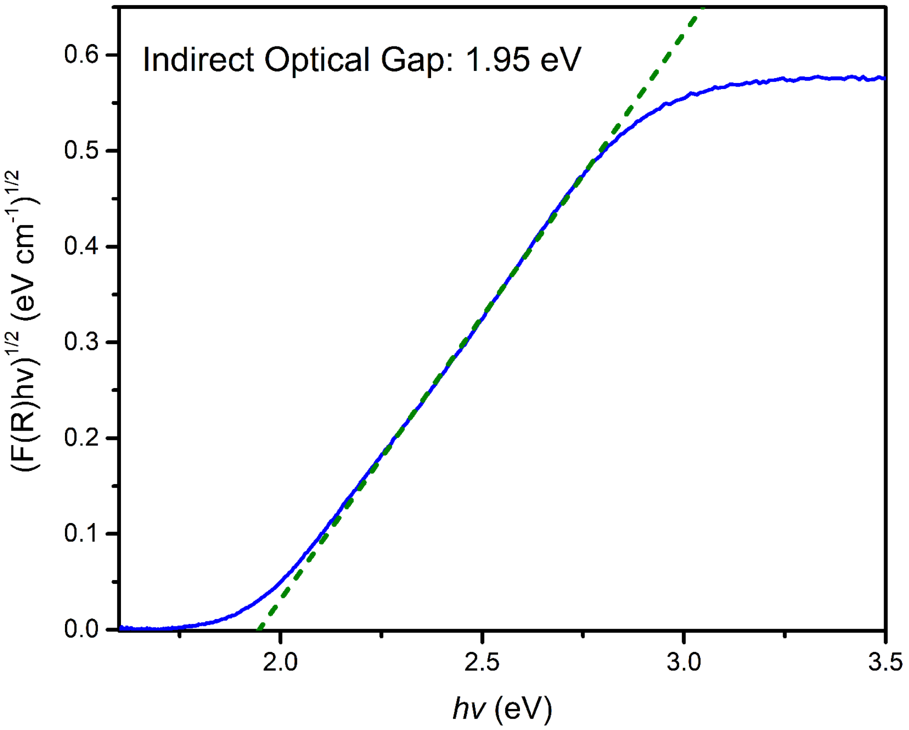

Figure S.65: Tauc plot constructed from the diffuse-reflectance spectrum of InSe, revealing an indirect optical gap of 1.95 eV.

S.1.15 SEM-EDX Analysis of InSe

Scanning electron microscopy (SEM) and energy-disperive X-ray (EDX) spectroscopy were collected on a SEM Nova 230. InSe was loaded onto adehesive carbon tape and data were collected using an accelerating voltage of 10 kV. Five spots were surveryed by EDX, which indicated an approximate 1:1 ratio of In to Se (Table S.2). Elemental mapping suggests an even distribution of indium and selenium throughout the sample (Fig. S.1.15)

Table S.2: EDX Analysis of InSe

**Spot**	**Element**	**Atom %**[a]	**Atom % Error**	**Atomic Ratio**
**1**	In	14.73	±0.12	0.98
	Se	14.96	±0.06	1.00
**2**	In	19.18	±0.13	1.17
	Se	16.46	±0.06	1.00
**3**	In	12.19	±0.08	0.94
	Se	12.99	±0.05	1.00
**4**	In	4.25	±0.05	1.12
	Se	3.80	±0.02	1.00
**5**	In	18.75	±0.11	1.13
	Se	16.61	±0.05	1.00

[a] C, N, O, and Cl make up the remaining atom % and are likely present due to the carbon tape and/or organic contamination.

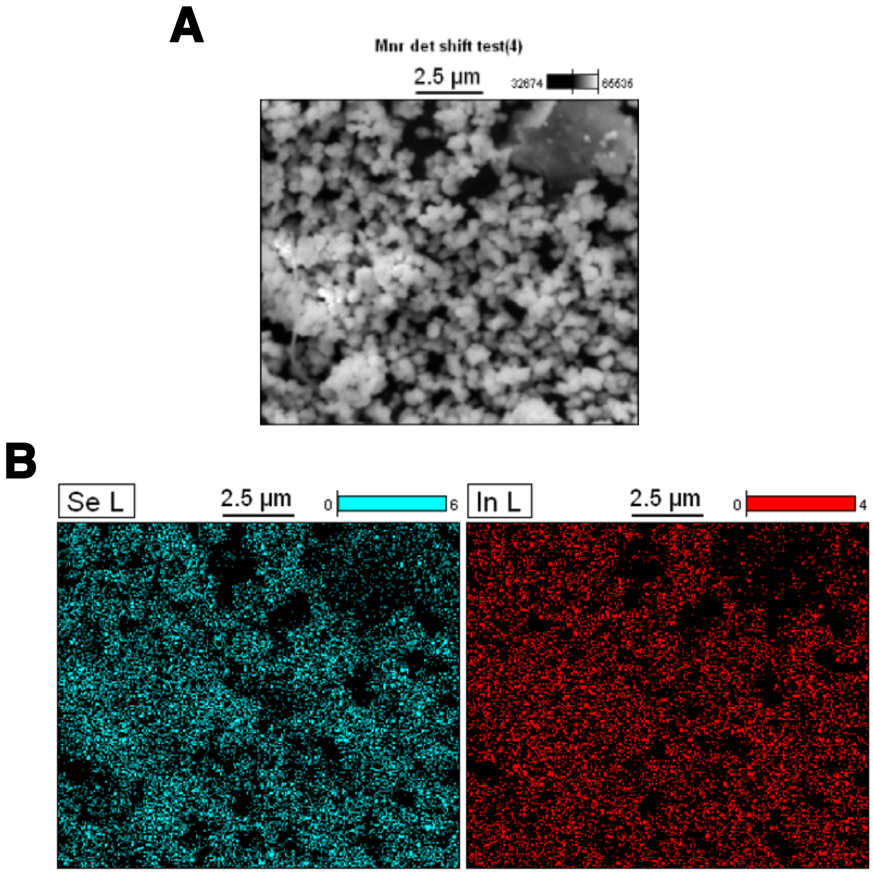

Figure S.66: A) SEM image of InSe. B) SEM image from A with overlay of regions containing Se (left) and In (right) as determined by EDX spectroscopy.

S.1.16 Monitoring the Treatment of **2**·Py with Ph_3PSe and H_2

2·Py (0.013 g, 0.020 mmol, 1.0 equiv) and Ph_3PSe (0.010 g, 0.029 mmol, 1.5 equiv) were dissolved in benzene-d_6 (0.6 mL) and the solution was transferred to a J Young tube. The sample was removed from the glovebox and degassed by three freeze-pump-thaw cycles. The tube was then backfilled with H_2 (*ca.* 3 atm, 0.25 mmol, 13 equiv). After several minutes, pale orange solids crashed out of solution (Fig. S.67), and NMR data was collected after 30 min. ^1H NMR analysis revealed free Ph_3P and pyridine, unreacted Ph_3PSe, and TEMPOH (Fig. S.68). Notably, resonances assigned to **5** were not observed. Additionally, $^{31}P\{^1H\}$ NMR analysis of the filtrate revealed a 1:2 ratio of Ph_3PSe to Ph_3P consistent with the generation of binary InSe (Fig. S.69).

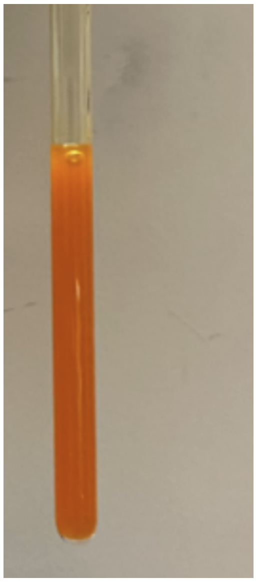

Figure S.67: Picture of the crude reaction mixture minutes after charging the tube with H_2.

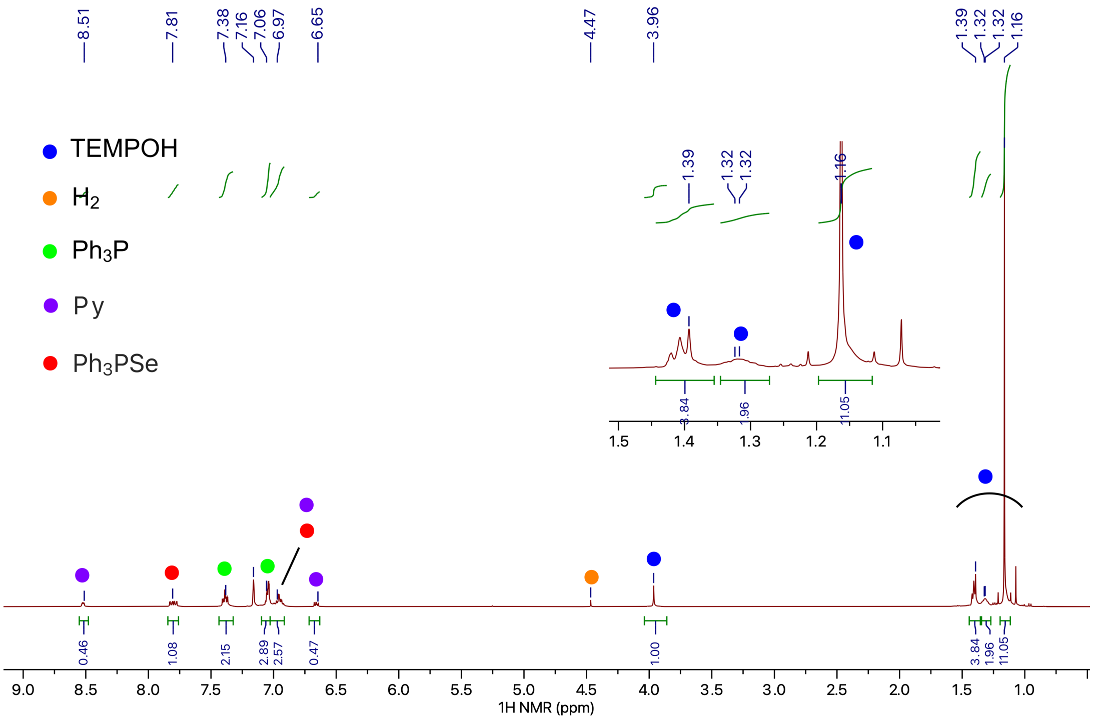

Figure S.68: ^{1}H NMR (400 MHz, benzene-d_6, 25 °C) spectrum of the crude reaction mixture 30 min after charging the J Young tube with H_2.

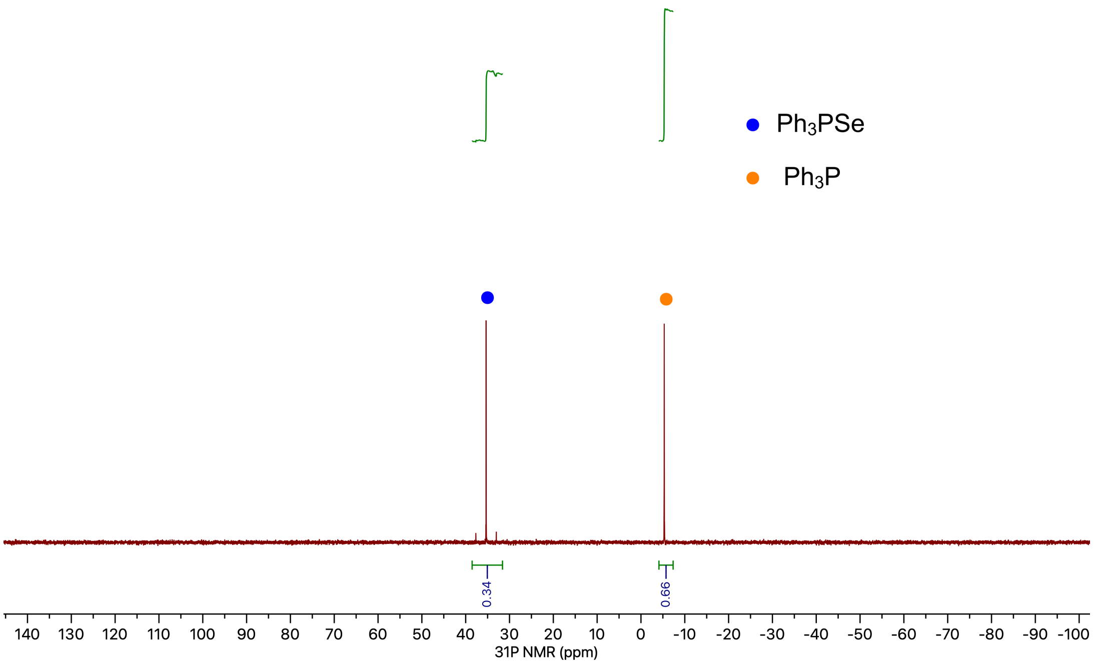

Figure S.69: ^{31}P{^{1}H} NMR (162 MHz, benzene-d_6, 25 °C) spectrum of the crude reaction mixture 30 min after charging the J Young tube with H_2.

S.1.17 Treatment of In Powder with Ph_3PSe

In the glovebox, indium powder (100-mesh; 0.003 g, 0.03 mmol, 1 equiv) was combined with Ph_3PSe (0.010 g, 0.030 mmol, 1 equiv) in benzene-d_6 (0.7 mL). After the suspension stirred for 48 h, the supernatant was transferred to an NMR tube for ^{31}P NMR analysis (Fig. S.70). Only a resonance assigned to Ph_3PSe was observed in the $^{31}P\{^1H\}$ NMR spectrum.

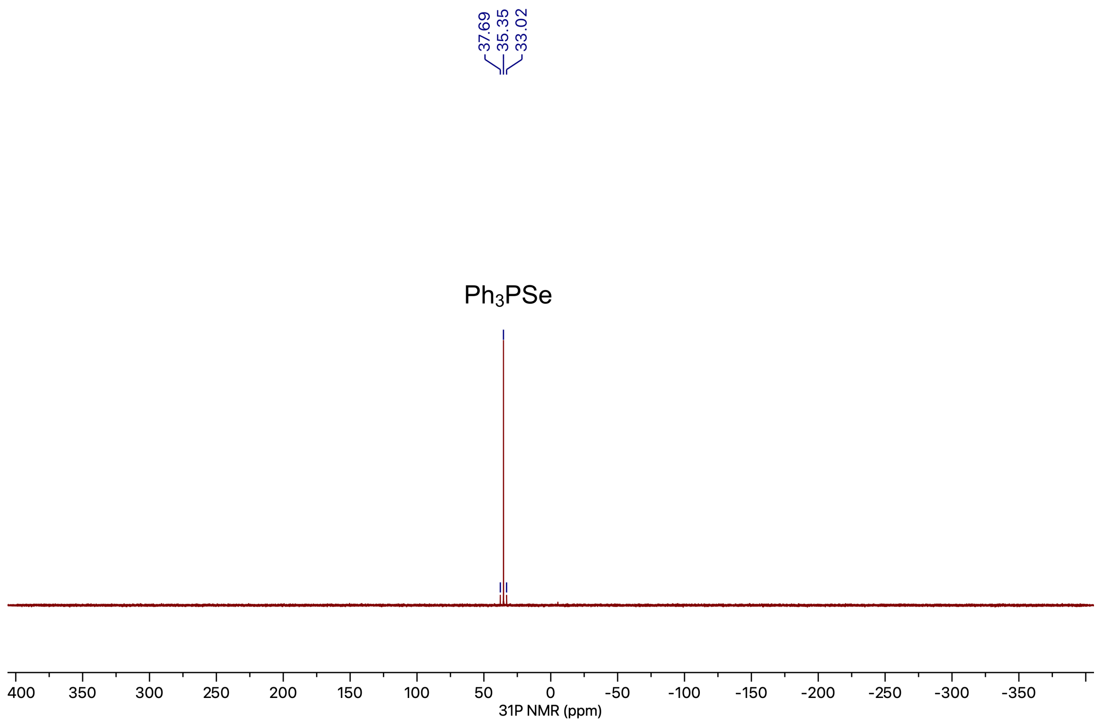

Figure S.70: $^{31}P\{^1H\}$ NMR (162 MHz, benzene-d_6, 25 °C) spectrum of the crude reaction mixture after 48 h.

S.1.18 Treatment of $\{In(TEMPO)\}_2$ with Ph_3PSe, followed by H_2

To a stirring, pale yellow benzene-d_6 solution of **5** (0.0050 g, 0.010 mmol, 1.0 equiv) was added Ph_3PSe (0.0034 g, 0.010 mmol, 1.0 equiv). After several min, the reaction mixture became intensely yellow. After 20 min, the solution was transferred to a J Young tube and analyzed by NMR spectroscopy. The $^{31}P\{^1H\}$ NMR spectrum revealed the complete conversion of Ph_3PSe to Ph_3P (Fig. S.71). Additionally, complete consumption of **5** was observed in the ^1H NMR spectrum and a new broad resonances were observed around 1.7 ppm. (Fig. S.72). An additional ^1H NMR spectrum was collected after 2.5 h, and a resonance, tentatively assigned to tetramethyl piperidine, grew in (Fig. S.73). The growth of this resonance possibly suggests the degradation of an intermediate en route to binary InSe. The NMR sample was degassed by three freeze-pump-thaw cycles and back-filled with H_2 (3 atm). Within several minutes, orange solids precipitated from solution, consistent with the formation of InSe. After 10 min, ^1H NMR data was collected, revealing the clean formation of TEMPOH (Fig. S.74). Additionally, the resonances assigned to tetramethylpiperidine are well-resolved in the ^1H NMR spectrum. These data suggest that Se is directly transferred from Ph_3PSe to **5** in the absence of H_2 and the metastable intermediate that is generated goes on to react with H_2 to form InSe and TEMPOH.

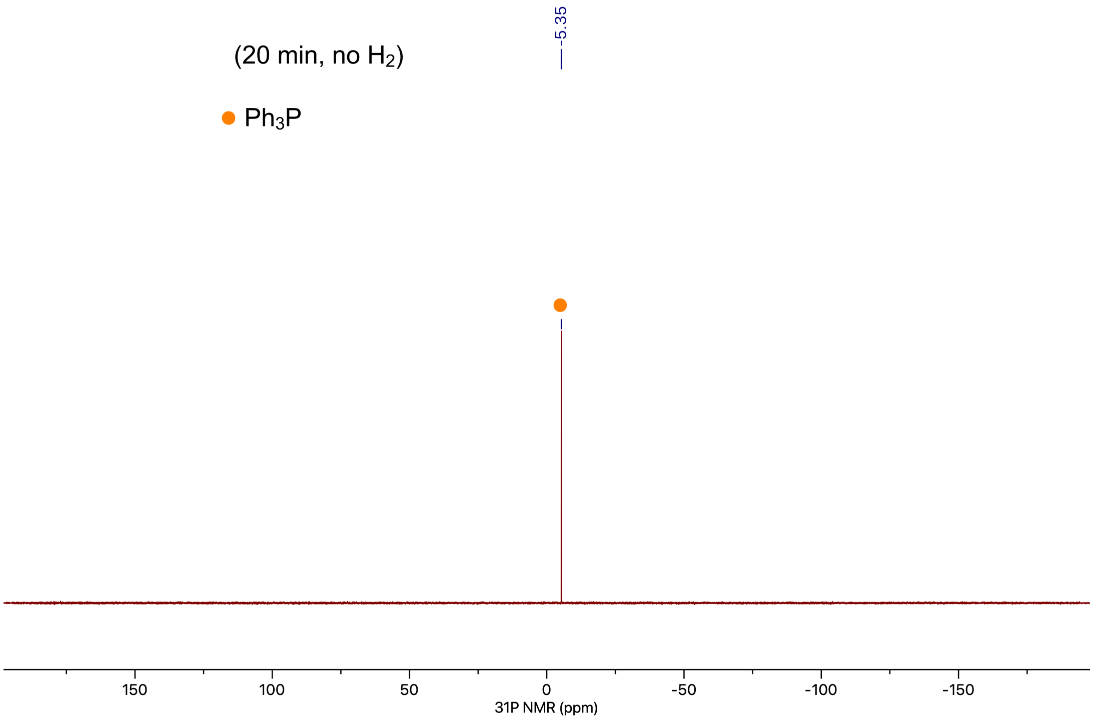

Figure S.71: $^{31}P\{^1H\}$ NMR (243 MHz, benzene-d_6, 25 °C) spectrum of the crude reaction mixture after 20 min, prior to adding in H_2.

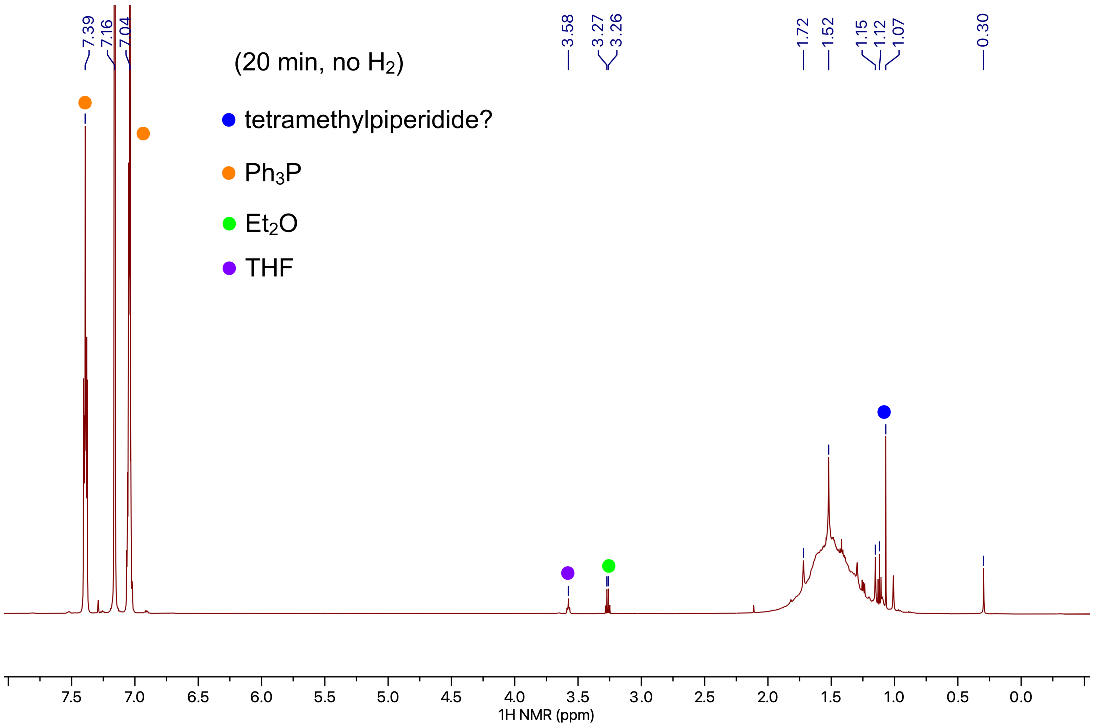

Figure S.72: ^1H NMR (600 MHz, benzene-d_6, 25 °C) spectrum of the crude reaction mixture after 20 min, prior to adding in H_2.

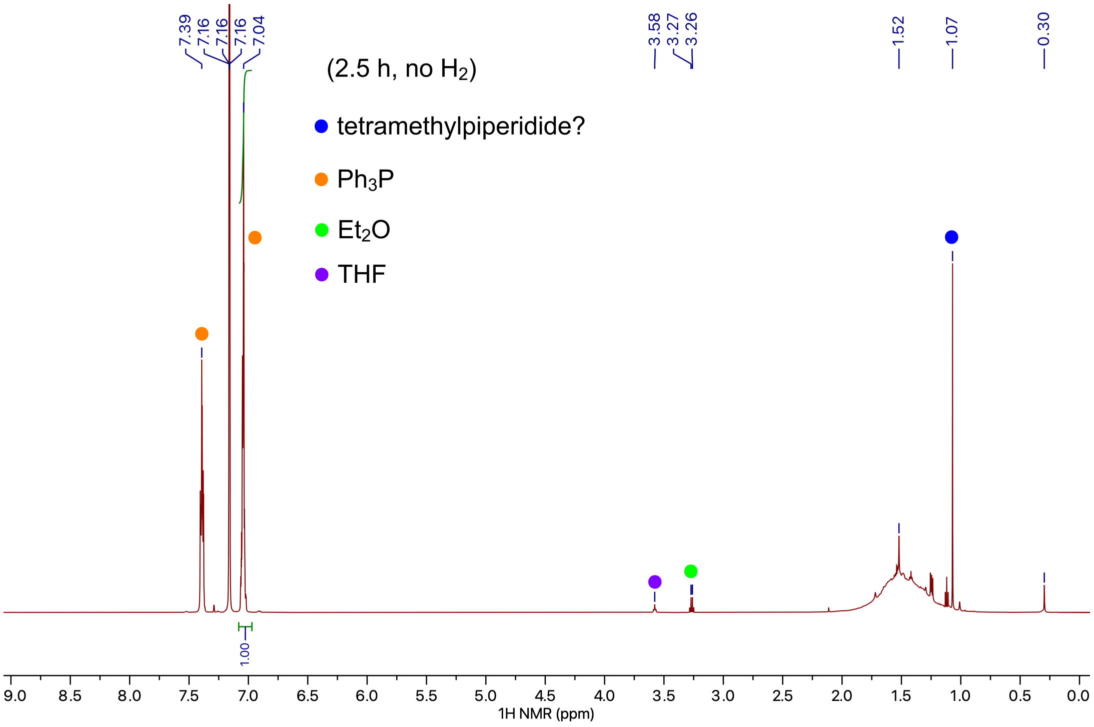

Figure S.73: ^{1}H NMR (600 MHz, benzene-d_6, 25 °C) spectrum of the crude reaction mixture after 2.5 h, prior to adding in H_2.

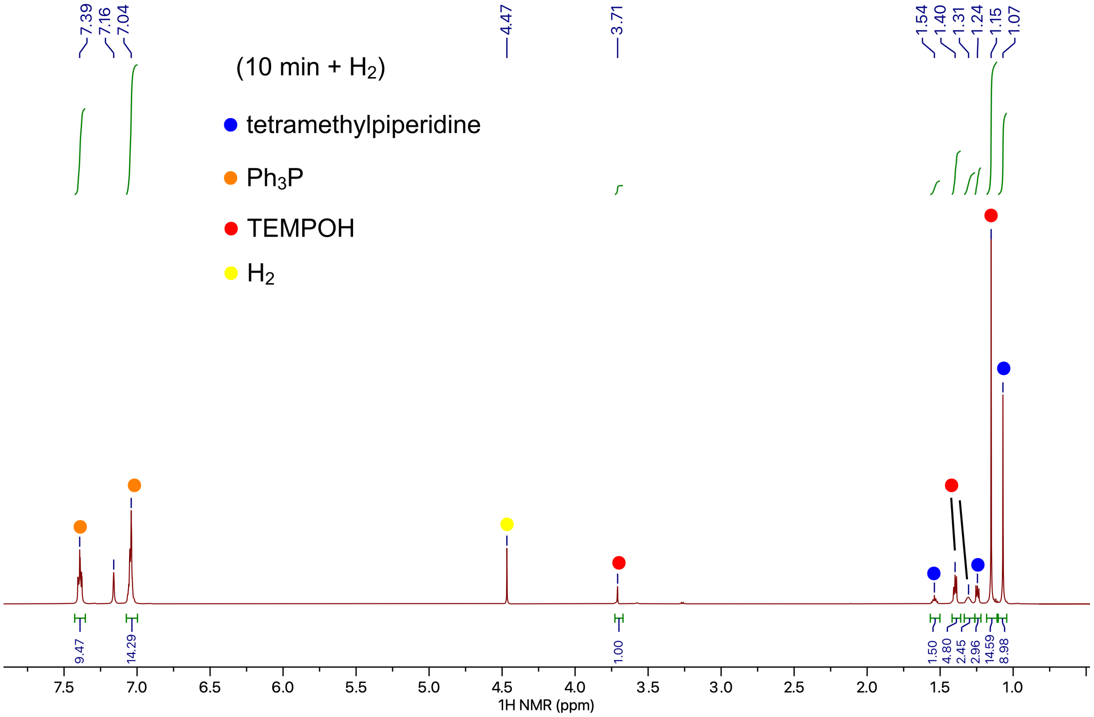

Figure S.74: ^{1}H NMR (600 MHz, benzene-d_6, 25 °C) spectrum of the crude reaction mixture 10 min after adding H_2.

S.1.19 Variable Temperature ^{1}H NMR Study on the Treatment of **3**·THF with H_2

3·THF (0.010 g) was dissolved in benzene-d_6 (0.6 mL) and the solution was transferred to a J Young tube. The sample was removed from the glovebox and degassed by three freeze-pump-thaw cycles. The tube was then backfilled with H_2 (*ca.* 3 atm). A variable temperature ^{1}H NMR experiment was performed on the sample and ^{1}H NMR data were collected at 300 K, 330 K, 343 K, and 300 K (after heating). All data were collected within 1 h of preparing the sample, and the sample was equilibrated at the designated temperatures for several minutes before data collection began (Fig. S.75 and S.76). (TEMPOH)Al(TEMPO)$_3$ was identified as a trace impurity in the starting material. No resonances solely assignable to an H_2 activation product were observed in these experiments. This is consistent with our calculations that suggest the H_2 adduct is endergonic by 5.8 kcal/mol. After heating the sample, **3**·THF dominates the spectrum; however, some resonances assigned to degradation products were observed in the baseline.

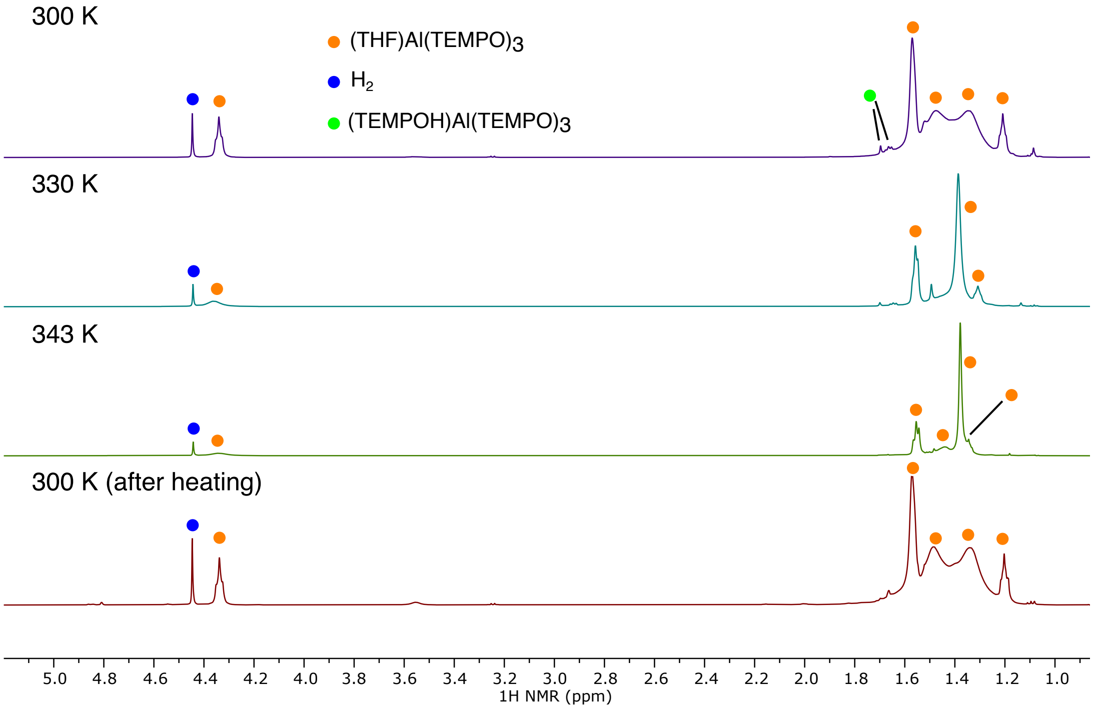

Figure S.75: ^{1}H NMR (400 MHz, benzene-d_6) spectra of **3**·THF at 300 K, 330 K, 343 K, and 300 K (after heating) in the presence of H_2 (3 atm).

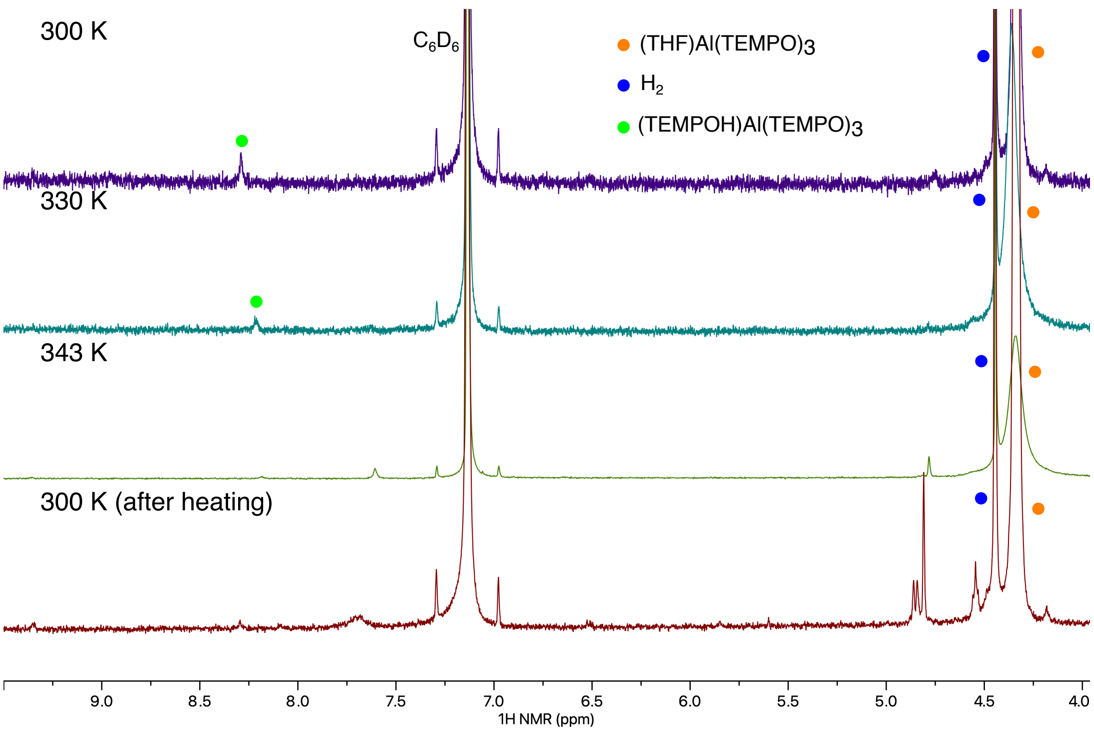

Figure S.76: ^{1}H NMR (400 MHz, benzene-d_6) spectra of **3**·THF at 300 K, 330 K, 343 K, and 300 K (after heating) in the presence of H_2 (3 atm).

S.2 X-Ray Diffraction Studies

Low-temperature (100 K) diffraction data for **1**·THF, **2**·THF, and **3**·THF were collected on a Bruker Apex-II CCD Mo/Cu three-circle diffractometer coupled to a Bruker Apex 2 CCD detector using Cu K_α radiation (λ = 1.54178 Å) while performing ϕ- and ω-scans. Low-temperature (100 K) diffraction data for **4** and **5** were collected on a Bruker D8 VENTURE dual wavelength Mo/Cu three-circle diffractometer with a microfocus sealed X-ray tube using a mirror optics as monochromator and a Bruker PHOTON III detector using Mo K_α radiation (λ = 0.71073 Å) while performing ϕ- and ω-scans. All data were integrated with SAINT and a multi-scan absorption correction using SADABS was applied.[9,10] Crystals were mounted on a MiTeGen Micromount. The structures were solved by direct methods using SHELXT[11] and refined by full-matrix least-squares methods against F^2 by SHELXL-2018/3[12] following established refinement strategies[13]. All non-hydrogen atoms were refined anisotropically. Unless otherwise noted, all hydrogen atoms were included into the model at geometrically calculated positions and refined using a riding model. The isotropic displacement parameters of all hydrogen atoms were fixed to 1.2 times the U-value of the atoms they are linked to (1.5 times for methyl groups). Details of the data quality and a summary of the residual values of the refinement are listed in Tables S.3-S.7. Further details can be found in the form of .cif files available from the Cambridge Crystallographic Data Centre.[14] Parts of this report and the .cif file were generated using FinalCif.[15]

A colorless, block-shaped crystal of **1**·THF was grown from cold hexanes and crystallized in the triclinic space group $P\bar{1}$ with one molecule of **1**·THF in the asymmetric unit (Fig. S.77). The molecule exhibits no disorder. A yellow, block-shaped crystal of **2**·THF was grown from cold diethyl ether and crystallized in the triclinic space group $P\bar{1}$ with one molecule of **2**·THF in the asymmetric unit (Fig. S.78). One TEMPO ligand and the THF ligand were disordered over two positions and were refined with the help of similarity restraints on 1−2 and 1−3 positions and displacement parameters. The disorder ratios for the TEMPO and THF ligands were refined freely and converged at 0.791(9) and 0.64(3), respectively. A colorless, block-

shaped crystal of **3**·THF was grown from cold hexanes and crystallized in the triclinic space group $P\bar{1}$ with one molecule of **3**·THF in the asymmetric unit (Fig. S.79). The crystal was twinned; therefore, the orientation matrices for two components was determined using CELL_NOW[16] and the twin ratio refined to 0.496(6). The molecule exhibits no disorder. A colorless, block-shaped crystal of **4** was grown from cold diethyl ether and crystallized in the monoclinic space group $P2_1/n$ with one molecule of **4** in the asymmetric unit (Fig. S.80). The molecule exhibits no disorder. A yellow, plate-shaped crystal of **5** was grown from cold diethyl ether and crystallized in the orthorhombic space group $Cmca$ with one quarter molecule of **5** (Fig. S.81). The molecule exhibits no disorder.

Table S.3: Crystallographic Data for **1**·THF

CCDC	CSD 2347343
Empirical formula, FW (g/mol)	$C_{31}H_{62}GaN_3O_4$, 610.55
Color / Morphology	Colorless / Block
Crystal size (mm^3)	$0.429 \times 0.296 \times 0.188$
Temperature (K)	100(2)
Wavelength (Å)	1.54178
Crystal system, Space group	Triclinic, $P\bar{1}$
Unit cell dimensions (Å, °)	$a = 11.7627(4)$, $\alpha = 118.4250(10)$<br>$b = 12.4302(5)$, $\beta = 91.2110(10)$<br>$c = 12.9451(5)$, $\gamma = 90.1390(10)$
Volume (Å^3)	1664.01(11)
Z	2
Density (calc., g/cm^3)	1.219
Absorption coefficient (mm^{-1})	1.413
$F(000)$	664
Theta range for data collection (°)	3.759 to 72.282
Index ranges	$-14 \leq h \leq 14$, $-15 \leq k \leq 15$,<br>$-15 \leq l \leq 14$
Reflections collected	49209
Independent reflections, R_{int}	6313, 0.0322
Completeness to θ_{max} (%)	96.6
Absorption correction	Semi-empirical from equivalents
Refinement method	Full-matrix least-squares on F^2
Data / Restraints / Parameters	6313 / 1 / 364
Goodness-of-fit[a]	1.085
Final R indices[b] $[I > 2\sigma(I)]$	$R_1 = 0.0306$, $wR_2 = 0.0791$
R indices[b] (all data)	$R_1 = 0.0320$, $wR_2 = 0.0800$
Largest diff. peak and hole (e·Å^{-3})	0.284 and -0.718

[a] GooF $= \sqrt{\frac{\Sigma[w(F_o^2-F_c^2)^2]}{(n-p)}}$ [b] $R_1 = \frac{\Sigma||F_o|-|F_c||}{\Sigma|F_o|}$; $wR_2 = \sqrt{\frac{\Sigma[w(F_o^2-F_c^2)^2}{\Sigma[w(F_o^2)^2]}}$; $w = \frac{1}{\sigma^2(F_o^2)+(aP)^2+bP}$; $P = \frac{2F_c^2+\max(F_o^2,0)}{3}$

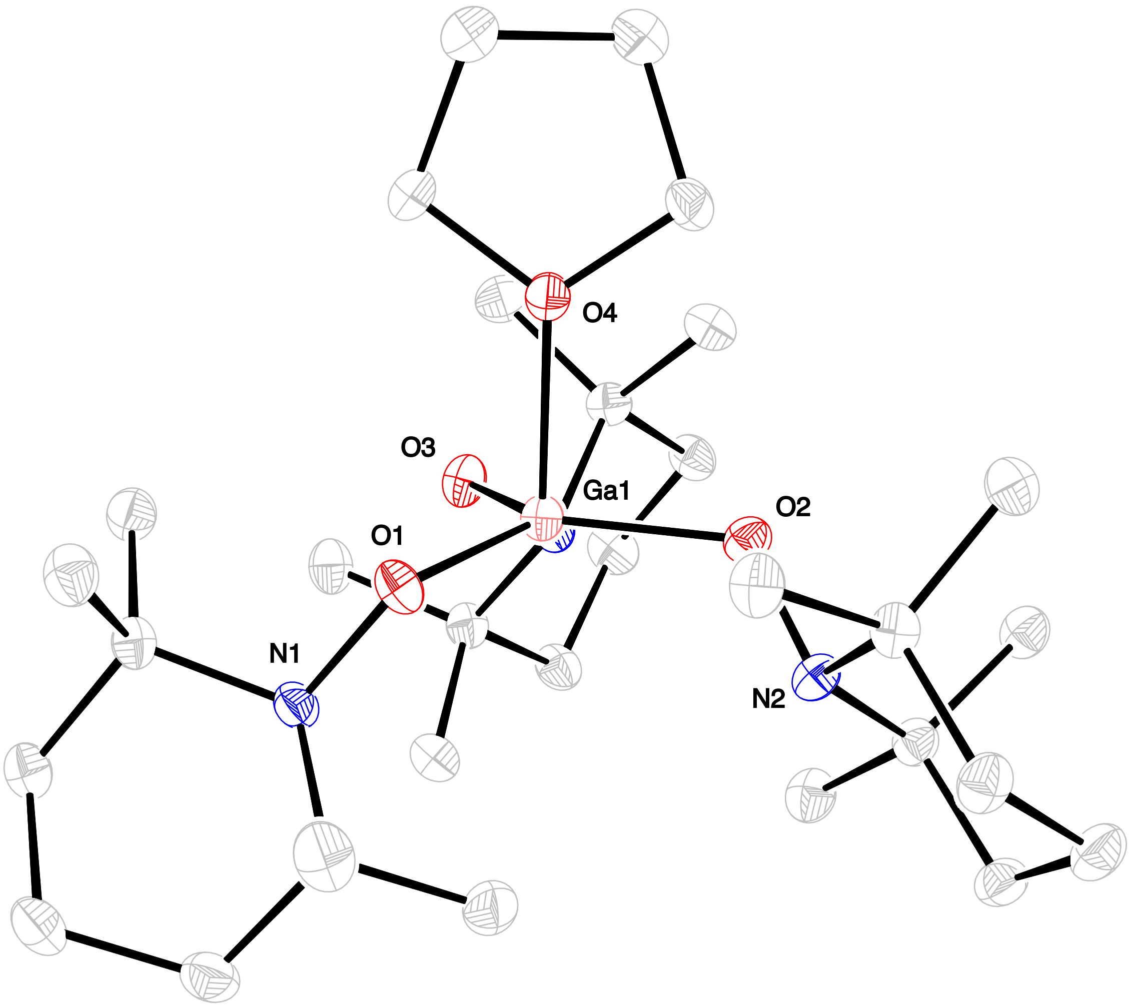

Figure S.77: Molecular structure of **1**·THF with thermal ellipsoids shown at the 50% probability level. Hydrogen atoms are omitted for clarity.

Table S.4: Crystallographic Data for **2**·THF

CCDC	CSD 2347342
Empirical formula, FW (g/mol)	$C_{2.95}H_{5.90}In_{0.10}N_{0.29}O_{0.38}$, 62.44
Color / Morphology	Yellow / Block
Crystal size (mm^3)	$0.487 \times 0.271 \times 0.221$
Temperature (K)	100(2)
Wavelength (Å)	1.54178
Crystal system, Space group	Triclinic, $P\bar{1}$
Unit cell dimensions (Å, °)	$a = 11.9190(3)$, $\alpha = 61.5040(10)$
	$b = 12.4961(3)$, $\beta = 89.2340(10)$
	$c = 13.0349(3)$, $\gamma = 89.8010(10)$
Volume (Å^3)	1706.07(7)
Z	21
Density (calc., g/cm^3)	1.276
Absorption coefficient (mm^{-1})	5.810
$F(000)$	700
Theta range for data collection (°)	3.709 to 70.126
Index ranges	$-14 \leq h \leq 14$, $-15 \leq k \leq 15$,
	$-15 \leq l \leq 15$
Reflections collected	42260
Independent reflections, R_{int}	6251, 0.0307
Completeness to θ_{max} (%)	96.7
Absorption correction	Semi-empirical from equivalents
Refinement method	Full-matrix least-squares on F^2
Data / Restraints / Parameters	6251 / 771 / 514
Goodness-of-fit^a	1.012
Final R indices^b $[I > 2\sigma(I)]$	$R_1 = 0.0272$, $wR_2 = 0.1050$
R indices^b (all data)	$R_1 = 0.0280$, $wR_2 = 0.1067$
Largest diff. peak and hole (e·Å^{-3})	1.159 and -1.294

^{a} GooF $= \sqrt{\frac{\Sigma[w(F_o^2-F_c^2)^2]}{(n-p)}}$ ^{b} $R_1 = \frac{\Sigma||F_o|-|F_c||}{\Sigma|F_o|}$; $wR_2 = \sqrt{\frac{\Sigma[w(F_o^2-F_c^2)^2}{\Sigma[w(F_o^2)^2]}}$; $w = \frac{1}{\sigma^2(F_o^2)+(aP)^2+bP}$; $P = \frac{2F_c^2+\max(F_o^2,0)}{3}$

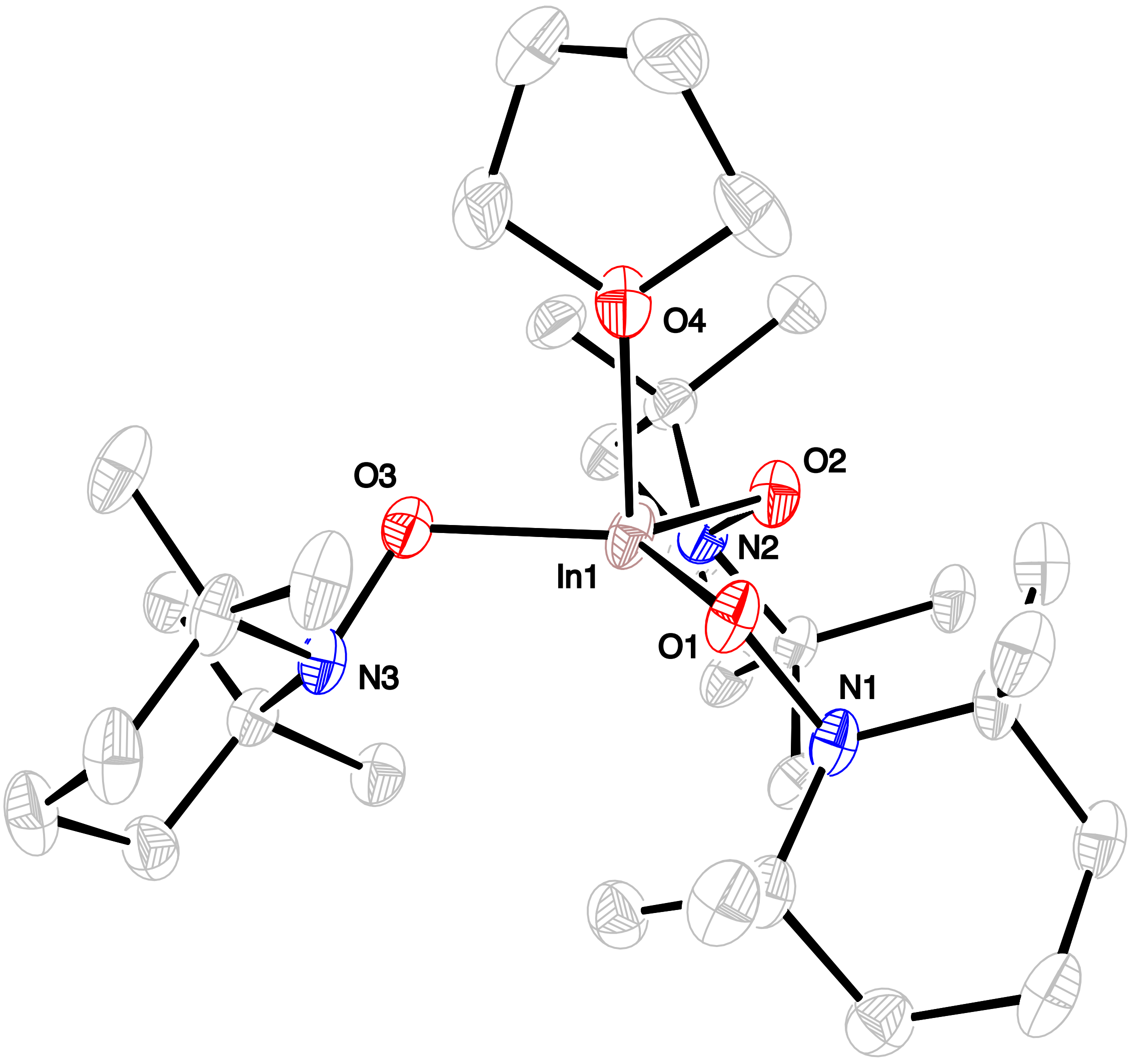

Figure S.78: Molecular structure of **2**·THF with thermal ellipsoids shown at the 50% probability level. Hydrogen atoms are omitted for clarity.

Table S.5: Crystallographic Data for **3**·THF

CCDC	CSD 2347344
Empirical formula, FW (g/mol)	$C_{31}H_{62}AlN_3O_4$, 567.81
Color / Morphology	Colorless / Block
Crystal size (mm^3)	$0.399 \times 0.292 \times 0.230$
Temperature (K)	100(2)
Wavelength (Å)	1.54178
Crystal system, Space group	Triclinic, $P\bar{1}$
Unit cell dimensions (Å, °)	$a = 11.7623(4)$, $\alpha = 118.532(2)$
	$b = 12.4407(5)$, $\beta = 91.442(2)$
	$c = 12.9344(5)$, $\gamma = 90.128(2)$
Volume (Å^3)	1662.09(11)
Z	2
Density (calc., g/cm^3)	1.135
Absorption coefficient (mm^{-1})	0.816
$F(000)$	628
Theta range for data collection (°)	3.759 to 68.502
Index ranges	$-14 \leq h \leq 14$, $-14 \leq k \leq 13$,
	$0 \leq l \leq 15$
Reflections collected	7779
Independent reflections, R_{int}	7779, 0.0454
Completeness to $\theta_{\max}$ (%)	96.4
Absorption correction	Semi-empirical from equivalents
Refinement method	Full-matrix least-squares on F^2
Data / Restraints / Parameters	7779 / 0 / 365
Goodness-of-fit^a	1.116
Final R indices^b $[I > 2\sigma(I)]$	$R_1 = 0.0493$, $wR_2 = 0.1465$
R indices^b (all data)	$R_1 = 0.0550$, $wR_2 = 0.1484$
Largest diff. peak and hole (e·Å^{-3})	0.436 and -0.268

^{a} GooF $= \sqrt{\frac{\Sigma[w(F_o^2 - F_c^2)^2]}{(n-p)}}$ ^{b} $R_1 = \frac{\Sigma||F_o|-|F_c||}{\Sigma|F_o|}$; $wR_2 = \sqrt{\frac{\Sigma[w(F_o^2 - F_c^2)^2}{\Sigma[w(F_o^2)^2]}}$; $w = \frac{1}{\sigma^2(F_o^2)+(aP)^2+bP}$; $P = \frac{2F_c^2+\max(F_o^2,0)}{3}$

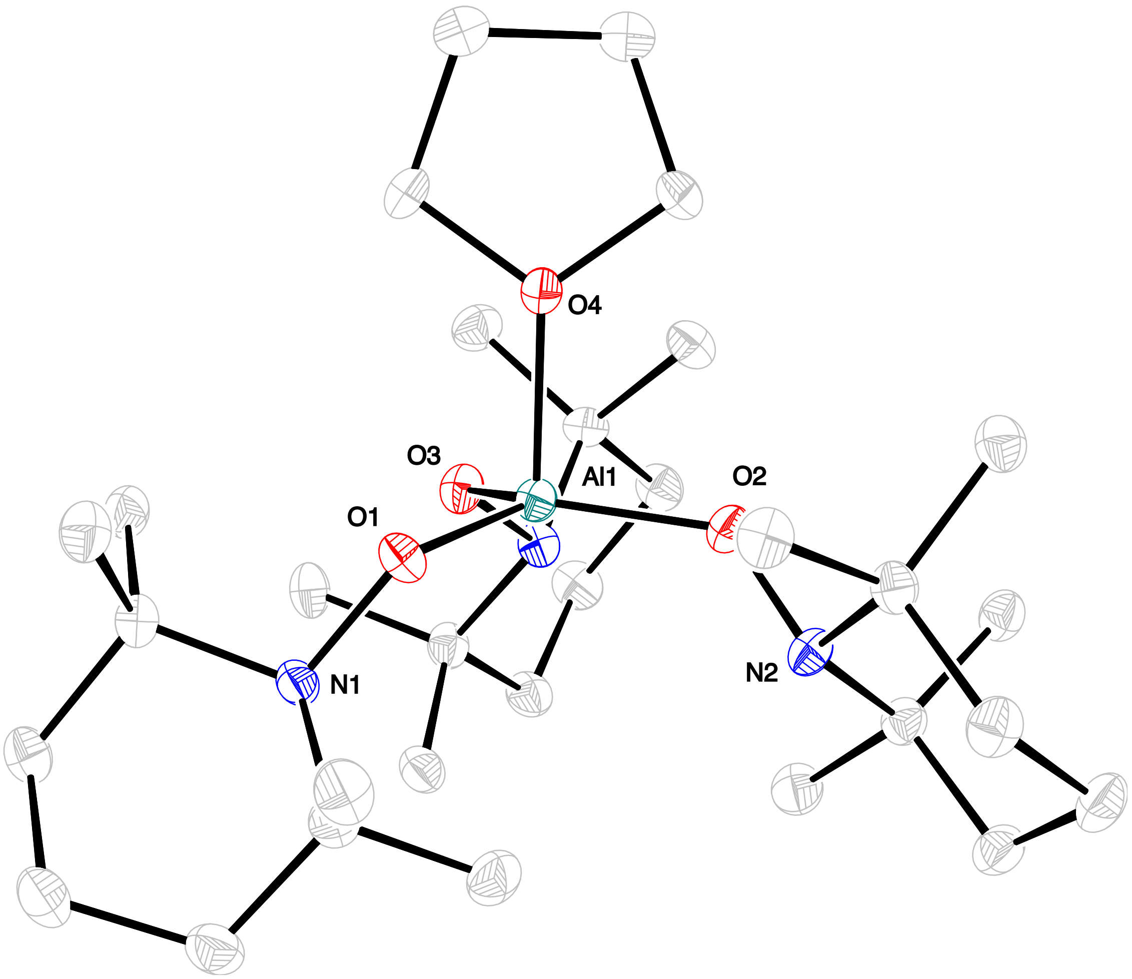

Figure S.79: Molecular structure of **3**·THF with thermal ellipsoids shown at the 50% probability level. Hydrogen atoms are omitted for clarity.

Table S.6: Crystallographic Data for **4**

CCDC	CSD 2347345
Empirical formula, FW (g/mol)	$C_{27}H_{56}GaN_3O_3$, 540.46
Color / Morphology	Colorless / Block
Crystal size (mm^3)	$0.265 \times 0.176 \times 0.129$
Temperature (K)	100(2)
Wavelength (Å)	0.71073
Crystal system, Space group	Monoclinic, $P2_1/n$
Unit cell dimensions (Å, °)	$a = 12.0657(5)$, $\alpha = 90$
	$b = 12.8632(5)$, $\beta = 94.979(2)$
	$c = 19.2485(9)$, $\gamma = 90$
Volume (Å^3)	2976.2(2)
Z	4
Density (calc., g/cm^3)	1.206
Absorption coefficient (mm^{-1})	0.954
$F(000)$	1176
Theta range for data collection (°)	3.81 to 59.18
Index ranges	$-16 \leq h \leq 16$, $-17 \leq k \leq 17$, $-26 \leq l \leq 26$
Reflections collected	50196
Independent reflections, R_{int}	8369, 0.0924
Completeness to θ_{max} (%)	100.0
Absorption correction	Semi-empirical from equivalents
Refinement method	Full-matrix least-squares on F^2
Data / Restraints / Parameters	8369/0/326
Goodness-of-fit[a]	1.045
Final R indices[b] $[I > 2\sigma(I)]$	$R_1 = 0.0422$, $wR_2 = 0.0831$
R indices[b] (all data)	$R_1 = 0.0567$, $wR_2 = 0.0918$
Largest diff. peak and hole ($e{\cdot}$Å^{-3})	0.43 and −0.40

[a] GooF $= \sqrt{\frac{\Sigma[w(F_o^2-F_c^2)^2]}{(n-p)}}$ [b] $R_1 = \frac{\Sigma||F_o|-|F_c||}{\Sigma|F_o|}$; $wR_2 = \sqrt{\frac{\Sigma[w(F_o^2-F_c^2)^2}{\Sigma[w(F_o^2)^2]}}$; $w = \frac{1}{\sigma^2(F_o^2)+(aP)^2+bP}$; $P = \frac{2F_c^2+\max(F_o^2,0)}{3}$

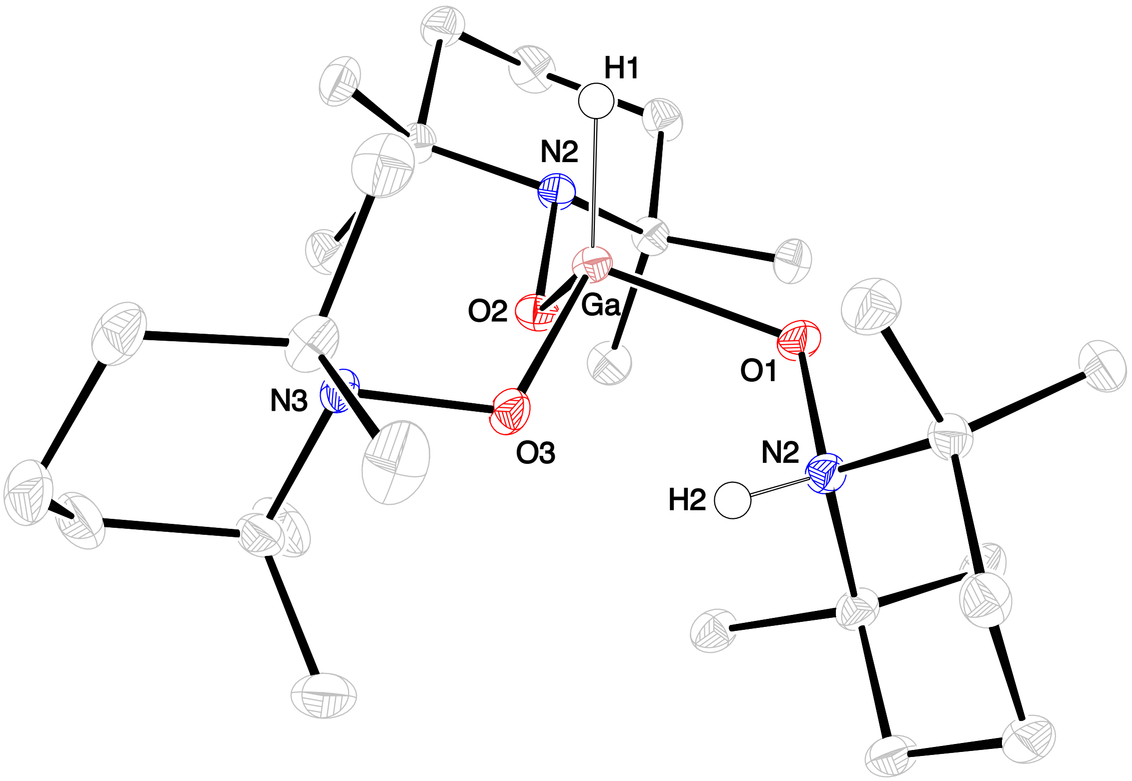

Figure S.80: Molecular structure of **4** with thermal ellipsoids shown at the 50% probability level. Selected hydrogen atoms are omitted for clarity.

Table S.7: Crystallographic Data for **5**

CCDC	CSD 2347346
Empirical formula, FW (g/mol)	$C_9H_{18}InNO$, 271.06
Color / Morphology	Yellow / Plate
Crystal size (mm^3)	$0.374 \times 0.300 \times 0.198$
Temperature (K)	100(2)
Wavelength (Å)	0.71073
Crystal system, Space group	Orthorhombic, $Cmca$
Unit cell dimensions (Å, °)	$a = 12.4969(10)$, $\alpha = 90$
	$b = 9.4605(5)$, $\beta = 90$
	$c = 18.0744(11)$, $\gamma = 90$
Volume (Å^3)	2136.9(2)
Z	8
Density (calc., g/cm^3)	1.685
Absorption coefficient (mm^{-1})	2.170
$F(000)$	1088
Theta range for data collection (°)	4.51 to 55.05
Index ranges	$-16 \leq h \leq 16$, $-12 \leq k \leq 12$,
	$-23 \leq l \leq 23$
Reflections collected	17176
Independent reflections, R_{int}	1291, 0.0797
Completeness to θ_{max} (%)	100.0
Absorption correction	Semi-empirical from equivalents
Refinement method	Full-matrix least-squares on F^2
Data / Restraints / Parameters	1291/0/63
Goodness-of-fit^a	1.035
Final R indices^b $[I > 2\sigma(I)]$	$R_1 = 0.0203$, $wR_2 = 0.0425$
R indices^b (all data)	$R_1 = 0.0283$, $wR_2 = 0.0443$
Largest diff. peak and hole (e·Å^{-3})	0.36 and -0.42

^{a} GooF $= \sqrt{\frac{\Sigma[w(F_o^2-F_c^2)^2]}{(n-p)}}$ ^{b} $R_1 = \frac{\Sigma||F_o|-|F_c||}{\Sigma|F_o|}$; $wR_2 = \sqrt{\frac{\Sigma[w(F_o^2-F_c^2)^2}{\Sigma[w(F_o^2)^2]}}$; $w = \frac{1}{\sigma^2(F_o^2)+(aP)^2+bP}$; $P = \frac{2F_c^2+\max(F_o^2,0)}{3}$

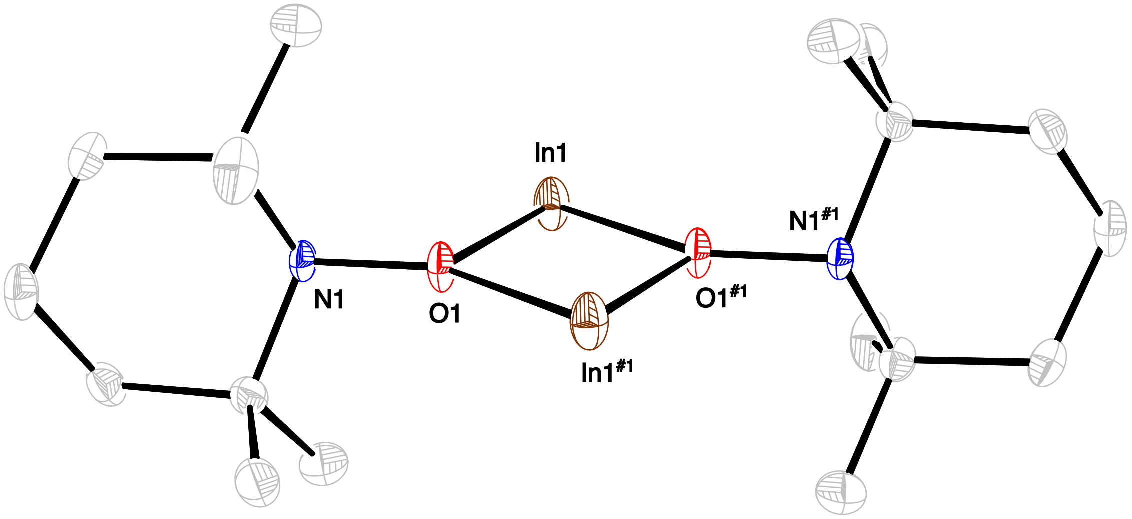

Figure S.81: Molecular structure of **5** with thermal ellipsoids shown at the 50% probability level. Hydrogen atoms are omitted for clarity.

S.3 Computational Studies

S.3.1 Reaction Pathway Calculations

S.3.1.1 General Information

Density functional theory (DFT) calculations were performed in Gaussian 16, Revision C.01.[17] Geometries for intermediates and transition states were optimized using the PBE0[18,19] functional with Grimme's D3 dispersion correction[20] and Becke-Johnson damping[21] (D3BJ) and with the def2-SVP[22] basis set, which include integral equation formalism of the polarizable continuum model (IEFPCM) of solvation[23] (solvent = benzene). Frequencies were computed at the same level of theory to determine whether the structures are minima (with no imaginary frequency) or transition states (with only one imaginary frequency) and to obtain zero-point energy and thermal energy corrections under 298.15 K and 1 atm pressure. Single-point solvation energies were calculated using the PBE0 functional with Grimme's D3 dispersion correction with Becke-Johnson damping[21] and with the def2-TZVPP basis set and solvent model density (SMD) continuum solvation model[24] (solvent = benzene). Grimme's

correction for entropy[25] were performed using Goodvibes v3.0.1.[26] Structures of transition states were illustrated using CYLview 1.0b.[27] Cartesian coordinates for structures shown in Figures 4, S.82, and S.83 are provided in section S.3.1.4. Cartesian coordinates for structures shown in Figure S.84 are provided in section S.3.1.5.

S.3.1.2 H_2 Activation

Fig. S.82 shows the proposed computed pathway after the formation of indium complex **IN3** (Fig. 5A). We should note that bimolecular pathways involving In−H−In intermediates have not been ruled out. Dissociation of the protonated TEMPO ligand is endergonic by 5.4 kcal/mol, forming the indium monohydride **IN4**. Similar to **IN1**, **IN4** is three-coordinate and trivalent and may possess the ability to activate dihydrogen as well. H_2 addition proceeds with a barrier of 15.0 kcal/mol *via* **TS2**, which is significantly higher than that of the initial H_2 activation *via* **TS1** (Fig. 5A), leading to H_2 complex **IN6**, which could go on to reductively eliminate H_2 to form a monovalent In center. However, we were unable to successfully locate the transition state for this process, presumably due to the involvement of bimolecular or more complicated reactions.[28] Nevertheless, DFT calculations suggest that **IN4** can undergo hydrogen atom transfer, directly forming **IN6** with an energy barrier of 19.9 kcal/mol via **TS2'**, which is feasible at room temperature. **IN6** readily undergoes further loss of the protonated TEMPO ligand, and subsequently dimerizes to form the thermodynamically favored **IN8** (C_{2h} symmetry), which is more stable by 39.4 kcal/mol as compared to complex **2**·THF (Fig. 5A). Similar to the reactions described above, a third dihydrogen activation could occur at **IN5** through transition state **TS3**, eventually affording the indium trihydride **IN10** and **IN11**. Alternatively, **IN9** could undergo hydrogen atom transfer via **TS3'**. However, all of these pathways are energetically uphill, making them substantially disfavored compared to the formation of **IN8**.

Fig. S.83 shows the calculated pathways that potentially lead to the formation of gallium dimer **IN8**, which should be isostructural to **5**, after the formation of **4** (Fig. 5A). The

energy profile clearly indicates that all of the possible intermediates and transition states are disfavored due to their higher free energies as compared to the isolated gallium complex **4**. We hope all of these results can provide more insight into the intrinsic reactivity and stability of indium and gallium complexes investigated in this work.

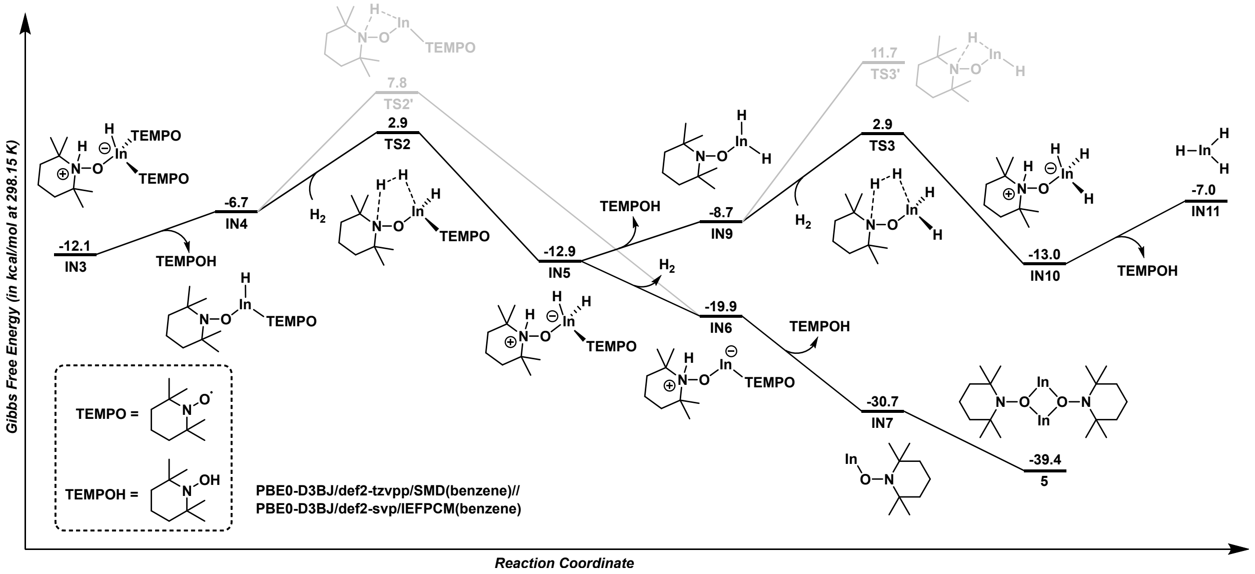

Figure S.82: Computed reaction pathway for the formation of **5**.

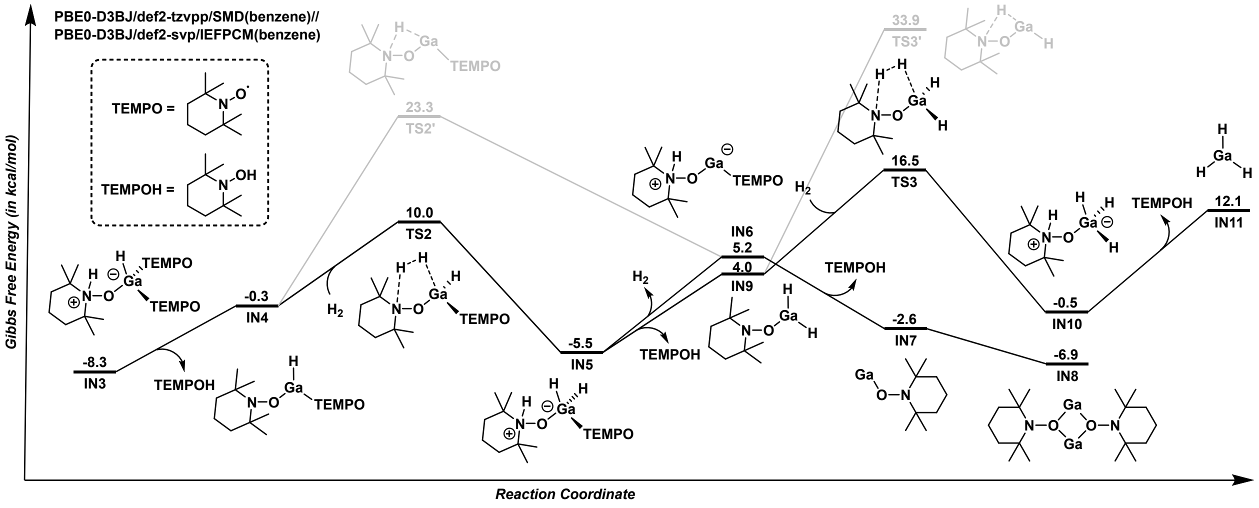

Figure S.83: Computed reaction pathway for further H_2 reactivity with **4**.

S.3.1.3 Benzene C−H Activation by **1**·THF

Fig. S.84 shows the calculated pathway for C−H activation of benzene by **1**·THF. The energy profile suggests that the barrier for C−H activation is surmountable at room temperature; however, the product of C−H insertion (IN3) is uphill by 5.0 kcal/mol. This step may be followed by dissociation of TEMPOH and degradation of the resulting gallium complex.

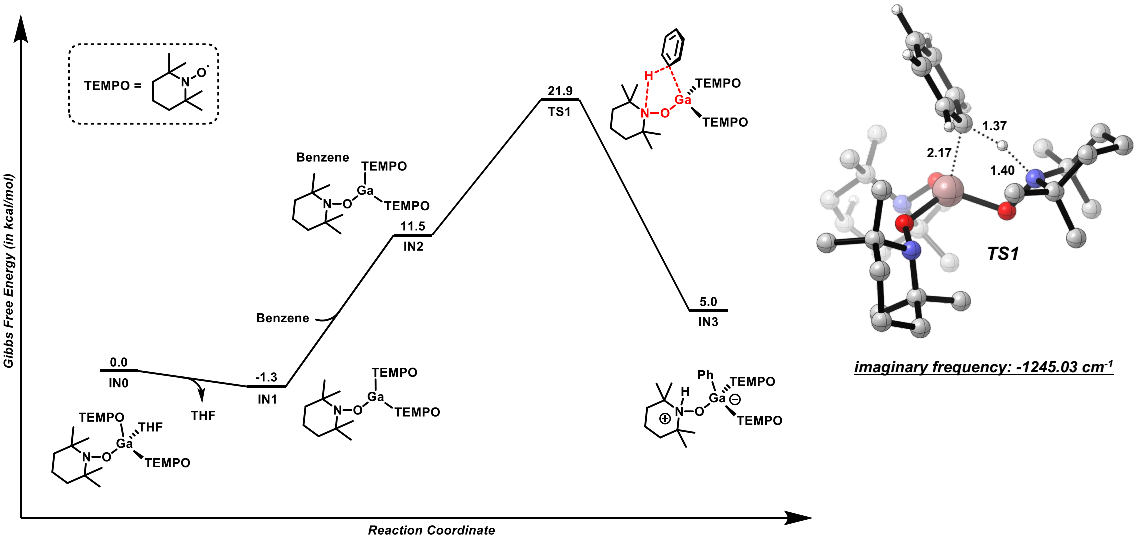

Figure S.84: Computed reaction pathway for the C−H activation of benzene by **1**·THF. The structure of TS1 and associated bond lengths (Å) are shown to the right.

S.3.1.4 Cartesian Coordinates of DFT Optimized Structures Involved in H_2 Activation

2

Dihydrogen (H2)

H	0.00000000	0.00000000	0.38047200
H	0.00000000	0.00000000	-0.38047200

13

Tetrahydrofuran (THF)

O	-0.00002600	1.23654200	-0.00000600
C	1.15418400	0.42874300	-0.13433700
C	0.72582100	-0.98618900	0.23272600
H	1.95239700	0.82172500	0.51676900
H	1.52390700	0.47025000	-1.17766300
C	-1.15419600	0.42870300	0.13434600
C	-0.72578600	-0.98621400	-0.23273000
H	-1.95242700	0.82166100	-0.51675200
H	-1.52391400	0.47019200	1.17767500
H	-0.77649100	-1.13282200	-1.32406100
H	-1.34550300	-1.76042500	0.24195800
H	0.77652800	-1.13280600	1.32405500
H	1.34557100	-1.76037100	-0.24196400

30

TEMPOH

C	-1.23931300	1.41723400	-0.47818400
C	-1.28114100	-0.04973000	-0.03233100
C	1.28114200	-0.04972100	-0.03234000
C	1.23929700	1.41726600	-0.47812000
C	-0.00003000	2.14988700	0.00502500
H	-1.25944000	1.44245200	-1.58112400
H	-2.15986700	1.91291300	-0.13010100
H	2.15981900	1.91295000	-0.12995900
H	1.25949600	1.44252900	-1.58105600
H	-0.00006100	2.22653700	1.10504200
H	-0.00002900	3.18527900	-0.37127600
C	1.61216700	-0.16704300	1.46270100
H	1.43896800	-1.19685400	1.80459200
H	2.67201900	0.07708500	1.63158000
H	1.01501500	0.50929200	2.08740200

C	2.37420900	-0.76983300	-0.82463100
H	3.34356800	-0.26817400	-0.68402200
H	2.47755400	-1.81241300	-0.49238200
H	2.12864400	-0.76526100	-1.89740400
C	-1.61216500	-0.16696200	1.46270300
H	-2.67200400	0.07722300	1.63158200
H	-1.43901000	-1.19676700	1.80460700
H	-1.01497000	0.50935900	2.08737600
C	-2.37420200	-0.76990600	-0.82457500
H	-2.47758000	-1.81245800	-0.49223400
H	-3.34356500	-0.26824400	-0.68401100
H	-2.12863900	-0.76541800	-1.89735100
N	0.00000500	-0.66703200	-0.42216300
O	0.00012300	-2.01197600	0.00068400
H	-0.00073200	-2.51214800	-0.82308000

101

INO (A1)

O	-1.55042900	-0.04952800	-0.76391700
O	0.76377200	1.40263800	0.49482400
O	0.81916800	-1.54999700	0.02898600
N	-2.80926500	-0.52870100	-0.36962500
N	0.68883300	2.63074700	-0.18487100
N	2.18158000	-1.61883900	-0.30040600
C	-3.18753000	-1.63487000	-1.28055100
C	-2.23166700	-2.79629400	-1.00416200
H	-2.47608700	-3.65211000	-1.65128600
H	-2.31733200	-3.12585600	0.04113300
H	-1.18883300	-2.50531300	-1.18590300
C	-3.08297300	-1.28253200	-2.77186400
H	-3.90178100	-0.64120400	-3.12101400
H	-3.10372800	-2.20209200	-3.37596300
H	-2.13299500	-0.76389800	-2.95903100
C	-4.60626200	-2.08900700	-0.91529800
H	-4.91381500	-2.87147600	-1.62784200
H	-4.56908600	-2.56395600	0.08063900
C	-5.60937900	-0.95148900	-0.87326900
H	-6.59940100	-1.32529500	-0.56607800
H	-5.74562300	-0.51783900	-1.87774900
C	-5.11881900	0.10499700	0.09914500
H	-5.08359700	-0.33292600	1.11214300

H	-5.81105800	0.96124400	0.14634400
C	-3.72089000	0.63183300	-0.24501900
C	-3.76048700	1.54385100	-1.47995000
H	-4.34343600	1.12290400	-2.30835400
H	-2.73580700	1.72510000	-1.83044800
H	-4.21334500	2.51145600	-1.21558800
C	-3.22072600	1.46846900	0.93286900
H	-2.23456600	1.90265500	0.71402800
H	-3.14934300	0.84349100	1.83490800
H	-3.91846300	2.29446900	1.13748300
C	1.37231000	2.52206800	-1.49318700
C	2.78915600	1.93372300	-1.43054700
H	3.15124600	1.73826000	-2.45209100
H	2.77612600	0.97594500	-0.88946700
H	3.51134900	2.60421000	-0.94797900
C	0.51827100	1.61192100	-2.37390100
H	-0.53085000	1.93430700	-2.35823100
H	0.55749000	0.56168300	-2.05470300
H	0.88885800	1.62943200	-3.41010500
C	1.37025000	3.90768300	-2.15357500
H	1.93348300	3.83682400	-3.09779700
H	0.32917700	4.15733600	-2.42112800
C	1.91652000	5.00287200	-1.26059300
H	1.86063000	5.97790400	-1.77081700
H	2.98370200	4.83103100	-1.04250900
C	1.10576400	5.02821900	0.01973700
H	0.06040800	5.28256200	-0.22589900
H	1.46945100	5.80315200	0.71375300
C	1.11061800	3.68498200	0.75963800
C	2.47110100	3.43551600	1.42867300
H	3.32032100	3.62878700	0.76074500
H	2.52700700	2.39170700	1.76541800
H	2.58478000	4.09234400	2.30484300
C	0.05179600	3.75993300	1.86031300
H	-0.95377600	3.79827800	1.41632800
H	0.20414700	4.66173900	2.47301300
H	0.11369900	2.88744600	2.52020200
C	2.33165800	-2.51435400	-1.46770500
C	1.72733800	-3.91418700	-1.27474600
H	0.73879600	-3.82568700	-0.80428200
H	2.34998100	-4.57172000	-0.65533100

H	1.60084400	-4.40577400	-2.25126200
C	1.62113500	-1.85485200	-2.64904000
H	0.53829300	-1.78609600	-2.47699900
H	1.78683200	-2.44437100	-3.56346200
H	2.01543200	-0.84298700	-2.81277900
C	3.82545800	-2.59323000	-1.80802300
H	3.95460200	-3.30234800	-2.64180600
H	4.14235600	-1.60297000	-2.17746100
C	4.69223400	-2.97206200	-0.62208200
H	5.75634700	-2.96159600	-0.90796900
H	4.47502100	-4.00351300	-0.29759900
C	4.44225000	-1.99482000	0.51126100
H	4.76493200	-0.98804100	0.19543600
H	5.03443700	-2.25629600	1.40337500
C	2.96554300	-1.91202200	0.91818700
C	2.53152600	-3.17610000	1.67757400
H	2.93049600	-3.15171400	2.70341300
H	2.88226300	-4.10502700	1.21160600
H	1.43507900	-3.21499400	1.73557200
C	2.80604300	-0.72372400	1.86626000
H	2.95636500	0.22325700	1.33151400
H	3.53308300	-0.79848700	2.68911900
H	1.80091700	-0.70700100	2.30804300
O	-0.75106100	-0.45037400	1.99841000
C	-0.67047800	0.39061000	3.17102800
C	-1.32616100	-0.42683400	4.26848400
H	-1.18200500	1.33430200	2.95175400
H	0.39263500	0.59459900	3.36495800
C	-1.26515500	-1.76519400	2.32677800
C	-1.07100500	-1.86286800	3.82238700
H	-0.68975800	-2.48616700	1.73264100
H	-2.31995900	-1.78595700	2.01504100
H	-0.03815900	-2.16734800	4.05519000
H	-1.75487000	-2.58940200	4.28170800
H	-2.40824600	-0.22459800	4.30112700
H	-0.90776400	-0.19286000	5.25679300
Al	-0.11393600	-0.07440800	0.22966300

88

IN1 (Al)

O	0.66150100	1.59753500	0.00000000

O	1.04586600	-1.37146900	0.00000000
O	-1.72336700	-0.21764400	0.00000000
N	-0.42237100	2.51876900	0.00000000
N	2.38510000	-0.89206800	0.00000000
N	-1.96659500	-1.61977000	0.00000000
C	-0.41471400	3.25186100	-1.27977500
C	-0.72595700	2.23244600	-2.37865500
H	-0.91761100	2.74484900	-3.33322500
H	-1.61942700	1.65014700	-2.10665200
H	0.11764200	1.54529700	-2.54123700
C	0.92177800	3.93011900	-1.60957900
H	1.09581600	4.83942200	-1.02033000
H	0.94438400	4.21526000	-2.67234300
H	1.74948600	3.23259500	-1.42020700
C	-1.56333500	4.26744400	-1.23757900
H	-1.52598300	4.87048500	-2.15903400
H	-2.51471000	3.70922400	-1.25675200
C	-1.53531900	5.14547900	0.00000000
H	-2.39827300	5.83019200	0.00000000
H	-0.63791900	5.78629300	0.00000000
C	-1.56333500	4.26744400	1.23757900
H	-2.51471000	3.70922400	1.25675200
H	-1.52598300	4.87048500	2.15903400
C	-0.41471400	3.25186100	1.27977500
C	0.92177800	3.93011900	1.60957900
H	1.09581600	4.83942200	1.02033000
H	1.74948600	3.23259500	1.42020700
H	0.94438400	4.21526000	2.67234300
C	-0.72595700	2.23244600	2.37865500
H	0.11764200	1.54529700	2.54123700
H	-1.61942700	1.65014700	2.10665200
H	-0.91761100	2.74484900	3.33322500
C	3.01662800	-1.26464000	-1.27976000
C	2.93297000	-2.76082600	-1.61041200
H	3.16829200	-2.92336500	-2.67316700
H	1.91417100	-3.12632000	-1.42070100
H	3.63198000	-3.36772300	-1.02099800
C	2.29203800	-0.48321200	-2.37880500
H	2.24180700	0.58266000	-2.10949300
H	1.27242100	-0.86412200	-2.53854200
H	2.82908500	-0.57926100	-3.33423900

C	4.47135600	-0.78013400	-1.23728600
H	4.97413900	-1.11465400	-2.15893000
H	4.46577700	0.32298000	-1.25628300
C	5.21731700	-1.24464400	0.00000000
H	6.24219900	-0.84072200	0.00000000
H	5.32264600	-2.34229400	0.00000000
C	4.47135600	-0.78013400	1.23728600
H	4.46577700	0.32298000	1.25628300
H	4.97413900	-1.11465400	2.15893000
C	3.01662800	-1.26464000	1.27976000
C	2.93297000	-2.76082600	1.61041200
H	3.63198000	-3.36772300	1.02099800
H	1.91417100	-3.12632000	1.42070100
H	3.16829200	-2.92336500	2.67316700
C	2.29203800	-0.48321200	2.37880500
H	2.24180700	0.58266000	2.10949300
H	2.82908500	-0.57926100	3.33423900
H	1.27242100	-0.86412200	2.53854200
C	-2.60112000	-1.98653700	-1.28004000
C	-3.86111000	-1.17621500	-1.61214100
H	-3.67533200	-0.10901900	-1.42765500
H	-4.73321600	-1.48157500	-1.02008500
H	-4.12013400	-1.30527000	-2.67396800
C	-1.56286300	-1.74231400	-2.37814100
H	-1.39099700	-0.66755700	-2.53864700
H	-1.90996300	-2.16275000	-3.33377200
H	-0.61147600	-2.22456800	-2.10695700
C	-2.89691700	-3.49098600	-1.23711200
H	-3.43542800	-3.76367800	-2.15893200
H	-1.93453400	-4.03019600	-1.25553100
C	-3.66920000	-3.91067400	0.00000000
H	-3.82330300	-5.00143800	0.00000000
H	-4.67598200	-3.46088200	0.00000000
C	-2.89691700	-3.49098600	1.23711200
H	-1.93453400	-4.03019600	1.25553100
H	-3.43542800	-3.76367800	2.15893200
C	-2.60112000	-1.98653700	1.28004000
C	-3.86111000	-1.17621500	1.61214100
H	-4.12013400	-1.30527000	2.67396800
H	-4.73321600	-1.48157500	1.02008500
H	-3.67533200	-0.10901900	1.42765500

C	-1.56286300	-1.74231400	2.37814100
H	-0.61147600	-2.22456800	2.10695700
H	-1.90996300	-2.16275000	3.33377200
H	-1.39099700	-0.66755700	2.53864700
Al	-0.00680900	0.00170500	0.00000000

90

IN2 (Al)

O	-1.40541800	-1.02300600	-0.05322300
O	1.56773400	-0.69656100	-0.03048200
O	-0.19340800	1.72445400	-0.04518000
N	-2.55783400	-0.18885600	-0.03451700
N	1.43501300	-2.11173000	-0.03618300
N	1.10901000	2.29634200	-0.03328900
C	-3.26515600	-0.39661800	1.24297300
C	-2.34751900	0.12900300	2.34936700
H	-2.88432400	0.16543100	3.30904700
H	-2.00566300	1.14501000	2.09985100
H	-1.47295200	-0.52257700	2.49161800
C	-3.60123500	-1.86331400	1.54464100
H	-4.44375700	-2.23913400	0.95019600
H	-3.86918600	-1.97525000	2.60620800
H	-2.72541100	-2.49458100	1.33945800
C	-4.52664700	0.47560400	1.22130200
H	-5.10121400	0.27518100	2.13990600
H	-4.21303600	1.53260400	1.26243000
C	-5.37486600	0.26448600	-0.01933000
H	-6.24576100	0.93889700	-0.00360100
H	-5.78318700	-0.75961400	-0.03999900
C	-4.53115000	0.52730400	-1.25317800
H	-4.21633800	1.58473700	-1.25086000
H	-5.10948600	0.36627100	-2.17714400
C	-3.27080700	-0.34456800	-1.31617900
C	-3.61078700	-1.79723500	-1.67522500
H	-4.44875500	-2.19695400	-1.09008700
H	-2.73401200	-2.43695000	-1.50358300
H	-3.88723400	-1.86498400	-2.73835200
C	-2.35785800	0.22414900	-2.40542700
H	-1.48736600	-0.42560500	-2.58086500
H	-2.00888300	1.22647500	-2.11423000
H	-2.90221500	0.30371300	-3.35823400

C	1.96371200	-2.63523200	1.23718000
C	3.39284200	-2.17910700	1.56073500
H	3.61759600	-2.37510500	2.62014300
H	3.48806700	-1.09941400	1.37983500
H	4.15200000	-2.69715100	0.96100600
C	1.03250300	-2.13424300	2.34401700
H	-0.01317900	-2.35753600	2.08398200
H	1.14209000	-1.05066300	2.49883200
H	1.27069500	-2.62786100	3.29818700
C	1.86087600	-4.16482700	1.18907000
H	2.31792400	-4.57117200	2.10563600
H	0.79210800	-4.43731800	1.21454600
C	2.48927200	-4.76368300	-0.05564600
H	2.35793100	-5.85742200	-0.05923400
H	3.57777600	-4.58759200	-0.06351500
C	1.84125000	-4.15427000	-1.28510200
H	0.77208100	-4.42614600	-1.29583500
H	2.28348800	-4.55315100	-2.21214100
C	1.94387100	-2.62432800	-1.32235800
C	3.36824800	-2.16619300	-1.66336900
H	4.13620300	-2.69096300	-1.08089600
H	3.46726600	-1.08850600	-1.47274700
H	3.57630100	-2.35133200	-2.72812400
C	0.99583200	-2.11488500	-2.41116500
H	-0.04609900	-2.33368700	-2.13262400
H	1.21484100	-2.60709100	-3.37048000
H	1.10818600	-1.03176700	-2.56887700
C	1.30092300	2.99957100	1.24863200
C	0.21377900	4.03460300	1.56851200
H	-0.77779300	3.60654200	1.36542500
H	0.31718600	4.95739700	0.98362500
H	0.26140500	4.31049500	2.63288800
C	1.29292300	1.93357100	2.34704200
H	0.28557800	1.51921600	2.49964200
H	1.61362300	2.36773600	3.30573800
H	1.98319400	1.11820700	2.08251400
C	2.69466100	3.63942500	1.21982100
H	2.82457600	4.22723200	2.14271600
H	3.44358600	2.82968000	1.24585400
C	2.93450400	4.48825500	-0.01530000
H	3.95951900	4.89174700	-0.00585000

H	2.26321400	5.36306100	-0.02084300
C	2.71685300	3.64088000	-1.25555200
H	3.46552500	2.83058000	-1.26883700
H	2.86386100	4.22970500	-2.17523600
C	1.32328600	3.00231800	-1.31000700
C	0.24324500	4.03967800	-1.64581900
H	0.31008300	4.31918400	-2.70821000
H	0.33722900	4.96020400	-1.05588100
H	-0.75225000	3.61226100	-1.46190600
C	1.33289800	1.93926500	-2.41148900
H	2.01707500	1.12204800	-2.13737600
H	1.67137300	2.37672900	-3.36257000
H	0.32721500	1.52698000	-2.58157900
H	-0.13344600	0.17453200	5.30057900
H	0.23221500	-0.28236200	4.81388900
Al	-0.01359800	0.00407800	-0.04029900

90

TS1 (Al)

O	-0.54943800	-2.00622200	-0.81161100
O	1.47754800	-0.40989400	0.57051700
O	-1.05789400	1.02391100	-0.04738900
N	-1.81780400	-1.73149800	-0.18814800
N	2.58531200	-0.72616500	-0.25378000
N	-0.52956200	2.32507600	0.04087300
C	-1.96821200	-2.52097200	1.06882800
C	-0.92059100	-1.97751500	2.04074000
H	-1.06683400	-2.38909400	3.04941200
H	-1.02961200	-0.88073800	2.14284400
H	0.10884900	-2.21924800	1.74053900
C	-1.74013200	-4.02143300	0.88463500
H	-2.60444700	-4.53222900	0.44395600
H	-1.54187400	-4.48820900	1.86069800
H	-0.86832500	-4.18633900	0.23693200
C	-3.34808800	-2.20406000	1.65991400
H	-3.48699800	-2.83450400	2.55261500
H	-3.33908100	-1.15664300	2.00802200
C	-4.48677500	-2.37558400	0.67353300
H	-5.44003100	-2.09466600	1.14788600
H	-4.59249900	-3.43293300	0.38010200
C	-4.22701300	-1.50479900	-0.54144900

H	-4.22088700	-0.44551800	-0.23217600
H	-5.02531200	-1.61497900	-1.29228800
C	-2.88867500	-1.81009600	-1.22114200
C	-2.90666400	-3.16693700	-1.93224300
H	-3.34974900	-3.96955100	-1.33069700
H	-1.88167300	-3.45462800	-2.20254300
H	-3.49714900	-3.08306100	-2.85631900
C	-2.61173100	-0.73033500	-2.26242700
H	-1.70119700	-0.95673300	-2.83498000
H	-2.49310000	0.24359300	-1.77114500
H	-3.45356200	-0.67299600	-2.96771300
C	3.20119900	-1.98970500	0.21594200
C	3.53671300	-2.00519100	1.71400000
H	3.70850000	-3.04009800	2.04693400
H	2.69354600	-1.59245900	2.28516500
H	4.43485700	-1.42595200	1.96125200
C	2.22683100	-3.13341100	-0.06453300
H	1.85223400	-3.08625500	-1.09581500
H	1.35787000	-3.10879600	0.60222500
H	2.73769300	-4.09675400	0.08597400
C	4.45068400	-2.25315600	-0.63792900
H	4.94045200	-3.16109300	-0.25043400
H	4.11841600	-2.48538800	-1.66422800
C	5.41429000	-1.08460300	-0.68985900
H	6.26150500	-1.31802600	-1.35451900
H	5.85061600	-0.89571100	0.30506600
C	4.67102200	0.13826000	-1.18910900
H	4.33479000	-0.04490900	-2.22401200
H	5.32529200	1.02461600	-1.21890600
C	3.44186500	0.48300400	-0.33918400
C	3.86429900	1.05731700	1.02160200
H	4.70268800	0.51525700	1.47701800
H	3.01669700	1.02718000	1.71782600
H	4.17651000	2.10586700	0.90256000
C	2.63874400	1.54679400	-1.08185000
H	2.29839400	1.14918500	-2.04957000
H	3.26952500	2.42740800	-1.27971800
H	1.75957200	1.87681500	-0.50797600
C	-0.71871800	2.82487300	1.42110000
C	-2.16704000	2.76603000	1.93007800
H	-2.61283700	1.79742500	1.66198400

H	-2.80263000	3.56288500	1.52513800
H	-2.17928600	2.86017400	3.02665400
C	0.13838300	1.96747800	2.35212900
H	-0.29719500	0.97119300	2.50911600
H	0.20936200	2.44739700	3.33977400
H	1.14711200	1.83598500	1.94199500
C	-0.16280600	4.25435400	1.47158600
H	-0.34472100	4.65956600	2.48006100
H	0.93153900	4.19776200	1.34221500
C	-0.73890900	5.16198000	0.40141400
H	-0.26734900	6.15645100	0.45124800
H	-1.81539900	5.32760200	0.57409800
C	-0.50773300	4.53856300	-0.96226800
H	0.57767600	4.48684900	-1.15321000
H	-0.94480200	5.15549400	-1.76403700
C	-1.07954200	3.11985700	-1.08076800
C	-2.61530900	3.15029500	-1.15013000
H	-2.93735600	3.45653300	-2.15680700
H	-3.06367500	3.85161500	-0.43576500
H	-3.02295600	2.15053800	-0.94951400
C	-0.55766400	2.51380100	-2.38493200
H	0.53696200	2.44493000	-2.37006200
H	-0.85300400	3.14981700	-3.23284100
H	-0.96906600	1.51148100	-2.55947200
H	1.68209700	-1.06726200	-2.60004500
H	1.31484100	-1.11374100	-3.27130100
Al	-0.14637700	-0.45755500	-0.07415600

IN3 (Al)

O	-0.56238500	1.37798900	-0.23908700
N	0.34291500	2.43134800	-0.03161900
C	0.16249800	3.42554000	-1.11489700
C	0.20135800	2.88616200	1.37142300
C	1.10969100	4.59957600	-0.83938300
C	-1.28259600	3.91394600	-1.29703200
C	0.59504700	2.76301100	-2.42378900
C	1.13023500	4.08909500	1.57923600
C	-1.24372100	3.20970600	1.78082800
C	0.70079700	1.75549000	2.27030500
C	0.93702500	5.18580600	0.54940300
H	2.14729700	4.23880700	-0.94929200

H	0.95296000	5.36281900	-1.61893300
H	-1.39334600	4.39356800	-2.28156500
H	-1.96642900	3.05624800	-1.25120000
H	-1.59135700	4.64412400	-0.53894800
H	-0.10460000	1.97096300	-2.72310000
H	0.62722500	3.51158500	-3.23016100
H	1.59453200	2.31774300	-2.31982100
H	0.98138900	4.46878200	2.60322800
H	2.17251500	3.72956100	1.52192900
H	-1.91156200	2.42558200	1.39700000
H	-1.32561500	3.23395800	2.87825100
H	-1.59168100	4.17982300	1.40427200
H	0.70608900	2.08474500	3.32070400
H	0.06087200	0.86841700	2.19027600
H	1.72194500	1.46710500	1.98877400
H	-0.06138100	5.64202000	0.65367000
H	1.66195700	5.99902700	0.71583300
O	-1.35389800	-1.38904900	-0.58592700
N	-2.58965800	-0.98740500	-0.05284400
C	-3.58211900	-0.94188600	-1.14014400
C	-2.86495700	-1.77313800	1.16019000
C	-4.91813200	-0.50481100	-0.52709200
C	-3.72913500	-2.25539100	-1.92369900
C	-3.12333700	0.14406800	-2.11701200
C	-4.22595600	-1.32370400	1.70735100
C	-2.82333000	-3.29628200	0.95795200
C	-1.79337900	-1.39804700	2.18595500
C	-5.32736700	-1.35456100	0.66291800
H	-4.81576400	0.54394000	-0.19936500
H	-5.68869600	-0.52279200	-1.31511300
H	-4.24426100	-2.06972000	-2.87893500
H	-2.73024600	-2.65874500	-2.14084500
H	-4.30346900	-3.01869700	-1.38254900
H	-2.24791000	-0.18545400	-2.69666400
H	-3.92816600	0.38227000	-2.82930700
H	-2.84664500	1.05126800	-1.56157800
H	-4.47981300	-1.95277400	2.57634500
H	-4.11834600	-0.29039200	2.07878500
H	-1.93560800	-3.56530100	0.36902700
H	-2.76173400	-3.80634200	1.93176400
H	-3.70925200	-3.68290000	0.43821600

H	-2.00689500	-1.87321100	3.15576200
H	-0.79642100	-1.71968500	1.85280100
H	-1.77613500	-0.30738800	2.32464100
H	-5.52812800	-2.39138400	0.34453500
H	-6.27069200	-0.98095200	1.09324300
O	1.34091900	-0.85936400	0.37504300
N	2.39874600	-1.40327400	-0.28509900
C	3.67411900	-0.60292600	0.00081000
C	2.41918900	-2.92656100	-0.11368900
C	4.83944900	-1.28981400	-0.71557800
C	3.90025500	-0.48747700	1.50151500
C	3.43944800	0.77728200	-0.60305700
C	3.66359100	-3.46716400	-0.82231400
C	2.37337500	-3.29333300	1.36196900
C	1.16611900	-3.44559800	-0.80955200
C	4.95259100	-2.77427600	-0.42006600
H	4.72169600	-1.14697900	-1.80418400
H	5.75729000	-0.75081600	-0.43436000
H	4.58828300	0.34943300	1.68483300
H	2.95298900	-0.27410700	2.01109700
H	4.34776900	-1.38665800	1.94114700
H	2.58704900	1.30247300	-0.14459900
H	4.34637000	1.38241200	-0.46270600
H	3.25050700	0.70538300	-1.68558700
H	3.70750600	-4.54910800	-0.62409600
H	3.52295500	-3.36100300	-1.91253600
H	1.58745300	-2.71916900	1.86873800
H	2.12804900	-4.36148000	1.44216400
H	3.32502100	-3.13054200	1.88058300
H	1.17060400	-4.54377000	-0.76809900
H	0.24546000	-3.07558400	-0.33729400
H	1.14431300	-3.14972800	-1.86983600
H	5.17372000	-2.94659300	0.64540900
H	5.79772300	-3.20332700	-0.97911500
H	0.77863000	-0.45634700	-2.10054800
H	2.20807400	-1.24962700	-1.30256400
Al	-0.03075000	-0.23007900	-0.70144200

101

INO (Ga)

Ga	-0.00882500	-0.09628300	0.15803000

O	-1.51388800	-0.14524100	-0.87401600
O	0.85838200	1.48250200	0.41947800
O	1.07515000	-1.56298400	-0.04120900
N	-2.64187200	-0.86037900	-0.43168800
N	0.30906600	2.68733500	-0.07489900
N	2.43335300	-1.34364500	-0.34485200
C	-2.88641800	-1.97564900	-1.37759800
C	-1.73270100	-2.96796700	-1.23047700
H	-1.88782700	-3.82778000	-1.89942500
H	-1.67410500	-3.34581000	-0.20062800
H	-0.76824400	-2.50561800	-1.47531500
C	-2.95145100	-1.54700400	-2.85140000
H	-3.89331700	-1.05056000	-3.11559500
H	-2.85092200	-2.42919100	-3.50144000
H	-2.12342700	-0.85892000	-3.06966100
C	-4.17650600	-2.68588900	-0.94691800
H	-4.39660400	-3.47825200	-1.68061900
H	-3.98464100	-3.19072400	0.01603600
C	-5.35547100	-1.74640200	-0.77630700
H	-6.23908400	-2.30215800	-0.42335800
H	-5.64102700	-1.30410100	-1.74501500
C	-4.98485600	-0.65846900	0.21465500
H	-4.79829400	-1.12099400	1.19979000
H	-5.80982700	0.05953400	0.35074500
C	-3.72882400	0.11851600	-0.19588100
C	-4.02027300	1.04893400	-1.38326300
H	-4.58218900	0.56033300	-2.18841300
H	-3.07525800	1.42488600	-1.79694100
H	-4.61448600	1.91041500	-1.04270700
C	-3.29575600	0.99509500	0.97862000
H	-2.41489800	1.59616300	0.70910000
H	-3.05450100	0.36765800	1.84777400
H	-4.10716500	1.68315200	1.25973500
C	0.79597800	2.92295500	-1.45668700
C	2.32076800	2.83742600	-1.61848600
H	2.57477500	2.74125900	-2.68532400
H	2.69641000	1.94768800	-1.09410200
H	2.84832000	3.71917300	-1.23409400
C	0.16666700	1.86269100	-2.35710000
H	-0.91530300	1.79846300	-2.18791300
H	0.59845700	0.86770800	-2.18568500

H	0.35632500	2.10652700	-3.41325700
C	0.25449400	4.28123400	-1.92344600
H	0.65797200	4.48321000	-2.92877500
H	-0.84004300	4.19047400	-2.03091900
C	0.55844800	5.41660200	-0.96668900
H	0.10504200	6.35364600	-1.32789200
H	1.64431400	5.60209300	-0.91672900
C	0.01539900	5.06037900	0.40321900
H	-1.08340100	4.97698400	0.34232300
H	0.23725900	5.84814500	1.14139200
C	0.56449900	3.73306200	0.94017100
C	2.03747100	3.87486000	1.35728600
H	2.64563000	4.41226800	0.61946600
H	2.47562800	2.87899100	1.50861200
H	2.10406800	4.43080700	2.30509200
C	-0.23340800	3.36944000	2.19048500
H	-1.27111200	3.12009800	1.92459700
H	-0.24480000	4.21904700	2.89012300
H	0.22154900	2.51421000	2.70242000
C	2.75326300	-2.07120200	-1.58998600
C	2.39940400	-3.56628800	-1.55803800
H	1.39887100	-3.69834200	-1.12409900
H	3.11201700	-4.16432200	-0.97618100
H	2.38829600	-3.97163800	-2.58121400
C	1.95637600	-1.41902300	-2.71938300
H	0.87482600	-1.54735900	-2.57516700
H	2.22837500	-1.87159200	-3.68489200
H	2.18033800	-0.34430100	-2.76654400
C	4.24278900	-1.85602500	-1.88982500
H	4.50623600	-2.44772800	-2.78157700
H	4.38553800	-0.79481300	-2.15589200
C	5.14725200	-2.18958100	-0.71841800
H	6.19640900	-1.96781400	-0.97184600
H	5.11009800	-3.26900700	-0.49528900
C	4.71541500	-1.38184200	0.49137200
H	4.85474200	-0.30872400	0.27603700
H	5.33554600	-1.61611600	1.37196400
C	3.24395600	-1.60255700	0.86265100
C	3.03711100	-2.98990000	1.49139200
H	3.42184200	-2.99391300	2.52300100
H	3.54884400	-3.79345000	0.94735300

H	1.96436700	-3.22600900	1.52238000
C	2.85524400	-0.55443000	1.90628400
H	2.81559700	0.44651900	1.45377100
H	3.58211100	-0.55517200	2.73267900
H	1.86691900	-0.77687100	2.32998200
O	-0.66238400	-0.68175900	1.99285200
C	-0.66900400	0.04149000	3.23936900
C	-1.22323900	-0.94551100	4.24961600
H	-1.28123900	0.94178100	3.11415600
H	0.36563900	0.33954700	3.46834000
C	-0.97561900	-2.08041700	2.19631000
C	-0.77782200	-2.28845700	3.68113300
H	-0.29672800	-2.65520300	1.55382100
H	-2.01496400	-2.23069700	1.86758800
H	0.28491900	-2.47349900	3.90507300
H	-1.36091100	-3.13817100	4.06152100
H	-2.32285900	-0.88784800	4.27690600
H	-0.84753400	-0.74905500	5.26307000

88

IN1 (Ga)

Ga	-0.00247700	-0.00604200	-0.00011100
O	0.40516100	-1.75571300	-0.00013700
O	-1.71974000	0.52044800	-0.00087500
O	1.30981300	1.22054300	0.00045500
N	1.82182500	-1.86998500	0.00044000
N	-2.53731400	-0.64135100	0.00002200
N	0.70899000	2.50816300	-0.00002500
C	2.24403800	-2.46377300	-1.28075900
C	1.89948000	-1.45066600	-2.37620600
H	2.35743000	-1.74981000	-3.33086200
H	2.28135800	-0.45576200	-2.10019100
H	0.81346100	-1.38717200	-2.53941600
C	1.55851800	-3.79544300	-1.61544900
H	1.94510800	-4.63482200	-1.02344900
H	1.71335600	-4.04000800	-2.67729200
H	0.47805400	-3.71344800	-1.43312800
C	3.76983700	-2.61518900	-1.23680600
H	4.09369500	-3.12534900	-2.15820100
H	4.21454500	-1.60580700	-1.25424100
C	4.25968400	-3.34419500	0.00111600

H	5.36012600	-3.39470100	0.00159100
H	3.90711800	-4.38899500	0.00066900
C	3.76873000	-2.61587100	1.23900400
H	4.21337800	-1.60648000	1.25738600
H	4.09178000	-3.12652800	2.16040900
C	2.24288500	-2.46456600	1.28165100
C	1.55722100	-3.79655800	1.61481800
H	1.94509000	-4.63560700	1.02318100
H	0.47703200	-3.71493800	1.43074500
H	1.71042700	-4.04144000	2.67682500
C	1.89724300	-1.45213500	2.37738400
H	0.81107100	-1.38870700	2.53957900
H	2.27938700	-0.45706500	2.10232700
H	2.35428700	-1.75184000	3.33229800
C	-3.26267200	-0.70386800	-1.28163400
C	-4.05922000	0.56394400	-1.61879700
H	-4.35205500	0.55082900	-2.67963600
H	-3.43724600	1.45261100	-1.44261500
H	-4.97644200	0.66201100	-1.02417900
C	-2.21488700	-0.92485100	-2.37641200
H	-1.55458500	-1.76123100	-2.10029500
H	-1.60581500	-0.02342700	-2.53935500
H	-2.70542800	-1.16579800	-3.33134700
C	-4.17033100	-1.93968800	-1.23729600
H	-4.77414700	-1.95860700	-2.15881200
H	-3.52883700	-2.83715600	-1.25463100
C	-5.04778800	-1.98909200	0.00034200
H	-5.65198700	-2.91021100	0.00111800
H	-5.76708400	-1.15326300	-0.00099800
C	-4.17156000	-1.93680700	1.23873500
H	-3.53010300	-2.83424600	1.25876700
H	-4.77628800	-1.95358600	2.15969500
C	-3.26390900	-0.70092300	1.28111100
C	-4.06069800	0.56772600	1.61457300
H	-4.97733100	0.66447000	1.01882400
H	-3.43849600	1.45594600	1.43692800
H	-4.35459000	0.55709400	2.67514700
C	-2.21724200	-0.91948100	2.37744400
H	-1.55721000	-1.75705400	2.10433300
H	-2.70879700	-1.15750700	3.33259000
H	-1.60774300	-0.01801700	2.53842300

C	1.01997900	3.16766600	-1.28124700
C	2.51685400	3.22345000	-1.61537300
H	2.97432000	2.24039300	-1.43670700
H	3.05952200	3.96936300	-1.02073800
H	2.65408700	3.48202200	-2.67631900
C	0.30756700	2.37000500	-2.37720300
H	0.78636900	1.39333300	-2.54112300
H	0.34263800	2.91638900	-3.33157600
H	-0.74616200	2.21282300	-2.10094300
C	0.40308000	4.57143200	-1.23832600
H	0.68917300	5.10336100	-2.15990800
H	-0.69479800	4.46404900	-1.25628100
C	0.79764000	5.35649400	-0.00069300
H	0.30252800	6.34055400	-0.00105400
H	1.88126200	5.56077300	-0.00047300
C	0.40246400	4.57203100	1.23712500
H	-0.69542500	4.46467000	1.25459500
H	0.68811100	5.10440300	2.15859000
C	1.01933900	3.16828500	1.28102600
C	2.51605200	3.22425300	1.61584700
H	2.65277700	3.48315900	2.67677700
H	3.05896300	3.97001100	1.02124000
H	2.97364100	2.24116700	1.43768900
C	0.30637900	2.37115900	2.37700400
H	-0.74725900	2.21404800	2.10036400
H	0.34113900	2.91791600	3.33117500
H	0.78492600	1.39445300	2.54148100

90

IN2 (Ga)

Ga	-0.01113500	0.00227100	-0.03884200
O	-1.51540300	-0.98033000	-0.04689800
O	1.59114400	-0.81022800	-0.02308800
O	-0.09744500	1.79684500	-0.05707100
N	-2.62259800	-0.09003000	-0.03181600
N	1.38426000	-2.21489000	-0.02979700
N	1.23024900	2.30153800	-0.02649000
C	-3.34647600	-0.26709500	1.23994000
C	-2.40800000	0.20041700	2.35552600
H	-2.95016100	0.26303100	3.31089300
H	-2.00692000	1.19702800	2.11538300

H	-1.57200100	-0.49919600	2.49940900
C	-3.76522700	-1.71473800	1.53117500
H	-4.61767900	-2.04285700	0.92274200
H	-4.05480800	-1.81514800	2.58823200
H	-2.92194000	-2.39235700	1.33768500
C	-4.55862300	0.67261500	1.21400100
H	-5.15162200	0.49759900	2.12606800
H	-4.18839600	1.71067000	1.26535400
C	-5.40602600	0.51618100	-0.03554700
H	-6.23954700	1.23631500	-0.02175500
H	-5.86870100	-0.48426900	-0.06699800
C	-4.53974100	0.74172300	-1.26139100
H	-4.16771500	1.78030800	-1.24862200
H	-5.11909100	0.61916200	-2.19061900
C	-3.32816000	-0.19711800	-1.32159600
C	-3.74438700	-1.62573100	-1.69733400
H	-4.60492400	-1.98568600	-1.11908200
H	-2.90432100	-2.31429700	-1.53062500
H	-4.02011000	-1.66726900	-2.76202600
C	-2.37363600	0.32955800	-2.39715800
H	-1.54261300	-0.36905000	-2.57575500
H	-1.96574900	1.30519300	-2.09136100
H	-2.90610800	0.45469300	-3.35183000
C	1.87508400	-2.76475500	1.24657400
C	3.32601100	-2.39044800	1.57860900
H	3.53558400	-2.60670600	2.63721400
H	3.48200400	-1.31621200	1.40733000
H	4.05757200	-2.94507800	0.97719600
C	0.96653700	-2.21051900	2.34747500
H	-0.08892300	-2.37027900	2.07921800
H	1.13866200	-1.13555700	2.50470100
H	1.16727000	-2.71998100	3.30194500
C	1.68673000	-4.28626200	1.19651500
H	2.11743200	-4.71871400	2.11389700
H	0.60426300	-4.49824100	1.21825600
C	2.28443900	-4.91821100	-0.04720300
H	2.09273200	-6.00299700	-0.05167200
H	3.38106600	-4.80281100	-0.05177200
C	1.67512500	-4.27391200	-1.27892400
H	0.59235900	-4.48502800	-1.29246500
H	2.09686500	-4.69767400	-2.20448000

C	1.86392500	-2.75207800	-1.31610600
C	3.31247000	-2.37595700	-1.65631500
H	4.04846800	-2.93870500	-1.06797200
H	3.47171500	-1.30407000	-1.47363100
H	3.51244300	-2.57985700	-2.71922000
C	0.94647700	-2.18741500	-2.40456600
H	-0.10683200	-2.34175500	-2.12455700
H	1.13308500	-2.69509200	-3.36270000
H	1.12355500	-1.11344500	-2.56404500
C	1.43558300	2.99932900	1.25549900
C	0.39789300	4.09024100	1.55388900
H	-0.61199400	3.71016300	1.34530200
H	0.55300500	5.00062700	0.96090300
H	0.44858200	4.37445600	2.61596000
C	1.35523300	1.93934700	2.35741400
H	0.32572000	1.57874500	2.49671500
H	1.68638100	2.36004800	3.31861200
H	2.00502600	1.08721600	2.10572900
C	2.86030000	3.56784500	1.24601400
H	3.00455400	4.15548800	2.16688600
H	3.56677000	2.72157400	1.29021200
C	3.16325100	4.39397200	0.00917600
H	4.20674700	4.74618700	0.03404400
H	2.53653100	5.30097500	-0.01416800
C	2.92544400	3.54928000	-1.22940100
H	3.63196900	2.70191400	-1.22335900
H	3.11901400	4.12296000	-2.15003800
C	1.50226500	2.98188100	-1.30531700
C	0.48405200	4.07059700	-1.67098200
H	0.59208800	4.34302400	-2.73184300
H	0.60877700	4.98706000	-1.08032200
H	-0.53633500	3.69458100	-1.51280800
C	1.47711700	1.90808800	-2.39670400
H	2.11191800	1.05858900	-2.10207100
H	1.85702900	2.31833300	-3.34430700
H	0.45486100	1.54656800	-2.58235000
H	-0.14630300	0.17766900	5.26298800
H	0.19825400	-0.28588000	4.76728100

90

TS1 (Ga)

Ga	0.13398500	-0.17649000	-0.03556400
O	1.39522500	-1.35829400	0.48033700
O	-1.47121600	-0.74253200	-0.63563100
O	0.62163900	1.54974000	-0.19190200
N	2.64634300	-0.78471500	0.13179300
N	-2.17301300	-1.64783500	0.19678600
N	-0.48540400	2.42745800	-0.07958500
C	3.07569800	-1.33218400	-1.16961900
C	2.06790100	-0.83535300	-2.21239100
H	2.43202400	-1.03476800	-3.23093800
H	1.92154200	0.25198200	-2.10922300
H	1.09624500	-1.35285200	-2.13040500
C	3.12716700	-2.86423100	-1.23256400
H	3.99704000	-3.28133900	-0.70992100
H	3.18091000	-3.19573200	-2.28073000
H	2.21854100	-3.28604900	-0.78212500
C	4.43875200	-0.71079000	-1.50042100
H	4.80668500	-1.15902100	-2.43736100
H	4.28790400	0.36472400	-1.69458500
C	5.44775700	-0.86948900	-0.37743800
H	6.39617900	-0.37907200	-0.64842800
H	5.68940400	-1.93412500	-0.22222800
C	4.88664900	-0.25837500	0.89383100
H	4.74898000	0.82492000	0.73726500
H	5.58631900	-0.37445200	1.73707300
C	3.53428300	-0.85476000	1.30439000
C	3.69653900	-2.26779100	1.88162700
H	4.34153400	-2.90898300	1.26734000
H	2.71259600	-2.74894900	1.96951000
H	4.14465000	-2.21217800	2.88523100
C	2.92220800	0.04289100	2.38041200
H	1.98336600	-0.38171200	2.76261400
H	2.71534400	1.03890100	1.96238800
H	3.61779600	0.15080900	3.22621700
C	-2.20370500	-2.97632900	-0.45537500
C	-2.75865600	-2.96791500	-1.88700200
H	-2.47286500	-3.89883500	-2.39961300
H	-2.33165000	-2.12334500	-2.44540300
H	-3.85214700	-2.89345300	-1.92830500
C	-0.76861000	-3.49933100	-0.51297200
H	-0.26737300	-3.39421700	0.45918200

H	-0.17391100	-2.95905700	-1.26134500
H	-0.77208200	-4.56240200	-0.79722800
C	-3.01261300	-3.91040900	0.45445000
H	-3.09312800	-4.88981400	-0.04403000
H	-2.43464700	-4.07034700	1.38068000
C	-4.38074000	-3.36152100	0.81503200
H	-4.89644900	-4.04886100	1.50440700
H	-5.01898000	-3.29516300	-0.08165600
C	-4.22168900	-1.99568400	1.45710700
H	-3.67311700	-2.10871800	2.40774400
H	-5.19959000	-1.55177900	1.70391300
C	-3.45250500	-1.00350300	0.57617600
C	-4.30836000	-0.53432900	-0.60957600
H	-4.82611200	-1.35393800	-1.12219400
H	-3.68058000	-0.00913900	-1.34187300
H	-5.07683000	0.16780200	-0.25265000
C	-3.10684400	0.22472800	1.41634000
H	-2.46521600	-0.05973800	2.26331000
H	-4.02493800	0.67489700	1.82306500
H	-2.58717300	0.98599900	0.81567000
C	-0.73534000	3.04630700	-1.39872900
C	0.47605100	3.77023000	-2.00484600
H	1.37680400	3.15143200	-1.88764500
H	0.66816200	4.74989600	-1.55057600
H	0.30878100	3.93345600	-3.08029500
C	-1.14318500	1.92976000	-2.36208900
H	-0.28129000	1.30733000	-2.64465500
H	-1.54894000	2.36522400	-3.28747700
H	-1.90347600	1.27949200	-1.90812300
C	-1.92931600	3.99413700	-1.22899200
H	-2.10174300	4.51032700	-2.18717600
H	-2.82506700	3.38148200	-1.02891600
C	-1.74429100	4.98702700	-0.09517000
H	-2.64565200	5.61091200	0.01454400
H	-0.91995500	5.68293800	-0.32365800
C	-1.46500100	4.23998400	1.19684300
H	-2.35178300	3.63895700	1.46137700
H	-1.29059200	4.93740700	2.03202200
C	-0.26192300	3.29352900	1.09828900
C	1.06515000	4.06639200	1.07725100
H	1.28875100	4.44553000	2.08586600

H	1.05352500	4.92767300	0.39831400
H	1.88062600	3.39502500	0.77389000
C	-0.25655700	2.39556900	2.33716100
H	-1.15293200	1.76187000	2.36151000
H	-0.25033900	3.01327000	3.24762300
H	0.63501000	1.75518200	2.36351900
H	-0.90820200	-1.76606600	2.33699600
H	-0.42906200	-1.64916700	2.92424300

90

IN3 (Ga)

Ga	-0.06609200	-0.19222800	-0.71748400
O	-0.61724200	1.45927700	-0.14502900
N	0.38417400	2.43591200	0.01237700
C	0.24579400	3.43729200	-1.07028400
C	0.33982500	2.90821500	1.41591600
C	1.30303800	4.52511800	-0.84325300
C	-1.15842100	4.04748600	-1.19824700
C	0.56527300	2.73828200	-2.39290200
C	1.38420700	4.02013600	1.57636900
C	-1.05051800	3.37163200	1.87837500
C	0.76027900	1.74017900	2.30665200
C	1.24593700	5.12701900	0.54830400
H	2.29903000	4.07446300	-0.99761000
H	1.17892000	5.29804200	-1.61925200
H	-1.26873000	4.52875600	-2.18210000
H	-1.91050400	3.25141700	-1.11603800
H	-1.37147000	4.80651100	-0.43558400
H	-0.21908700	2.01947200	-2.66657900
H	0.63799000	3.48219100	-3.20089600
H	1.52137800	2.20013600	-2.32598800
H	1.31531100	4.41544300	2.60298600
H	2.38554000	3.56578800	1.47694800
H	-1.80517000	2.65143100	1.53267100
H	-1.08396800	3.41191500	2.97789900
H	-1.32093300	4.36744900	1.50548900
H	0.85003600	2.07780200	3.35058700
H	0.02715600	0.92548900	2.26742300
H	1.72839500	1.33903800	1.98152100
H	0.29805100	5.67137900	0.69329200
H	2.04752900	5.87231200	0.67707800

O	-1.44430500	-1.40750900	-0.59990700
N	-2.63287300	-0.93754500	-0.01485900
C	-3.66174600	-0.81574600	-1.06120400
C	-2.90621500	-1.71740200	1.20225000
C	-4.95181500	-0.32412300	-0.39306500
C	-3.90544200	-2.10388900	-1.86265300
C	-3.18519400	0.26883300	-2.03171400
C	-4.22393300	-1.20945600	1.80180600
C	-2.94624800	-3.23862400	0.98643900
C	-1.78363700	-1.40113600	2.19246500
C	-5.36132500	-1.17276200	0.79728700
H	-4.78630000	0.71231800	-0.05232300
H	-5.74906900	-0.28981200	-1.15358200
H	-4.45002000	-1.87518000	-2.79192800
H	-2.93753000	-2.55183200	-2.12745200
H	-4.49378900	-2.84859500	-1.31047200
H	-2.34124000	-0.08568800	-2.64230500
H	-3.99879900	0.55230600	-2.71710300
H	-2.85822200	1.15462500	-1.46797800
H	-4.47718000	-1.83686300	2.67220800
H	-4.05339900	-0.18760400	2.18137200
H	-2.08822600	-3.54495600	0.37225500
H	-2.88592000	-3.76078600	1.95395900
H	-3.86352600	-3.57478100	0.48589700
H	-1.98714400	-1.87006000	3.16754400
H	-0.81420800	-1.76542400	1.82480900
H	-1.71188400	-0.31276800	2.33201800
H	-5.62210300	-2.19379800	0.47143000
H	-6.27038200	-0.76163700	1.26554500
O	1.33554100	-0.89472000	0.45463000
N	2.36803700	-1.45629800	-0.22684600
C	3.67129600	-0.69733400	0.05390500
C	2.34831300	-2.98354500	-0.08166900
C	4.81086700	-1.40875700	-0.67969900
C	3.91431100	-0.60506500	1.55340900
C	3.46932400	0.69511500	-0.53421500
C	3.57212700	-3.54965300	-0.80595200
C	2.30100900	-3.37090600	1.38846700
C	1.07620200	-3.45298200	-0.77812300
C	4.88298400	-2.89943100	-0.40294800
H	4.68936000	-1.24999400	-1.76578200

H	5.74631500	-0.89995100	-0.39998400
H	4.62928100	0.20814400	1.74032200
H	2.97809000	-0.37013100	2.07379700
H	4.33769100	-1.52292400	1.97829300
H	2.62550100	1.23071300	-0.07321700
H	4.38813400	1.27974100	-0.38493500
H	3.28277600	0.64051000	-1.61845300
H	3.58657400	-4.63519100	-0.62335200
H	3.42844200	-3.42450600	-1.89390300
H	1.53420700	-2.78171600	1.90699500
H	2.02767300	-4.43314600	1.45553900
H	3.25995500	-3.24048100	1.90305400
H	1.04285800	-4.55109100	-0.74909300
H	0.16903500	-3.05608800	-0.30127900
H	1.06353600	-3.14719700	-1.83610200
H	5.10489800	-3.09114200	0.65905000
H	5.71234700	-3.34569000	-0.97209900
H	0.76872800	-0.40185900	-2.06756800
H	2.18786300	-1.28927900	-1.24155800

101

INO (In)

O	-1.52545600	-0.48292500	-1.14164800
O	0.66213000	1.83986800	0.44209600
O	1.53130300	-1.52728100	-0.02289400
N	-2.45785100	-1.35629400	-0.55766700
N	-0.25474400	2.79504800	-0.04364200
N	2.76693800	-0.94681200	-0.38082500
C	-2.54934500	-2.57826400	-1.38568000
C	-1.19874300	-3.29042300	-1.30454000
H	-1.24918200	-4.25634200	-1.82940500
H	-0.92056300	-3.48200600	-0.25938700
H	-0.40203500	-2.69142300	-1.76314100
C	-2.85084800	-2.31185600	-2.86830500
H	-3.90117500	-2.05556400	-3.05493800
H	-2.61858300	-3.20670700	-3.46546000
H	-2.22248500	-1.48426900	-3.22527300
C	-3.60927700	-3.49080600	-0.75542100
H	-3.73364300	-4.37684700	-1.39900500
H	-3.21808600	-3.85293400	0.21144100
C	-4.93600800	-2.79337600	-0.51533400

H	-5.64310200	-3.47848400	-0.02042700
H	-5.40390900	-2.51538600	-1.47431300
C	-4.71083900	-1.55961800	0.34020100
H	-4.33753100	-1.87433200	1.33060300
H	-5.65297500	-1.01415200	0.51254200
C	-3.69021300	-0.59125700	-0.26816800
C	-4.27876900	0.15044700	-1.47797700
H	-4.80640300	-0.51154400	-2.17557500
H	-3.47250900	0.65950100	-2.02376600
H	-4.99778400	0.91057600	-1.13616400
C	-3.32570000	0.45699700	0.78314000
H	-2.64362100	1.21305900	0.36534300
H	-2.83937600	-0.02760200	1.64174700
H	-4.22931800	0.97475500	1.13876600
C	0.13225200	3.20779000	-1.41170600
C	1.57266300	3.72398700	-1.54240700
H	1.87545800	3.72002800	-2.60077300
H	2.25011500	3.05891700	-0.98816700
H	1.70297300	4.74659900	-1.16778100
C	-0.00470500	1.98370000	-2.31807500
H	-0.95908500	1.46169300	-2.14867300
H	0.83030400	1.28045900	-2.17028500
H	0.04074100	2.28687600	-3.37480400
C	-0.88886400	4.25081300	-1.88340200
H	-0.58673200	4.60738200	-2.88148500
H	-1.86130300	3.74302100	-2.00419200
C	-1.05284500	5.40578500	-0.91300100
H	-1.82976300	6.09916500	-1.27312000
H	-0.12249900	5.99515100	-0.85546800
C	-1.42363100	4.86571100	0.45599800
H	-2.41031000	4.37542500	0.39221800
H	-1.51597900	5.67861500	1.19469400
C	-0.41758100	3.83835500	0.98932300
C	0.89269400	4.51373500	1.42591800
H	1.26171500	5.25022500	0.70187600
H	1.66968700	3.75056900	1.57216900
H	0.74095600	5.03977800	2.38092200
C	-1.02253200	3.17304000	2.22441200
H	-1.90985100	2.58533300	1.94982100
H	-1.32277600	3.93901000	2.95551300
H	-0.28960200	2.51079000	2.70106600

C	3.25173000	-1.59519500	-1.61547400
C	3.33181400	-3.12674300	-1.52878600
H	2.41281500	-3.51456800	-1.06774000
H	4.19034600	-3.47815600	-0.94258500
H	3.42242700	-3.55733200	-2.53760400
C	2.27119800	-1.23465700	-2.73236500
H	1.27594700	-1.65938900	-2.54315600
H	2.63105100	-1.62328000	-3.69686000
H	2.17832400	-0.14217800	-2.81862200
C	4.61288000	-0.98117700	-1.96775600
H	5.01589600	-1.50851700	-2.84767000
H	4.44556400	0.06650300	-2.27112500
C	5.59692300	-1.00112000	-0.81254300
H	6.53737200	-0.50710000	-1.10511400
H	5.86665300	-2.03825300	-0.55192300
C	4.98168500	-0.29612900	0.38267600
H	4.80765500	0.76254900	0.12464400
H	5.66258900	-0.30803200	1.24937300
C	3.64125100	-0.90641900	0.80855000
C	3.84351200	-2.26576500	1.49585600
H	4.24271400	-2.11684500	2.51106300
H	4.54225800	-2.92126600	0.96103600
H	2.87752200	-2.78407700	1.57576600
C	2.98462900	0.03624100	1.82002300
H	2.68075500	0.97946600	1.33983200
H	3.68559200	0.26435400	2.63735200
H	2.09531700	-0.43121500	2.26602700
O	-0.50250000	-0.88018000	2.09984700
C	-0.60403000	-0.21185400	3.36911800
C	-0.86534900	-1.32226500	4.37071500
H	-1.40992300	0.53100100	3.30870200
H	0.34612800	0.31073400	3.56455600
C	-0.46067400	-2.31345900	2.26692500
C	-0.15151700	-2.51114700	3.73592100
H	0.30925800	-2.69834400	1.58390000
H	-1.44652100	-2.71279500	1.98030600
H	0.93479500	-2.45027900	3.90928200
H	-0.50335300	-3.48266200	4.10917200
H	-1.94591800	-1.52143900	4.44722000
H	-0.49449100	-1.06932700	5.37346400
In	0.08638100	-0.07834200	0.05168000

88

IN1 (In)

O	-0.39082300	1.97918900	-0.04233900
O	-1.52876800	-1.33516100	-0.01612200
O	1.90284500	-0.64796900	0.03297700
N	0.89733700	2.57072000	0.00713100
N	-2.68654500	-0.51436900	0.00201000
N	1.78573500	-2.06137600	0.00845700
C	1.05589500	3.26402500	1.29721800
C	1.03257500	2.18510600	2.38479000
H	1.34160300	2.60735300	3.35267800
H	1.73384700	1.37521700	2.12413800
H	0.02304500	1.76820000	2.51800900
C	-0.04816200	4.28547200	1.60064800
H	0.05254200	5.20707300	1.01309900
H	-0.02015000	4.56592700	2.66464100
H	-1.03031500	3.84271400	1.38389400
C	2.44350500	3.91739200	1.30627500
H	2.54508600	4.50420300	2.23343300
H	3.19996300	3.11534600	1.34792200
C	2.70440300	4.77191400	0.07913300
H	3.72242600	5.19104900	0.11766600
H	2.01988700	5.63618500	0.05641000
C	2.53436200	3.92404000	-1.16842600
H	3.29277600	3.12275700	-1.15950300
H	2.70277600	4.51612000	-2.08242500
C	1.15021100	3.27008100	-1.26438500
C	0.07125400	4.29366000	-1.64207700
H	0.12881000	5.21196100	-1.04361100
H	-0.92429600	3.85058000	-1.50008300
H	0.17691800	4.57984000	-2.69962800
C	1.20734300	2.19667800	-2.35589800
H	0.21189100	1.77523200	-2.56088800
H	1.89106900	1.38856200	-2.04886300
H	1.58230800	2.62537500	-3.29729400
C	-3.40645900	-0.73639000	1.26790800
C	-3.74937800	-2.20650800	1.54376700
H	-4.03560700	-2.33517600	2.59872400
H	-2.86991300	-2.83424500	1.34338900
H	-4.58187500	-2.57146400	0.92852300

C	-2.49458300	-0.23123700	2.39027100
H	-2.14380400	0.78746900	2.15723000
H	-1.62726500	-0.89232000	2.53435100
H	-3.04264600	-0.19426900	3.34360500
C	-4.66538500	0.13963800	1.24661700
H	-5.25402700	-0.07614000	2.15284600
H	-4.35040800	1.19511100	1.31118100
C	-5.49656000	-0.04633000	-0.00997300
H	-6.36724000	0.62830800	0.00763800
H	-5.90533700	-1.06945100	-0.05614400
C	-4.63755400	0.23991800	-1.22843400
H	-4.31973300	1.29611600	-1.19937100
H	-5.20603600	0.10043300	-2.16206300
C	-3.37935100	-0.63498000	-1.29283300
C	-3.71722300	-2.07734900	-1.69187100
H	-4.56078600	-2.48913000	-1.12324300
H	-2.84172200	-2.72059800	-1.52648800
H	-3.98389600	-2.12044500	-2.75886800
C	-2.44418700	-0.04379500	-2.35294200
H	-2.08876000	0.94835800	-2.02933700
H	-2.97521700	0.07733800	-3.30892900
H	-1.58089400	-0.70003500	-2.54138300
C	2.29074000	-2.60150600	1.28252100
C	3.71429300	-2.14918200	1.63367500
H	3.80733900	-1.06609800	1.47263300
H	4.48129000	-2.65486500	1.03296100
H	3.92736200	-2.36333100	2.69207500
C	1.33944500	-2.11056200	2.37847200
H	1.45866200	-1.03263100	2.56390300
H	1.54322300	-2.63125600	3.32610600
H	0.29658000	-2.31822800	2.08840700
C	2.18894900	-4.13034100	1.21325000
H	2.63646400	-4.55001000	2.12860400
H	1.12021800	-4.40413700	1.22261600
C	2.83144200	-4.71124400	-0.03334200
H	2.70387000	-5.80528100	-0.05181900
H	3.91944300	-4.53160200	-0.02798700
C	2.19434900	-4.08945200	-1.26283700
H	1.12568800	-4.36266100	-1.28566600
H	2.64574100	-4.47862500	-2.18970400
C	2.29632400	-2.55914700	-1.28078700

C	3.72104600	-2.09549400	-1.61166900
H	3.93768600	-2.27234500	-2.67621200
H	4.48553600	-2.62275700	-1.02654600
H	3.81474200	-1.01895800	-1.41180800
C	1.34827200	-2.03310100	-2.36348800
H	0.30458500	-2.25690500	-2.08777100
H	1.55888000	-2.51722300	-3.32880100
H	1.46429700	-0.94860700	-2.51060600
In	-0.01024000	-0.00406500	-0.00682500

90

IN2 (In)

O	-1.31519900	-1.53926000	-0.04821100
O	1.97782700	-0.37126200	-0.01813800
O	-0.67686300	1.90576800	-0.06794900
N	-2.58199700	-0.90081100	-0.01880600
N	2.06920700	-1.78575100	-0.00913300
N	0.50940600	2.68172400	-0.01302700
C	-3.26069600	-1.25094500	1.24079500
C	-2.43284100	-0.63750000	2.37373000
H	-2.97723300	-0.70209900	3.32774600
H	-2.23840000	0.42662100	2.16166800
H	-1.47595400	-1.16311800	2.50508500
C	-3.38027000	-2.76066100	1.48863900
H	-4.14927300	-3.23443900	0.86499600
H	-3.64324400	-2.95025000	2.54047100
H	-2.41675800	-3.24562300	1.27860100
C	-4.63591700	-0.57186800	1.23225200
H	-5.18611400	-0.89023500	2.13239400
H	-4.48168700	0.51729600	1.31789100
C	-5.42986000	-0.85553400	-0.03021900
H	-6.39114500	-0.31820500	-0.00292500
H	-5.68200900	-1.92701700	-0.09628300
C	-4.62272300	-0.42267400	-1.24096900
H	-4.46480600	0.66818600	-1.19217300
H	-5.16432700	-0.62794700	-2.17843000
C	-3.24870200	-1.09998200	-1.31747800
C	-3.36971800	-2.56917400	-1.74330500
H	-4.14447600	-3.11099100	-1.18579600
H	-2.40963900	-3.08022200	-1.58593400
H	-3.62552000	-2.63159000	-2.81201000

C	-2.41094700	-0.35774900	-2.36410400
H	-1.46010600	-0.87578500	-2.56199400
H	-2.20542200	0.66968300	-2.02135100
H	-2.95366800	-0.29888400	-3.31938900
C	2.69403300	-2.21367400	1.25426700
C	4.05232400	-1.55749000	1.53673000
H	4.33233700	-1.71033500	2.59016500
H	3.98745200	-0.47678100	1.34840400
H	4.85993900	-1.96825100	0.91730300
C	1.72659500	-1.82882900	2.37752100
H	0.71401200	-2.19501900	2.14217200
H	1.69495900	-0.73925400	2.52404600
H	2.04276000	-2.28164300	3.32936000
C	2.80567200	-3.74318300	1.22287800
H	3.34242500	-4.07110100	2.12769000
H	1.78707000	-4.16297700	1.28284400
C	3.47574000	-4.26272800	-0.03624300
H	3.50071000	-5.36400900	-0.02642200
H	4.52776600	-3.93485500	-0.07832900
C	2.71439700	-3.76758200	-1.25258800
H	1.69397400	-4.18638000	-1.22837400
H	3.18265800	-4.11455900	-2.18781900
C	2.60107000	-2.23891600	-1.30633400
C	3.93510900	-1.59053500	-1.69958800
H	4.78629500	-1.99465200	-1.13662600
H	3.88606600	-0.50718800	-1.52155000
H	4.13521900	-1.75852600	-2.76881500
C	1.55376300	-1.87795500	-2.36494700
H	0.55869600	-2.22506600	-2.04142100
H	1.79188700	-2.36402200	-3.32286500
H	1.52266900	-0.79350000	-2.54995600
C	0.52884400	3.43225400	1.25409500
C	-0.71759500	4.29540400	1.49184500
H	-1.62001700	3.71165700	1.26254300
H	-0.72888400	5.20501400	0.87770600
H	-0.76401000	4.60723600	2.54642500
C	0.62311100	2.39716800	2.37887000
H	-0.31917900	1.84247300	2.49600700
H	0.83698000	2.88928500	3.33945200
H	1.44047300	1.68793800	2.16864000
C	1.80927700	4.27643400	1.27275100

H	1.79998700	4.90224100	2.17972400
H	2.66978800	3.59176100	1.36251900
C	1.98263700	5.11872500	0.02164900
H	2.93115100	5.67702000	0.06829200
H	1.18588600	5.87801700	-0.04720900
C	1.96612000	4.21686600	-1.19949900
H	2.82891200	3.53121600	-1.14721200
H	2.07326700	4.79864600	-2.12922300
C	0.69058900	3.37111300	-1.30221400
C	-0.51224400	4.21951200	-1.73600400
H	-0.42312900	4.48249200	-2.80107600
H	-0.59799400	5.15610000	-1.17031100
H	-1.43911700	3.64575200	-1.59679300
C	0.92440200	2.28447200	-2.35672700
H	1.70409800	1.58670500	-2.00969700
H	1.26159400	2.73351500	-3.30289600
H	0.00085200	1.72573900	-2.57189900
In	-0.00759400	0.00023500	-0.03182200
H	-0.24563600	0.27957400	4.86568200
H	0.26452500	-0.27905400	4.78258300

90

TS1 (In)

O	1.29789000	-1.76166900	0.63444300
O	-1.67995200	-0.74897000	-0.90593700
O	0.82945700	1.61743900	0.01119400
N	2.51993100	-1.21043800	0.14930600
N	-2.42220800	-1.45551800	0.07094500
N	-0.15911900	2.61253600	-0.06311600
C	2.93859300	-1.93390600	-1.06762300
C	1.88390400	-1.64524100	-2.14003900
H	2.21448800	-2.00957400	-3.12386700
H	1.72880500	-0.55511900	-2.23721500
H	0.92953200	-2.14512200	-1.91642600
C	3.04008700	-3.45398500	-0.89131100
H	3.93366000	-3.75633900	-0.33119500
H	3.08335900	-3.94566900	-1.87492500
H	2.15372400	-3.82309400	-0.35698800
C	4.26682200	-1.32656300	-1.53717600
H	4.62687600	-1.90649400	-2.40224100
H	4.06748000	-0.30265700	-1.89715500

C	5.31285600	-1.26150900	-0.43944400
H	6.23057700	-0.78526400	-0.81948700
H	5.60557700	-2.27650200	-0.12309400
C	4.76075900	-0.47462100	0.73547100
H	4.56670000	0.56248300	0.41273900
H	5.48911500	-0.42300500	1.56068500
C	3.45174900	-1.05524500	1.28336900
C	3.69525000	-2.35486000	2.06172100
H	4.36020200	-3.05207900	1.53617000
H	2.73674300	-2.85984900	2.24531000
H	4.15771100	-2.12563100	3.03365200
C	2.83001200	-0.02964100	2.23364400
H	1.94914800	-0.44656500	2.74297800
H	2.52505400	0.86746700	1.67325900
H	3.55903700	0.26283300	3.00432300
C	-2.75219000	-2.79439800	-0.45865400
C	-3.48157000	-2.76946000	-1.80929600
H	-3.44128700	-3.76727700	-2.27190200
H	-2.98698800	-2.05657100	-2.48354400
H	-4.53877800	-2.48907600	-1.72295200
C	-1.43418800	-3.54792400	-0.64653000
H	-0.79801300	-3.46339100	0.24665600
H	-0.87675000	-3.15531800	-1.50782600
H	-1.63500900	-4.61319000	-0.83593200
C	-3.57984900	-3.52130000	0.60877500
H	-3.88824800	-4.49760200	0.20134800
H	-2.92403200	-3.72923400	1.47151000
C	-4.77915100	-2.71858500	1.08075800
H	-5.31575000	-3.26551200	1.87227500
H	-5.50233900	-2.58644000	0.25895600
C	-4.31341600	-1.37046500	1.60052400
H	-3.68020300	-1.53156800	2.48969800
H	-5.16432200	-0.74984000	1.92493900
C	-3.50195100	-0.57612600	0.56932600
C	-4.40451100	0.00311600	-0.52840300
H	-5.12218200	-0.72697500	-0.92278400
H	-3.78867100	0.36857000	-1.36156800
H	-4.98151400	0.85023400	-0.12760200
C	-2.81987300	0.58859600	1.28785000
H	-2.12191900	0.20589900	2.05106900
H	-3.56231000	1.21004300	1.81132100

H	-2.27628800	1.25062700	0.59366800
C	-0.23483600	3.12747900	-1.44749200
C	1.09529600	3.65347600	-2.00603000
H	1.90302100	2.95121700	-1.75587300
H	1.36508500	4.64417700	-1.62027700
H	1.03374600	3.73265300	-3.10216200
C	-0.69380500	1.97501200	-2.34426500
H	0.11968400	1.25521000	-2.52670400
H	-0.99561700	2.36280700	-3.32864500
H	-1.54845300	1.43911700	-1.90487000
C	-1.32180900	4.20940700	-1.47063800
H	-1.35572300	4.64749700	-2.48126000
H	-2.29582200	3.71893100	-1.30083800
C	-1.11595100	5.27995900	-0.41410500
H	-1.94355400	6.00662300	-0.44307600
H	-0.19936300	5.85604700	-0.62380800
C	-1.03451500	4.63372100	0.95719200
H	-2.00399900	4.15891200	1.18618100
H	-0.85252300	5.38500800	1.74267200
C	0.05516300	3.55846100	1.05300000
C	1.45830400	4.18381500	1.08956300
H	1.63659600	4.64022800	2.07496000
H	1.60204200	4.96669300	0.33489500
H	2.21688300	3.40499900	0.92978900
C	-0.14404900	2.78907600	2.36022600
H	-1.12100600	2.28800000	2.37190900
H	-0.10702700	3.48528800	3.21138500
H	0.64346200	2.03677100	2.50343000
H	-1.13059600	-1.92238200	2.28287400
H	-0.58855000	-1.92372900	2.82352500
In	0.08359900	-0.26852000	-0.01077500

90

IN3-In

O	1.36427800	0.69237000	-0.09457000
N	2.04314500	1.90055300	0.05171800
C	1.77572400	2.85594400	-1.03835800
C	1.95524700	2.35847200	1.45143000
C	2.68329100	4.07572200	-0.81892200
C	0.31786100	3.33362400	-1.16492000
C	2.19895300	2.20582200	-2.35646700

C	2.81933500	3.61952300	1.60172100
C	0.52171600	2.59498600	1.94862800
C	2.59901000	1.28036500	2.32593700
C	2.53391300	4.68415700	0.56186500
H	3.72854700	3.75142200	-0.95719600
H	2.46731500	4.81378100	-1.60837100
H	0.14351000	3.76438800	-2.16189400
H	-0.41272500	2.52190700	-1.04548200
H	0.05615200	4.09573800	-0.42096400
H	1.54911400	1.36770600	-2.64104100
H	2.15630700	2.94835600	-3.16740900
H	3.22976500	1.83236400	-2.27700700
H	2.68399200	4.00943200	2.62350600
H	3.87692300	3.31729600	1.51298900
H	-0.12715100	1.75876200	1.64748200
H	0.50512600	2.65543500	3.04757800
H	0.08516800	3.52577700	1.56341400
H	2.76880300	1.66912200	3.34159200
H	1.95884600	0.39549500	2.41100900
H	3.56585200	0.97656400	1.89692800
H	1.52153600	5.09955100	0.69779300
H	3.22718900	5.53181300	0.68326000
O	-1.83508500	-0.09409400	0.46354100
N	-3.11490800	-0.03443100	-0.12017400
C	-3.72498900	-1.37885800	-0.09829700
C	-3.85023700	1.10184400	0.46569100
C	-5.12737300	-1.27218400	-0.71127600
C	-3.77744500	-2.02723400	1.29371000
C	-2.88226600	-2.27734900	-1.00814600
C	-5.24897900	1.13815500	-0.16193300
C	-3.92577200	1.07502400	2.00023600
C	-3.11057400	2.37605300	0.05485100
C	-5.97668000	-0.19044600	-0.06956700
H	-5.01451400	-1.04487000	-1.78520900
H	-5.61493400	-2.25844000	-0.64199400
H	-3.94621000	-3.11126600	1.20140900
H	-2.82062000	-1.86676000	1.80854200
H	-4.57897100	-1.62160900	1.92408000
H	-1.88656500	-2.47481900	-0.58465100
H	-3.38234400	-3.24746200	-1.15132500
H	-2.75849000	-1.80895200	-1.99757200

H	-5.82638800	1.94550200	0.31759900
H	-5.13895300	1.41014800	-1.22587000
H	-2.93913600	0.81076600	2.40553800
H	-4.20568400	2.06855700	2.38314100
H	-4.66235200	0.35592000	2.38105200
H	-3.70608800	3.26386600	0.31773300
H	-2.14292200	2.45133200	0.56745000
H	-2.93929100	2.38251200	-1.03273000
H	-6.19102300	-0.44080600	0.98295300
H	-6.95556000	-0.12594800	-0.57161600
O	0.58928500	-1.89682000	-0.89730400
N	1.70824700	-1.91576600	-0.10469500
C	1.42547600	-2.66779100	1.19422700
C	2.93254500	-2.32945400	-0.91743300
C	2.70794400	-2.67127900	2.03040500
C	0.90935600	-4.07342900	0.91268700
C	0.33461100	-1.88528800	1.91456900
C	4.13670700	-2.34479900	0.02868200
C	2.70707400	-3.66344900	-1.61521900
C	3.13287700	-1.24012300	-1.96389100
C	3.92415800	-3.18080000	1.27787900
H	2.90482800	-1.64391400	2.37982200
H	2.51276500	-3.27240500	2.93203000
H	0.41436400	-4.44562100	1.82078000
H	0.16783300	-4.04367100	0.10411000
H	1.69798100	-4.78781800	0.65058500
H	-0.62632800	-1.94213200	1.39010200
H	0.21150300	-2.29326300	2.92781700
H	0.58880000	-0.82060600	2.00060400
H	5.00632500	-2.69731700	-0.54750700
H	4.36163300	-1.30444900	0.32316500
H	1.69893000	-3.68794400	-2.04879500
H	3.43778100	-3.75644500	-2.43103300
H	2.83557400	-4.52992000	-0.95673700
H	4.07190100	-1.43287900	-2.50211900
H	2.30972200	-1.23542100	-2.69052000
H	3.19026400	-0.25041800	-1.49242900
H	3.80430500	-4.24492400	1.01760000
H	4.81468600	-3.12421100	1.92236800
H	-0.83095700	0.34635900	-2.65311700
H	1.88986500	-0.90005100	0.14876000

In	-0.40996100	0.02239000	-1.02306200

60

IN4 (In)

O	-1.42828400	0.23952200	1.10751000
O	1.32852900	0.53950200	-0.73351100
N	-2.39081000	-0.00624400	0.07782000
N	2.43451800	-0.04538300	-0.05986900
C	-2.71140900	1.27055400	-0.62004300
C	-1.44841700	1.73273300	-1.34388800
H	-1.67012900	2.61308400	-1.96496100
H	-1.07212600	0.94615300	-2.02098900
H	-0.64671400	2.00262800	-0.64390300
C	-3.14080700	2.39073600	0.33524000
H	-4.17465900	2.28612000	0.68542200
H	-3.06237300	3.36194700	-0.17527600
H	-2.47334200	2.40151900	1.20745500
C	-3.78516800	0.98214700	-1.67620600
H	-4.06230200	1.93652800	-2.15198400
H	-3.33307400	0.35493200	-2.46422400
C	-5.00137200	0.26696900	-1.11965500
H	-5.71865800	0.05097300	-1.92719600
H	-5.53566900	0.91135900	-0.40222300
C	-4.55828800	-1.02156100	-0.45312100
H	-4.12222500	-1.68708800	-1.21829800
H	-5.41234900	-1.56121400	-0.01329700
C	-3.51212000	-0.80480900	0.64616900
C	-4.12844400	-0.16204500	1.89478700
H	-4.80127300	0.67208800	1.66299900
H	-3.32643000	0.20414500	2.54983500
H	-4.71294000	-0.91345100	2.44611900
C	-2.95649200	-2.17046400	1.04901600
H	-2.22889800	-2.07745100	1.86757900
H	-2.47216700	-2.66561500	0.19432600
H	-3.77601900	-2.81781200	1.39430300
C	2.84043400	0.82867500	1.06746000
C	3.11506400	2.28134800	0.65642200
H	2.32427900	2.62478200	-0.02427300
H	4.08375000	2.40916800	0.15766300
H	3.11401700	2.92870300	1.54622000
C	1.69003700	0.83443300	2.07590500

H	0.79773800	1.34385500	1.68487400
H	1.99285800	1.35160800	2.99861400
H	1.42084800	-0.19886000	2.35917600
C	4.06213800	0.19407900	1.74325200
H	4.40649400	0.87128600	2.54149000
H	3.73732300	-0.73915000	2.23516900
C	5.18173600	-0.12994500	0.77163000
H	6.01602000	-0.61688400	1.30120100
H	5.59582500	0.79471700	0.33669200
C	4.64953500	-1.04037800	-0.31967400
H	4.33707300	-1.99627300	0.13505800
H	5.43088100	-1.27971800	-1.05902800
C	3.44674100	-0.44872400	-1.06362300
C	3.86844500	0.69569600	-1.99500800
H	4.37195800	0.28739300	-2.88424000
H	4.56001900	1.40288200	-1.52060500
H	2.97585100	1.24614900	-2.32221700
C	2.82458200	-1.55169700	-1.92131400
H	2.45368100	-2.37150800	-1.28790800
H	3.57780800	-1.96301100	-2.60986000
H	1.99063700	-1.16191800	-2.52194400
In	-0.05597900	-0.80060500	-0.02728800
H	0.03218000	-2.47969000	-0.23640600

62

TS2 (In)

O	-1.40144700	0.15736700	-0.84301000
O	1.37493200	0.22559300	0.96563300
N	-2.48736200	-0.02379600	0.04347700
N	2.44979700	-0.02326400	0.05935900
C	-3.57216800	-0.73754300	-0.66855500
C	-3.99699900	-0.07385100	-1.98608300
H	-4.56310100	-0.79008600	-2.60084400
H	-3.10209400	0.23287700	-2.54454100
H	-4.63390900	0.80694100	-1.83813500
C	-3.05719800	-2.14067100	-0.99803500
H	-2.66322400	-2.63594500	-0.09702300
H	-2.26599800	-2.10692000	-1.76030600
H	-3.87649300	-2.76006200	-1.39278900
C	-4.75746700	-0.87749900	0.29466500
H	-5.58988900	-1.35592900	-0.24645900

H	-4.46564200	-1.56862800	1.10454200
C	-5.18414200	0.44374800	0.90737200
H	-6.00971000	0.28483200	1.61942400
H	-5.57860800	1.11949100	0.13050700
C	-3.99802300	1.07712200	1.61094200
H	-3.69364900	0.42789700	2.45016700
H	-4.26511400	2.05327400	2.04716600
C	-2.78470500	1.27523000	0.69350000
C	-3.01531900	2.42541800	-0.29680800
H	-4.00068200	2.38889900	-0.77777600
H	-2.24494300	2.39549500	-1.07935600
H	-2.94007200	3.38964100	0.22817800
C	-1.58181700	1.63916400	1.56583500
H	-1.35935200	0.83755200	2.28571900
H	-1.79826900	2.55369500	2.13825000
H	-0.67915600	1.81501500	0.96571500
C	3.58496300	-0.60699500	0.81746400
C	4.00405200	0.24635900	2.02133800
H	3.10833200	0.57638800	2.56478200
H	4.58747000	1.13113400	1.73861300
H	4.62439400	-0.35432900	2.70320000
C	3.13432700	-1.97174500	1.33935800
H	2.33181100	-1.86450600	2.08274400
H	3.97881700	-2.48587600	1.82179100
H	2.77478300	-2.60554400	0.51483300
C	4.75289700	-0.82292400	-0.15139800
H	5.61475500	-1.19595100	0.42494800
H	4.47274300	-1.62262400	-0.85874100
C	5.11097100	0.42441000	-0.93751300
H	5.92855500	0.20893700	-1.64351300
H	5.48914100	1.21158900	-0.26462700
C	3.88506000	0.90826600	-1.68848400
H	3.59233100	0.13780500	-2.42284900
H	4.09907200	1.82558500	-2.26015900
C	2.68258800	1.18298400	-0.77680000
C	2.87221900	2.46627700	0.04065400
H	2.73960800	3.34491700	-0.60803700
H	3.86757900	2.53990600	0.49570100
H	2.11880900	2.50504300	0.83911600
C	1.45087700	1.36394000	-1.66452700
H	1.25437900	0.44260000	-2.24389400

H	1.61981900	2.16625600	-2.39797000
H	0.55150400	1.62260800	-1.08810600
H	-1.63114900	-1.41021000	1.78330000
H	-1.12082400	-1.82561900	2.18765900
In	0.13605100	-0.99691000	-0.12229800
H	0.35583300	-2.65194800	-0.44699700

60

TS2' (In)

O	1.29790600	-0.21932700	0.93996800
O	-2.10041000	-1.64855600	0.00460300
N	2.30251200	0.05387700	0.07951900
N	-2.30318300	-0.23764800	0.03626400
C	3.52706500	-0.72926400	0.40070300
C	3.17567400	-2.21361200	0.30865000
H	4.08828700	-2.81231300	0.44441600
H	2.75015300	-2.45819900	-0.67583800
H	2.45748200	-2.50227300	1.08868700
C	4.02922800	-0.45450600	1.82381400
H	4.54646300	0.50792700	1.92105200
H	4.73633100	-1.24187700	2.12581000
H	3.17594700	-0.46699600	2.51570700
C	4.59737900	-0.41789600	-0.65007700
H	5.52678100	-0.93383900	-0.36012000
H	4.27525100	-0.85745600	-1.60992400
C	4.83108500	1.06890700	-0.84568500
H	5.58237100	1.23433700	-1.63401000
H	5.24768900	1.51953500	0.07031100
C	3.52292500	1.73963000	-1.22277400
H	3.18304000	1.33709900	-2.19268900
H	3.65399900	2.82536300	-1.35744800
C	2.40856100	1.51363300	-0.19606800
C	2.64342200	2.32480700	1.08479700
H	3.67034400	2.24561300	1.46151100
H	1.95588800	1.97629600	1.86760700
H	2.44320000	3.38961000	0.89190100
C	1.08857500	1.98448700	-0.80666800
H	0.25374700	1.87039600	-0.10053100
H	0.85699800	1.42564000	-1.72592900
H	1.16897700	3.04871900	-1.07200700
C	-2.69573600	0.17603200	1.41136700

C	-3.94540400	-0.55169500	1.92227800
H	-3.87082900	-1.61924900	1.67398100
H	-4.88042900	-0.15815800	1.50615900
H	-4.00486500	-0.45365600	3.01641900
C	-1.54156100	-0.16811200	2.35245500
H	-1.43121400	-1.25684800	2.45505200
H	-1.74727800	0.24508200	3.35121200
H	-0.58739400	0.25325300	1.99999500
C	-2.88436200	1.69692700	1.40967500
H	-3.23143100	2.00334100	2.40951600
H	-1.89619300	2.16555500	1.25976200
C	-3.83149600	2.17987100	0.32645000
H	-3.89662100	3.27941900	0.33939700
H	-4.85387500	1.81315200	0.51614200
C	-3.33825300	1.70281200	-1.02710100
H	-2.36246800	2.17480900	-1.23655700
H	-4.02120000	2.01307900	-1.83437000
C	-3.15841000	0.18178400	-1.10655300
C	-4.50251900	-0.55447500	-1.15899100
H	-4.94197300	-0.45640900	-2.16280300
H	-5.23441400	-0.16641200	-0.44098000
H	-4.33907000	-1.62121000	-0.95349500
C	-2.40628000	-0.13912800	-2.39838700
H	-1.41033500	0.33628100	-2.40401900
H	-2.95842200	0.25674300	-3.26342900
H	-2.29293500	-1.22387300	-2.53634200
In	-0.11714300	-1.15849300	-0.45282600
H	1.43340100	-0.73807400	-1.30001800

62

IN5 (In)

O	-1.39408100	-0.05672400	0.63815000
N	-2.58798900	-0.04812900	-0.10075500
C	-3.70082900	-0.42086500	0.79058700
C	-2.66678200	1.20003400	-0.88049900
C	-5.00024300	-0.37609300	-0.02285000
C	-3.80511300	0.44168800	2.05811200
C	-3.46097500	-1.86983300	1.22425200
C	-3.99540600	1.19901200	-1.64714400
C	-2.51262200	2.48069000	-0.04435300
C	-1.52698400	1.16194900	-1.90182200

C	-5.19560600	0.93942100	-0.75467700
H	-4.96836000	-1.19301000	-0.76417700
H	-5.84535700	-0.58829000	0.65262400
H	-4.43472300	-0.05818600	2.81056500
H	-2.79872400	0.57884300	2.47750900
H	-4.24146800	1.43096900	1.86767800
H	-2.59217300	-1.94229000	1.89414300
H	-4.33864200	-2.25895600	1.76324700
H	-3.28384400	-2.50434800	0.34183500
H	-4.09322100	2.15827100	-2.18172700
H	-3.94626700	0.40746100	-2.41451500
H	-1.67663400	2.35912300	0.65807800
H	-2.29543500	3.33839800	-0.69994300
H	-3.41375100	2.72620700	0.53241000
H	-1.61125300	2.00853700	-2.60090400
H	-0.54614800	1.21545600	-1.40890300
H	-1.57122100	0.22682500	-2.48140500
H	-5.32714000	1.76491100	-0.03501600
H	-6.11938600	0.90949300	-1.35508800
O	1.50526200	-0.14876400	-0.77871500
N	2.56186200	-0.02348600	0.05823100
C	3.84457400	-0.51138900	-0.62511600
C	2.56930900	1.35064200	0.74565800
C	5.01076100	-0.32031500	0.34620700
C	4.05136700	0.21470600	-1.94598500
C	3.62560500	-1.99653100	-0.89353400
C	3.82124800	1.44168300	1.62239200
C	2.49069300	2.45929000	-0.29244500
C	1.32488600	1.38120600	1.62525000
C	5.11023500	1.08904700	0.90234800
H	4.89858400	-1.03243600	1.18341100
H	5.93194800	-0.61133500	-0.18198900
H	4.76430300	-0.36207000	-2.55181100
H	3.09856300	0.27211600	-2.48746800
H	4.45900400	1.22526000	-1.82727700
H	2.84007300	-2.15068200	-1.64454000
H	4.56119400	-2.43394500	-1.26930500
H	3.34549100	-2.53642500	0.02474800
H	3.85753500	2.46083700	2.03735100
H	3.69680600	0.76495300	2.48642300
H	1.71698200	2.22186700	-1.03347400

H	2.20823100	3.39072900	0.21768600
H	3.44015700	2.63855900	-0.81025000
H	1.30540200	2.33178000	2.17695800
H	0.39568400	1.28731600	1.04455800
H	1.34184700	0.56735700	2.36739200
H	5.31885000	1.81156400	0.09709500
H	5.95881000	1.15746500	1.59970600
H	0.98514000	-1.95435400	1.31425600
H	2.42611100	-0.69327500	0.84600600
In	-0.06991400	-1.50002300	-0.03513500
H	-0.63245400	-2.47443500	-1.34038600

60

IN6 (In)

O	1.31675300	0.19606000	-0.75703000
N	2.48084000	0.01032400	0.00406300
C	3.64782800	-0.10789100	-0.89862200
C	2.52615900	0.99866300	1.10673000
C	4.91323900	-0.27843800	-0.05010400
C	3.77979500	1.07100400	-1.87424100
C	3.45503200	-1.37858200	-1.72769800
C	3.82621200	0.79551800	1.89387800
C	2.37895700	2.45121900	0.63019700
C	1.35289800	0.69714500	2.04002600
C	5.06289700	0.79231100	1.01473400
H	4.86592100	-1.26492300	0.44293400
H	5.78614000	-0.29953800	-0.72319400
H	4.43779300	0.79887600	-2.71390300
H	2.78282100	1.31043800	-2.26957700
H	4.19771600	1.97141800	-1.40581800
H	2.59747700	-1.27383200	-2.40639600
H	4.35409800	-1.58009400	-2.33031800
H	3.27752600	-2.24328500	-1.06874200
H	3.88908000	1.57373000	2.67225700
H	3.76107400	-0.17436300	2.41685200
H	1.56985400	2.49876100	-0.11177100
H	2.11973100	3.10488700	1.47732900
H	3.29499200	2.84851300	0.17455100
H	1.40193900	1.33501700	2.93612700
H	0.38959100	0.86885900	1.53942700
H	1.39495300	-0.35386200	2.37464400

H	5.20865900	1.78169300	0.54966400
H	5.96365100	0.60567000	1.62175900
O	-1.48708400	-0.18977400	0.65411400
N	-2.61780400	-0.10142000	-0.06527600
C	-3.84410000	-0.44919100	0.79267500
C	-2.65542300	1.18863900	-0.90388300
C	-5.09932000	-0.30922300	-0.07019700
C	-3.87486200	0.42317300	2.03769000
C	-3.64292000	-1.90450500	1.19768600
C	-3.98370500	1.23312700	-1.66222500
C	-2.43752800	2.40050000	-0.01229300
C	-1.49395800	1.06532100	-1.88203900
C	-5.20323000	1.02905900	-0.78062300
H	-5.10503300	-1.11707300	-0.82429400
H	-5.97140300	-0.49071400	0.57722700
H	-4.52662600	-0.05147000	2.78461100
H	-2.86021400	0.49632300	2.45012600
H	-4.26183100	1.43176800	1.85097300
H	-2.76428900	-2.00955400	1.84614800
H	-4.53342700	-2.25899400	1.73564500
H	-3.50396700	-2.55136800	0.31571400
H	-4.02889400	2.19549600	-2.19527100
H	-3.97204500	0.45244500	-2.44442700
H	-1.61148400	2.19941100	0.68176900
H	-2.15860600	3.25332600	-0.64687600
H	-3.32835100	2.68725000	0.55906600
H	-1.52929100	1.90802900	-2.58743000
H	-0.52036000	1.06946000	-1.36772600
H	-1.57612300	0.14013000	-2.47866100
H	-5.29846600	1.84984800	-0.05185300
H	-6.11759500	1.05806000	-1.39253500
H	-2.63511500	-0.84239800	-0.79147100
In	0.30783000	-1.60505400	-0.10316100

30

IN7 (In)

O	-0.51801900	-0.00001900	1.29576700
N	0.33368600	-0.00000700	0.17985400
C	1.09137200	1.28035200	0.11068100
C	1.09141500	-1.28033900	0.11067000
C	2.01378800	1.23791300	-1.11233600

C	1.86373700	1.57842500	1.40131600
C	0.07967600	2.40781700	-0.09182100
C	2.01381000	-1.23786200	-1.11236000
C	1.86384400	-1.57838700	1.40127500
C	0.07975200	-2.40783900	-0.09179500
C	2.89022500	0.00003300	-1.15666200
H	1.38534900	1.26152200	-2.01975300
H	2.61991200	2.15817400	-1.12203700
H	2.13313300	2.64457600	1.43957200
H	1.21894400	1.34723400	2.26027200
H	2.79190600	1.00105400	1.49058300
H	-0.56794600	2.52235500	0.78862800
H	0.60421800	3.36043800	-0.25793000
H	-0.54549000	2.21891700	-0.98217300
H	2.61994900	-2.15811400	-1.12208700
H	1.38535900	-1.26146400	-2.01976800
H	1.21910800	-1.34723900	2.26028400
H	2.13328400	-2.64452900	1.43950400
H	2.79199600	-1.00098300	1.49050400
H	0.60432700	-3.36045300	-0.25784000
H	-0.56791200	-2.52233000	0.78862600
H	-0.54539700	-2.21900400	-0.98217100
H	3.60238000	0.00003100	-0.31497600
H	3.50232500	0.00004900	-2.07248300
In	-2.09189400	-0.00001400	-0.18746300

60

IN8 (In)

C	-0.53779400	-4.72993800	1.23992100
C	0.01129800	-3.29680400	1.27770800
C	0.01129800	-3.29680400	-1.27770800
C	-0.53779400	-4.72993800	-1.23992100
C	-0.11317300	-5.49597900	0.00000000
H	-1.63914200	-4.67246500	1.26346000
H	-0.22311100	-5.25129500	2.15839300
H	-0.22311100	-5.25129500	-2.15839300
H	-1.63914200	-4.67246500	-1.26346000
H	0.97782900	-5.65723800	0.00000000
H	-0.56699200	-6.49986800	0.00000000
C	1.51110600	-3.30346100	-1.61166500
H	1.94997600	-2.30364400	-1.47051000

H	1.65972100	-3.57858900	-2.66686100
H	2.08579200	-4.01120500	-1.00165800
C	-0.71476400	-2.53789100	-2.39067400
H	-0.62091000	-3.08133000	-3.34293600
H	-0.29509900	-1.53145300	-2.53084800
H	-1.78559400	-2.44265600	-2.15092000
C	1.51110600	-3.30346100	1.61166500
H	1.65972100	-3.57858900	2.66686100
H	1.94997600	-2.30364400	1.47051000
H	2.08579200	-4.01120500	1.00165800
C	-0.71476400	-2.53789100	2.39067400
H	-0.29509900	-1.53145300	2.53084800
H	-0.62091000	-3.08133000	3.34293600
H	-1.78559400	-2.44265600	2.15092000
N	-0.34434900	-2.65678000	0.00000000
O	0.00412800	-1.30373000	0.00000000
C	0.53768900	4.72996200	1.23992100
C	-0.01136200	3.29681100	1.27771400
C	-0.01136200	3.29681100	-1.27771400
C	0.53768900	4.72996200	-1.23992100
C	0.11306400	5.49599300	0.00000000
H	1.63903900	4.67251600	1.26347000
H	0.22298200	5.25131600	2.15838600
H	0.22298200	5.25131600	-2.15838600
H	1.63903900	4.67251600	-1.26347000
H	-0.97794000	5.65723900	0.00000000
H	0.56686900	6.49988700	0.00000000
C	-1.51116400	3.30343200	-1.61166500
H	-1.94997900	2.30359700	-1.47054900
H	-1.65979300	3.57858900	-2.66685300
H	-2.08586700	4.01115200	-1.00164600
C	0.71471500	2.53793700	-2.39069900
H	0.62084600	3.08139500	-3.34294800
H	0.29507700	1.53149000	-2.53089400
H	1.78554800	2.44272200	-2.15095100
C	-1.51116400	3.30343200	1.61166500
H	-1.65979300	3.57858900	2.66685300
H	-1.94997900	2.30359700	1.47054900
H	-2.08586700	4.01115200	1.00164600
C	0.71471500	2.53793700	2.39069900
H	0.29507700	1.53149000	2.53089400

H	0.62084600	3.08139500	3.34294800
H	1.78554800	2.44272200	2.15095100
N	0.34433100	2.65679600	0.00000000
O	-0.00403000	1.30370300	0.00000000
In	-1.85603200	-0.06929400	0.00000000
In	1.85612500	0.06927700	0.00000000

32

IN9 (In)

O	0.52393800	-0.07695100	0.86044500
N	-0.54812000	-0.02421600	-0.05264400
C	-1.33477400	-1.26635800	0.06385400
C	-1.21993600	1.28294800	0.07593000
C	-2.50714300	-1.17148300	-0.92097200
C	-1.82295700	-1.57360100	1.48721300
C	-0.42780700	-2.41489200	-0.38604400
C	-2.39486400	1.30364000	-0.91004300
C	-1.67794900	1.62199300	1.50181800
C	-0.21485100	2.35123800	-0.36519900
C	-3.31380300	0.10449400	-0.76149900
H	-2.09890000	-1.20853000	-1.94557700
H	-3.14130200	-2.06421000	-0.79592900
H	-2.13369100	-2.62704500	1.56172800
H	-1.00294100	-1.40121000	2.19788600
H	-2.67859900	-0.95587200	1.78861500
H	0.37001100	-2.60205900	0.34726500
H	-1.01033400	-3.34280400	-0.49067200
H	0.02660600	-2.17915100	-1.36124900
H	-2.94581700	2.24897500	-0.77759200
H	-1.98391800	1.31186900	-1.93424100
H	-0.87423900	1.37527600	2.20932200
H	-1.89794300	2.69753900	1.58383900
H	-2.58230100	1.07700600	1.80058100
H	-0.71410600	3.32601200	-0.47341900
H	0.58838700	2.47635400	0.37694900
H	0.22392900	2.08062500	-1.33908500
H	-3.81657100	0.12306900	0.21978800
H	-4.11678700	0.14450200	-1.51487100
H	3.57901500	0.07874500	1.03600900
In	2.30867200	-0.02270700	-0.12011500
H	2.21441500	-0.08918000	-1.82912300

34

TS3 (In)

O	-0.93982800	-1.46308500	-0.33568900
N	-0.49240000	-2.47192700	-1.19423500
C	-1.38967800	-2.57966500	-2.36662200
C	-2.87206100	-2.77128700	-2.01450000
H	-3.49934300	-2.53856100	-2.88855800
H	-3.14507500	-2.08706000	-1.19918800
H	-3.11133100	-3.79491700	-1.70152600
C	-1.26640900	-1.28328100	-3.17068600
H	-0.21121200	-1.03092700	-3.35063800
H	-1.75233300	-0.43926800	-2.65866300
H	-1.76530900	-1.39959000	-4.14464800
C	-0.86975400	-3.72487300	-3.24589900
H	-1.56355700	-3.85429800	-4.09242700
H	0.10025200	-3.41676400	-3.67271400
C	-0.68104000	-5.02491300	-2.48616100
H	-0.26536100	-5.79764800	-3.15248400
H	-1.65309800	-5.41357300	-2.13991700
C	0.24713900	-4.79113100	-1.30834300
H	1.24357600	-4.50785300	-1.68890900
H	0.38136500	-5.71100600	-0.71631900
C	-0.24242900	-3.67994600	-0.37190300
C	-1.45369900	-4.13903400	0.45391200
H	-2.21078400	-4.66273400	-0.14278700
H	-1.92382800	-3.26618700	0.92688500
H	-1.12522100	-4.82848200	1.24648100
C	0.88468100	-3.34313500	0.60697600
H	1.76373200	-2.95334400	0.07463300
H	1.18834700	-4.24971400	1.15192900
H	0.55577200	-2.59630800	1.34248100
H	1.54317400	-1.64815900	-1.69704900
H	2.17872800	-1.20777300	-1.71377100
In	-0.10667700	0.40150400	-0.32617700
H	0.54631100	1.11426500	-1.74079700
H	-0.44643700	1.01659900	1.24612100

32

TS3’ (In)

O	-1.13642300	-1.23681700	-0.07285100

N	-2.17795100	-0.48901400	0.40769000
C	-3.33517100	-1.35531300	0.77553800
C	-2.87537900	-2.31541700	1.87286700
H	-3.74023100	-2.88770700	2.23931100
H	-2.44574200	-1.76820900	2.72545000
H	-2.13231800	-3.03013200	1.49195000
C	-3.82887600	-2.19955700	-0.40467400
H	-4.42651300	-1.62892600	-1.12556400
H	-4.45801600	-3.02151800	-0.03086600
H	-2.96366600	-2.63079700	-0.92612800
C	-4.44433800	-0.46476600	1.34541400
H	-5.32386000	-1.09658900	1.54838600
H	-4.10442400	-0.07135500	2.31919200
C	-4.80065800	0.70137100	0.44185500
H	-5.57337800	1.32651600	0.91645400
H	-5.24032800	0.33923800	-0.50209300
C	-3.55675300	1.52651600	0.16819600
H	-3.20011400	1.96088100	1.11828500
H	-3.77703500	2.37156600	-0.50362600
C	-2.41259800	0.71231300	-0.44507200
C	-2.69142700	0.34199400	-1.90605000
H	-3.71054800	-0.02702300	-2.07266500
H	-1.97906000	-0.43184800	-2.22275100
H	-2.55249700	1.22837800	-2.54334200
C	-1.14408000	1.56506900	-0.41635400
H	-0.30885300	1.05318000	-0.91466600
H	-0.85288200	1.80970500	0.61623300
H	-1.32979700	2.50952400	-0.94856000
In	0.29961200	-0.93361500	1.51906900
H	-1.33442200	-0.06833100	1.75674100
H	1.83707900	-1.76867600	1.25453900

34

IN10 (In)

O	0.43522100	0.00005000	0.88754100
N	-0.59443000	0.00002100	0.00869900
C	-1.37842900	-1.31548000	0.07677000
C	-1.37849400	1.31547400	0.07676000
C	-2.51968800	-1.24171900	-0.94002100
C	-1.86059900	-1.57724400	1.49582800
C	-0.39349800	-2.40688100	-0.32753800

C	-2.51970800	1.24163800	-0.94006800
C	-1.86072200	1.57719300	1.49580400
C	-0.39360300	2.40693200	-0.32750100
C	-3.38301800	-0.00004400	-0.80150900
H	-2.08955700	-1.27056000	-1.95710900
H	-3.11759300	-2.16028900	-0.83458600
H	-2.11141200	-2.64340500	1.58692500
H	-1.05465600	-1.34772800	2.20477100
H	-2.75385300	-1.00283200	1.76654000
H	0.40739300	-2.51173700	0.41546900
H	-0.93221600	-3.36194600	-0.40343300
H	0.06391500	-2.19691000	-1.30670400
H	-3.11763000	2.16020700	-0.83472100
H	-2.08953200	1.27041400	-1.95714200
H	-1.05471500	1.34793700	2.20475800
H	-2.11183100	2.64329000	1.58683400
H	-2.75381100	1.00254800	1.76656000
H	-0.93231200	3.36201800	-0.40319600
H	0.40738500	2.51168200	0.41541600
H	0.06369600	2.19710200	-1.30675100
H	-3.91219900	-0.00002900	0.16495900
H	-4.16511600	-0.00006800	-1.57575000
H	1.54621000	-0.00012500	-1.67884100
H	-0.18294800	0.00002300	-0.95252500
In	2.41654000	0.00000600	-0.11511700
H	3.14329000	-1.51303400	0.36363000
H	3.14283500	1.51340400	0.36327600

4

IN11 (In)

H	-1.72238100	-0.19701600	0.00016000
In	-0.00000600	0.00000400	-0.00001000
H	1.03191800	-1.39315600	0.00016000
H	0.69076700	1.58995300	0.00016000

60

IN4 (Ga)

O	-1.47144600	-1.24102700	0.57724600
O	1.11619900	0.45437200	0.33467000
N	-2.22593100	-0.15234200	0.01670800
N	2.33526800	-0.02639400	-0.18999300

C	-2.56715700	0.83504300	1.09681300
C	-1.27193500	1.45649000	1.60691900
H	-1.51078900	2.24381300	2.33636300
H	-0.69536600	1.91635500	0.79104900
H	-0.63104900	0.71360700	2.09825900
C	-3.26944100	0.17946200	2.29046300
H	-4.33305800	-0.01959100	2.11794400
H	-3.19794300	0.84792900	3.16058400
H	-2.76760900	-0.76491600	2.53956800
C	-3.41478100	1.95103700	0.47598000
H	-3.69928500	2.64517400	1.28263300
H	-2.77558700	2.52160300	-0.22000200
C	-4.63111700	1.44519300	-0.27377900
H	-5.17400300	2.28731600	-0.73082700
H	-5.34232200	0.96368600	0.41718000
C	-4.18091600	0.47093200	-1.34412700
H	-3.56130700	1.01058200	-2.08122400
H	-5.03860600	0.05608800	-1.89718600
C	-3.36135100	-0.70402800	-0.79878700
C	-4.22737200	-1.68281200	0.00161500
H	-4.92774300	-1.18797600	0.68332000
H	-3.58249000	-2.35534100	0.58274800
H	-4.82233700	-2.29086400	-0.69511900
C	-2.78929800	-1.46626200	-1.99162800
H	-2.20426200	-2.33900500	-1.67076400
H	-2.15407800	-0.82074700	-2.61368300
H	-3.61944100	-1.82520000	-2.61699300
C	2.96819600	-0.91408300	0.80663700
C	3.17225600	-0.27156800	2.18576700
H	2.25765500	0.26009800	2.48271900
H	4.00868600	0.43825700	2.20752500
H	3.37985400	-1.04987200	2.93585100
C	2.04549600	-2.12395300	0.98110300
H	1.11816800	-1.85688400	1.51047500
H	2.54627700	-2.90494900	1.57266400
H	1.79332600	-2.55734700	-0.00106700
C	4.29551200	-1.40292600	0.21384400
H	4.81231800	-2.01374600	0.97165900
H	4.06742000	-2.06966800	-0.63537600
C	5.17806900	-0.26917200	-0.27685300
H	6.09953900	-0.67229800	-0.72648500

H	5.50276300	0.36059800	0.56812100
C	4.41667000	0.55907700	-1.29653400
H	4.19471400	-0.07272400	-2.17344900
H	5.02253500	1.40617900	-1.65688700
C	3.08964700	1.10694000	-0.75682200
C	3.32028300	2.25946700	0.23114600
H	3.62841200	3.16477500	-0.31380800
H	4.09977500	2.03935000	0.97141700
H	2.38600600	2.47867100	0.76639600
C	2.27664300	1.64525900	-1.93603900
H	2.02016100	0.82892200	-2.62745700
H	2.86057600	2.39939200	-2.48507800
H	1.34663300	2.11964100	-1.59213900
H	0.10240000	-0.98512100	-1.98024100
Ga	-0.17515300	-0.51019200	-0.53726900

62

TS2 (Ga)

O	-1.35682000	0.15030100	-0.89129200
O	1.24022500	0.12693600	0.69598400
N	-2.40863600	-0.09970500	0.03255400
N	2.43945200	-0.17213000	-0.00426200
C	-3.49323900	-0.82561700	-0.66840800
C	-3.99185700	-0.12807700	-1.94130300
H	-4.55681300	-0.84210000	-2.55935500
H	-3.13312900	0.23061200	-2.52483800
H	-4.65285500	0.72257600	-1.73542200
C	-2.94482200	-2.19634100	-1.06954000
H	-2.49482100	-2.70710500	-0.20467300
H	-2.18778300	-2.10897300	-1.86100900
H	-3.76002500	-2.82893000	-1.45151700
C	-4.63309600	-1.04220500	0.33508900
H	-5.46885600	-1.53005300	-0.19199300
H	-4.28489700	-1.75226300	1.10517600
C	-5.08099800	0.23856000	1.01472600
H	-5.86928000	0.02202900	1.75320500
H	-5.53238400	0.92736600	0.28160000
C	-3.89009500	0.88939300	1.69353400
H	-3.52652700	0.22168200	2.49364700
H	-4.17295200	1.83839300	2.17684900
C	-2.72419200	1.16718800	0.73623400

C	-3.03795200	2.33951000	-0.20356300
H	-4.04034400	2.28377800	-0.64556800
H	-2.29986000	2.36833900	-1.01662400
H	-2.97566900	3.28653600	0.35322700
C	-1.50075800	1.54874200	1.57042100
H	-1.20638900	0.72690700	2.23933700
H	-1.73339800	2.42559200	2.19292200
H	-0.63851200	1.79489400	0.93690300
C	3.42732400	-0.71263400	0.95059500
C	3.68885500	0.20803300	2.15035600
H	2.73376600	0.58515800	2.54120600
H	4.32566800	1.06638500	1.90166300
H	4.19212900	-0.35571500	2.95028500
C	2.87003500	-2.03680700	1.47582300
H	1.98687300	-1.86973000	2.10836900
H	3.63057700	-2.55130700	2.08217100
H	2.58765600	-2.69380600	0.63980600
C	4.71698400	-1.00283800	0.17316400
H	5.48557800	-1.33609300	0.88896400
H	4.52493900	-1.84969500	-0.50766000
C	5.20017300	0.18283800	-0.64278000
H	6.10314000	-0.08998500	-1.21165000
H	5.49746000	1.01297500	0.01928900
C	4.10000300	0.62631300	-1.59020000
H	3.89325000	-0.19054300	-2.30280700
H	4.41131400	1.49943500	-2.18591900
C	2.79226400	0.97513500	-0.86911900
C	2.89554600	2.31304700	-0.12624000
H	2.87886600	3.14409500	-0.84741200
H	3.81743700	2.40469400	0.46124200
H	2.03829800	2.42539200	0.55179000
C	1.68276400	1.09776000	-1.91735900
H	1.51083900	0.12263300	-2.40824700
H	1.97191900	1.80772600	-2.70640500
H	0.73926800	1.46194300	-1.48280500
H	-1.30365400	-1.52772100	1.61711800
H	-0.69532400	-1.89623200	1.91491300
H	0.33146000	-2.29244500	-0.78445200
Ga	0.06866000	-0.84269300	-0.31114400

60

TS2' (Ga)

O	1.21524800	0.10890900	0.87344600
O	-1.70204500	-1.43779000	0.64583000
N	2.26126000	-0.00137500	0.00969400
N	-2.24387600	-0.24533000	0.08247200
C	3.31383900	-0.92396900	0.53816600
C	2.68041100	-2.30175500	0.72925100
H	3.46211200	-3.01667400	1.02446900
H	2.22134000	-2.66513500	-0.20176600
H	1.91616900	-2.28615000	1.51859400
C	3.84804500	-0.45969200	1.89573600
H	4.53763500	0.38978100	1.82264400
H	4.39180600	-1.28740800	2.37535700
H	3.00526200	-0.17636000	2.54048800
C	4.42681500	-1.03868800	-0.50851100
H	5.23536500	-1.65387700	-0.08267600
H	4.02982100	-1.59467700	-1.37555800
C	4.94793800	0.30536900	-0.98297900
H	5.71855300	0.16066500	-1.75630300
H	5.44292100	0.84277500	-0.15745300
C	3.79832900	1.12462300	-1.54026300
H	3.38946700	0.60993900	-2.42708100
H	4.14047200	2.11508200	-1.88012500
C	2.65787200	1.33260000	-0.53820000
C	3.04343900	2.31431500	0.57168400
H	4.03762500	2.12249100	0.99253000
H	2.30291700	2.26143200	1.38104300
H	3.04683200	3.33777600	0.16739600
C	1.45666700	1.91411200	-1.28434700
H	0.62914000	2.14752900	-0.60020900
H	1.08871900	1.22087700	-2.05447400
H	1.76320400	2.84609800	-1.78126300
C	-2.66728800	0.68150600	1.17415500
C	-3.66779600	0.03768600	2.14157900
H	-3.33671600	-0.98132200	2.38374800
H	-4.69009100	-0.00774700	1.74839700
H	-3.69958500	0.62152100	3.07328700
C	-1.42973900	1.06670300	1.97966600
H	-1.01529300	0.20089600	2.51293600
H	-1.70198300	1.83158300	2.72179300
H	-0.63829000	1.47510500	1.33623500

C	-3.23440100	1.94864900	0.52528100
H	-3.59510300	2.61233900	1.32735300
H	-2.40439000	2.47709700	0.02464200
C	-4.32556300	1.66742100	-0.49048600
H	-4.66490100	2.60662500	-0.95529100
H	-5.21174000	1.23522800	0.00290400
C	-3.79230500	0.72325700	-1.55128400
H	-2.97948100	1.22802400	-2.10183400
H	-4.56870400	0.47260500	-2.29189000
C	-3.23769700	-0.58677600	-0.97965500
C	-4.35122300	-1.51152900	-0.47515800
H	-4.84817500	-1.98948400	-1.33230600
H	-5.12283800	-0.98760700	0.10056300
H	-3.91326400	-2.29808200	0.15425900
C	-2.50527400	-1.31877800	-2.10351800
H	-1.65785400	-0.72361600	-2.47955100
H	-3.19603200	-1.48747600	-2.94294600
H	-2.13023900	-2.29624200	-1.76903900
H	1.42171300	-0.72921900	-1.01454600
Ga	-0.14213300	-0.84279800	-0.23904500

62

IN5 (Ga)

O	-1.30699400	-0.19354800	0.57507200
N	-2.52056400	-0.19655600	-0.13219800
C	-3.59298900	-0.64709100	0.77241100
C	-2.66154000	1.07935400	-0.85548600
C	-4.91360800	-0.61631300	-0.00695600
C	-3.69608700	0.15753800	2.07735200
C	-3.28947400	-2.10269100	1.13672400
C	-4.00933100	1.06035200	-1.58792100
C	-2.53240300	2.32970200	0.02929300
C	-1.54864000	1.12431500	-1.90541300
C	-5.17570500	0.71989400	-0.67790200
H	-4.87057100	-1.40033700	-0.78226200
H	-5.73239600	-0.88777400	0.67948300
H	-4.29673700	-0.39344300	2.81760400
H	-2.68743800	0.30612800	2.48704300
H	-4.16430500	1.14079500	1.93891600
H	-2.39806200	-2.17324700	1.77625900
H	-4.13690900	-2.54416800	1.68371800

H	-3.11519000	-2.69091400	0.22325600
H	-4.15515800	2.03674600	-2.07860600
H	-3.95127500	0.30378100	-2.38904700
H	-1.67235900	2.21522900	0.70332000
H	-2.36742600	3.22076500	-0.59626400
H	-3.42495400	2.51625900	0.64038300
H	-1.68767800	1.98979600	-2.57176400
H	-0.55913500	1.20267700	-1.43437100
H	-1.56717600	0.20641600	-2.51244800
H	-5.31886900	1.50995900	0.07836800
H	-6.11284000	0.67975100	-1.25655900
O	1.37769500	-0.22144000	-0.72997800
N	2.47031900	-0.15620700	0.07203400
C	3.70115800	-0.69800100	-0.66151800
C	2.56271500	1.20658300	0.77110300
C	4.90656400	-0.57363800	0.27246000
C	3.89951100	0.03298500	-1.98120800
C	3.40251100	-2.16785300	-0.93721600
C	3.84327800	1.22441300	1.60994200
C	2.50917800	2.33156200	-0.25134600
C	1.34583600	1.28693000	1.68536800
C	5.09051600	0.82095400	0.84426200
H	4.79010000	-1.29271100	1.10290400
H	5.79472400	-0.89832300	-0.29119600
H	4.57163700	-0.56674100	-2.61090200
H	2.93680700	0.13430100	-2.49809200
H	4.35144000	1.02478800	-1.86564600
H	2.57997300	-2.27792600	-1.65582000
H	4.30136400	-2.64098200	-1.35681300
H	3.13605500	-2.70678500	-0.01468800
H	3.94145500	2.23461700	2.03636200
H	3.71217200	0.54214700	2.46849500
H	1.70001900	2.14809800	-0.96951000
H	2.29377000	3.26893400	0.28013300
H	3.44931600	2.46839800	-0.79805700
H	1.38919100	2.22931400	2.24929100
H	0.40226400	1.24583600	1.12329200
H	1.33833900	0.46070100	2.41337400
H	5.30895700	1.54512900	0.04315100
H	5.96257700	0.83786800	1.51521500
H	0.82206300	-1.94166400	1.19137600

H	2.31292200	-0.83278400	0.85050200
H	-0.51514400	-2.32164500	-1.24394100
Ga	-0.05239900	-1.40399100	-0.05547600

60

IN6 (Ga)

O	-1.37820800	-0.42012700	0.40008500
N	-2.61365600	-0.04270000	-0.15888200
C	-3.58833300	-1.13617100	0.01577100
C	-2.95510600	1.31290000	0.31620000
C	-4.92679700	-0.67851100	-0.57773500
C	-3.74917300	-1.60189500	1.47101500
C	-3.08780700	-2.32557400	-0.80671700
C	-4.30753900	1.70323900	-0.29172600
C	-2.96522500	1.45510800	1.84611900
C	-1.88940400	2.26533200	-0.23173900
C	-5.38755300	0.66559100	-0.04347300
H	-4.80699100	-0.60311400	-1.67215600
H	-5.67936100	-1.46218700	-0.39096900
H	-4.22666600	-2.59352900	1.50242100
H	-2.75512100	-1.67645100	1.93332400
H	-4.36352800	-0.92098000	2.07420000
H	-2.17494600	-2.75317300	-0.36912200
H	-3.85531300	-3.11445500	-0.83965100
H	-2.86412400	-2.00807800	-1.83683300
H	-4.60090700	2.68877700	0.10589600
H	-4.17191400	1.82287000	-1.38048700
H	-2.08385100	0.94285300	2.25617900
H	-2.91806300	2.51807500	2.12965700
H	-3.86418600	1.03002000	2.31059500
H	-2.18169800	3.31147200	-0.05186700
H	-0.92091900	2.08940800	0.25852700
H	-1.76975800	2.11705700	-1.31700800
H	-5.61291000	0.59532600	1.03391600
H	-6.32819000	0.96840400	-0.53152900
O	1.34045900	-0.03501800	0.38176800
N	2.58689400	-0.06485200	-0.13910400
C	3.21740200	1.33361200	-0.14993700
C	3.40065500	-1.22823100	0.44274600
C	4.62275600	1.21776100	-0.74470200
C	3.20701200	1.93020300	1.24913800

C	2.33721000	2.17871200	-1.06347300
C	4.79484600	-1.19753600	-0.18694000
C	3.42876400	-1.14700100	1.96139100
C	2.66684200	-2.49593500	0.02129200
C	5.48137000	0.15332900	-0.08435600
H	4.53403600	0.99268300	-1.82273600
H	5.09345300	2.21080300	-0.67836000
H	3.34907600	3.01679400	1.16485100
H	2.23398800	1.74063200	1.72066500
H	4.00248600	1.54099700	1.89478400
H	1.34617400	2.34347800	-0.62196100
H	2.81959400	3.15413000	-1.21904700
H	2.20266300	1.70542100	-2.04922000
H	5.39259500	-1.98822300	0.29234800
H	4.71047400	-1.48056900	-1.25155300
H	2.42581800	-0.90009300	2.33312800
H	3.71518700	-2.13169500	2.35671300
H	4.14737800	-0.41397500	2.34535400
H	3.28109600	-3.36820800	0.28602300
H	1.69771500	-2.58096200	0.52965600
H	2.49337300	-2.52139700	-1.06658600
H	5.67786400	0.41046900	0.96884300
H	6.46553500	0.11037400	-0.57485100
H	2.50881700	-0.30341600	-1.15121400
Ga	-0.13642100	-0.46855200	-1.02251900

30

IN7 (Ga)

O	0.99498100	0.00000000	1.16331700
N	0.04492500	0.00000000	0.12151300
C	-0.70992900	-1.28533300	0.10913600
C	-0.70992900	1.28533200	0.10913600
C	-1.71975400	-1.23824300	-1.04275200
C	-1.38532500	-1.58290800	1.45198900
C	0.28729100	-2.40873200	-0.17180300
C	-1.71975400	1.23824300	-1.04275200
C	-1.38532600	1.58290800	1.45198900
C	0.28729100	2.40873300	-0.17180300
C	-2.59647300	0.00000000	-1.02033600
H	-1.16013900	-1.26220100	-1.99384800
H	-2.32514600	-2.15806200	-1.00727700

H	-1.65900700	-2.64741400	1.50031300
H	-0.68130400	-1.36690500	2.26712100
H	-2.30028300	-1.00032700	1.61317100
H	0.98673300	-2.54563300	0.66479000
H	-0.25172100	-3.35558800	-0.32311300
H	0.86133300	-2.20507500	-1.09201900
H	-2.32514700	2.15806100	-1.00727700
H	-1.16014000	1.26220100	-1.99384800
H	-0.68130500	1.36690500	2.26712100
H	-1.65900700	2.64741400	1.50031300
H	-2.30028300	1.00032700	1.61317100
H	-0.25172200	3.35558800	-0.32311300
H	0.98673300	2.54563300	0.66479000
H	0.86133200	2.20507500	-1.09201900
H	-3.24432500	0.00000000	-0.12833500
H	-3.27456300	0.00000000	-1.88815700
Ga	2.23274500	0.00000000	-0.30437800

60

IN8 (Ga)

C	0.54205000	4.63098800	1.24031100
C	0.00614400	3.19266300	1.28208300
C	0.00614400	3.19266300	-1.28208300
C	0.54205000	4.63098800	-1.24031100
C	0.10869900	5.39151900	0.00000000
H	1.64378600	4.58376800	1.26346800
H	0.22241400	5.14929200	2.15849700
H	0.22241400	5.14929200	-2.15849700
H	1.64378600	4.58376800	-1.26346800
H	-0.98378200	5.54179200	0.00000000
H	0.55245600	6.39974500	0.00000000
C	-1.49251600	3.18192200	-1.61688600
H	-1.92214700	2.17641500	-1.48261400
H	-1.64090800	3.46212100	-2.67070200
H	-2.07639600	3.87923300	-1.00363700
C	0.74633100	2.43977400	-2.38991600
H	0.66387500	2.99121600	-3.33845000
H	0.32736300	1.43548000	-2.54852100
H	1.81290100	2.33926200	-2.13636500
C	-1.49251600	3.18192200	1.61688600
H	-1.64090800	3.46212100	2.67070200

H	-1.92214700	2.17641500	1.48261400
H	-2.07639600	3.87923300	1.00363700
C	0.74633100	2.43977400	2.38991600
H	0.32736300	1.43548000	2.54852100
H	0.66387500	2.99121600	3.33845000
H	1.81290100	2.33926200	2.13636500
N	0.36698900	2.56393400	0.00000000
O	0.03621600	1.20441500	0.00000000
C	-0.54205100	-4.63098800	1.24031100
C	-0.00614400	-3.19266300	1.28208300
C	-0.00614400	-3.19266300	-1.28208300
C	-0.54205100	-4.63098800	-1.24031100
C	-0.10870000	-5.39151900	0.00000000
H	-1.64378700	-4.58376800	1.26346800
H	-0.22241500	-5.14929200	2.15849700
H	-0.22241500	-5.14929200	-2.15849700
H	-1.64378700	-4.58376800	-1.26346800
H	0.98378100	-5.54179200	0.00000000
H	-0.55245800	-6.39974500	0.00000000
C	1.49251600	-3.18192300	-1.61688600
H	1.92214700	-2.17641600	-1.48261400
H	1.64090800	-3.46212100	-2.67070200
H	2.07639600	-3.87923400	-1.00363700
C	-0.74633100	-2.43977400	-2.38991600
H	-0.66387500	-2.99121600	-3.33845000
H	-0.32736200	-1.43548000	-2.54852100
H	-1.81290100	-2.33926200	-2.13636500
C	1.49251600	-3.18192300	1.61688600
H	1.64090800	-3.46212100	2.67070200
H	1.92214700	-2.17641600	1.48261400
H	2.07639600	-3.87923400	1.00363700
C	-0.74633100	-2.43977400	2.38991600
H	-0.32736200	-1.43548000	2.54852100
H	-0.66387500	-2.99121600	3.33845000
H	-1.81290100	-2.33926200	2.13636500
N	-0.36698800	-2.56393400	0.00000000
O	-0.03621500	-1.20441500	0.00000000
Ga	1.65383500	0.02594100	0.00000000
Ga	-1.65383400	-0.02594000	0.00000000

32

IN9 (Ga)

O	-0.96340300	-0.00297600	0.74337300
N	0.16398400	-0.00086500	-0.10807700
C	0.87879800	1.27913400	0.06039700
C	0.88310400	-1.27852900	0.05983900
C	2.10908600	1.24150600	-0.85548700
C	1.26959800	1.60322200	1.50932200
C	-0.05318500	2.38561500	-0.44143400
C	2.11338300	-1.23646200	-0.85592200
C	1.27483500	-1.60175400	1.50872100
C	-0.04526100	-2.38783300	-0.44242300
C	2.96403700	0.00395000	-0.65105100
H	1.75895800	1.26365000	-1.90159600
H	2.69193300	2.16239500	-0.69252300
H	1.53962800	2.66676400	1.59598600
H	0.41759800	1.40727700	2.17491000
H	2.12670000	1.01627800	1.86264900
H	-0.89466700	2.54796400	0.24869000
H	0.49457700	3.33674900	-0.52025000
H	-0.44713600	2.12887600	-1.43697100
H	2.69931300	-2.15541200	-0.69304700
H	1.76347100	-1.25964400	-1.90207900
H	0.42248700	-1.40749200	2.17434400
H	1.54709500	-2.66471800	1.59544800
H	2.13075700	-1.01299200	1.86192000
H	0.50546600	-3.33726300	-0.52118800
H	-0.88646200	-2.55275800	0.24738800
H	-0.43969300	-2.13224500	-1.43806900
H	3.41007400	0.00452300	0.35738200
H	3.80975800	0.00553300	-1.35703600
H	-3.74763000	-0.00211000	0.80303500
H	-2.47090300	0.00087400	-1.72769800
Ga	-2.52381800	-0.00127300	-0.17011300

34

TS3 (Ga)

O	0.92779600	-0.29650700	0.56982900
N	-0.23300200	-0.06927100	-0.18244400
C	-0.75701900	1.29328600	0.07343900
C	-0.99225500	1.61731300	1.55568100
H	-1.08820000	2.70568300	1.68778100

H	-0.13678500	1.27250600	2.15292500
H	-1.90198500	1.15641800	1.95881100
C	0.25798700	2.30139100	-0.46911700
H	0.56587300	2.03770300	-1.49119900
H	1.15475100	2.36447400	0.16423200
H	-0.19331800	3.30474800	-0.48942400
C	-2.04672000	1.44896000	-0.74420100
H	-2.47760100	2.43817700	-0.52053100
H	-1.77515900	1.45365600	-1.81378000
C	-3.05369000	0.34142300	-0.49743000
H	-3.93653600	0.48050700	-1.14148900
H	-3.42534600	0.38089200	0.53987000
C	-2.40353600	-1.00012500	-0.77940700
H	-2.14075400	-1.05235800	-1.84987600
H	-3.09822200	-1.83196400	-0.58034400
C	-1.12768100	-1.23336800	0.03856900
C	-1.45359200	-1.50520600	1.51468100
H	-2.19372400	-0.81031100	1.92977100
H	-0.53604700	-1.43756000	2.11463100
H	-1.86149200	-2.52157600	1.62208800
C	-0.42323900	-2.47248500	-0.51769400
H	-0.12779900	-2.32051300	-1.56500100
H	-1.10542300	-3.33480300	-0.47590300
H	0.47200400	-2.71739900	0.07005600
H	0.86847700	-0.43983400	-2.10945500
H	1.45852200	-0.62558100	-2.57224600
H	3.02703300	1.00912300	-1.09264600
H	3.55792900	-0.99462300	0.79539600
Ga	2.61615900	-0.07766300	-0.05362300

32

TS3’ (Ga)

O	0.94435300	-0.00106300	0.95881700
N	-0.08843800	-0.00032500	0.04697600
C	-0.84205100	1.29223400	0.09236400
C	0.12949000	2.41588700	-0.26967500
H	-0.43278700	3.35637300	-0.36286400
H	0.62882600	2.22149900	-1.23038000
H	0.89318400	2.55452500	0.50836700
C	-1.40180400	1.58374700	1.48676600
H	-2.29423100	0.99458400	1.72837200

H	-1.68031800	2.64641300	1.54858900
H	-0.63000200	1.38246100	2.24162000
C	-1.94418600	1.23927500	-0.97106000
H	-2.54348300	2.15955100	-0.88652800
H	-1.46679200	1.26338600	-1.96601800
C	-2.81675500	0.00160100	-0.87303000
H	-3.56496800	0.00190100	-1.68092700
H	-3.38762100	0.00290300	0.06995100
C	-1.94707000	-1.23815300	-0.96962800
H	-1.47097900	-1.26513800	-1.96511500
H	-2.54812200	-2.15706800	-0.88270100
C	-0.84364600	-1.29208100	0.09246400
C	-1.40124000	-1.58299600	1.48784100
H	-2.29470400	-0.99540600	1.72957000
H	-0.62870200	-1.37867100	2.24119100
H	-1.67734100	-2.64614200	1.55173700
C	0.12634200	-2.41644000	-0.27145600
H	0.89218500	-2.55457400	0.50449400
H	0.62305100	-2.22293800	-1.23369700
H	-0.43659000	-3.35677600	-0.36227400
H	0.86253300	-0.00084800	-1.07335600
H	3.81310200	0.00263300	0.61844100
Ga	2.45333900	-0.00052700	-0.25565700

34

IN10 (Ga)

O	-0.88405600	-0.00000200	0.79185800
N	0.18934000	-0.00000100	-0.03842000
C	0.96445200	1.31727500	0.06895500
C	0.96445600	-1.31727400	0.06895500
C	2.15938700	1.24101500	-0.88438700
C	1.37072500	1.58272900	1.51093500
C	0.00131800	2.40599900	-0.39134400
C	2.15939000	-1.24101100	-0.88438800
C	1.37072800	-1.58273000	1.51093400
C	0.00132200	-2.40599900	-0.39134600
C	3.01394300	0.00000300	-0.69672000
H	1.78523300	1.26755200	-1.92335500
H	2.74972100	2.16028900	-0.74809300
H	1.62609700	2.64722800	1.60801700
H	0.52705600	1.36625600	2.17873400

H	2.24307100	1.00261500	1.83251800
H	-0.84102000	2.51179200	0.30420900
H	0.54296600	3.36116600	-0.43845600
H	-0.40110200	2.19409000	-1.39383100
H	2.74972600	-2.16028400	-0.74809400
H	1.78523500	-1.26754800	-1.92335500
H	0.52705900	-1.36625800	2.17873300
H	1.62610100	-2.64722800	1.60801500
H	2.24307500	-1.00261600	1.83251800
H	0.54297300	-3.36116400	-0.43846400
H	-0.84101200	-2.51179800	0.30420900
H	-0.40110100	-2.19408700	-1.39383100
H	3.48831400	0.00000300	0.29776400
H	3.83795200	0.00000400	-1.42610300
H	-1.92846600	0.00002500	-1.63234600
H	-0.18857100	-0.00000300	-1.01209000
H	-3.33443500	1.35912200	0.24298700
H	-3.33441400	-1.35914400	0.24295700
Ga	-2.62231300	-0.00000100	-0.16413300

4

IN11 (Ga)

H	1.56797700	-0.10727200	0.00002900
H	-0.69121800	1.41140400	0.00002900
H	-0.87690300	-1.30434900	0.00002900
Ga	0.00000500	0.00000700	-0.00000300

S.3.1.5 Cartesian Coordinates of DFT Optimized Structures Involved in Benzene C−H Activation

101

INO (Ga)

Ga	-0.00882500	-0.09628300	0.15803000
O	-1.51388800	-0.14524100	-0.87401600
O	0.85838200	1.48250200	0.41947800
O	1.07515000	-1.56298400	-0.04120900
N	-2.64187200	-0.86037900	-0.43168800
N	0.30906600	2.68733500	-0.07489900
N	2.43335300	-1.34364500	-0.34485200
C	-2.88641800	-1.97564900	-1.37759800
C	-1.73270100	-2.96796700	-1.23047700
H	-1.88782700	-3.82778000	-1.89942500
H	-1.67410500	-3.34581000	-0.20062800
H	-0.76824400	-2.50561800	-1.47531500
C	-2.95145100	-1.54700400	-2.85140000
H	-3.89331700	-1.05056000	-3.11559500
H	-2.85092200	-2.42919100	-3.50144000
H	-2.12342700	-0.85892000	-3.06966100
C	-4.17650600	-2.68588900	-0.94691800
H	-4.39660400	-3.47825200	-1.68061900
H	-3.98464100	-3.19072400	0.01603600
C	-5.35547100	-1.74640200	-0.77630700
H	-6.23908400	-2.30215800	-0.42335800
H	-5.64102700	-1.30410100	-1.74501500
C	-4.98485600	-0.65846900	0.21465500
H	-4.79829400	-1.12099400	1.19979000
H	-5.80982700	0.05953400	0.35074500
C	-3.72882400	0.11851600	-0.19588100
C	-4.02027300	1.04893400	-1.38326300
H	-4.58218900	0.56033300	-2.18841300
H	-3.07525800	1.42488600	-1.79694100
H	-4.61448600	1.91041500	-1.04270700
C	-3.29575600	0.99509500	0.97862000
H	-2.41489800	1.59616300	0.70910000
H	-3.05450100	0.36765800	1.84777400
H	-4.10716500	1.68315200	1.25973500
C	0.79597800	2.92295500	-1.45668700
C	2.32076800	2.83742600	-1.61848600

H	2.57477500	2.74125900	-2.68532400
H	2.69641000	1.94768800	-1.09410200
H	2.84832000	3.71917300	-1.23409400
C	0.16666700	1.86269100	-2.35710000
H	-0.91530300	1.79846300	-2.18791300
H	0.59845700	0.86770800	-2.18568500
H	0.35632500	2.10652700	-3.41325700
C	0.25449400	4.28123400	-1.92344600
H	0.65797200	4.48321000	-2.92877500
H	-0.84004300	4.19047400	-2.03091900
C	0.55844800	5.41660200	-0.96668900
H	0.10504200	6.35364600	-1.32789200
H	1.64431400	5.60209300	-0.91672900
C	0.01539900	5.06037900	0.40321900
H	-1.08340100	4.97698400	0.34232300
H	0.23725900	5.84814500	1.14139200
C	0.56449900	3.73306200	0.94017100
C	2.03747100	3.87486000	1.35728600
H	2.64563000	4.41226800	0.61946600
H	2.47562800	2.87899100	1.50861200
H	2.10406800	4.43080700	2.30509200
C	-0.23340800	3.36944000	2.19048500
H	-1.27111200	3.12009800	1.92459700
H	-0.24480000	4.21904700	2.89012300
H	0.22154900	2.51421000	2.70242000
C	2.75326300	-2.07120200	-1.58998600
C	2.39940400	-3.56628800	-1.55803800
H	1.39887100	-3.69834200	-1.12409900
H	3.11201700	-4.16432200	-0.97618100
H	2.38829600	-3.97163800	-2.58121400
C	1.95637600	-1.41902300	-2.71938300
H	0.87482600	-1.54735900	-2.57516700
H	2.22837500	-1.87159200	-3.68489200
H	2.18033800	-0.34430100	-2.76654400
C	4.24278900	-1.85602500	-1.88982500
H	4.50623600	-2.44772800	-2.78157700
H	4.38553800	-0.79481300	-2.15589200
C	5.14725200	-2.18958100	-0.71841800
H	6.19640900	-1.96781400	-0.97184600
H	5.11009800	-3.26900700	-0.49528900
C	4.71541500	-1.38184200	0.49137200

H	4.85474200	-0.30872400	0.27603700
H	5.33554600	-1.61611600	1.37196400
C	3.24395600	-1.60255700	0.86265100
C	3.03711100	-2.98990000	1.49139200
H	3.42184200	-2.99391300	2.52300100
H	3.54884400	-3.79345000	0.94735300
H	1.96436700	-3.22600900	1.52238000
C	2.85524400	-0.55443000	1.90628400
H	2.81559700	0.44651900	1.45377100
H	3.58211100	-0.55517200	2.73267900
H	1.86691900	-0.77687100	2.32998200
O	-0.66238400	-0.68175900	1.99285200
C	-0.66900400	0.04149000	3.23936900
C	-1.22323900	-0.94551100	4.24961600
H	-1.28123900	0.94178100	3.11415600
H	0.36563900	0.33954700	3.46834000
C	-0.97561900	-2.08041700	2.19631000
C	-0.77782200	-2.28845700	3.68113300
H	-0.29672800	-2.65520300	1.55382100
H	-2.01496400	-2.23069700	1.86758800
H	0.28491900	-2.47349900	3.90507300
H	-1.36091100	-3.13817100	4.06152100
H	-2.32285900	-0.88784800	4.27690600
H	-0.84753400	-0.74905500	5.26307000

13

Tetrahydrofuran (THF)

O	-0.00002600	1.23654200	-0.00000600
C	1.15418400	0.42874300	-0.13433700
C	0.72582100	-0.98618900	0.23272600
H	1.95239700	0.82172500	0.51676900
H	1.52390700	0.47025000	-1.17766300
C	-1.15419600	0.42870300	0.13434600
C	-0.72578600	-0.98621400	-0.23273000
H	-1.95242700	0.82166100	-0.51675200
H	-1.52391400	0.47019200	1.17767500
H	-0.77649100	-1.13282200	-1.32406100
H	-1.34550300	-1.76042500	0.24195800
H	0.77652800	-1.13280600	1.32405500
H	1.34557100	-1.76037100	-0.24196400

88

IN1 (Ga)

Ga	-0.00247700	-0.00604200	-0.00011100
O	0.40516100	-1.75571300	-0.00013700
O	-1.71974000	0.52044800	-0.00087500
O	1.30981300	1.22054300	0.00045500
N	1.82182500	-1.86998500	0.00044000
N	-2.53731400	-0.64135100	0.00002200
N	0.70899000	2.50816300	-0.00002500
C	2.24403800	-2.46377300	-1.28075900
C	1.89948000	-1.45066600	-2.37620600
H	2.35743000	-1.74981000	-3.33086200
H	2.28135800	-0.45576200	-2.10019100
H	0.81346100	-1.38717200	-2.53941600
C	1.55851800	-3.79544300	-1.61544900
H	1.94510800	-4.63482200	-1.02344900
H	1.71335600	-4.04000800	-2.67729200
H	0.47805400	-3.71344800	-1.43312800
C	3.76983700	-2.61518900	-1.23680600
H	4.09369500	-3.12534900	-2.15820100
H	4.21454500	-1.60580700	-1.25424100
C	4.25968400	-3.34419500	0.00111600
H	5.36012600	-3.39470100	0.00159100
H	3.90711800	-4.38899500	0.00066900
C	3.76873000	-2.61587100	1.23900400
H	4.21337800	-1.60648000	1.25738600
H	4.09178000	-3.12652800	2.16040900
C	2.24288500	-2.46456600	1.28165100
C	1.55722100	-3.79655800	1.61481800
H	1.94509000	-4.63560700	1.02318100
H	0.47703200	-3.71493800	1.43074500
H	1.71042700	-4.04144000	2.67682500
C	1.89724300	-1.45213500	2.37738400
H	0.81107100	-1.38870700	2.53957900
H	2.27938700	-0.45706500	2.10232700
H	2.35428700	-1.75184000	3.33229800
C	-3.26267200	-0.70386800	-1.28163400
C	-4.05922000	0.56394400	-1.61879700
H	-4.35205500	0.55082900	-2.67963600
H	-3.43724600	1.45261100	-1.44261500
H	-4.97644200	0.66201100	-1.02417900

C	-2.21488700	-0.92485100	-2.37641200
H	-1.55458500	-1.76123100	-2.10029500
H	-1.60581500	-0.02342700	-2.53935500
H	-2.70542800	-1.16579800	-3.33134700
C	-4.17033100	-1.93968800	-1.23729600
H	-4.77414700	-1.95860700	-2.15881200
H	-3.52883700	-2.83715600	-1.25463100
C	-5.04778800	-1.98909200	0.00034200
H	-5.65198700	-2.91021100	0.00111800
H	-5.76708400	-1.15326300	-0.00099800
C	-4.17156000	-1.93680700	1.23873500
H	-3.53010300	-2.83424600	1.25876700
H	-4.77628800	-1.95358600	2.15969500
C	-3.26390900	-0.70092300	1.28111100
C	-4.06069800	0.56772600	1.61457300
H	-4.97733100	0.66447000	1.01882400
H	-3.43849600	1.45594600	1.43692800
H	-4.35459000	0.55709400	2.67514700
C	-2.21724200	-0.91948100	2.37744400
H	-1.55721000	-1.75705400	2.10433300
H	-2.70879700	-1.15750700	3.33259000
H	-1.60774300	-0.01801700	2.53842300
C	1.01997900	3.16766600	-1.28124700
C	2.51685400	3.22345000	-1.61537300
H	2.97432000	2.24039300	-1.43670700
H	3.05952200	3.96936300	-1.02073800
H	2.65408700	3.48202200	-2.67631900
C	0.30756700	2.37000500	-2.37720300
H	0.78636900	1.39333300	-2.54112300
H	0.34263800	2.91638900	-3.33157600
H	-0.74616200	2.21282300	-2.10094300
C	0.40308000	4.57143200	-1.23832600
H	0.68917300	5.10336100	-2.15990800
H	-0.69479800	4.46404900	-1.25628100
C	0.79764000	5.35649400	-0.00069300
H	0.30252800	6.34055400	-0.00105400
H	1.88126200	5.56077300	-0.00047300
C	0.40246400	4.57203100	1.23712500
H	-0.69542500	4.46467000	1.25459500
H	0.68811100	5.10440300	2.15859000
C	1.01933900	3.16828500	1.28102600

C	2.51605200	3.22425300	1.61584700
H	2.65277700	3.48315900	2.67677700
H	3.05896300	3.97001100	1.02124000
H	2.97364100	2.24116700	1.43768900
C	0.30637900	2.37115900	2.37700400
H	-0.74725900	2.21404800	2.10036400
H	0.34113900	2.91791600	3.33117500
H	0.78492600	1.39445300	2.54148100

100

Ga - Benzene Activation - IN2

Ga	0.02856600	-0.15096400	0.17844300
O	0.52331800	1.59212200	0.30890700
N	-0.31384200	2.60759100	-0.19694800
C	-0.30722700	3.72112800	0.77910600
C	0.07682300	2.90753800	-1.59662400
C	-1.20405000	4.83201300	0.21821600
C	1.08972500	4.25634000	1.13336000
C	-0.93791600	3.21055800	2.07164900
C	-0.83236600	4.03789300	-2.09460300
C	1.56182100	3.24757500	-1.79446600
C	-0.22039500	1.66716100	-2.43306400
C	-0.82420300	5.25423100	-1.18847700
H	-2.24347600	4.46123700	0.21259900
H	-1.17219000	5.68552900	0.91470300
H	1.03549800	4.84182500	2.06371800
H	1.77867100	3.41637000	1.29694600
H	1.51383100	4.90968900	0.36153100
H	-0.27801100	2.48303100	2.55936000
H	-1.10094000	4.04973100	2.76470100
H	-1.90721500	2.73484500	1.86683300
H	-0.52638400	4.30041500	-3.12014600
H	-1.86088500	3.64288500	-2.15777400
H	2.18232700	2.52380700	-1.24793900
H	1.81534400	3.17394500	-2.86300400
H	1.82762300	4.25857400	-1.46395900
H	-0.13175500	1.90309000	-3.50391300
H	0.50676300	0.86942200	-2.22659800
H	-1.22816200	1.28381400	-2.23101700
H	0.16981300	5.73160000	-1.19302600
H	-1.52842100	6.01456800	-1.56251300

O	1.39828000	-1.35576900	0.10148100
N	2.54509400	-0.90255000	-0.58637000
C	3.63195700	-0.69704000	0.39816400
C	2.78574600	-1.80237200	-1.73614900
C	4.86648600	-0.22156800	-0.37654400
C	3.95548000	-1.93153600	1.25442700
C	3.19669200	0.42337400	1.34300100
C	4.05570600	-1.31409400	-2.44627300
C	2.88601900	-3.29028400	-1.36521200
C	1.61811500	-1.64170600	-2.70973400
C	5.24357300	-1.15074300	-1.51612400
H	4.64808000	0.78025600	-0.78470100
H	5.69938700	-0.10352100	0.33546600
H	4.50784500	-1.62334400	2.15527400
H	3.02093800	-2.41074500	1.57382100
H	4.56933300	-2.67571600	0.73198100
H	2.39178100	0.09038900	2.01383800
H	4.04679700	0.73065200	1.97089900
H	2.83067800	1.29055400	0.77773100
H	4.28292000	-2.01140900	-3.26897700
H	3.83065600	-0.33730900	-2.90742000
H	2.07370600	-3.55580000	-0.67462400
H	2.78401500	-3.90822900	-2.27000500
H	3.84040800	-3.55335800	-0.89362600
H	1.78755600	-2.25866600	-3.60479800
H	0.66634300	-1.95203000	-2.25778700
H	1.52773500	-0.59569000	-3.03207200
H	5.56612000	-2.13005900	-1.12479200
H	6.10621200	-0.74662600	-2.06982300
O	-1.47251400	-0.61542200	-0.75597100
N	-2.54943100	-1.31936400	-0.21950400
C	-3.75055500	-0.45096500	-0.29519400
C	-2.59585500	-2.67116100	-0.83045000
C	-4.94061800	-1.24647400	0.25201500
C	-4.05155500	0.08800400	-1.70090400
C	-3.49641200	0.74664700	0.61847700
C	-3.83915300	-3.39179800	-0.29176400
C	-2.57783200	-2.65536700	-2.36503300
C	-1.36635300	-3.43883600	-0.34304200
C	-5.11833400	-2.58851100	-0.43391500
H	-4.77818800	-1.41438200	1.33072100

H	-5.84484700	-0.62383800	0.15849200
H	-4.77113400	0.91748000	-1.63068700
H	-3.13177900	0.47140600	-2.16097400
H	-4.48317300	-0.66597100	-2.37013200
H	-2.65832500	1.35785600	0.25089900
H	-4.39223400	1.38410400	0.66151200
H	-3.27815000	0.40623300	1.64127500
H	-3.91718000	-4.36665100	-0.79947500
H	-3.67458800	-3.60424100	0.77859100
H	-1.81175400	-1.95309900	-2.72078100
H	-2.33265700	-3.65814600	-2.74539700
H	-3.54130600	-2.36439000	-2.80116100
H	-1.36585800	-4.45356000	-0.76769800
H	-0.42897300	-2.94542100	-0.63429500
H	-1.38645600	-3.53513100	0.75186700
H	-5.37521000	-2.44962900	-1.49705900
H	-5.96220800	-3.13766100	0.01318900
H	-1.56718700	-1.36301500	1.60053300
C	-0.77462000	-1.15546300	2.35490700
C	0.22354900	-2.10765600	2.62465300
H	0.32056100	-2.98613300	1.98621300
C	-0.88591800	-0.03150400	3.18837200
C	-0.00159700	0.15728500	4.24785900
C	0.98821800	-0.79243800	4.49921000
C	1.09679400	-1.92642200	3.69243400
H	-1.67589000	0.69705000	3.00543400
H	-0.08998000	1.04152400	4.88286500
H	1.68047900	-0.64975200	5.33263800
H	1.87164000	-2.66849400	3.89557200

100

Ga - Benzene Activation - TS1

Ga	-0.02759600	-0.27588600	-0.35956200
O	-0.74547300	1.35698000	0.01582200
N	0.00681100	2.41058800	0.56509800
C	-0.25099500	3.61319400	-0.26217000
C	-0.27484800	2.49086300	2.02091600
C	0.50217000	4.79314800	0.36298300
C	-1.73810100	3.95016000	-0.45319800
C	0.34465900	3.34886500	-1.64258900
C	0.46071100	3.71573200	2.58134200

C	-1.77030600	2.52258900	2.37092100
C	0.33771200	1.25802600	2.68214800
C	0.16827700	4.99832100	1.82787800
H	1.58482300	4.60023300	0.26718300
H	0.28410100	5.69586300	-0.23048600
H	-1.85367100	4.65605200	-1.28972000
H	-2.29788200	3.03663200	-0.69293100
H	-2.19321700	4.41220000	0.43115500
H	-0.19534900	2.54139500	-2.15395300
H	0.28058000	4.25539900	-2.26330600
H	1.40323100	3.06682500	-1.54921200
H	0.20501000	3.80934500	3.64919400
H	1.54477000	3.51444000	2.53043700
H	-2.30272400	1.77260900	1.76929300
H	-1.90794500	2.27618500	3.43486400
H	-2.23453400	3.50174300	2.19893300
H	0.27187100	1.35144100	3.77685000
H	-0.18576200	0.33686200	2.39937700
H	1.39157800	1.15020500	2.39714500
H	-0.88936000	5.28624600	1.94723000
H	0.75950000	5.82984700	2.24411300
O	-1.30943700	-1.58946400	-0.35323000
N	-2.58789300	-1.24331700	0.11495600
C	-3.50354800	-1.12435000	-1.03567200
C	-2.93340400	-2.13763000	1.23428700
C	-4.89015800	-0.76715100	-0.48637400
C	-3.56921700	-2.36916300	-1.93428000
C	-3.01089800	0.04925500	-1.88691700
C	-4.34164400	-1.76272100	1.71362100
C	-2.83677300	-3.63500000	0.90187500
C	-1.95279600	-1.83925700	2.36904100
C	-5.36408900	-1.72411100	0.59257600
H	-4.83719900	0.25081400	-0.06423600
H	-5.60034900	-0.72962600	-1.32845800
H	-4.01972700	-2.10716600	-2.90409200
H	-2.55312100	-2.74352400	-2.11793200
H	-4.16577500	-3.18306500	-1.50269300
H	-2.10104400	-0.21289200	-2.44675900
H	-3.77994000	0.33146700	-2.62216800
H	-2.78635500	0.91219700	-1.24420100
H	-4.64079500	-2.46823100	2.50595800

H	-4.28754100	-0.76285600	2.17705400
H	-1.89481200	-3.83358700	0.37190600
H	-2.84405300	-4.22836100	1.82904400
H	-3.66592700	-3.99012800	0.27690200
H	-2.22041200	-2.41773800	3.26639800
H	-0.92369200	-2.09992700	2.08416700
H	-1.98601500	-0.77022100	2.62242200
H	-5.51675300	-2.73291200	0.17356400
H	-6.34443700	-1.40553500	0.98223100
O	1.44550000	-0.82986100	0.68178300
N	2.50722400	-1.24200500	-0.08058000
C	3.67259800	-0.30632900	0.14059800
C	2.75448400	-2.71688900	0.13569500
C	4.85298900	-0.80751600	-0.69648400
C	4.04019100	-0.17979900	1.62223000
C	3.26639500	1.07842800	-0.35472800
C	3.98160700	-3.12117000	-0.68827400
C	2.91411600	-3.06077800	1.61868400
C	1.54582600	-3.48537400	-0.39085500
C	5.20368400	-2.25990100	-0.43208300
H	4.59786500	-0.68859900	-1.76404900
H	5.71067700	-0.14392700	-0.50320700
H	4.63107300	0.73634300	1.76379800
H	3.13042800	-0.09968700	2.23013900
H	4.64136500	-1.01523600	1.99679600
H	2.35702200	1.45620200	0.13863000
H	4.08278400	1.78453100	-0.14691800
H	3.11661400	1.07436400	-1.43987000
H	4.18770500	-4.18412900	-0.48592900
H	3.71601900	-3.05230800	-1.75748300
H	2.12605300	-2.56719500	2.20256800
H	2.80724600	-4.14783200	1.74280200
H	3.88788500	-2.77952600	2.03308000
H	1.66653400	-4.55019600	-0.14754400
H	0.60534600	-3.13327700	0.05640300
H	1.47501300	-3.40665300	-1.48187400
H	5.56570800	-2.39232200	0.60045900
H	6.03118100	-2.57496600	-1.08663600
H	1.86346500	-1.01015300	-1.30619500
C	0.93396400	-0.68477700	-2.26239300
C	0.19574600	-1.72850700	-2.87521300

H	-0.14810900	-2.56514000	-2.26760500
C	1.27433100	0.39970700	-3.10286100
C	0.92182000	0.44189700	-4.44869900
C	0.20356200	-0.61099600	-5.01341900
C	-0.16266700	-1.69705700	-4.21856900
H	1.82610300	1.24705300	-2.69847800
H	1.20578100	1.30346900	-5.05761900
H	-0.07741600	-0.58170200	-6.06924400
H	-0.74225200	-2.51957700	-4.64442400

100

Ga - Benzene Activation - IN3

Ga	0.06072400	-0.17764100	0.44338500
O	1.05029600	1.35483100	0.26708200
N	0.47947800	2.62080300	0.05511800
C	0.73333900	3.44610000	1.26111100
C	0.97058300	3.12549500	-1.25186600
C	0.22734200	4.86862000	0.99337900
C	2.19853700	3.46688200	1.72238400
C	-0.10606900	2.85592800	2.39167600
C	0.48114000	4.56778900	-1.43763900
C	2.49379000	3.02477400	-1.42413000
C	0.31555800	2.27991300	-2.34138400
C	0.80851200	5.47549700	-0.26874000
H	-0.87205700	4.83235100	0.89738600
H	0.44987100	5.48672400	1.87839800
H	2.25854200	3.83741500	2.75713000
H	2.60717800	2.44855600	1.69383300
H	2.83533500	4.11235000	1.10469900
H	0.24156200	1.85168200	2.66812200
H	-0.04869000	3.49540800	3.28557100
H	-1.15874200	2.79134800	2.07977100
H	0.90235800	4.95440100	-2.37994900
H	-0.61502200	4.54352400	-1.56716100
H	2.82883200	2.03079900	-1.09640600
H	2.75857200	3.15353800	-2.48481400
H	3.04330400	3.78417700	-0.85351100
H	0.61304700	2.64710200	-3.33572800
H	0.60521400	1.22689500	-2.25729200
H	-0.77725200	2.33948600	-2.25925300
H	1.89908300	5.60412200	-0.17011100

H	0.39579200	6.48334500	-0.43752300
O	1.13525300	-1.63701300	0.06601000
N	2.46259700	-1.40298600	-0.32598000
C	3.35740000	-1.72067100	0.80122000
C	2.69031200	-2.03549600	-1.63543800
C	4.79729000	-1.46545300	0.33901000
C	3.20514400	-3.14515800	1.35962100
C	3.03347300	-0.73164500	1.92457300
C	4.15303300	-1.79183200	-2.02982600
C	2.34199400	-3.53216900	-1.68436400
C	1.80340300	-1.30481800	-2.64416100
C	5.14036200	-2.19231800	-0.94897700
H	4.91766500	-0.37995500	0.18206200
H	5.48274200	-1.75073100	1.15388600
H	3.64881400	-3.20611300	2.36545100
H	2.13852400	-3.39702100	1.43711600
H	3.69319200	-3.90668400	0.73748800
H	2.08102900	-0.97748700	2.41673300
H	3.82088000	-0.75823500	2.69345600
H	2.95617500	0.28584000	1.51517100
H	4.35453200	-2.32317100	-2.97459600
H	4.27303900	-0.71452100	-2.23574300
H	1.37053900	-3.69723100	-1.19810600
H	2.27075400	-3.87205400	-2.72917200
H	3.08827600	-4.16154400	-1.18279500
H	1.96984500	-1.69859300	-3.65874700
H	0.74159400	-1.42016200	-2.38788300
H	2.04139100	-0.23183100	-2.64192300
H	5.12023500	-3.28401400	-0.79236900
H	6.16852000	-1.94987000	-1.26336900
O	-1.23805300	-0.21415500	-1.01428200
N	-2.51452800	-0.59346300	-0.74885400
C	-3.48753300	0.53605500	-1.12077300
C	-2.79898300	-2.00071400	-1.30138400
C	-4.90350800	0.05430200	-0.79880700
C	-3.31598300	0.92550100	-2.58293000
C	-3.12153500	1.71938000	-0.23290600
C	-4.25857600	-2.33617000	-0.98413500
C	-2.47724800	-2.06782800	-2.78715300
C	-1.87946700	-2.95065600	-0.54530500
C	-5.25159500	-1.27987900	-1.43514300

H	-5.00831400	-0.02840800	0.29797600
H	-5.60068900	0.84584600	-1.11434200
H	-3.71080900	1.94161000	-2.71878200
H	-2.25232300	0.93290900	-2.84832000
H	-3.85491100	0.26769100	-3.27360900
H	-2.08085000	2.04572500	-0.38105800
H	-3.79236400	2.55724000	-0.46866600
H	-3.26712600	1.47338200	0.82807600
H	-4.47680100	-3.31405400	-1.44016600
H	-4.35488900	-2.48006300	0.10677000
H	-1.51440800	-1.58038700	-2.98621600
H	-2.38928200	-3.12612200	-3.06907000
H	-3.24645800	-1.61877300	-3.42467200
H	-2.07748300	-3.97510100	-0.89042200
H	-0.81790200	-2.71847300	-0.71413400
H	-2.07232800	-2.91865900	0.53656800
H	-5.26097100	-1.19711300	-2.53364000
H	-6.27032300	-1.57791500	-1.14472800
H	-2.61755700	-0.70334700	0.27944300
C	-0.98029400	-0.74653100	2.05616300
C	-0.65826600	-2.02742900	2.54665200
H	0.12349800	-2.59840800	2.03841200
C	-1.98412300	-0.04421200	2.74337900
C	-2.65095000	-0.59210800	3.84275700
C	-2.31613700	-1.86538600	4.29791700
C	-1.30844700	-2.58017900	3.64935700
H	-2.24679400	0.97115300	2.44146500
H	-3.42729800	-0.01397100	4.35122900
H	-2.83140500	-2.29600800	5.16043400
H	-1.02713900	-3.57501900	4.00537000

12

Ga - Benzene Activation - Benzene

C	0.98322200	-0.98974400	0.00000000
C	-0.36563800	-1.34632500	-0.00010800
C	-1.34876200	-0.35662300	0.00008300
C	-0.98314400	0.98981600	-0.00001800
C	0.36553200	1.34635100	-0.00007900
C	1.34879100	0.35652700	0.00008100
H	1.75307100	-1.76490600	0.00006800
H	-0.65180100	-2.40068200	-0.00006000

```
H        -2.40500400   -0.63569700    0.00018400
H        -1.75318500    1.76478000    0.00000800
H         0.65193800    2.40063200   -0.00003700
H         2.40497400    0.63586000    0.00007200
```

S.3.2 Calculated Fluoride and Hydride Ion Affinities

Calculated fluoride (FIA) and hydride ion affinities (HIA) for **1**, **2**, **3**, $B(C_6F_5)_3$, $Al(C_6F_5)_3$, $Ga(C_6F_5)_3$, and $In(C_6F_5)_3$ were computed from isodesmic reactions using the enthalpy change of Me_3SiF to Me_3Si^+ and F^- or Me_3SiH to Me_3Si^+ and H^- as an anchor, as previously described (depicted in Fig. S.85).[29,30] All calculations described in this section were performed using ORCA 5.0.0.[31,32] Geometry optimizations were performed using the PBEh-3c functional[33] with the def2-mSVP basis and frequencies were computed at the same level of theory to determine whether the structures are minima (with no imaginary frequency). The cartesian coordinates of the structures are provided in section. S.3.2.1. The following geometry optimization ORCA input file was used (file was modified to account for charge):

```
!PBEh-3c def2-mSVP def2/J RIJCOSX TightSCF
!Opt Freq
%PAL nprocs 8 end
* xyzfile 0 1 file.xyz
```

Single point energy calculations were performed on the optimized geometries using the RI-DSD-BLYP functional[34] with Grimme's D3 dispersion correction[20] and Becke-Johnson damping[21] with the def2-QZVPP basis set. The following ORCA input file was used (file was modified to account for charge):

```
!RI-DSD-BLYP D3BJ def2-QZVPP def2/J def2-QZVPP/C RIJCOSX TightSCF
%PAL nprocs 8 end
* xyzfile 0 1 file.xyz
```

The obtained single point energies were combined with the thermal correction from the

preceding geometry optimization calculation and combined to obtain the total enthalpies of the Lewis acids and the corresponding fluoride adducts. FIA and HIA values were obtained using the set of equations shown in Fig. S.85, where ΔH for eqn. 2 is 953 kJ/mol (X = F) and 924 kJ/mol (X = H; calculated at the CCSD(T)/CBS level of theory).[29,30]

$$\mathrm{LA + Me_3SiX \xrightarrow{\Delta H} [LA\text{-}X]^- + Me_3Si^+ \ (eqn.\ 1)}$$

$$\mathrm{Me_3SiX \xrightarrow{\Delta H} Me_3Si^+ + X^- \ (eqn.\ 2)}$$

$$\mathrm{LA + X^- \xrightarrow{FIA/HIA} [LA\text{-}X]^- \ (eqn.\ 1{-}2)}$$

X = F or H

Figure S.85: Isodesmic reaction for FIA and HIA computations using Me_3SiF and Me_3SiH.

S.3.2.1 Cartesian Coordinates of DFT Optimized Structures

```
88
Al-3TEMPO
 Al                 8.79323700    6.12095100    3.51570700
 O                  8.91634300    7.00749400    2.04990500
 O                  8.46927300    4.43530300    3.48903300
 O                  8.96784500    6.92911100    5.02009900
 N                  9.18908900    8.36553800    2.34754300
 N                  8.43189200    3.95947700    2.15614200
 N                  8.82856600    6.02516300    6.10169000
 C                  8.04841900    9.17991600    1.90090900
 C                  6.83572600    8.78667100    2.75119200
 H                  6.02928400    9.50713300    2.60406600
 H                  7.09164300    8.78550700    3.81230400
 H                  6.43153600    7.80959500    2.47954200
 C                  7.66189300    9.01726000    0.42358400
 H                  8.35168100    9.50869400   -0.26000600
 H                  6.67777000    9.45708500    0.25161900
 H                  7.60609000    7.96604500    0.14383800
 C                  8.37585500   10.64386900    2.22144100
 H                  7.56776000   11.27227400    1.83806800
 H                  8.38399500   10.76936200    3.30888500
 C                  9.71863200   11.08696100    1.66849700
 H                  9.92479600   12.11679700    1.96853000
 H                  9.70709600   11.09035500    0.57533700
 C                 10.80386200   10.16556200    2.19503900
 H                 10.86873600   10.28174400    3.28156200
 H                 11.78154800   10.43963800    1.79020200
 C                 10.54615000    8.68707500    1.87892400
 C                 10.81591300    8.38724300    0.39731100
 H                 10.35167400    9.10305700   -0.27820900
 H                 10.46289100    7.39285800    0.12827600
 H                 11.88991800    8.42088700    0.20559900
 C                 11.53549800    7.86772500    2.71478600
 H                 11.53454700    6.80904300    2.44870300
 H                 11.31989700    7.97084300    3.77974000
 H                 12.55215900    8.22720100    2.54605000
 C                  7.06219600    3.51448900    1.85660700
 C                  6.47512000    2.48231000    2.83097600
 H                  5.40243500    2.38307300    2.65444100
 H                  6.61119800    2.79467100    3.86547800
```

H	6.90689700	1.48915600	2.72291300
C	6.15601800	4.75018200	1.88195000
H	6.58510800	5.55926300	1.28832400
H	5.97130300	5.11069500	2.89590500
H	5.18081900	4.50471600	1.45795400
C	7.05750000	2.97093100	0.42368000
H	6.06093700	2.57826000	0.20479300
H	7.22621900	3.80202600	-0.26888000
C	8.12330900	1.91571700	0.18643800
H	8.10860200	1.59885100	-0.85887000
H	7.91833600	1.01606900	0.77313000
C	9.48266100	2.49669000	0.53129400
H	9.69979700	3.31302700	-0.16547700
H	10.27294500	1.75335400	0.39702400
C	9.56238300	3.03789600	1.96339700
C	9.64668100	1.88663400	2.97679100
H	8.90497900	1.10944200	2.80520500
H	9.52075800	2.24948000	3.99591800
H	10.62676600	1.41018700	2.91293200
C	10.85279700	3.85857200	2.07025700
H	10.79343600	4.75621500	1.45100200
H	11.70132200	3.26980000	1.71735500
H	11.08229000	4.14673200	3.09748100
C	7.59536500	6.35850200	6.83115800
C	7.48215000	7.81422300	7.30701500
H	7.71929400	8.51321100	6.50592600
H	8.12970000	8.04487400	8.15033100
H	6.45853100	8.01483900	7.62857200
C	6.41261600	6.07526500	5.89787900
H	6.30916200	6.82584000	5.11153800
H	5.47938500	6.09248800	6.46372900
H	6.50577700	5.08812100	5.44172000
C	7.48108300	5.39107900	8.01512300
H	6.60586300	5.67023000	8.60770400
H	7.28964400	4.38515800	7.62771400
C	8.73123800	5.35165800	8.87537300
H	8.61361500	4.61290400	9.67125100
H	8.88981100	6.30982400	9.37671300
C	9.92111300	4.99367700	8.00418400
H	9.78059000	3.98105800	7.61230000
H	10.84540500	4.97895900	8.58766400

C	10.10981200	5.95481700	6.82311700
C	10.66829600	7.30215400	7.30822200
H	11.71498800	7.18363600	7.59472600
H	10.14453400	7.69355600	8.17795000
H	10.62216500	8.05545700	6.52305500
C	11.14169400	5.32590800	5.88279700
H	10.74333000	4.42147700	5.41891200
H	12.03414300	5.04467800	6.44473100
H	11.47101900	6.01200100	5.10089100

89

Al-3TEMPO-F

Al	8.09230000	5.83022800	3.85360300
O	8.38879700	6.91765600	2.47926600
O	8.36587900	4.18525200	3.22310700
O	9.21296100	6.12350400	5.20740100
N	8.43141500	8.30950300	2.58258300
N	8.72664400	3.96729100	1.89575200
N	8.69621700	6.11158200	6.50614300
C	7.25518500	8.88093400	1.90081600
C	6.00445200	8.47066400	2.68171100
H	5.14728800	9.04931700	2.32625000
H	6.13138300	8.66622300	3.74613500
H	5.76461000	7.41733000	2.56485500
C	7.05589200	8.43576100	0.44001300
H	7.70136400	8.95622300	-0.26706200
H	6.02584900	8.62605300	0.12903400
H	7.24032600	7.36799500	0.33954600
C	7.35089500	10.40978900	1.98029700
H	6.50337900	10.84083900	1.43866000
H	7.24013500	10.70963700	3.02810700
C	8.66643800	10.95278000	1.45487200
H	8.70657800	12.03903000	1.57992500
H	8.75954300	10.77027400	0.38005200
C	9.80283500	10.29387300	2.21318100
H	9.74314000	10.59856000	3.26378500
H	10.77353200	10.63559400	1.84139300
C	9.76587700	8.76177200	2.15125500
C	10.20750000	8.26691700	0.76250900
H	9.72745800	8.79786500	-0.05859600
H	9.98797400	7.20561800	0.65145000

H	11.28506800	8.40444200	0.64608600
C	10.78737300	8.25326200	3.17006200
H	10.92431800	7.17642100	3.12059400
H	10.47246400	8.49800900	4.18233400
H	11.75767200	8.72263700	2.98656400
C	7.61560100	3.31619800	1.19551700
C	7.11809700	2.00104400	1.82265300
H	6.14951600	1.72950200	1.39628500
H	6.99085100	2.12409200	2.89717300
H	7.79056800	1.16101700	1.65337800
C	6.43757600	4.29607800	1.17970600
H	6.76984100	5.27820100	0.84494400
H	5.98291400	4.41075800	2.16222700
H	5.66456800	3.93978200	0.49328000
C	8.04416100	3.09476100	-0.26074100
H	7.24999500	2.55462700	-0.78531100
H	8.13360300	4.07341600	-0.74419100
C	9.37181800	2.36809300	-0.38891000
H	9.65819100	2.28760200	-1.44184100
H	9.28632600	1.34081800	-0.02128700
C	10.43216900	3.12998800	0.38670200
H	10.57405500	4.11001500	-0.08114100
H	11.39723800	2.61590800	0.34233800
C	10.05520200	3.34849100	1.85747100
C	10.20435100	2.03754800	2.65176300
H	9.76711900	1.17661600	2.14758800
H	9.73359000	2.13108300	3.62913300
H	11.26234400	1.81441500	2.80900100
C	11.04983700	4.35647300	2.43956600
H	10.92740200	5.32668600	1.95845100
H	12.07392200	4.01467100	2.26779800
H	10.91910700	4.49171500	3.51267200
C	8.83198000	7.44825400	7.09334200
C	10.26528400	8.01226500	7.13456400
H	10.77731900	7.81489000	6.19521900
H	10.87265800	7.59524000	7.93777200
H	10.23868400	9.09446700	7.28319200
C	7.96784200	8.40760800	6.26681700
H	8.38031700	8.60233100	5.27663400
H	7.87891400	9.36618600	6.78588500
H	6.96870400	7.99222200	6.13339100

C	8.23969200	7.40503200	8.50805100
H	8.38479800	8.38205600	8.97939300
H	7.15898300	7.25104700	8.42406900
C	8.82721700	6.29509500	9.36151800
H	8.34328400	6.27249800	10.34244600
H	9.88890300	6.47678400	9.55240200
C	8.63246500	4.97040400	8.64736300
H	7.55909700	4.76881000	8.56988000
H	9.06814800	4.14608000	9.22063700
C	9.22680900	4.95426600	7.23214100
C	10.76195800	4.85900200	7.28804900
H	11.06527000	3.85422300	7.59189000
H	11.21510900	5.55660400	7.99218300
H	11.18321800	5.05249100	6.30252400
C	8.70481000	3.69088100	6.54073700
H	7.63115700	3.76986200	6.36663500
H	8.88850900	2.81868100	7.17418900
H	9.18515000	3.51164200	5.58116400
F	6.50511100	5.89975500	4.42322600

89

Al-3TEMPO-H

Al	8.03221400	5.82011100	3.85789300
O	8.38268400	6.91768500	2.48838800
O	8.33960200	4.17889200	3.20342400
O	9.18315200	6.09982700	5.20396800
N	8.42126800	8.30776500	2.59701400
N	8.73435000	3.96463700	1.88580700
N	8.68158200	6.11207200	6.50590500
C	7.23400100	8.87763900	1.93581000
C	6.00006600	8.45058100	2.73492600
H	5.12644000	9.01472300	2.39636700
H	6.14293700	8.64342100	3.79846800
H	5.77963700	7.39112700	2.62365900
C	7.01414200	8.44002200	0.47560700
H	7.64972400	8.96525700	-0.23683900
H	5.97972600	8.63019900	0.17919800
H	7.19998000	7.37337000	0.36781800
C	7.32228400	10.40635400	2.02221800
H	6.46378400	10.83677600	1.49752600
H	7.22880100	10.70027500	3.07348500

C	8.62679200	10.95672600	1.47629800
H	8.66445300	12.04283400	1.60367700
H	8.70262400	10.77767800	0.39948300
C	9.77903600	10.30078300	2.21316200
H	9.73708400	10.60451200	3.26486100
H	10.74155400	10.64672300	1.82429600
C	9.74759900	8.76830800	2.15075800
C	10.17458200	8.27503400	0.75678100
H	9.67939300	8.80124300	-0.05854000
H	9.96095100	7.21187900	0.65144900
H	11.24940000	8.42031100	0.62537500
C	10.78370900	8.26492800	3.15714500
H	10.90834200	7.18622300	3.12078600
H	10.49026400	8.52734200	4.17150200
H	11.75433500	8.72358800	2.94913900
C	7.63915000	3.31919800	1.15765500
C	7.12832300	1.99872600	1.76297500
H	6.16912600	1.72995200	1.31393500
H	6.97921700	2.11297700	2.83563500
H	7.80479400	1.16056700	1.60059100
C	6.46243200	4.30033900	1.12826200
H	6.80194300	5.28468800	0.80787700
H	5.99443900	4.40873000	2.10618100
H	5.69916300	3.95075100	0.42759000
C	8.09723400	3.10954600	-0.29131600
H	7.31396000	2.57555400	-0.83825300
H	8.19974200	4.09235100	-0.76357400
C	9.42633700	2.38115300	-0.39573900
H	9.73574300	2.30833300	-1.44275000
H	9.33080600	1.35108300	-0.03839300
C	10.47102300	3.13457700	0.40902400
H	10.62585200	4.11698700	-0.04957500
H	11.43535600	2.61780100	0.38236500
C	10.06280800	3.34581400	1.87272000
C	10.19563100	2.03160100	2.66476600
H	9.76886000	1.17202200	2.14918000
H	9.70475400	2.12262300	3.63246200
H	11.25005000	1.80768800	2.84350000
C	11.04514800	4.35126800	2.47932700
H	10.93386100	5.32199900	1.99679900
H	12.07266600	4.00832700	2.33110100

H	10.88781100	4.48618300	3.54883800
C	8.86291800	7.44978500	7.08069300
C	10.31207600	7.97257100	7.09106500
H	10.80160700	7.73797400	6.14844300
H	10.91817400	7.55464000	7.89485500
H	10.32139500	9.05820500	7.21581600
C	8.01259500	8.42304900	6.25671200
H	8.41535400	8.59990500	5.25924800
H	7.94959900	9.38760700	6.76879800
H	7.00211300	8.02857800	6.13814700
C	8.29439000	7.43738400	8.50547500
H	8.47555800	8.41487000	8.96310600
H	7.20838400	7.31228000	8.44337900
C	8.86674400	6.32056100	9.36028000
H	8.39811300	6.31937800	10.34886000
H	9.93554200	6.47686700	9.53321000
C	8.62705800	4.99428100	8.66206300
H	7.54754900	4.81894500	8.60723900
H	9.05263500	4.16517100	9.23600600
C	9.19467500	4.94936400	7.23698800
C	10.72761200	4.81404000	7.26644300
H	11.00872200	3.80640800	7.58193600
H	11.21333200	5.51057300	7.94958900
H	11.13357900	4.97949500	6.26969000
C	8.62940200	3.69307300	6.56690800
H	7.55567500	3.79944600	6.40641800
H	8.79815000	2.82226500	7.20657900
H	9.09071400	3.49193600	5.60221400
H	6.50770300	5.87278100	4.38306100

88

Ga-3TEMPO

Ga	8.77574600	6.12097400	3.51568200
O	8.90528900	7.05321300	2.00513800
O	8.43989700	4.37461700	3.47444800
O	8.95919000	6.95471900	5.07602700
N	9.18663400	8.40684000	2.31291900
N	8.42238300	3.90490000	2.13915200
N	8.82665000	6.04110200	6.14995300
C	8.04642800	9.22875900	1.87827800
C	6.83555800	8.83787900	2.73321000

H	6.03573500	9.56781400	2.59626700
H	7.09603700	8.82617300	3.79340300
H	6.42009200	7.86752000	2.45570400
C	7.65098800	9.07731700	0.40197100
H	8.34044000	9.56852700	-0.28215700
H	6.66879000	9.52432200	0.23753400
H	7.58640900	8.02830600	0.11581200
C	8.38308800	10.68931900	2.20496100
H	7.57602100	11.32361700	1.82922600
H	8.39762800	10.80871900	3.29300600
C	9.72493700	11.12906700	1.64721100
H	9.93742900	12.15641500	1.95135700
H	9.70764400	11.13824900	0.55413300
C	10.80853500	10.20006200	2.16329700
H	10.87930200	10.30987500	3.25013400
H	11.78548700	10.47206000	1.75526000
C	10.54361700	8.72416200	1.84044300
C	10.80657100	8.43243000	0.35575700
H	10.33887400	9.15150700	-0.31396800
H	10.45390800	7.43911800	0.08239000
H	11.87971400	8.46802100	0.15939000
C	11.53578100	7.89745300	2.66654800
H	11.53450000	6.84104700	2.39280800
H	11.32732500	7.99319700	3.73387100
H	12.55114800	8.25956500	2.49499100
C	7.05734900	3.45621400	1.82188600
C	6.46026300	2.42302600	2.78942900
H	5.39044300	2.32013700	2.59824200
H	6.58049200	2.73641000	3.82559000
H	6.89647200	1.43112600	2.68783300
C	6.14551500	4.68824900	1.83148400
H	6.57776100	5.49840600	1.24139700
H	5.94276100	5.05016500	2.84116600
H	5.17879000	4.43520700	1.39267400
C	7.07349900	2.90940100	0.39019500
H	6.08090900	2.51344200	0.15951600
H	7.24887600	3.73925300	-0.30223000
C	8.14488500	1.85658100	0.16928500
H	8.14428500	1.53715100	-0.87536200
H	7.93492000	0.95770400	0.75543100
C	9.49773000	2.44258500	0.52968300

H	9.72069800	3.25834700	-0.16596200
H	10.29217100	1.70175800	0.40625100
C	9.55896900	2.98667700	1.96156500
C	9.63469200	1.83650700	2.97706000
H	8.89217800	1.06072600	2.80252400
H	9.50474500	2.20029700	3.99534400
H	10.61406800	1.35779700	2.91899300
C	10.84747100	3.80915600	2.08100900
H	10.79430600	4.70706600	1.46132300
H	11.69828300	3.22022900	1.73366700
H	11.06960600	4.09595000	3.10999100
C	7.59579600	6.36866100	6.88675600
C	7.48208900	7.82243100	7.36899500
H	7.70443400	8.52560900	6.56727700
H	8.13992700	8.05320800	8.20424800
H	6.46188700	8.01720600	7.70478400
C	6.40669900	6.08621600	5.96050600
H	6.29371200	6.84000000	5.17876000
H	5.47890900	6.09798000	6.53547700
H	6.49687000	5.10075100	5.49951300
C	7.49132300	5.39716300	8.06833400
H	6.61899100	5.67195200	8.66721900
H	7.29994200	4.39201500	7.67882100
C	8.74652000	5.35781900	8.92121600
H	8.63486000	4.61676000	9.71583000
H	8.90629200	6.31488200	9.42437000
C	9.93195500	5.00455700	8.04228200
H	9.79103900	3.99301100	7.64773000
H	10.85948000	4.98925500	8.62062100
C	10.11291600	5.97000200	6.86344200
C	10.67210900	7.31630900	7.35097700
H	11.71812300	7.19583000	7.63917100
H	10.14735800	7.70758500	8.22014800
H	10.62925900	8.07066900	6.56658200
C	11.14248400	5.34682900	5.91629300
H	10.74512500	4.44380900	5.44839400
H	12.03550900	5.06293000	6.47610400
H	11.47087100	6.03756500	5.13825000

89

Ga-3TEMPO-F

Ga	8.19978200	6.68677900	3.79806900
O	9.42683300	7.42608000	2.63167100
O	8.01503800	4.87441300	3.50500100
O	8.82114300	6.89188600	5.52975900
N	9.13451700	8.69614800	2.12134300
N	7.75477100	4.37842800	2.22275500
N	9.02381100	5.74090700	6.29321500
C	8.96006600	8.59381800	0.66722400
C	7.73166500	7.71494500	0.40943900
H	7.44554200	7.77570500	-0.64433700
H	6.89089400	8.05479700	1.01535900
H	7.91023300	6.66551000	0.64526200
C	10.14840500	7.98511100	-0.10000500
H	10.99521100	8.66331900	-0.19776100
H	9.83825000	7.70865700	-1.11053600
H	10.49839400	7.08562600	0.40110300
C	8.64510900	9.99499900	0.12996400
H	8.55743000	9.94259000	-0.95973200
H	7.66470500	10.29865500	0.51159600
C	9.67367100	11.03109900	0.54584500
H	9.38414000	12.02111300	0.18098100
H	10.64532700	10.81560100	0.09205700
C	9.77722300	11.04046900	2.06017800
H	8.82025700	11.37758500	2.47196500
H	10.53422000	11.75477000	2.39833100
C	10.09834800	9.66470700	2.65786600
C	11.58087700	9.30779800	2.44378200
H	11.92705900	9.46415800	1.42255900
H	11.75500300	8.26431600	2.70214900
H	12.20956000	9.92278700	3.09230600
C	9.86335800	9.76672200	4.16829800
H	10.20360400	8.88236500	4.70233200
H	8.80187300	9.89863900	4.38182900
H	10.40497000	10.62716000	4.57079500
C	6.38751900	3.82685600	2.19709300
C	6.06635200	2.80338100	3.30166100
H	4.98600400	2.65073800	3.35877400
H	6.40263500	3.16988500	4.26966500
H	6.51769500	1.82561300	3.13871400
C	5.39991900	4.98556400	2.35243700
H	5.62894500	5.78779400	1.65115700

H	5.40325400	5.40727700	3.35368700
H	4.38711400	4.62993700	2.14406000
C	6.13974700	3.20914500	0.81504100
H	5.14235100	2.75927600	0.80993600
H	6.12454300	4.01534900	0.07337500
C	7.19634200	2.19676800	0.41544300
H	7.00161700	1.81877000	-0.59256700
H	7.16690500	1.32496800	1.07616800
C	8.55561300	2.86674100	0.47449400
H	8.58994600	3.65882700	-0.28125000
H	9.35344900	2.16068800	0.22509100
C	8.86680900	3.48748900	1.84255600
C	9.20487600	2.38732800	2.86582600
H	8.49644000	1.55925000	2.86839600
H	9.23861800	2.80410600	3.87148300
H	10.18724600	1.96586500	2.64055800
C	10.11956200	4.34587000	1.65933600
H	9.91595900	5.17932500	0.99026600
H	10.92101300	3.74212800	1.22478100
H	10.48497800	4.75742100	2.59628400
C	8.02292900	5.69209900	7.36472000
C	7.96813600	6.93187100	8.27585100
H	7.97495000	7.83899000	7.67341900
H	8.79304100	6.98991000	8.98549300
H	7.04452800	6.92376200	8.85975400
C	6.64451700	5.54261100	6.71233100
H	6.32283800	6.45241600	6.20878100
H	5.89860300	5.30609200	7.47608900
H	6.65529400	4.73452200	5.98026600
C	8.27778800	4.42451700	8.18995800
H	7.58033200	4.40572200	9.03324700
H	8.04369800	3.55539900	7.56624200
C	9.71301000	4.30496000	8.67315000
H	9.85509800	3.35872100	9.20369300
H	9.94288300	5.09246300	9.39719500
C	10.64818000	4.38251600	7.47955300
H	10.46928900	3.51419100	6.83644700
H	11.69454800	4.33076100	7.79624800
C	10.44551200	5.64897100	6.63994000
C	11.03653500	6.87611500	7.35832200
H	12.12793700	6.82294000	7.34933500

H	10.72935200	6.96297800	8.39971000
H	10.74228700	7.79088100	6.84601500
C	11.22655600	5.46053100	5.33593200
H	10.78319500	4.65701300	4.74792800
H	12.26453100	5.19495600	5.55312600
H	11.23515100	6.36368400	4.72698600
F	6.64666000	7.54753900	3.75835300

89

Ga-3TEMPO-H

Ga	8.13915900	6.71120500	3.74882100
O	9.47861700	7.43453800	2.66221400
O	8.10565000	4.85927700	3.49869700
O	8.72170000	6.91349000	5.51404000
N	9.17949100	8.69129000	2.12840800
N	7.75182900	4.35757600	2.24248200
N	8.96545700	5.76815100	6.27064100
C	9.04836200	8.57953700	0.66968900
C	7.83483600	7.69044600	0.37527100
H	7.59332000	7.73388100	-0.69058100
H	6.96699400	8.03820300	0.93767400
H	7.99855600	6.64497200	0.63611200
C	10.26682400	7.97809800	-0.05455400
H	11.09691000	8.67667300	-0.15584300
H	9.98862400	7.66159700	-1.06281700
H	10.62844600	7.10564700	0.48460100
C	8.73320900	9.97218000	0.10904900
H	8.68100200	9.90854600	-0.98241300
H	7.73682300	10.26686400	0.45545400
C	9.73133500	11.02827800	0.54670300
H	9.43538200	12.01037400	0.16541600
H	10.71971900	10.82783600	0.12306400
C	9.78985400	11.04977500	2.06307200
H	8.81661000	11.37811300	2.44326600
H	10.52691000	11.77701800	2.41738100
C	10.11257600	9.68272000	2.67965700
C	11.60507300	9.34544700	2.50492300
H	11.97096400	9.49872500	1.49022500
H	11.78485900	8.30608400	2.77493000
H	12.21036800	9.97403700	3.16262000
C	9.83784300	9.79481500	4.18245900

H	10.15918900	8.91187500	4.72955300
H	8.77097000	9.92943200	4.36599700
H	10.36831500	10.65790600	4.59430100
C	6.39979900	3.77463600	2.32234100
C	6.19690400	2.72358000	3.43023100
H	5.13148800	2.60131100	3.63931100
H	6.68474200	3.04626300	4.34849800
H	6.58261800	1.73851000	3.17068600
C	5.40485000	4.90850200	2.58803200
H	5.57857600	5.74551700	1.91185600
H	5.46975700	5.28445200	3.60701300
H	4.38495300	4.54563300	2.43318200
C	6.04816000	3.17925300	0.95305900
H	5.06283300	2.70709400	1.01849600
H	5.95627500	3.99708200	0.22975900
C	7.08921000	2.19718700	0.44802300
H	6.82120700	1.83886300	-0.55048700
H	7.12470000	1.30966500	1.08708700
C	8.43713600	2.89192100	0.41481900
H	8.38935900	3.70421200	-0.31847800
H	9.22503400	2.21052000	0.07984800
C	8.84447800	3.48866900	1.76789500
C	9.27088100	2.37719600	2.74466000
H	8.55741800	1.55731900	2.81521300
H	9.41079300	2.78776100	3.74357400
H	10.22156900	1.94545400	2.42212800
C	10.07117500	4.36565900	1.51356200
H	9.81719100	5.19376100	0.85471400
H	10.85581500	3.77326800	1.03495100
H	10.47804300	4.78825200	2.42858300
C	7.97850700	5.68916600	7.35017800
C	7.91041900	6.91655500	8.27814800
H	7.90404400	7.83023600	7.68546000
H	8.73964800	6.97632300	8.98257100
H	6.99085300	6.88994200	8.86828300
C	6.59749600	5.52704000	6.70576600
H	6.26450800	6.44265200	6.21871400
H	5.85763200	5.26897800	7.46838300
H	6.61793400	4.73292900	5.95905500
C	8.26051400	4.41544500	8.15693600
H	7.57107200	4.37322800	9.00603800

H	8.03700100	3.55130400	7.52254600
C	9.70263400	4.31618400	8.62455300
H	9.86722300	3.36594000	9.14132100
H	9.92498300	5.09868200	9.35642700
C	10.62538500	4.42635400	7.42332900
H	10.45700700	3.56250600	6.77155600
H	11.67512100	4.39054900	7.73112900
C	10.39151500	5.69800900	6.59946300
C	10.96875600	6.92862500	7.32350700
H	12.06086300	6.89688600	7.30115800
H	10.67153600	7.00136200	8.36913700
H	10.65123500	7.84138600	6.82150900
C	11.16296300	5.53630500	5.28556900
H	10.71918100	4.74202900	4.68551500
H	12.20441000	5.27445700	5.49210800
H	11.16030800	6.44846500	4.69032600
H	6.77063000	7.46346800	3.54345800

88

In-3TEMPO

In	8.70883100	6.11866900	3.51470800
O	8.82885700	7.12699100	1.79925600
O	8.36233000	4.15588400	3.51585900
O	8.90759800	7.11081500	5.23206900
N	9.17258900	8.44974900	2.17031900
N	8.40710200	3.74390600	2.16233300
N	8.80526500	6.15583400	6.27137200
C	8.05614700	9.34258300	1.82639700
C	6.85668000	8.94055700	2.69314100
H	6.07889600	9.70358400	2.62920500
H	7.14694000	8.85730800	3.74345800
H	6.40025400	8.00407700	2.36718100
C	7.61560200	9.30135500	0.35579800
H	8.29657900	9.82749200	-0.31070100
H	6.63857600	9.77619800	0.24896500
H	7.52420600	8.27610700	0.00018300
C	8.45890600	10.76491700	2.23383400
H	7.66904100	11.45359800	1.92255200
H	8.50856800	10.81559900	3.32633400
C	9.80237000	11.18733200	1.66577200
H	10.06333100	12.18408500	2.02849100

H	9.75708900	11.26813800	0.57633700
C	10.86143800	10.18676600	2.09100100
H	10.96343800	10.22527900	3.18056900
H	11.83778600	10.44756200	1.67407800
C	10.52755900	8.74747700	1.68156100
C	10.74250100	8.54189900	0.17453700
H	10.29038300	9.32053100	-0.43714700
H	10.34008900	7.58370300	-0.15009900
H	11.81110300	8.54552100	-0.04918700
C	11.50511500	7.82677100	2.42092200
H	11.45445300	6.79533300	2.06889300
H	11.32677800	7.85478700	3.49896100
H	12.53039700	8.16225200	2.25469500
C	7.06106000	3.31578600	1.75394600
C	6.41794100	2.24074600	2.64280600
H	5.35887300	2.14501600	2.39623000
H	6.49003600	2.50786300	3.69604000
H	6.86207400	1.25503800	2.51793800
C	6.15235800	4.55027600	1.77824200
H	6.60489300	5.38212100	1.23314800
H	5.91229400	4.86977900	2.79427500
H	5.20129500	4.32098700	1.29447600
C	7.14483800	2.83662000	0.30030800
H	6.16481000	2.45383300	0.00378100
H	7.35552500	3.69698200	-0.34333200
C	8.22523800	1.79236400	0.08329300
H	8.27319900	1.51892700	-0.97317000
H	7.98911100	0.86956100	0.62043100
C	9.55942100	2.35929500	0.53168700
H	9.81387200	3.20613700	-0.11466600
H	10.35961800	1.62409600	0.41101200
C	9.55284900	2.83712000	1.98798500
C	9.59075400	1.64219600	2.95238500
H	8.86149400	0.87137500	2.71098900
H	9.41208900	1.96071300	3.97816200
H	10.57446700	1.16997600	2.91780000
C	10.82955600	3.65848400	2.19960300
H	10.80343500	4.57067400	1.59776900
H	11.70196600	3.08525700	1.88118300
H	10.99404100	3.91902900	3.24597200
C	7.58176000	6.42934900	7.03954700

C	7.46034800	7.85545400	7.59822100
H	7.66187800	8.59799800	6.82714100
H	8.13274700	8.04879400	8.43085900
H	6.44499600	8.02354800	7.96216400
C	6.38598200	6.18664500	6.11124300
H	6.26172100	6.98035600	5.37188800
H	5.46251400	6.15783700	6.69221300
H	6.47786900	5.22533100	5.59898900
C	7.50241100	5.39468800	8.16796400
H	6.63634400	5.62485600	8.79453300
H	7.31652500	4.40951200	7.72760800
C	8.76959000	5.32391300	9.00175000
H	8.67619300	4.53685500	9.75350500
H	8.92072100	6.25230900	9.55935200
C	9.95147900	5.03797400	8.09366700
H	9.82455200	4.04463400	7.65069600
H	10.88484500	5.01018800	8.66233200
C	10.10066400	6.06584300	6.96449400
C	10.64893300	7.39348000	7.51196200
H	11.70047500	7.27490200	7.78038900
H	10.13067400	7.73221700	8.40668700
H	10.58360100	8.18406000	6.76600800
C	11.12565700	5.51029000	5.97030600
H	10.73889300	4.61991700	5.46794000
H	12.03419100	5.21585500	6.49856200
H	11.42492400	6.24771500	5.22428100

89

In-3TEMPO-F

In	7.62522200	5.62606600	3.85976500
O	7.56741200	6.95912400	2.29566100
O	8.01629900	3.75036100	3.08696800
O	9.10041200	6.02747300	5.22746100
N	8.02477000	8.26037700	2.49183800
N	8.87215700	3.77219900	1.97665900
N	8.68708600	6.22841300	6.54821900
C	6.90528400	9.18530200	2.26125000
C	5.84622400	8.91382300	3.33327800
H	5.07322100	9.68582100	3.29509000
H	6.28692100	8.93083700	4.32924100
H	5.36083100	7.94941800	3.19941200

C	6.20903400	9.04892500	0.89415900
H	6.77540000	9.47702000	0.06827900
H	5.24475500	9.56155100	0.91861400
H	6.02308000	7.99941700	0.67114400
C	7.42270900	10.61440100	2.46845600
H	6.61981600	11.31959300	2.23177800
H	7.65381200	10.74721900	3.53073000
C	8.67035600	10.92127100	1.65810000
H	9.03284600	11.92723200	1.88965600
H	8.44424600	10.92215100	0.58768300
C	9.74079800	9.89038800	1.97393100
H	10.02672600	9.98609900	3.02714400
H	10.64474300	10.07380900	1.38474800
C	9.26560400	8.45452900	1.72718200
C	9.18496600	8.16809800	0.21523800
H	8.66662500	8.93704100	-0.35530800
H	8.68768100	7.21616700	0.03289300
H	10.19540400	8.09367700	-0.19416200
C	10.31726200	7.49744000	2.29348100
H	10.12986400	6.47159100	1.97444400
H	10.33021400	7.52266100	3.38386200
H	11.30975900	7.77788600	1.93162800
C	8.12404600	3.33724700	0.78554400
C	7.48861700	1.93700000	0.87923400
H	6.73933500	1.81598500	0.09321300
H	6.98744900	1.81946300	1.83900800
H	8.20195400	1.12213700	0.76494500
C	6.98278600	4.32772800	0.54165100
H	7.33627600	5.35646100	0.58247200
H	6.18445400	4.21389500	1.27412000
H	6.55050800	4.14709300	-0.44574500
C	9.07363000	3.42477600	-0.41551000
H	8.54949800	3.07238700	-1.30928900
H	9.31456100	4.48001400	-0.58455700
C	10.36449100	2.65429700	-0.20649100
H	11.03370100	2.79474300	-1.06061100
H	10.16692200	1.57931700	-0.15250300
C	11.03439100	3.13811800	1.06762600
H	11.33971600	4.18069500	0.92767100
H	11.94688400	2.56912100	1.27201500
C	10.12095800	3.06359000	2.29889400

C	9.96071100	1.60513400	2.77061800
H	9.78351900	0.89727300	1.96174600
H	9.13466200	1.52467200	3.47613100
H	10.86921900	1.27896300	3.28257100
C	10.82604100	3.81434900	3.42954200
H	10.90931200	4.87902100	3.21548100
H	11.83729900	3.41923100	3.55888000
H	10.30593100	3.70939700	4.37737100
C	9.11983600	7.56272800	6.97599900
C	10.62186100	7.85813700	6.81023700
H	10.96838200	7.55094900	5.82465400
H	11.24518900	7.36297900	7.55445100
H	10.80125100	8.93156200	6.90813800
C	8.34594000	8.58010600	6.13589600
H	8.63543900	8.56691000	5.08533500
H	8.51115000	9.59104600	6.51872900
H	7.27699100	8.36778900	6.19345200
C	8.69411200	7.76230400	8.43581000
H	9.07341000	8.72789800	8.78448400
H	7.60124400	7.82324200	8.47151300
C	9.14263600	6.63710200	9.35079500
H	8.75997300	6.79929400	10.36288300
H	10.23282000	6.62102500	9.44238300
C	8.62884600	5.31804300	8.80112100
H	7.53439000	5.32402900	8.83492100
H	8.95943200	4.47952600	9.42174200
C	9.06911500	5.06176600	7.35408000
C	10.56214000	4.68802400	7.30817600
H	10.70536400	3.67120500	7.68185900
H	11.19085700	5.34143700	7.91213500
H	10.92943500	4.72279800	6.28453400
C	8.26915500	3.85988600	6.83849600
H	7.21499600	4.11788200	6.72558100
H	8.34362800	3.03170700	7.54831500
H	8.63303000	3.49171400	5.87950200
F	5.81505700	5.52053400	4.64344900

89

In-3TEMPO-H

In	7.89876300	5.79904500	3.80425300
O	8.37298500	6.96460300	2.13946100

O	8.21555600	3.83874300	3.20284700
O	9.34088700	6.14588800	5.25871300
N	8.38496700	8.33251900	2.43753500
N	8.67480400	3.69325500	1.89263400
N	8.78947400	6.18213200	6.54251800
C	7.20584600	8.98233700	1.84463200
C	5.95089600	8.39325000	2.49557500
H	5.08006400	8.99801100	2.22869200
H	6.03745200	8.38434500	3.58222100
H	5.75665900	7.37424100	2.16516900
C	7.04131100	8.80876600	0.32322200
H	7.68805300	9.45998000	-0.26261200
H	6.01334500	9.03548700	0.02951200
H	7.24843700	7.77807100	0.03744200
C	7.25145000	10.46750700	2.22437600
H	6.40340300	10.98047500	1.75957000
H	7.11209800	10.54446800	3.30820600
C	8.56208800	11.14109700	1.85716200
H	8.56579600	12.17569300	2.21305500
H	8.67696900	11.19831800	0.77054000
C	9.71265100	10.36872800	2.47792000
H	9.64299400	10.44851400	3.56805200
H	10.67453900	10.80494100	2.19093400
C	9.71008200	8.88084800	2.10470700
C	10.15864500	8.68467300	0.64481300
H	9.69082300	9.37557900	-0.05521600
H	9.93971300	7.66816100	0.31940100
H	11.23774400	8.83761500	0.56541800
C	10.74945200	8.18733200	2.98794900
H	10.92037600	7.15718700	2.68408500
H	10.43579200	8.17247700	4.02951800
H	11.70329000	8.71713800	2.91952200
C	7.61769500	3.09308800	1.07571500
C	7.06393200	1.75595200	1.59997000
H	6.12837600	1.50834800	1.09179300
H	6.86060000	1.83186800	2.66697900
H	7.74508300	0.92037500	1.44266400
C	6.45551100	4.09246000	1.02109200
H	6.82431000	5.08858800	0.77096800
H	5.92123300	4.15004500	1.96918500
H	5.73426000	3.79049900	0.25660700

C	8.14885200	2.93465000	-0.35434100
H	7.39186000	2.42632900	-0.95995800
H	8.28069700	3.93315000	-0.78439900
C	9.47765600	2.20109900	-0.41534500
H	9.83941300	2.16013100	-1.44711900
H	9.35959600	1.16080600	-0.09672800
C	10.48316600	2.92187700	0.46601700
H	10.65946700	3.91949800	0.05026100
H	11.44737800	2.40417000	0.46646700
C	10.00485300	3.08002200	1.91466200
C	10.10479300	1.74067200	2.66773600
H	9.68687700	0.89965400	2.11477100
H	9.58402800	1.80899800	3.62155700
H	11.15160400	1.50281000	2.87161300
C	10.94895400	4.07093100	2.60290000
H	10.83804300	5.06043400	2.15779400
H	11.98731400	3.75169400	2.47907800
H	10.75289400	4.15940700	3.67077700
C	8.92852700	7.53706700	7.09087500
C	10.36401300	8.09559800	7.10905600
H	10.86419600	7.87799600	6.16774500
H	10.97552900	7.68776100	7.91363000
H	10.34548700	9.18029800	7.23993900
C	8.06993500	8.47408100	6.23249300
H	8.47201400	8.62418200	5.23055200
H	7.99739400	9.45514300	6.71043100
H	7.06026400	8.07239300	6.12788900
C	8.33832100	7.54991400	8.50725600
H	8.49287500	8.54237500	8.94177300
H	7.25572300	7.40227600	8.43314600
C	8.91692900	6.46590600	9.39919700
H	8.43606700	6.48419400	10.38168500
H	9.98110100	6.64258500	9.58036500
C	8.70508200	5.11848700	8.73295400
H	7.62868500	4.92797700	8.66610200
H	9.13106800	4.30996200	9.33524700
C	9.29827500	5.04562800	7.31949700
C	10.83181900	4.93126700	7.38120800
H	11.11948500	3.94244400	7.74634300
H	11.29515700	5.66364300	8.04190900
H	11.25181200	5.06099600	6.38531500

C	8.76297700	3.76365700	6.67281100
H	7.68467900	3.83514900	6.52020100
H	8.95629700	2.90774500	7.32522500
H	9.22332800	3.55552800	5.70879800
H	6.28120000	5.94787900	4.41703200

13

Me3Si+

C	-3.32553300	3.95162200	0.30975800
Si	-2.41789600	2.39159600	-0.00441300
H	-3.90363800	4.23500500	-0.57643300
H	-2.67577700	4.78434400	0.57315600
H	-4.06283800	3.81505100	1.10653900
C	-0.58608400	2.37256200	0.03455900
H	-0.16160700	1.38052700	-0.10902800
H	-0.21680500	2.77972900	0.98057100
H	-0.18603100	3.03286700	-0.74250800
C	-3.34616100	0.85144900	-0.35491600
H	-2.96669700	0.36278800	-1.25673600
H	-4.41780100	1.00919800	-0.46340300
H	-3.18966400	0.13083100	0.45567200

14

Me3Si-F

F	-3.14275400	3.31878700	1.52138700
Si	-3.17398400	1.96490000	0.61533800
C	-4.60504800	2.11463400	-0.58222600
H	-5.55569000	2.24663100	-0.06422600
H	-4.69450100	1.21562300	-1.19539200
H	-4.48144300	2.95794500	-1.26256400
C	-3.41111800	0.50799700	1.76713000
H	-4.33759700	0.58576900	2.33734600
H	-2.59320800	0.41673200	2.48305400
H	-3.45470300	-0.42784700	1.20671300
C	-1.54520900	1.83177200	-0.29896600
H	-1.52258900	0.92799700	-0.91158700
H	-0.69604500	1.77802400	0.38316400
H	-1.37769100	2.67721800	-0.96716200

14

Me3Si-H

H	-3.13150900	3.23573000	1.46014500
Si	-3.17162300	1.99521200	0.63411300
C	-4.60877600	2.11999200	-0.57873600
H	-5.56046700	2.24180500	-0.05992400
H	-4.68575500	1.22114500	-1.19304800
H	-4.49409700	2.96844800	-1.25419400
C	-3.41385300	0.51191700	1.77254300
H	-4.34405300	0.58627400	2.33736200
H	-2.60172600	0.42241400	2.49533800
H	-3.45002000	-0.42017200	1.20613300
C	-1.54147700	1.83539500	-0.29862100
H	-1.52249600	0.93146700	-0.90990900
H	-0.69305200	1.78367800	0.38485400
H	-1.37267600	2.68287500	-0.96404700

34

B(C6F5)3

B	0.02067300	0.02550200	0.00950700
C	-0.27779000	-1.50470500	-0.00156700
C	-1.21491700	-2.05231100	-0.86482800
C	0.37120700	-2.37763700	0.85836300
C	-1.49434500	-3.40450200	-0.89015000
C	0.09831100	-3.73145100	0.88042100
C	-0.83614800	-4.24244400	-0.00527000
C	-1.16978700	1.02743100	0.03620400
C	-1.17248400	2.18661000	-0.72969300
C	-2.30059500	0.78743300	0.80767000
C	-2.24310800	3.05852800	-0.74967200
C	-3.37987500	1.64845400	0.82934300
C	-3.34878800	2.78475200	0.03861100
C	1.49289500	0.52634100	-0.01276600
C	1.90711000	1.62257200	0.73490600
C	2.46399100	-0.10770800	-0.77931200
C	3.21368800	2.06872100	0.73479400
C	3.77537400	0.32384900	-0.81922100
C	4.14776400	1.41456800	-0.05182400
F	-2.37628600	-3.90513200	-1.74213900
F	-1.84736900	-1.26442300	-1.72991000
F	1.25999600	-1.90106000	1.72535900
F	0.71236000	-4.54021100	1.73097000
F	-1.10130700	-5.53598100	-0.00609200

F	-0.13244700	2.46399900	-1.50848700
F	-2.22667500	4.14334200	-1.50904500
F	-4.37834800	3.61006400	0.03668400
F	-4.43604600	1.40130200	1.58914500
F	-2.35105300	-0.28627300	1.58848900
F	1.03652500	2.25578600	1.51311300
F	2.13225600	-1.14500900	-1.53959600
F	4.67390600	-0.28774400	-1.57595100
F	5.39905700	1.83258400	-0.07003000
F	3.58256600	3.10521700	1.47199000

34

Al(C6F5)3

C	0.25836100	1.89143000	-0.03343100
C	1.16588500	2.47510100	-0.89430700
C	-0.46745600	2.73510300	0.78332500
C	1.35672600	3.84218600	-0.95851000
C	-0.31376800	4.10776100	0.75768400
C	0.60631100	4.65549900	-0.12347400
C	1.55045900	-1.20396200	0.02400300
C	1.73028200	-2.18607100	-0.92703700
C	2.53143000	-1.06484100	0.98286900
C	2.84046200	-3.00835600	-0.94346200
C	3.65911300	-1.86234900	1.01279600
C	3.80526600	-2.83714100	0.03771300
C	-1.80499600	-0.76392000	0.01539800
C	-2.13187300	-1.87964600	0.76214300
C	-2.82551300	-0.16620600	-0.69964700
C	-3.41438900	-2.39240900	0.81061500
C	-4.12321600	-0.63986800	-0.68218800
C	-4.40980400	-1.76097700	0.08119000
F	4.58878300	-1.71537900	1.94499500
F	2.37622200	-0.12846700	1.92377500
F	2.99986800	-3.94131200	-1.87034100
F	4.87611900	-3.61119800	0.04333500
F	0.80147800	-2.33458200	-1.87609300
F	-1.34278900	2.20409700	1.64093300
F	-1.02015700	4.89910600	1.55142600
F	0.76946400	5.96587900	-0.16743900
F	2.22832700	4.38112200	-1.79829300
F	1.87612800	1.69073400	-1.70926300

F	-1.17920600	-2.47927400	1.47860100
F	-2.54909600	0.90385600	-1.44757700
F	-5.08595600	-0.04949500	-1.37479000
F	-5.64355800	-2.23168600	0.11329200
F	-3.70667000	-3.45965600	1.53901200
Al	-0.00228000	-0.03518900	0.00291500

34

Ga(C6F5)3

C	-0.12781600	-1.94368000	0.06352800
C	-1.03586800	-2.57570800	-0.76226500
C	0.67250500	-2.74115700	0.85661800
C	-1.15581300	-3.95135200	-0.81394000
C	0.58419400	-4.12061600	0.84154800
C	-0.33718800	-4.72093800	-0.00231800
C	-1.60330400	1.08445600	0.04420800
C	-1.68851400	2.22027400	-0.73670600
C	-2.71819200	0.73484600	0.78029700
C	-2.83383000	2.99144400	-0.79645900
C	-3.88284600	1.47832300	0.75463900
C	-3.93240600	2.61158900	-0.04190600
C	1.76275000	0.85671900	0.04682200
C	2.04479000	1.96144400	0.82512100
C	2.75951700	0.39647300	-0.78961800
C	3.27083500	2.59816900	0.78460400
C	3.99852300	1.00376500	-0.86639900
C	4.24712200	2.11084600	-0.07051900
F	-4.93940200	1.12978400	1.47369900
F	-2.67205600	-0.34854500	1.55649800
F	-2.89800800	4.07304500	-1.55868100
F	-5.03597900	3.33638600	-0.08247300
F	-0.64014600	2.58150000	-1.47764000
F	1.55291800	-2.16754200	1.67804600
F	1.35628600	-4.86970100	1.61477200
F	-0.43516400	-6.03814100	-0.03383300
F	-2.02769700	-4.53762100	-1.62087800
F	-1.81444200	-1.83755300	-1.55560000
F	1.11107600	2.42750400	1.65569000
F	2.51686500	-0.65877200	-1.56925800
F	4.93719600	0.55220200	-1.68479100
F	5.42451800	2.70768100	-0.12787100

F	3.52511200	3.65488600	1.54205100
Ga	0.01385800	-0.00045600	0.07491800

34

In(C6F5)3

C	-0.12656400	-2.09973300	-0.03902800
C	-1.09938400	-2.75755900	-0.75769300
C	0.72464000	-2.85495700	0.73621800
C	-1.24035200	-4.13208000	-0.71541200
C	0.62032600	-4.23179000	0.81130700
C	-0.37131600	-4.86492400	0.07824100
C	-1.77610200	1.18401700	0.20650700
C	-1.97917500	2.38439200	-0.44004600
C	-2.75806900	0.74381600	1.06781900
C	-3.12130300	3.13892900	-0.24347300
C	-3.91486300	1.46537300	1.29551300
C	-4.08914500	2.66929600	0.63109900
C	1.90257100	0.97448400	-0.30289200
C	2.25970100	2.07174700	0.44994300
C	2.82434100	0.48047400	-1.19967000
C	3.49830100	2.67365100	0.32635700
C	4.07349000	1.05092700	-1.35821800
C	4.40340600	2.15389600	-0.58611200
F	-4.84537500	1.03078800	2.13268900
F	-2.58715500	-0.41105000	1.71580400
F	-3.30465400	4.29149800	-0.87117400
F	-5.18793100	3.37556600	0.83275000
F	-1.05538800	2.83305000	-1.29175500
F	1.67184800	-2.24064500	1.45040000
F	1.43845500	-4.94801200	1.56880500
F	-0.48928100	-6.18035500	0.13660100
F	-2.18142600	-4.75364600	-1.41111800
F	-1.92936900	-2.04666900	-1.52631500
F	1.39250000	2.56363000	1.33760900
F	2.50134400	-0.57779400	-1.94713000
F	4.94924800	0.56709000	-2.22686500
F	5.59227400	2.71519200	-0.72107000
F	3.83260100	3.72489100	1.06038400
In	0.00175800	0.03273500	-0.06800500

35

B(C6F5)3-F

C	-1.58845500	-0.50195100	0.47389900
C	-1.94913700	-1.71146900	-0.09101000
C	-2.65305300	0.30168900	0.85867200
C	-3.26700300	-2.09588800	-0.29509600
C	-3.97893500	-0.04086800	0.67538500
C	-4.28831700	-1.25449700	0.08982100
C	1.10391600	-1.04699900	0.30975800
C	1.89622300	-1.85395000	1.10971700
C	1.38908700	-1.10419500	-1.04485900
C	2.91500100	-2.64817500	0.60134800
C	2.39174100	-1.88061000	-1.59214900
C	3.16642500	-2.65999600	-0.75466600
C	0.34165900	1.47040600	0.27064800
C	1.47360800	2.09237000	0.77737800
C	-0.30983700	2.17047700	-0.72996600
C	1.92521700	3.32829800	0.34714000
C	0.10820900	3.40825500	-1.19095700
C	1.23305200	3.99283300	-0.64555800
F	-3.55628100	-3.27528100	-0.85027300
F	-1.03001800	-2.60571100	-0.46949600
F	-5.56068600	-1.60652400	-0.09634500
F	-4.96388900	0.77632200	1.05466600
F	-2.42241100	1.48671600	1.42767200
F	-1.39362800	1.66759100	-1.32807600
F	2.21250400	1.49098600	1.71088700
F	-0.55784800	4.04151200	-2.15938700
F	3.02222600	3.88101400	0.87013700
F	1.65277500	5.18151600	-1.07953200
F	0.64978200	-0.39355200	-1.90392500
F	2.61747700	-1.89507200	-2.90772000
F	4.14127600	-3.42096600	-1.25364100
F	3.65758100	-3.40751700	1.41079400
F	1.71496500	-1.92609500	2.42721300
F	-0.04322000	0.04814400	2.27362500
B	-0.06135400	-0.02174900	0.87008900

35

Al(C6F5)3-F

C	-1.92145600	-0.60964500	0.56845200
C	-2.22658500	-1.82584100	-0.00532900

C	-2.99865900	0.21009200	0.83912800
C	-3.51941500	-2.21478900	-0.31790200
C	-4.30832600	-0.12862200	0.55124400
C	-4.56374800	-1.35497400	-0.03577000
C	1.33753900	-1.23802500	0.37288500
C	2.18152500	-2.04258500	1.10991800
C	1.53267300	-1.26153000	-0.99276000
C	3.17279900	-2.82348200	0.53614000
C	2.50608800	-2.01944900	-1.61755900
C	3.33355300	-2.80604900	-0.83664400
C	0.40939800	1.79101400	0.34364500
C	1.59171300	2.36630100	0.76322900
C	-0.26854500	2.47766800	-0.64270200
C	2.08690500	3.55734600	0.26175500
C	0.18167500	3.67170200	-1.18197300
C	1.36752900	4.21251400	-0.72027600
F	-3.77443500	-3.40018300	-0.87591900
F	-1.25605100	-2.70491800	-0.28101200
F	-5.81603500	-1.70732100	-0.32387600
F	-5.32415100	0.69062100	0.82963400
F	-2.79100700	1.39820300	1.41957800
F	-1.40926300	1.99010900	-1.14213000
F	2.32668900	1.74963700	1.69702800
F	-0.49909300	4.30599800	-2.13887900
F	3.23535700	4.07796400	0.69858000
F	1.81915500	5.36009300	-1.22496300
F	0.74240300	-0.51693900	-1.77910500
F	2.66129300	-2.00958000	-2.94293400
F	4.28320000	-3.54644100	-1.40771600
F	3.97222400	-3.59007000	1.28137000
F	2.06817400	-2.10029200	2.43888500
Al	-0.07820400	-0.01032900	1.12475400
F	-0.06626700	0.03886600	2.79742000

35

Ga(C6F5)3-F

C	-1.93758100	-0.60058000	0.55923300
C	-2.23770500	-1.82141600	-0.00571200
C	-3.01276600	0.21905100	0.83634700
C	-3.53162100	-2.21807100	-0.30340200
C	-4.32256200	-0.13144400	0.56139400

C	-4.57758000	-1.36220900	-0.01624400
C	1.34476900	-1.25079800	0.34442800
C	2.17313300	-2.05420300	1.09928900
C	1.55521600	-1.27509400	-1.01827100
C	3.16882900	-2.83914700	0.53811200
C	2.53423300	-2.03942400	-1.62642800
C	3.34798200	-2.82681000	-0.83233300
C	0.42676200	1.80125900	0.33380500
C	1.60395600	2.37969500	0.76211700
C	-0.25940700	2.48579600	-0.64730400
C	2.08505800	3.57999800	0.26812600
C	0.18015600	3.68844400	-1.17606000
C	1.36039900	4.23643300	-0.70909200
F	-3.78467700	-3.40725500	-0.85365100
F	-1.26392900	-2.69389000	-0.28741500
F	-5.83057200	-1.72203200	-0.29129400
F	-5.33976500	0.68446000	0.84337200
F	-2.80851500	1.41159500	1.40398800
F	-1.39367300	1.98865300	-1.15063200
F	2.34606900	1.76411000	1.68784500
F	-0.50589900	4.32306400	-2.12866900
F	3.22851000	4.10787100	0.70875300
F	1.80233100	5.39151500	-1.20482800
F	0.77945200	-0.52872100	-1.81620900
F	2.70677500	-2.03385700	-2.94950200
F	4.30199600	-3.57295500	-1.38824900
F	3.95619200	-3.60585700	1.29542400
F	2.04151800	-2.10870600	2.42463700
F	-0.06995400	0.03215100	2.83547500
Ga	-0.07848000	-0.01456100	1.06914400

35

In(C6F5)3-F

C	-2.10471000	-0.65003400	0.58404800
C	-2.39049800	-1.87022400	0.01575700
C	-3.17474600	0.17870000	0.83778500
C	-3.67968400	-2.26510400	-0.30218600
C	-4.48081200	-0.16932500	0.54079600
C	-4.72730700	-1.40280400	-0.03573200
C	1.47323600	-1.36285300	0.33124100
C	2.30530600	-2.16273100	1.08134500

C	1.66180800	-1.38532100	-1.03204100
C	3.29112400	-2.95284100	0.51114600
C	2.63133100	-2.15479200	-1.64985400
C	3.45143100	-2.94313200	-0.86254400
C	0.47660000	1.96656900	0.33746800
C	1.65015300	2.54602800	0.76404100
C	-0.21724400	2.63395100	-0.64658300
C	2.12719400	3.74341500	0.25926700
C	0.21724900	3.83379300	-1.18569700
C	1.39769400	4.38755600	-0.72357200
F	-3.92971200	-3.45422800	-0.85342900
F	-1.40488500	-2.73624400	-0.24655500
F	-5.97613200	-1.76037900	-0.33107100
F	-5.50151300	0.64974400	0.79891300
F	-2.96883800	1.37261900	1.40390700
F	-1.35294000	2.12208100	-1.13310600
F	2.38634400	1.93594400	1.69971900
F	-0.47042300	4.46134000	-2.14158000
F	3.26855500	4.28129600	0.69247100
F	1.83494200	5.54002900	-1.22896100
F	0.87984900	-0.63087700	-1.81723800
F	2.79091700	-2.15334600	-2.97440900
F	4.39603300	-3.69321900	-1.42851300
F	4.08663200	-3.72040000	1.25850800
F	2.18487200	-2.20318500	2.40931600
F	-0.06954000	0.02033900	3.09782200
In	-0.07363300	-0.01530300	1.13571500

B(C6F5)3-H

H	-0.03699200	0.00234100	2.05349100
C	-1.53579900	-0.60497800	0.47249200
C	-1.83412800	-1.80513400	-0.14913400
C	-2.64476700	0.11378500	0.89964500
C	-3.12881700	-2.26331800	-0.34672400
C	-3.95019400	-0.30023200	0.71931400
C	-4.19520900	-1.50526500	0.08732700
C	1.18260400	-0.99338800	0.35498700
C	1.89788300	-1.79427600	1.22937700
C	1.62822600	-1.01319700	-0.95675700
C	2.99075700	-2.55388900	0.84102200
C	2.71187900	-1.75541300	-1.38825100

C	3.40139900	-2.53164400	-0.47632300
C	0.23814600	1.47806400	0.32344800
C	1.24363700	2.21024400	0.93756200
C	-0.42200700	2.13970600	-0.69814300
C	1.56994500	3.50890300	0.58881600
C	-0.13141100	3.44030300	-1.07747900
C	0.87180700	4.13084700	-0.42768900
F	-3.35415000	-3.43216000	-0.95245400
F	-0.86494200	-2.61157800	-0.59666400
F	-5.44732100	-1.92903100	-0.09587800
F	-4.97595300	0.44020700	1.14719200
F	-2.47129500	1.28025900	1.52953000
F	-1.39294100	1.53192100	-1.38632900
F	1.96163900	1.66305900	1.92228400
F	-0.80263800	4.03450600	-2.06737900
F	2.54582000	4.16788400	1.21935600
F	1.16724300	5.38354000	-0.77980800
F	0.98486400	-0.30050100	-1.88631500
F	3.09722600	-1.73999000	-2.66659200
F	4.45075100	-3.25689400	-0.86720700
F	3.65268800	-3.30761700	1.72318600
F	1.55376500	-1.87845800	2.51616700
B	-0.05110800	-0.04511400	0.84430600

Al(C6F5)3-H

H	0.02653500	-0.12493700	-2.71738400
C	-1.82407800	0.70938700	-0.57336400
C	-2.07401800	1.91219900	0.05252100
C	-2.94033100	-0.03394000	-0.89913100
C	-3.35073800	2.36169400	0.35216200
C	-4.23561100	0.36535200	-0.62588400
C	-4.43586500	1.57771400	0.00957400
C	1.45291200	1.17170400	-0.37551300
C	2.27365400	1.97110900	-1.14333000
C	1.69869300	1.19360100	0.98211100
C	3.29060100	2.74752000	-0.61066800
C	2.70078900	1.94721400	1.56662400
C	3.50226800	2.72965800	0.75497400
C	0.31681100	-1.82496700	-0.27385500
C	1.43388000	-2.52648000	-0.67911300
C	-0.45305500	-2.44275100	0.69026300

C	1.78116500	-3.77263400	-0.18690100
C	-0.15380800	-3.68857500	1.21734500
C	0.97129800	-4.35580900	0.76982400
F	-3.55177100	3.53432300	0.95814100
F	-1.06252200	2.71805200	0.39763600
F	-5.67332600	1.98909100	0.28676800
F	-5.28792100	-0.38431300	-0.96072300
F	-2.78389900	-1.21011600	-1.52149500
F	-1.54338400	-1.83872400	1.17557700
F	2.24940900	-1.98843700	-1.59660600
F	-0.92494400	-4.25475800	2.14848200
F	2.87207900	-4.41558500	-0.61001900
F	1.27635100	-5.55644900	1.26264800
F	0.93547000	0.45926400	1.80152400
F	2.90761000	1.93848200	2.88524000
F	4.47605200	3.46642200	1.28962100
F	4.06563200	3.50996800	-1.38602600
F	2.10734700	2.02828700	-2.46892100
Al	-0.01129000	-0.01025600	-1.12784600

35

Ga(C6F5)3-H

H	0.06031700	-0.10546300	-2.70027100
C	-1.82357700	0.69683600	-0.58764200
C	-2.07043200	1.89771100	0.04200000
C	-2.93790800	-0.05154300	-0.90737100
C	-3.34668100	2.34093200	0.35353700
C	-4.23156500	0.34384200	-0.61982600
C	-4.43194200	1.55380800	0.01980200
C	1.45576700	1.18376900	-0.36709100
C	2.26645100	1.98886100	-1.13887700
C	1.71138600	1.19495300	0.98831100
C	3.28603200	2.76412900	-0.60950700
C	2.71736000	1.94890000	1.56663100
C	3.51005100	2.73868800	0.75387400
C	0.32967800	-1.81505400	-0.28269200
C	1.43936800	-2.53159400	-0.68138400
C	-0.45493100	-2.41698000	0.67889200
C	1.76474700	-3.78086200	-0.18096800
C	-0.17647300	-3.66592300	1.21008600
C	0.94143300	-4.35064600	0.77208200

F	-3.54601100	3.51178300	0.96359400
F	-1.05968200	2.70679400	0.38002900
F	-5.66878300	1.95919400	0.30935300
F	-5.28374600	-0.40981800	-0.94656000
F	-2.78654800	-1.22636000	-1.53203000
F	-1.53914800	-1.79733200	1.15688100
F	2.26899300	-2.01271400	-1.59621900
F	-0.96311500	-4.21841800	2.13647400
F	2.84880900	-4.44083900	-0.59590400
F	1.22637600	-5.55440200	1.26984900
F	0.96031900	0.45440100	1.81161800
F	2.93592000	1.93222000	2.88336100
F	4.48675100	3.47474500	1.28472000
F	4.05300000	3.53227400	-1.38741200
F	2.08904600	2.05481900	-2.46263200
Ga	0.00073200	0.00159900	-1.14045100

35

In(C6F5)3-H

H	0.11591400	-0.12584800	-3.02676700
C	-1.94523500	0.74431200	-0.64816300
C	-2.16879400	1.92242100	0.02630900
C	-3.05821500	-0.01328900	-0.93625100
C	-3.43310700	2.34028300	0.41131700
C	-4.34133500	0.35577000	-0.57443200
C	-4.52289000	1.54557600	0.10798100
C	1.59852300	1.29462900	-0.40543500
C	2.41370600	2.11574700	-1.15042100
C	1.80954300	1.29637400	0.95527900
C	3.40378700	2.90535900	-0.58857200
C	2.78580500	2.06399100	1.56681000
C	3.58726900	2.87316500	0.78139000
C	0.36811600	-1.96462400	-0.30869100
C	1.47876900	-2.70046500	-0.65499400
C	-0.46220200	-2.52753300	0.63465200
C	1.76753900	-3.94244300	-0.11542300
C	-0.22151000	-3.76689500	1.20499900
C	0.90233100	-4.47580900	0.82226500
F	-3.62034600	3.49085600	1.06249300
F	-1.14538600	2.72778700	0.33621700
F	-5.74869800	1.92681900	0.46799100

F	-5.40059700	-0.40115300	-0.86927500
F	-2.91758100	-1.16834800	-1.60207000
F	-1.55496100	-1.87750300	1.04970400
F	2.34284800	-2.21055600	-1.55627200
F	-1.04899100	-4.28981500	2.11257000
F	2.85196000	-4.63202200	-0.47769900
F	1.15220800	-5.67057800	1.35857100
F	1.04981600	0.53114900	1.74802700
F	2.96964200	2.04248800	2.88871400
F	4.53571200	3.62126200	1.34539500
F	4.17993500	3.69202000	-1.33836400
F	2.26851300	2.18085800	-2.47986200
In	0.03990500	-0.00167900	-1.30373600

References

[1] A. B. Pangborn, M. A. Giardello, R. H. Grubbs, R. K. Rosen and F. J. Timmers, *Organometallics*, 1996, **15**, 1518–1520.

[2] D. B. G. Williams and M. Lawton, *J. Org. Chem.*, 2010, **75**, 8351–8354.

[3] A. K. Kayser, P. T. Wolczanski, T. R. Cundari, M. M. Bollmeyer, K. M. Lancaster and S. N. MacMillan, *Chem. Commun.*, 2022, **58**, 9818–9821.

[4] G. R. Fulmer, A. J. M. Miller, N. H. Sherden, H. E. Gottlieb, A. Nudelman, B. M. Stoltz, J. E. Bercaw and K. I. Goldberg, *Organometallics*, 2010, **29**, 2176–2179.

[5] J. J. Fajardo, A. T. Barth, M. Morales, M. K. Takase, J. R. Winkler and H. B. Gray, *J. Am. Chem. Soc.*, 2021, **143**, 19389–19398.

[6] P. Erdmann and L. Greb, *Angew. Chem. Int. Ed.*, 2022, **61**, e202114550.

[7] A. Wu, E. A. Mader, A. Datta, D. A. Hrovat, W. T. Borden and J. M. Mayer, *J. Am. Chem. Soc.*, 2009, **131**, 11985–11997.

[8] P. N. Bartlett, D. Cook, C. H. K. de Groot, A. L. Hector, R. Huang, A. Jolleys, G. P. Kissling, W. Levason, S. J. Pearce and G. Reid, *RSC Adv.*, 2013, **3**, 15645–15654.

[9] *Bruker, Saint, V8.40B*, Bruker AXS Inc.: Madison, WI, USA, 2008.

[10] L. Krause, R. Herbst-Irmer, G. M. Sheldrick and D. Stalke, *J. Appl. Cryst.*, 2015, **48**, 3–10.

[11] G. M. Sheldrick, *Acta Cryst. A*, 2015, **71**, 3–8.

[12] G. M. Sheldrick, *Acta Cryst. C*, 2015, **71**, 3–8.

[13] P. Müller, *Crystallogr. Rev.*, 2009, **15**, 57–83.

[14] C. R. Groom, I. J. Bruno, M. P. Lightfoot and S. C. Ward, *Acta Cryst. B*, 2016, **72**, 171–179.

[15] D. Kratzert, *FinalCif, V136*, `https://dkratzert.de/finalcif.html`.

[16] G. M. Sheldrick, *CELL_NOW*, University of Göttingen: Germany, 2008.

[17] M. J. Frisch, G. W. Trucks, H. B. Schlegel, G. E. Scuseria, M. A. Robb, J. R. Cheeseman, G. Scalmani, V. Barone, G. A. Petersson, H. Nakatsuji, X. Li, M. Caricato, A. V. Marenich, J. Bloino, B. G. Janesko, R. Gomperts, B. Mennucci, H. P. Hratchian, J. V. Ortiz, A. F. Izmaylov, J. L. Sonnenberg, D. Williams-Young, F. Ding, F. Lipparini, F. Egidi, J. Goings, B. Peng, A. Petrone, T. Henderson, D. Ranasinghe, V. G. Zakrzewski, J. Gao, N. Rega, G. Zheng, W. Liang, M. Hada, M. Ehara, K. Toyota, R. Fukuda, J. Hasegawa, M. Ishida, T. Nakajima, Y. Honda, O. Kitao, H. Nakai, T. Vreven, K. Throssell, J. A. Montgomery, Jr., J. E. Peralta, F. Ogliaro, M. J. Bearpark, J. J. Heyd, E. N. Brothers, K. N. Kudin, V. N. Staroverov, T. A. Keith, R. Kobayashi, J. Normand, K. Raghavachari, A. P. Rendell, J. C. Burant, S. S. Iyengar, J. Tomasi, M. Cossi, J. M. Millam, M. Klene, C. Adamo, R. Cammi, J. W. Ochterski, R. L. Martin,

K. Morokuma, O. Farkas, J. B. Foresman and D. J. Fox, *Gaussian 16 Revision C.01*, 2016, Gaussian Inc. Wallingford CT.

[18] J. P. Perdew, K. Burke and M. Ernzerhof, *Phys. Rev. Lett.*, 1996, **77**, 3865–3868.

[19] C. Adamo and V. Barone, *J. Chem. Phys.*, 1999, **110**, 6158–6170.

[20] S. Grimme, J. Antony, S. Ehrlich and H. Krieg, *J. Chem. Phys.*, 2010, **132**, 154104.

[21] S. Grimme, S. Ehrlich and L. Goerigk, *J. Comput. Chem.*, 2011, **32**, 1456–1465.

[22] F. Weigend and R. Ahlrichs, *Phys. Chem. Chem. Phys.*, 2005, **7**, 3297–3305.

[23] E. Cancès, B. Mennucci and J. Tomasi, *J. Chem. Phys.*, 1997, **107**, 3032–3041.

[24] A. V. Marenich, C. J. Cramer and D. G. Truhlar, *J. Phys. Chem. B*, 2009, **113**, 6378–6396.

[25] S. Grimme, *Chem. Eur. J.*, 2012, **18**, 9955–9964.

[26] G. Luchini, J. Alegre-Requena, I. Funes-Ardoiz and R. Paton, *F1000Research*, 2020, **9**,.

[27] *CYLview Visualization Software, 1.0b; Legault, C. Y., Université de Sherbrook (2009)*, `http://www.cylview.org/`.

[28] A. J. Downs and C. R. Pulham, *Chem. Soc. Rev.*, 1994, **23**, 175–184.

[29] P. Erdmann, J. Leitner, J. Schwarz and L. Greb, *ChemPhysChem*, 2020, **21**, 987–994.

[30] P. Erdmann and L. Greb, *ChemPhysChem*, 2021, **22**, 935–943.

[31] F. Neese, *WIREs Comput. Mol. Sci.*, 2012, **2**, 73–78.

[32] F. Neese, *WIREs Comput. Mol. Sci.*, 2022, **12**, e1606.

[33] S. Grimme, J. G. Brandenburg, C. Bannwarth and A. Hansen, *J. Chem. Phys.*, 2015, **143**, 054107.

[34] S. Kozuch, D. Gruzman and J. M. L. Martin, *J. Phys. Chem. C*, 2010, **114**, 20801–20808.
